# Multicomponent Reactions Involving Diazo Reagents: A 5-Year Update

**DOI:** 10.3390/molecules26216563

**Published:** 2021-10-29

**Authors:** Olga Bakulina, Anna Inyutina, Dmitry Dar’in, Mikhail Krasavin

**Affiliations:** Institute of Chemistry, St. Petersburg State University, 26 Universitetskii Pr., 198504 Peterhof, Russia; ann.inyutina@gmail.com (A.I.); d.dariin@spbu.ru (D.D.)

**Keywords:** diazo compounds, multicomponent, metal catalysis, cycloaddition, stereoselectivity, denitrogenation, heterocycles, quaternary carbon, asymmetric

## Abstract

This review summarizes recent developments in multicomponent reactions of diazo compounds. The role of diazo reagent and the type of interaction between components was analyzed to structure the discussion. In contrast to previous reviews on related topics mostly focused on metal catalyzed transformations, a substantial amount of organocatalytic or catalyst-free methodologies is covered in this work.

## 1. Introduction

Multicomponent reaction (MCR) is a chemical transformation where three or more compounds react in a highly selective manner to form complex organic molecules in a convergent way. While three- and four-component reactions are the most common, there have been reported examples of up to nine-component ones [1]. The main advantages [2] of MCRs over traditional multi-step assembly are atom-economy, cost-effectiveness, and easy access to structural diversity which makes this strategy one of the most powerful instruments for medicinal chemistry. Although the first MCRs were discovered more than 150 years ago (Strecker synthesis, 1851) they are still heavily investigated [3].

Diazo compounds, in turn, are reagents with unique and surprisingly diverse reactivity modes, including transformations with both the retention of nitrogen atoms and the elimination of dinitrogen during their thermal, catalytic, or photolytic activation. They find their application in obtaining a variety of privileged in drug design motifs, such as a wide range of heterocyclic systems [4] and spirocyclic scaffolds [5], as well as in the synthesis of natural compounds [6] and the modification of biomolecules [7]. Given the vast and varied synthetic applications of diazo reagents, it is not surprising that they have found their implementation in MCRs. Nowadays, exploring the potential of diazo compounds for multicomponent approach is one of the most rapidly developing areas and this review is aimed at summarizing these recent achievements.

To the best of our knowledge, all recently published reviews on related subjects were focused on some specific aspects of diazo MCRs only—Rh- [8], Fe- [9] or general transition metal catalysis [10], asymmetric synthesis [11], reactions of ylides [12], and reactions of diazo carbonyl compounds [8]. In this work we aimed to present a comprehensive review on all novel diazo MCRs regardless of the structure of reagents or type of catalysis. It was found that a substantial amount of organocatalytic and catalyst-free methodologies have emerged recently.

In this review we primarily analyzed the type of MCR by the mode in which the components are combined, at which step the diazo reagent becomes involved and which type of intermediate is formed—stable or reactive. Another point of our classification was the mechanism of the key step in the MCR: e.g., “cycloaddition”, “radical reaction”, “CH-activation”, etc. Most articles discussed in this review have appeared within the last five years, although some relevant works from 2015 (not covered in the most recent review on the subject [4]) were also included.

## 2. Reactions Based on Generation of Stable Intermediates. Other Components React to Generate a New Substrate First, Followed by Reaction with Diazo Compound

### 2.1. Generation of Imine/Iminium

**Scheme 1:**
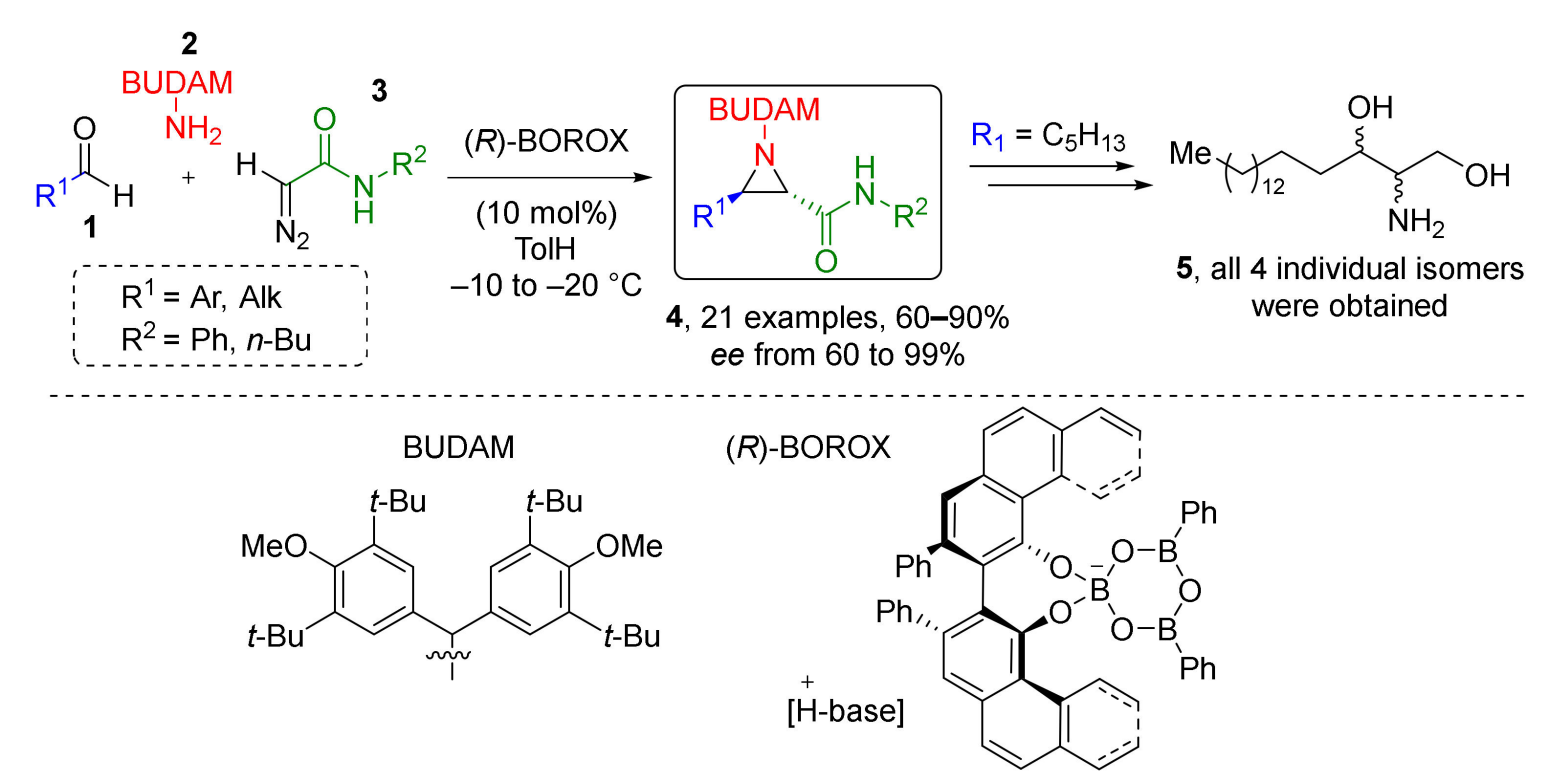
Asymmetric synthesis of *trans*-aziridines and synthesis of sphinganine isomers.

This type of MCR was found extremely useful for the preparation of 3-7 membered heterocycles. For the synthesis of aziridines, a series of Aza-Darzen reaction-based methodologies have been developed. For example, aziridine-3-carboxamides **4** were prepared using BOROX-type catalyst (chiral boronic ester) from a series of aldehydes **1**, BUDAM-amine **2** (BUDAM: 3,5-di-*tert*-butyldianisylmethyl), and diazo acetamides **3** (Scheme 1) [13]. To achieve full stereoselectivity, a bulky substituent (BUDAM) was introduced into the amine component. Aziridine assembly was carried out at low temperatures and did not require pre-reacting the catalyst, the aldehyde, and the amine, so the diazo compound could be added directly into the reaction mixture. The reaction showed remarkable tolerance to functional groups in the aldehyde component (e.g., isocyanide, oxirane, alkyne, NHPG were tolerated). Obtained products were smoothly deprotected with TfOH to deliver NH-aziridines. This methodology was applied in the total synthesis of all four stereoisomers of naturally occurring aminodiol sphinganine **5**.

**Scheme 2:**
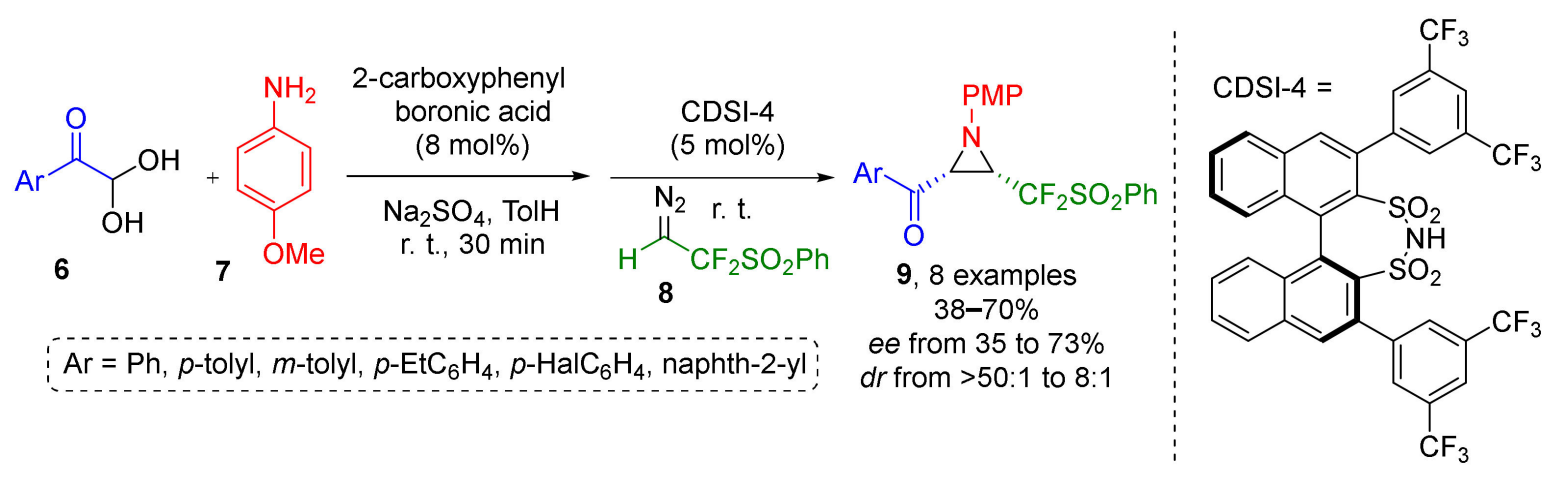
Asymmetric synthesis of CF_2_-functionalized aziridines.

An important extension to this methodology by introduction of fluorinated substituents to aziridines was reported in 2020 by J.-A. Ma and co-authors. Fluorinated diazo reagent **8**, was reacted with imines generated from *p*-anisidine **6** and aroyl heminal diols **7** to give aziridines **9** with fluorinated side chain (Scheme 2) [14]. Combination of a strong Brønsted acid (disulfonimide with axial chirality CDSI-4) and boronic acid was used to achieve diastereo- and enantioselectivity but with corresponding excess values were only moderate (up to 73%ee). Noteworthy, both protocols employ organocatalysis.

**Scheme 3:**
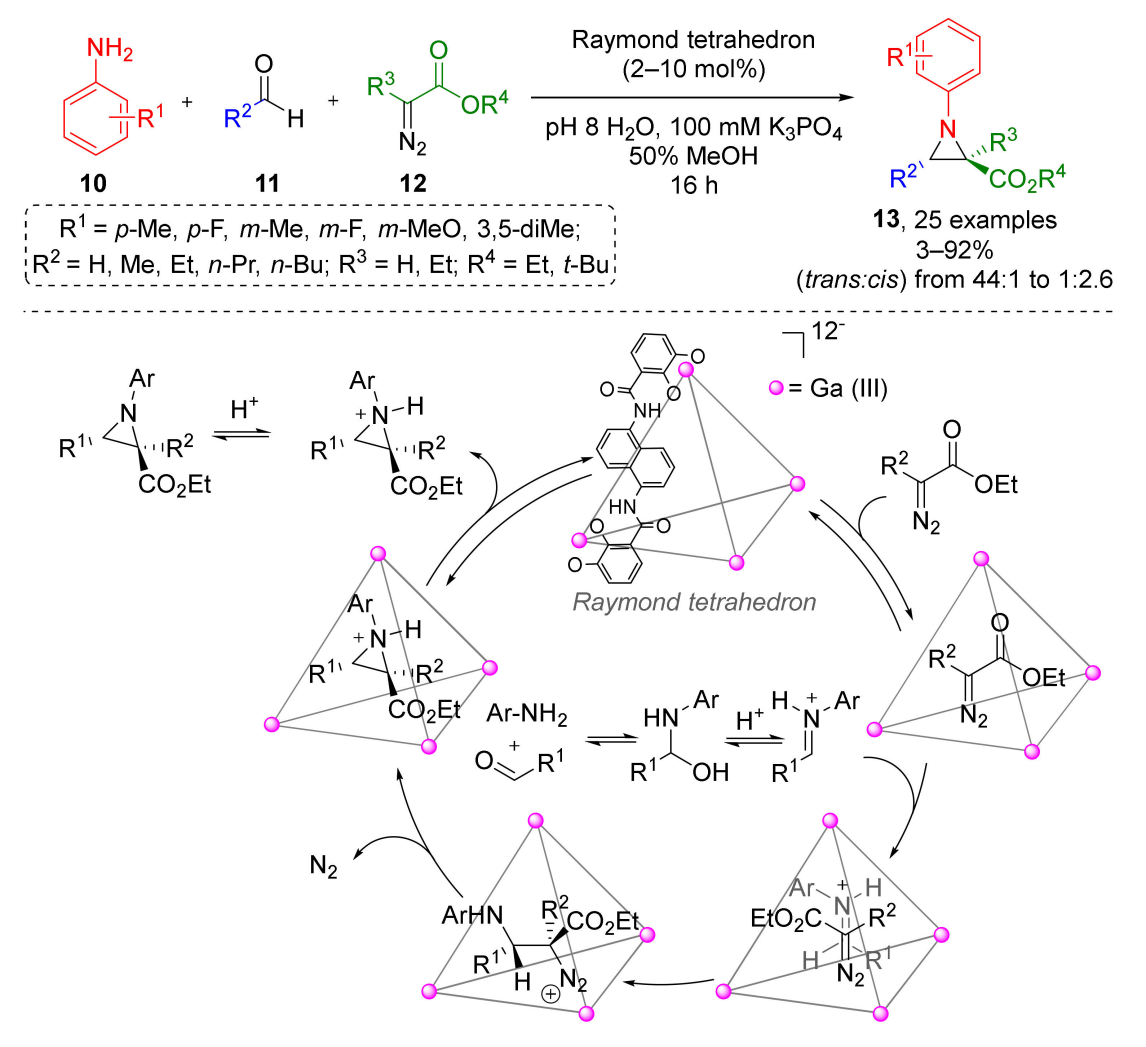
Nanovessel-catalyzed three-component Aza-Darzens reaction.

In 2020, the Raymond group reported similar reaction driven by supramolecular catalysis (Scheme 3) [15]. A large series of *N*-aryl substituted aziridine-2-carboxylates **13** was prepared from anilines **10**, aliphatic aldehydes **11** (including formaldehyde), and alkyl diazoacetates **12** under mild conditions (aqueous MeOH at r. t.). The authors developed and applied a very unusual catalyst—gallium(III) nanovessels (so-called “Raymond tetrahedron”), which allowed inversing the usual diastereoselectivity (trans vs. cis) via formation of host-guest complexes. Kinetic studies (NMR) showed that aniline or aldehyde do not bind to the catalyst and formation of imine proceeds outside the host molecule. In contrast, diazo compound formed host-guest complex readily. Encapsulation of iminium ions was shown to be rate-determining. Expectedly, the size of the host cavity is a limiting factor and therefore bulky anilines were found to be unreactive.

**Scheme 4:**
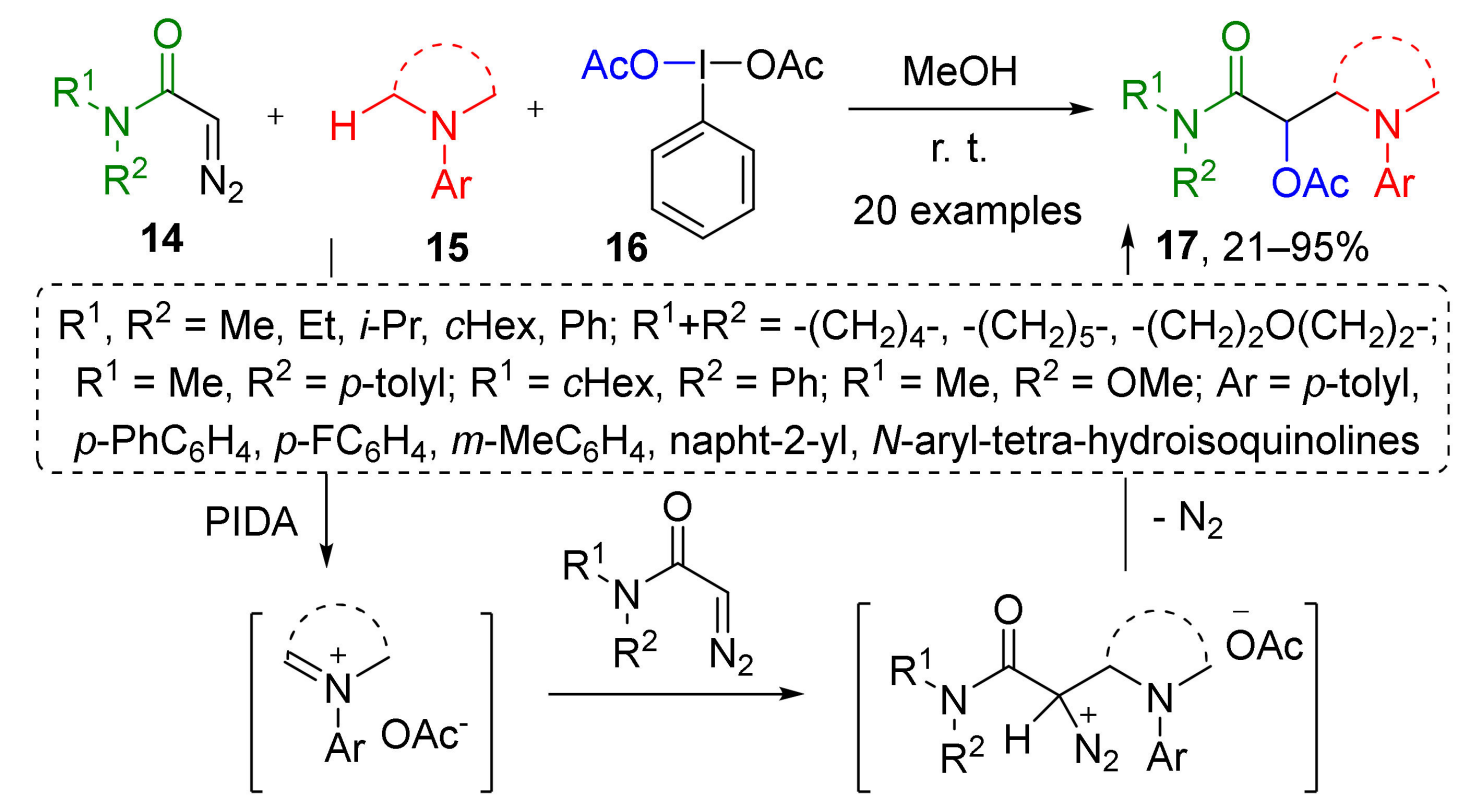
Acetoxyaminoalkylation of α-diazo amides.

Several catalyst-free methodologies involving iminium species have also been developed, which is much less common. In the first one, diazo acetamides **14** were involved in a reaction with iminium acetate which was generated by oxidation of *N*-substituted anilines **15** by PIDA **16** (phenyliododiacetate), a hypervalent iodine compound (Scheme 4) [16]. This approach has another remarkable feature—iminium generation by oxidation instead of much more common condensations. The developed protocol delivered both cyclic and acyclic products **17** (derivatives of α-acetoxy-β-aminopropionic acid) depending on substation pattern in aniline. Interestingly, the third component contributing to the structure of product—acetoxy group comes from the same reagent (PIDA), therefore it has a double function.

**Scheme 5:**
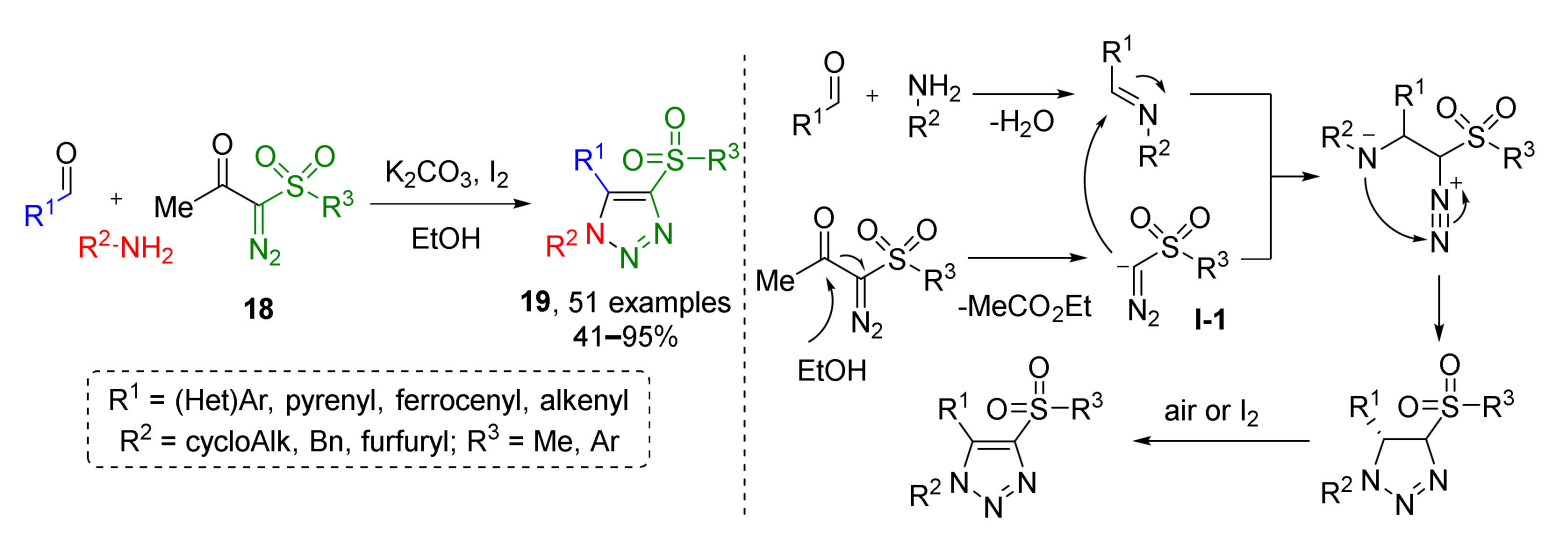
Metal-free three-component assembly of fully substituted 1,2,3-triazoles.

Another metal-free protocol was developed for the preparation of trisubstituted 1,2,3-triazoles **19** from imines and diazo ketosulfones **18** (Scheme 5) [17]. Initially generated triazolines were oxidized by iodine in a one-pot fashion. At first, diazo ketosulfones were deacylated under basic conditions to form terminal diazo sulfones in situ which, in turn, attack imines with nucleophilic carbon atoms to give intermediate diazo compound **I-1**. The latter undergoes addition of the former imine nitrogen atom at the diazo group which results in the formation of the triazoline cycle. The reaction was found to be very general and high-yielding except for when aliphatic substituents at the sulfur atom are used.

**Scheme 6:**
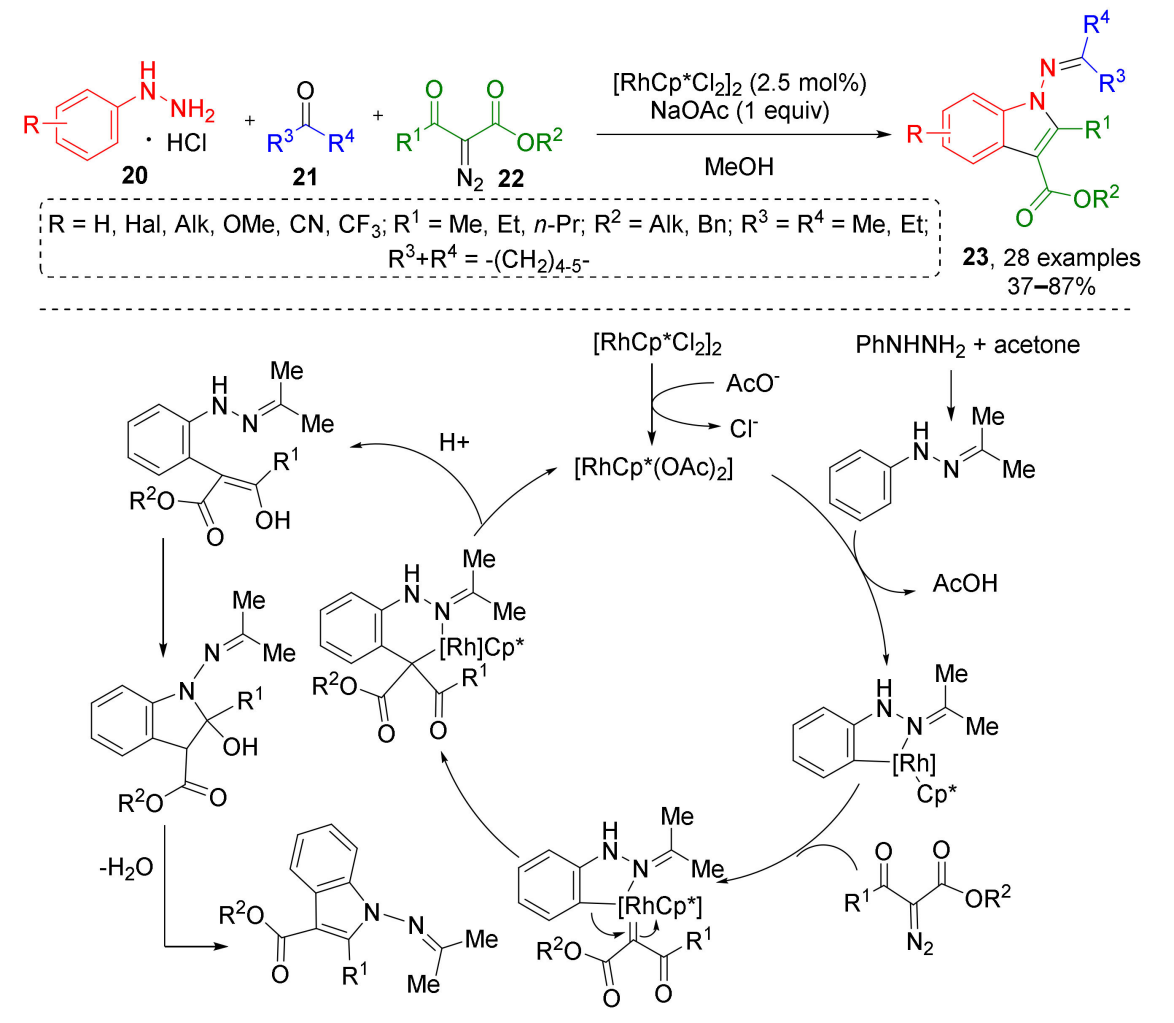
Synthesis of 1-iminoindoles via Rh(III)-catalyzed three-component annulation.

An interesting example of Rh-catalyzed indole synthesis was reported by X. Cui in 2017 (Scheme 6) [18]. Aryl hydrazines **20** reacted with ketones **21** and diazo ketoesters **22** to provide rare *N*-iminoindoles **23**. Reaction pathway included formation of imine, followed by CH-activation and cyclometallation, generation of Rh-carbenoid after addition of diazo compound with subsequent aryl migration, protonolysis, and intramolecular condensation.

**Scheme 7:**
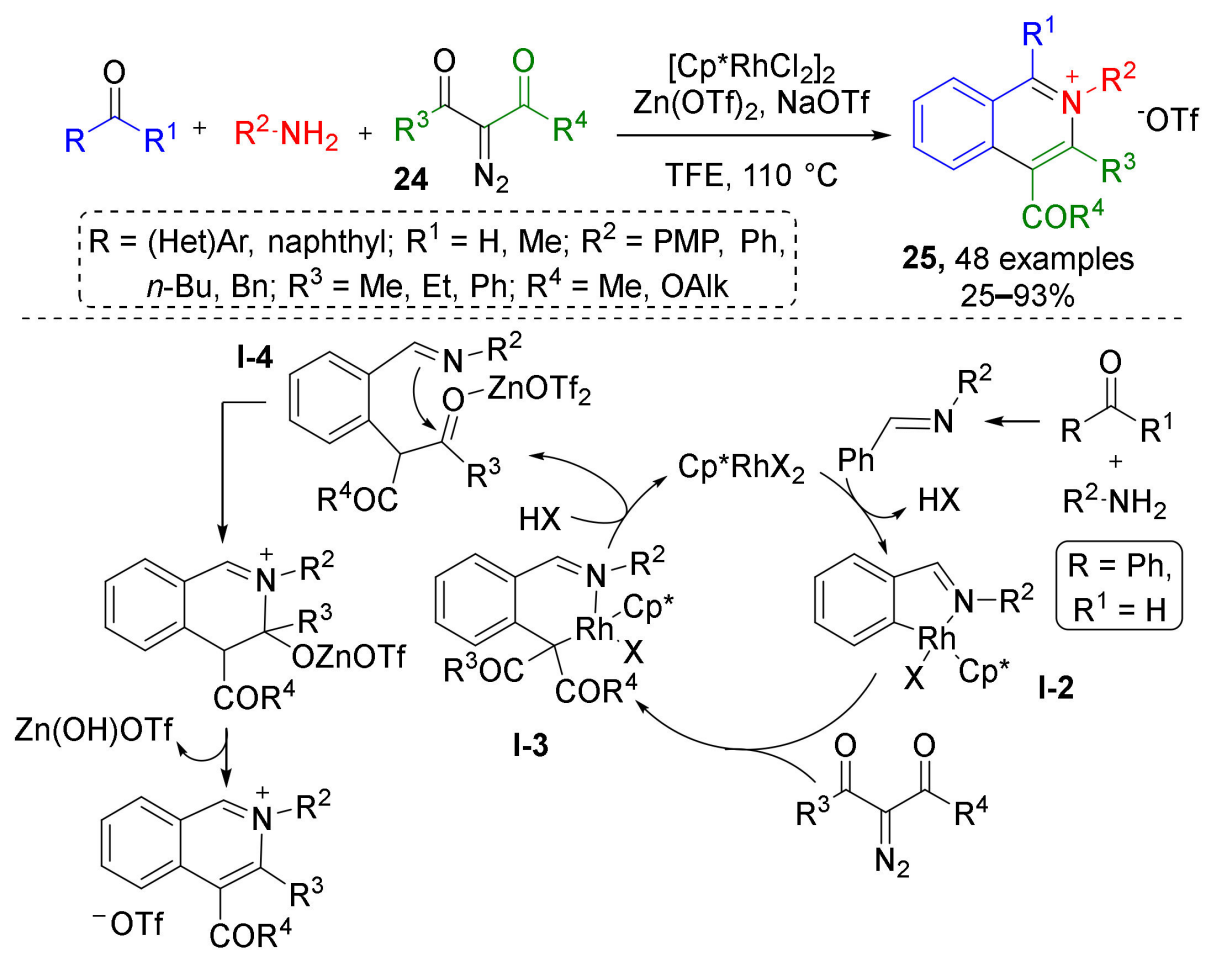
Rhodium(III)-catalyzed redox-neutral synthesis of isoquinolinium salts.

Based on similar methodology, a novel azine synthesis has also been developed. In 2018, X. Li et al. showed that synthetically challenging tetrasubstituted isoquinolinium salts **25** can be prepared from diazo ketoesters **24** and imines generated in situ under mixed Rh(III)/Zn(II) catalysis (Scheme 7) [19]. This reaction mechanistically resembles the previous case—it begins with CH-insertion of Rh in ortho-position of imine arylidene group (intermediate **I-2**) followed by addition of diazo compound and formation of Rh-carbenoid. Migratory insertion of the Rh-aryl bond to the carbene then delivers a Rh(III) alkyl intermediate **I-3**, which releases Rh after protonation giving intermediate **I-4**. Its keto group is then activated with Lewis acid (Zn(OTf)_2_, taken in equimolar amount) for nucleophilic attack with imine nitrogen. This cyclization is the final step on the way to isoquinolinium salts **25**. The reaction mechanism was thoroughly investigated and supported by X-ray studies (rhodacyclic intermediate **I-2** was isolated).

**Scheme 8:**
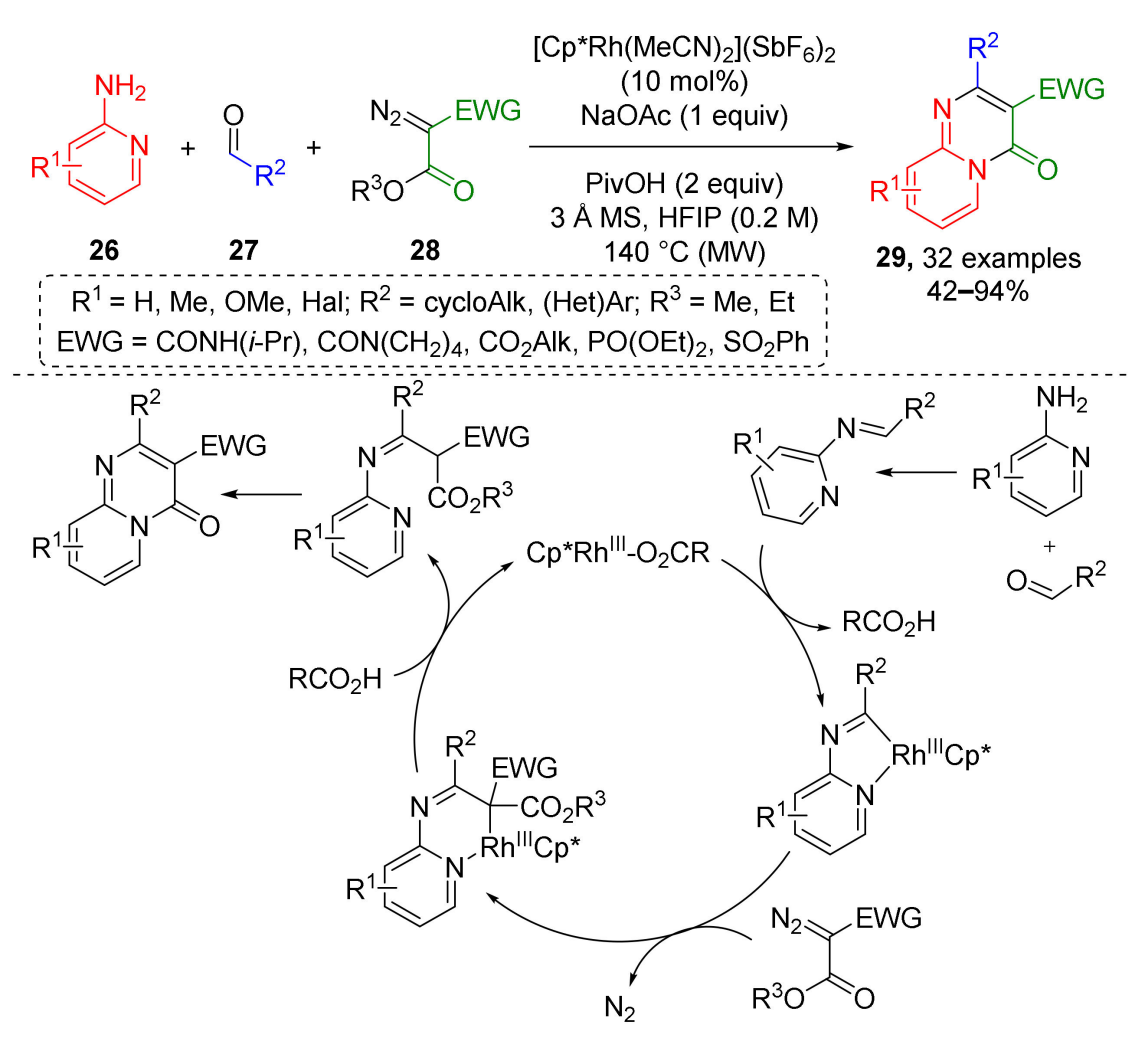
Synthesis of pyridopyrimidinones via Rh(III)-catalyzed imidoyl C−H activation.

Another type of Rh-mediated CH activation was employed to build an azine-fused heterocyclic system. Reaction of 2-aminopyridines **26**, aldehydes **27**, and α-acyl diazo esters **28** provided a series of pyrido[1,2-*a*]pyrimidin-4-ones **29** (Scheme 8) [20]. This transformation also included a cyclometallation step but another type of CH-bond was activated—in imine moiety, not aryl. Reaction was carried out at high temperature (140 °C) under MW irradiation. A broad spectrum of diazo compounds was successfully used—diakyl diazo malonates, carbamoyl-, sulfonyl-, and phosphoryl diazo esters. The nature of substituents in two other components—aldehydes and amines showed no significant impact to the reaction outcome making this protocol very general.

**Scheme 9:**
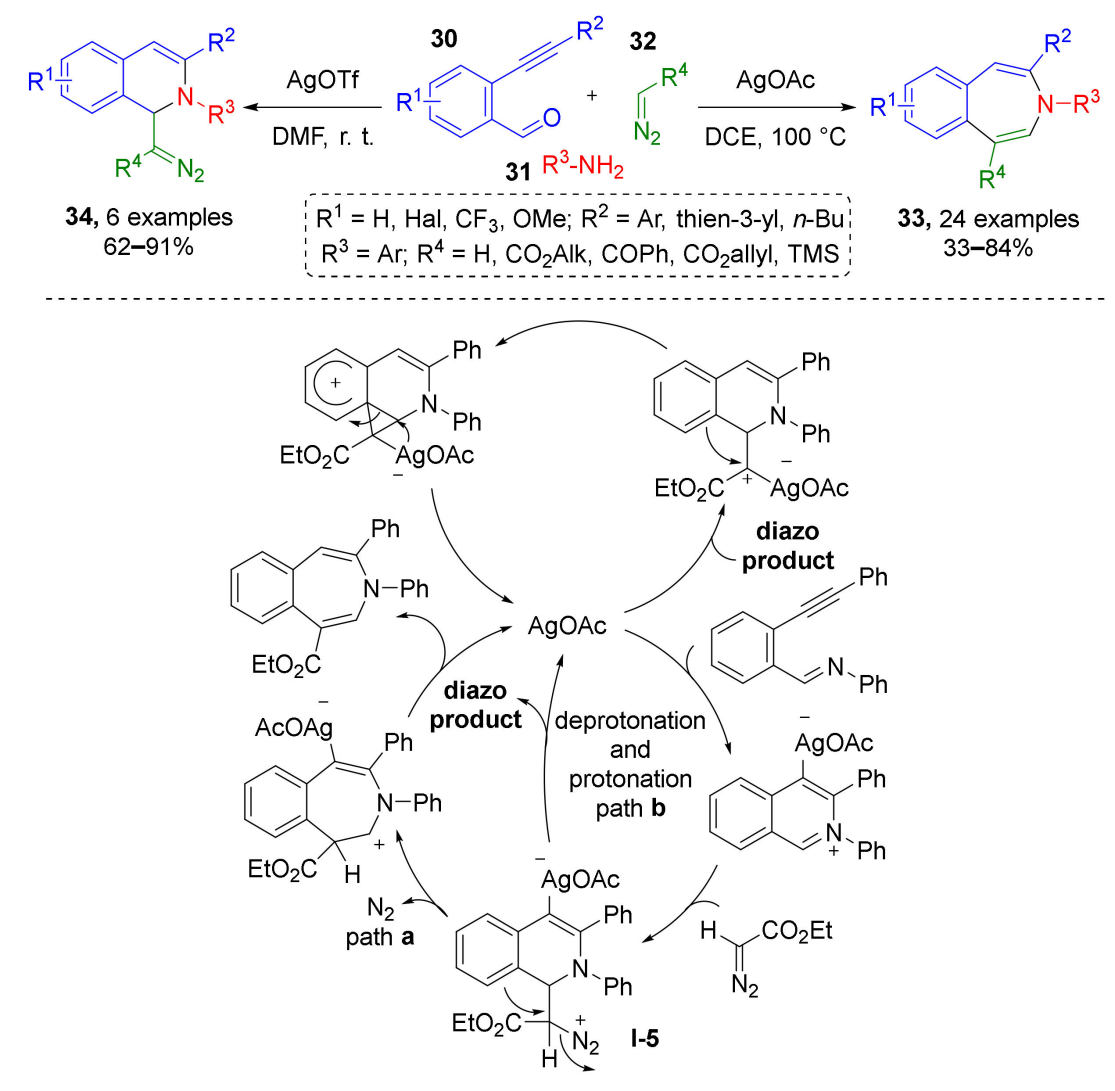
Ag(I)-catalyzed synthesis of 3-benzazepines and diazo-containing dihydroisoquinolines.

In 2015 L. Zhou and co-authors reported a novel approach towards seven-membered heterocycles using diazo-imine MCR. Furthermore, 3-Benzazepines **33** were obtained via a complex cascade of transformations: at first iminium substrate (*N*-alkylisoquinolinium) was generated from 2-formylphenylacetylenes **30** and anilines **31** via condensation and silver-catalyzed (AgOAc) imine−yne cyclization, which is a rare type (Scheme 9) [21]. According to one of the two mechanisms proposed by authors, this reaction was followed by addition of iminium to diazo compound **32** (terminal diazo esters or TMS-diazomethane), formation of dihydroisoquinolinic diazo intermediate **I-5** and 1,2-aryl migration with ring expansion and nitrogen extrusion. Noteworthy, catalyst screening revealed that another silver source (AgOTf) can interrupt the last two steps, thus intermediate dihydroisoquinoline-derived diazo compounds **34** can be isolated.

**Scheme 10:**
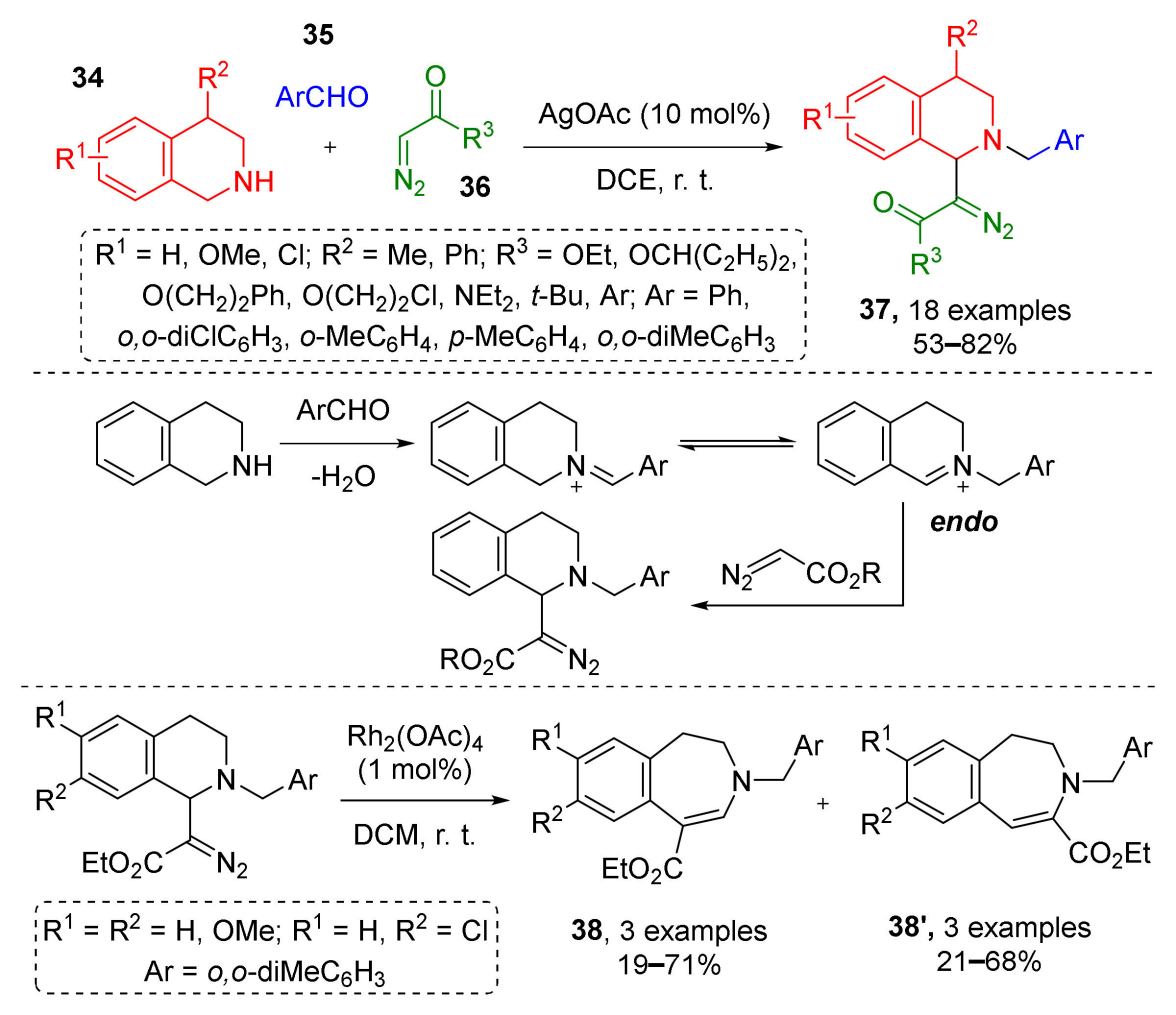
Functionalization of tetrahydroisoquinolines with diazo carbonyl compounds.

The same group later described a similar three-component protocol where iminium was generated via condensation of NH-tetrahydroisoquinoline **34** and aromatic aldehydes **35** followed by silver-catalyzed (AgOAc) nucleophilic addition of terminal diazo esters or amides **36** (Scheme 10) [22]. Obtained tetrahydroisoquinolinic diazo compounds **37** were isolated and applied in rhodium-catalyzed synthesis of dihydrobenzazepines **38** and **38′**. The latter were formed as isomeric mixtures due to the following two types of transformations of the Rh-carbenoid—classic 1,2-aryl- or unexpected 1,2-*N* migration.

### 2.2. Alkene/Alkyne-Based MCRs

**Scheme 11:**
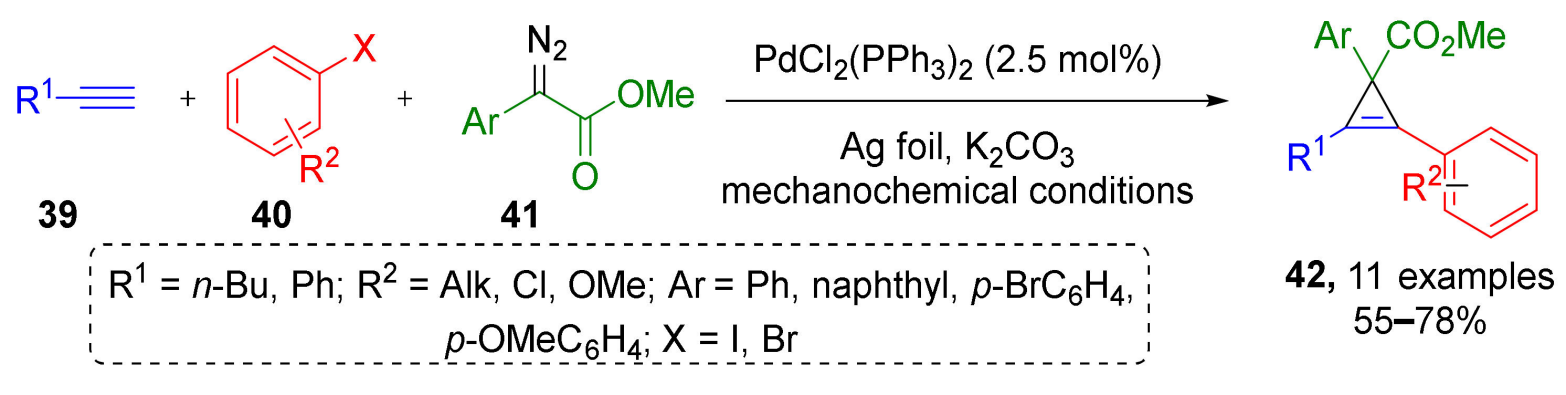
Cyclopropene synthesis via domino Sonogashira coupling/cyclopropenation.

In this subsection we will discuss MCRs where a substrate with unsaturated C-C bond is generated in situ to react with diazo compound via cycloaddition reaction. In 2018, J. Mack et al. reported a three-component protocol, which combined preparation of internal acetylenes via Sonogashira reaction of terminal acetylenes **39** and aryl halides **40** followed by cyclopropenation with diazo esters **41** (including terminal ones) under dual Pd/Ag or Pd/Cu catalysis and solvent-free conditions—ball milling (Scheme 11) [23]. Using this methodology, a series of tetrasubstituted cyclopropenes **42** was prepared in high yields. Both aliphatic and aromatic terminal acetylenes were involved successfully.

**Scheme 12:**
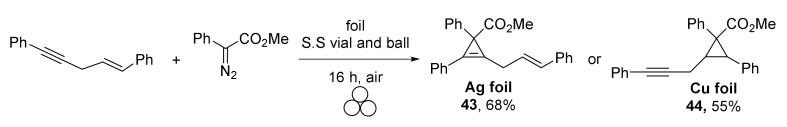
Chemoselectivity of cyclopropa(e)nation of enynes under Ag(0) and Cu(0) catalysis.

Interestingly, the authors were able to switch chemoselectivity in the case of enynes by applying silver or copper foil: the triple bond was involved in the reaction under Ag catalysis giving product **43** (Scheme 12), while copper activated the double bond selectively (cyclopropane **44** was isolated in this case).

**Scheme 13:**
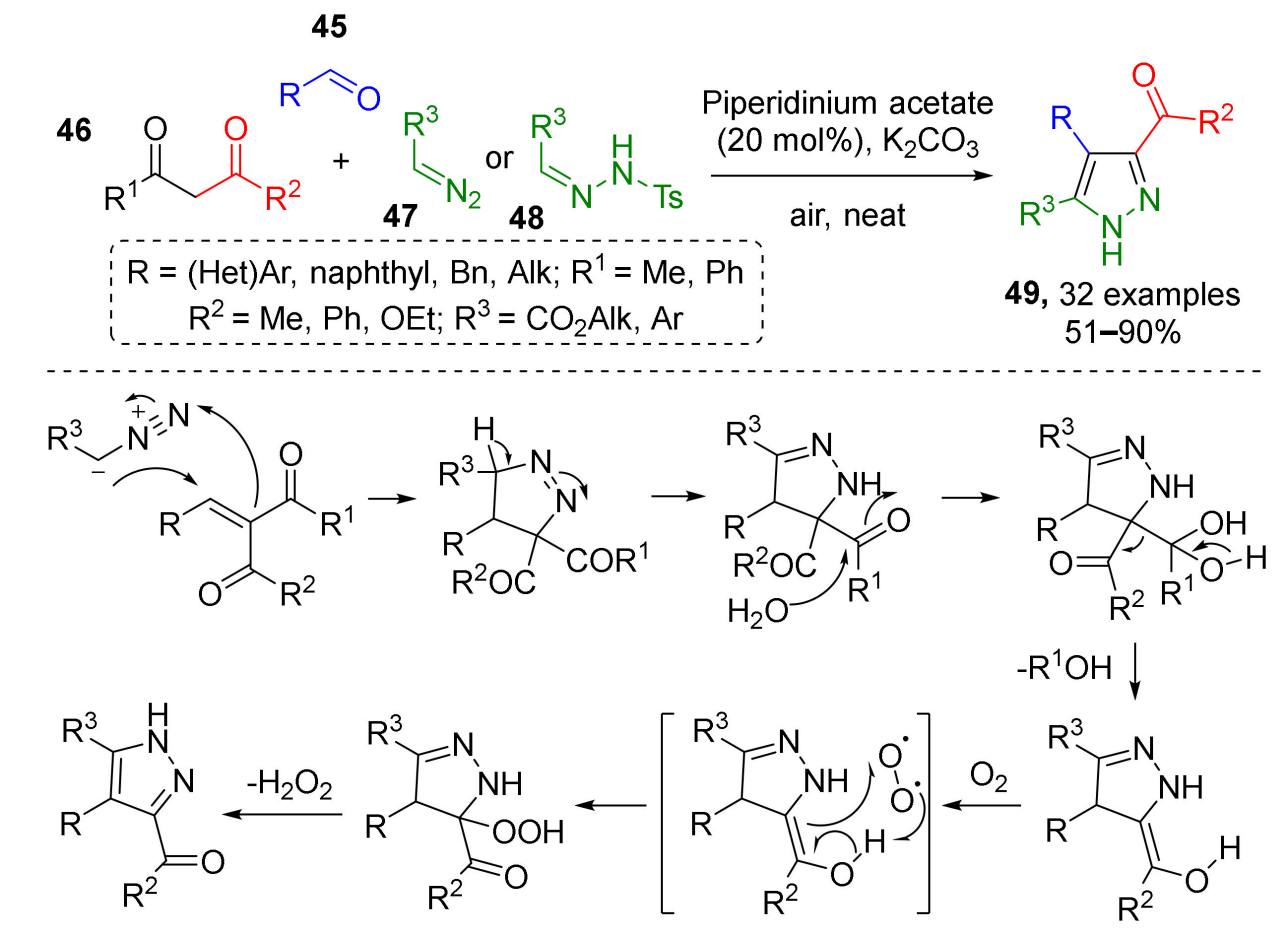
Three-component approach to the synthesis of 3,4,5-trisubstituted pyrazoles.

In situ generation of alkenes and subsequent 1,3-dipolar cycloaddition was shown to be an efficient strategy for the preparation of pyrazoles. In one such protocol, reported by S. A. Maurya et al., alkenes were generated in situ via the Knoevenagel condensation of aldehydes **45** and 1,3-dicarbonyl compounds **46** (Scheme 13) [24]. Olefins then reacted with terminal diazo compounds **47** or their precursors, *N*-tosylhydrazones **48,** to provide 3,4,5-trisubstituted pyrazoles **49**. Reaction intermediates, pyrazolines, were aromatized by air-mediated oxidative elimination of acetyl group.

**Scheme 14:**
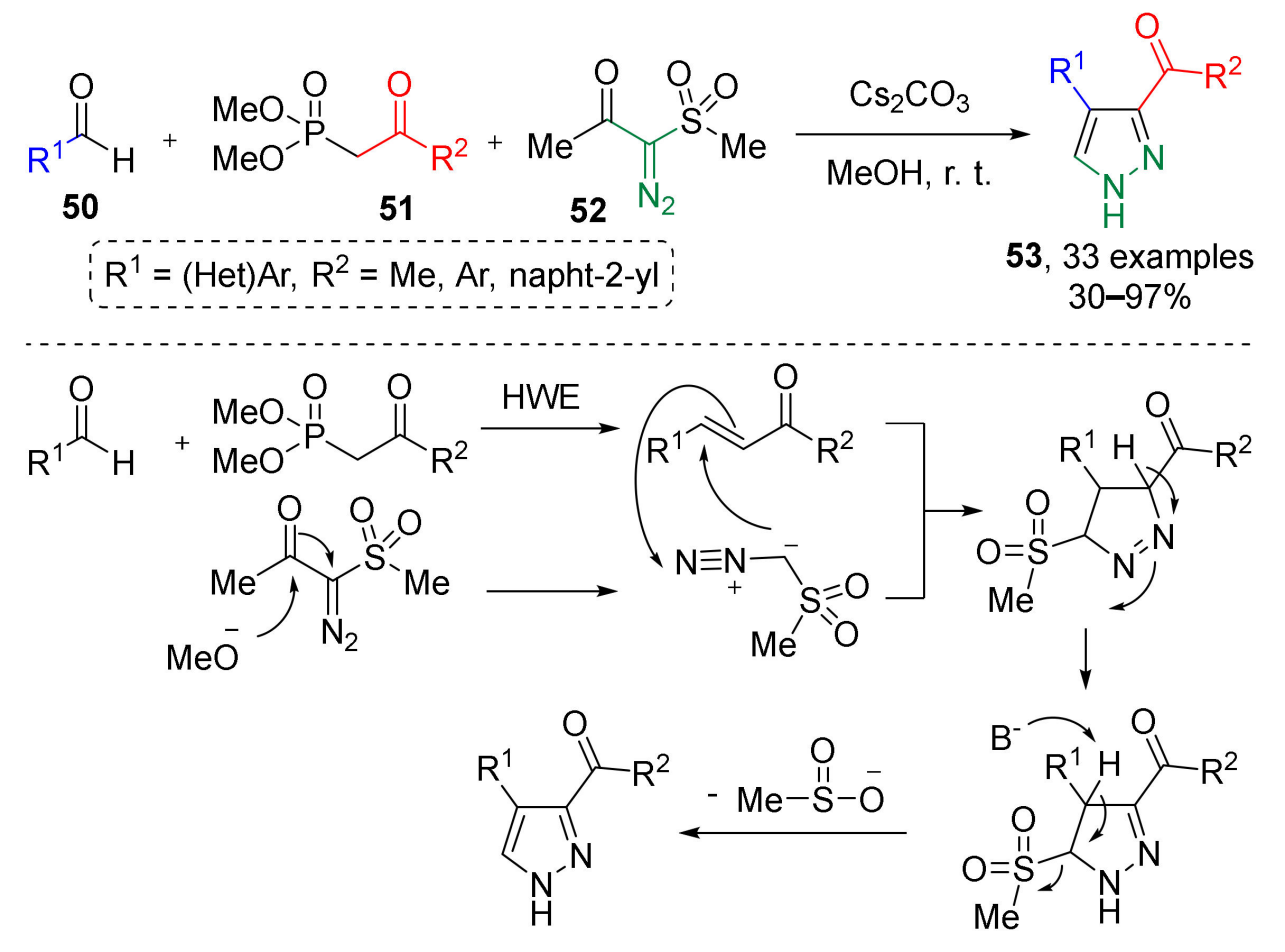
Synthesis of pyrazoles using diazo sulfone as a diazomethane surrogate.

Another type of pyrazoles **53**, namely, 3,4-disubstitued, were prepared via a similar approach reported by K. Mohanan and co-authors in 2017 (Scheme 14) [25]. In this case alkene was generated via Horner–Wadsworth–Emmons reaction of aromatic or heteroaromatic aldehydes **50** and ketophosphonates **51**. The third reaction partner, terminal diazo sulfone, was also generated in situ—by base-promoted diacylation of corresponding diazo keto sulfone **52**. Initial cycloaddition product, trisubstituted sulfonyl pyrazoline, was aromatized via desulfonylation reaction with elimination of MeSO_2_H.

### 2.3. MCRs Based on Generation of Other Types of Stable Intermediates

Judging by the number of relevant publications, imine-based MCRs involving diazo compounds can be considered as the most explored at the moment. Among other types of MCR involving generation of stable organic intermediates amine- and carbamate-based ones can be mentioned.

**Scheme 15:**
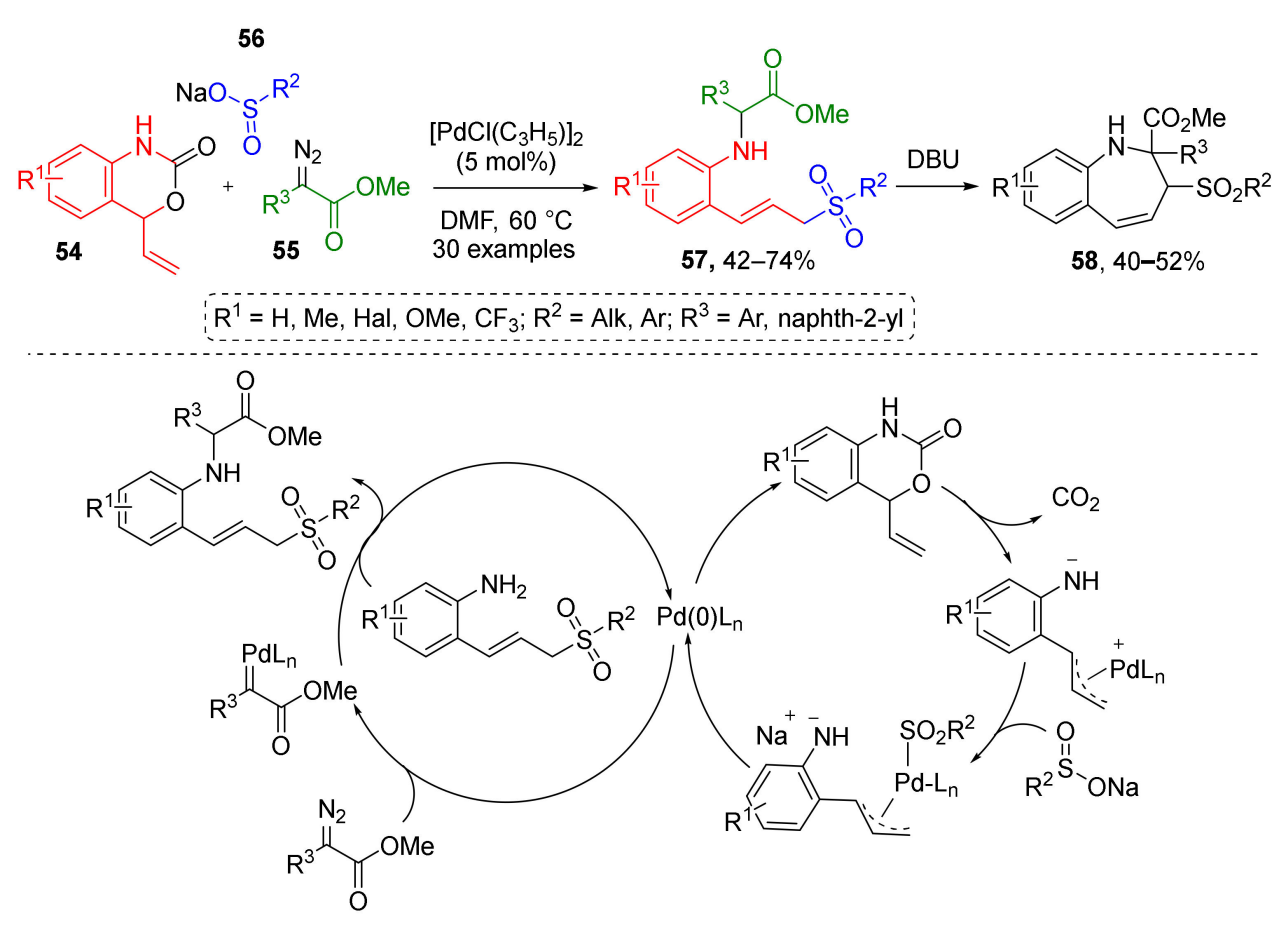
Pd-catalyzed synthesis of allylic sulfone-containing amino acid derivatives.

The first type was employed by W. Yang and co-authors in 2018 for Pd-catalyzed preparation of *N*-2-(3-sulfonylallyl)aryl substituted amino acid derivatives **57** from vinyl benzoxazinones **54**, diazo esters **55**, and aryl or alkylsulfinates **56** (Scheme 15) [26]. Benzoxazinones gave rise to 2-allylanilines via CO_2_ extrusion, followed by addition of sulfonate to form 2-(3-sulfonyl-prop-1-en-1yl)anilines which reacted with diazo compounds via NH-insertion. These products were further employed in the preparation of dihydro-1*H*-benzo[*b*]azepines **58** via base-promoted dehydrogenative cyclization.

**Scheme 16:**
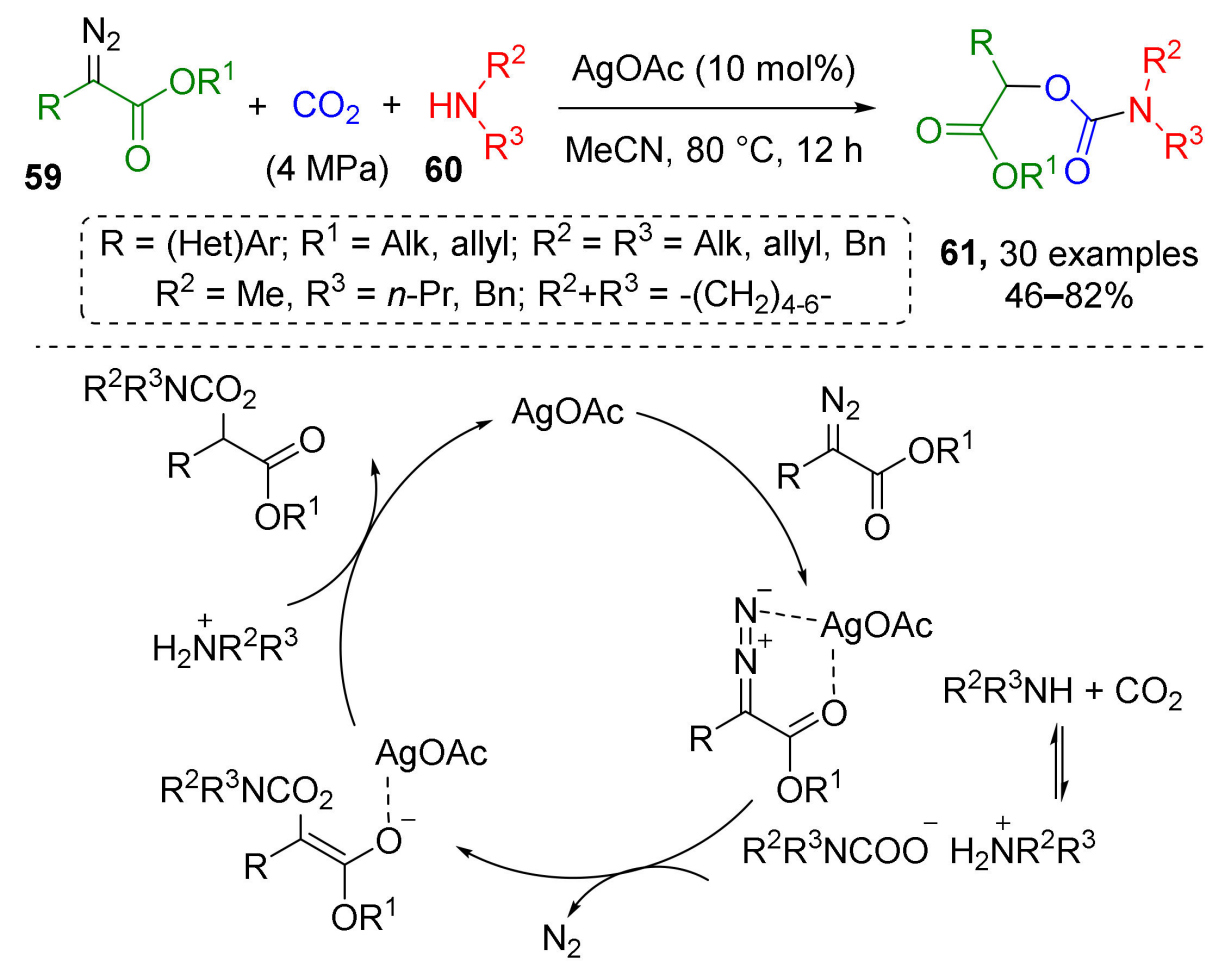
Silver-catalyzed coupling of carbon dioxide, amines, and α-diazoesters.

A carbamate-based MCR was reported in 2018 by H. Jiang and co-authors who showed that silver-catalyzed reaction of *N*,*N*-dialkylcarbamates **59** and α-diazoesters **60** opens facile access to α-carbamoyloxy esters **61** (Scheme 16) [27]. Carbamates were generated in situ from both cyclic and acyclic amines and CO_2_ gas (4 MPa) in a steel autoclave. The final reaction step was denitrogenative addition of *O*-nucleophilic center of carbamate to carbon atom of diazo group.

**Scheme 17:**
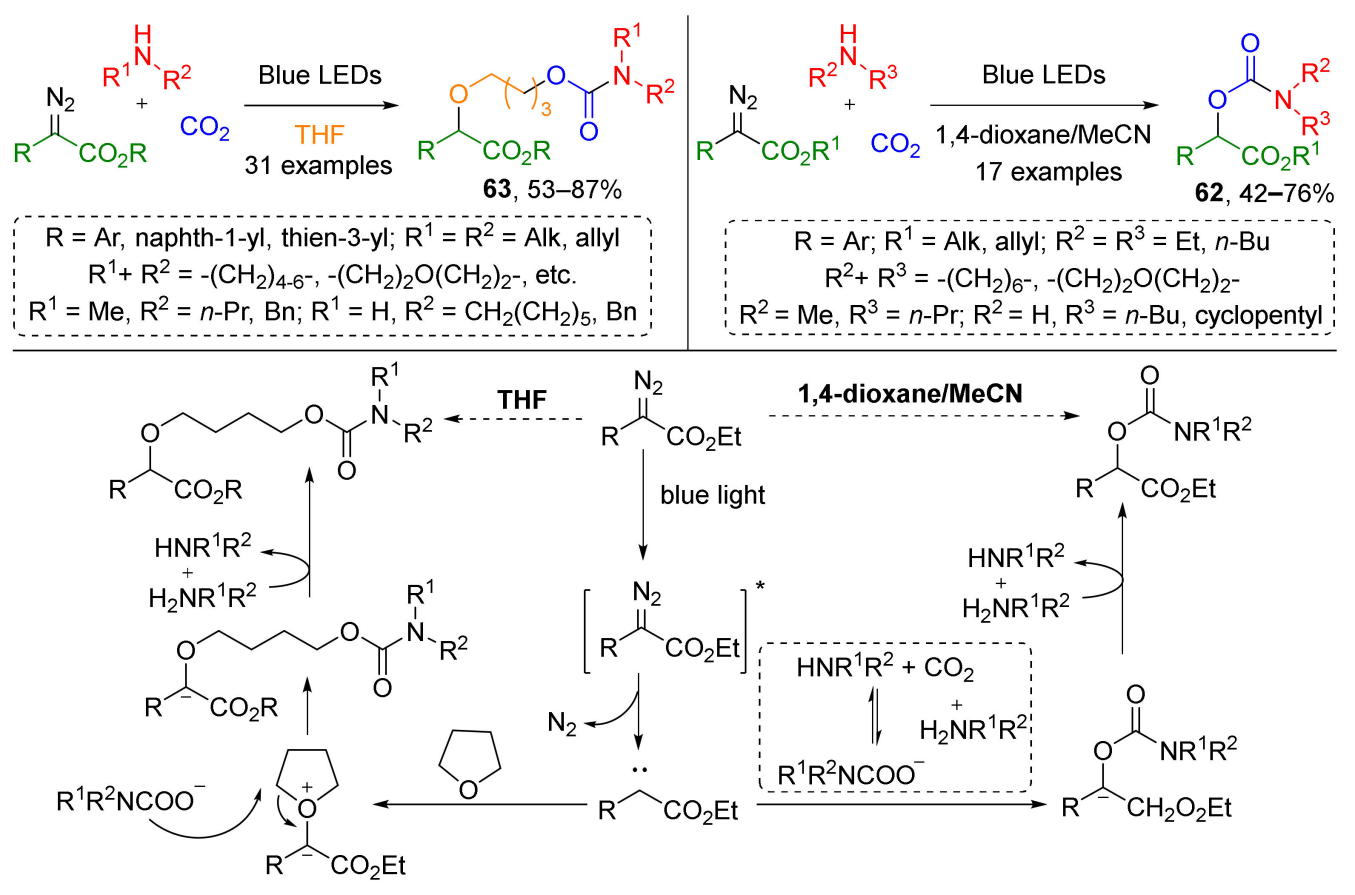
Visible light-promoted synthesis of organic carbamates from carbon dioxide.

The same group recently reported a substantial improvement of this methodology—a catalyst-free visible light-driven protocol for the preparation of compounds **62** (Scheme 17) [28]. One of its important advantages is the use of CO_2_ at atmospheric pressure. Interestingly, when THF was used as solvent, a four-component coupling reaction occurred giving product with additional 1-butoxy linker between carbamoyl and arylacetate moieties giving products **63**. In this case, THF presumably reacted with carbene generated photolytically from diazo compound giving oxonium ylide, which further underwent a nucleophilic attack by the carbamate anion.

**Scheme 18:**
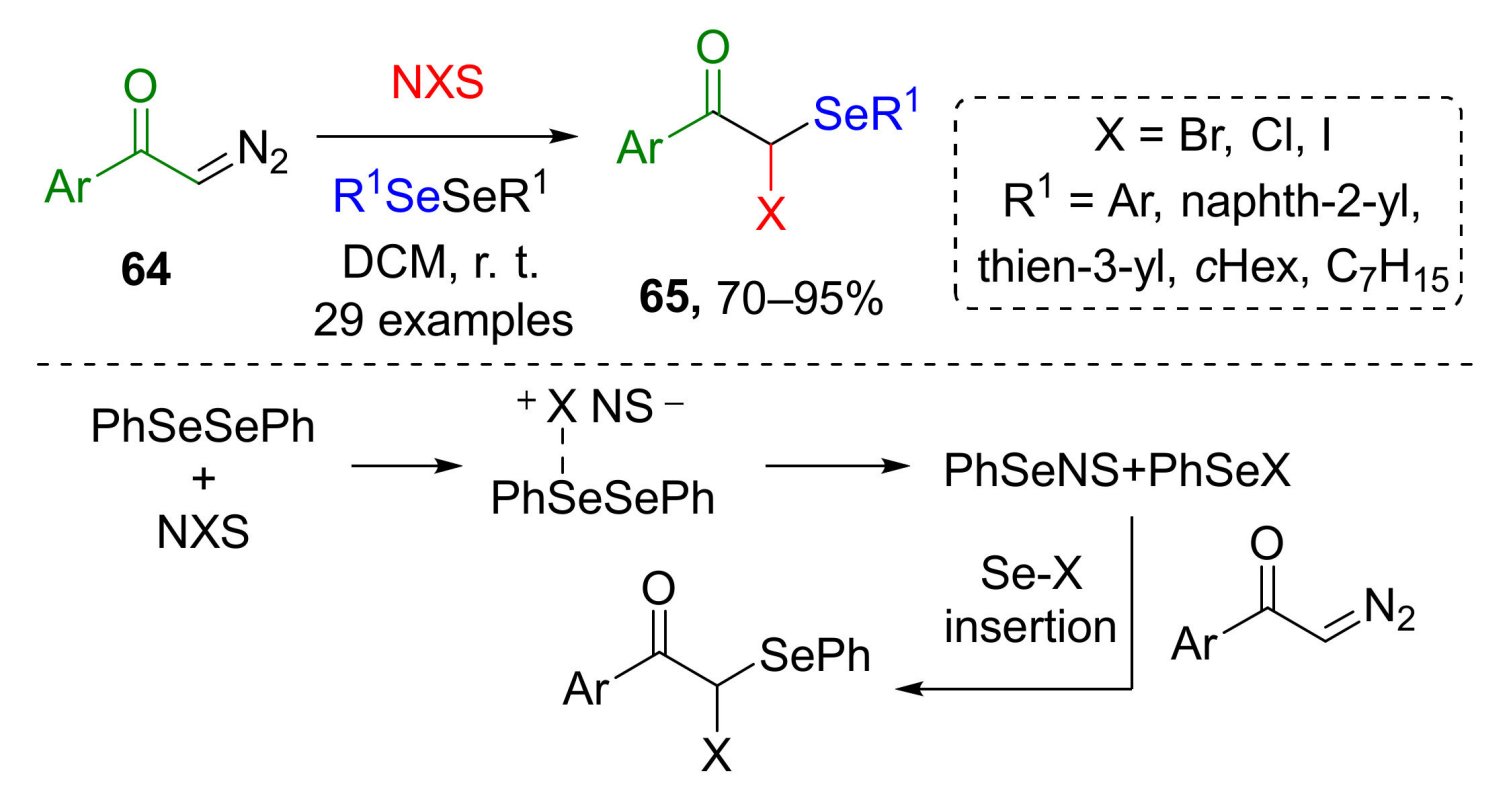
Difunctionalization of α-diazoarylketones with diaryldiselenides and *N*-halosuccinimides.

The approach we would like to discuss last in this category was based on in situ generation of organoselenium compound (ArSeBr) from aryl diselenide and *N*-halosuccinimides NXS (Scheme 18) [29]. The third component of this MCR was terminal diazo ketones **64**, which underwent *gem*-difunctionalization with introduction of SeAr and X group via Se-X insertion to provide compounds **65**. Reaction scope was limited to aryl substituents both in diazo and organoselenium compound.

## 3. MCRs Where One of the Reaction Components Is a Reactive Intermediate Generated from Diazo Compound

### 3.1. Generation of Ylides Followed by Reaction with Carbonyl Compounds

**Scheme 19:**
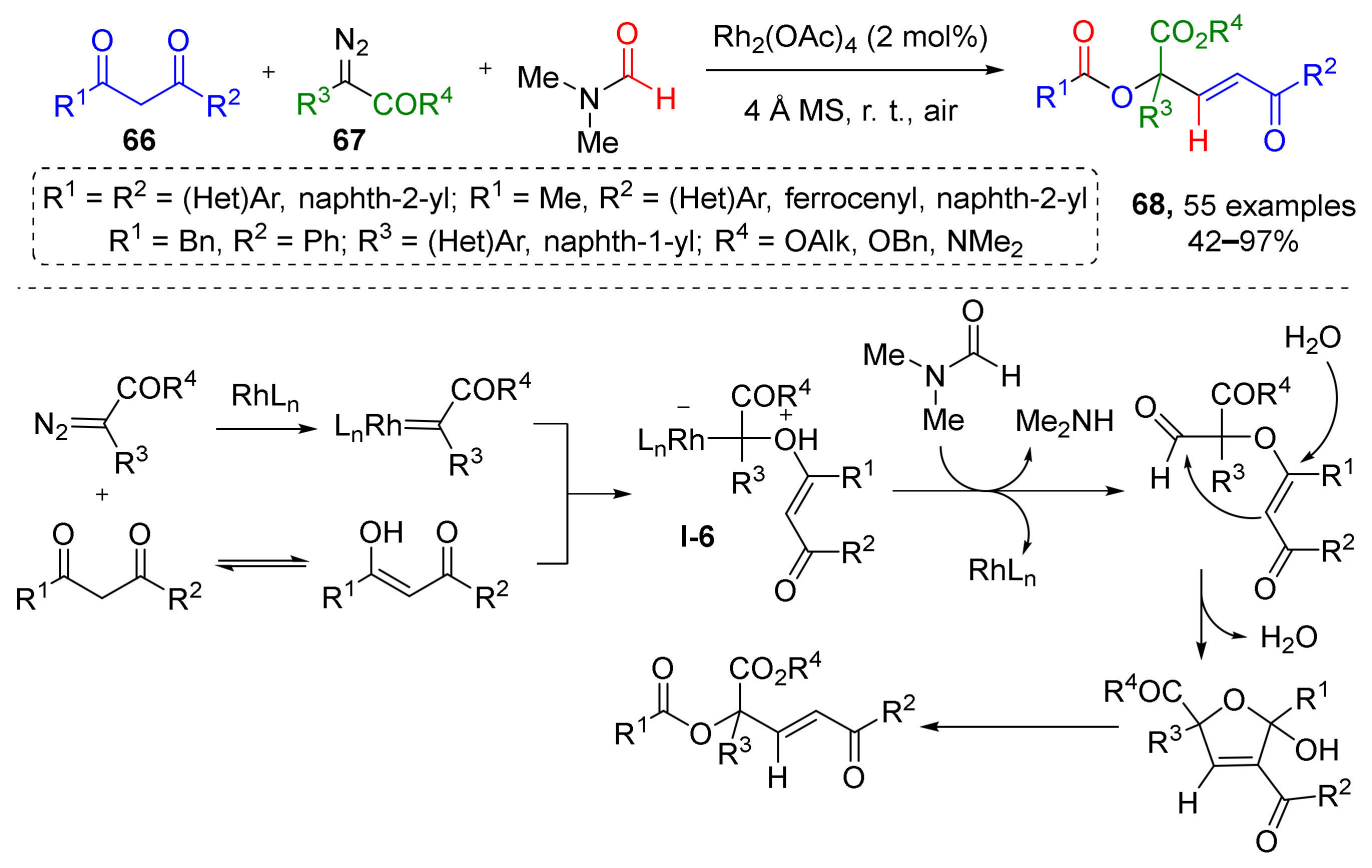
Rhodium(II)-catalyzed multicomponent assembly of α,α,α-trisubstituted esters.

Reactions where diazo compounds react with alcohols or ethers to form oxonium ylides via formal nucleophilic substitution of diazo group are one of the most common. Recently, this methodology was extended by three-component reaction of 1,3-diones **66**, diazo esters **67**, and *N*,*N*-dimethylformamide (DMF), leading to an unusual formal insertion of O–C(sp3)–C(sp2) into unstrained C(CO)−C bonds and preparation of compounds **68** (Scheme 19) [30]. The reaction was conducted in DMF in the presence of rhodium tetraacetate and molecular sieves at room temperature. This protocol was applied to obtain a large series (50+ compounds) of previously inaccessible α,α,α-trisubstituted esters or amides. The key step of this transformation was Rh-catalyzed formation of oxonium ylide **I-6** from enol tautomer of diketone and diazo compound. Mechanistic study involving isotopic labeling showed that O, C(sp3), and C(sp2) units of the product originate from 1,3-diones, diazo esters, and DMF, respectively.

**Scheme 20:**
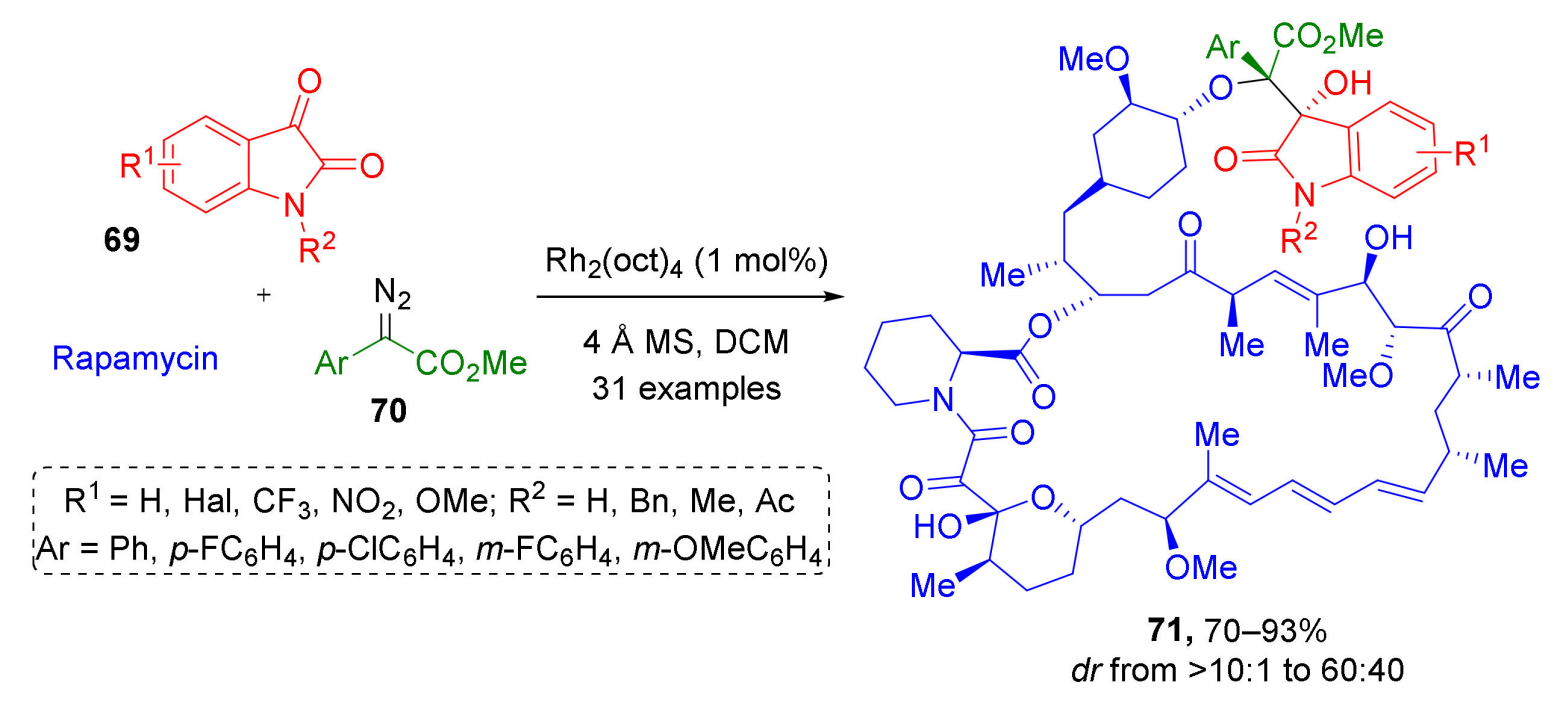
Semisynthesis of rapamycin analogues through trapping of oxonium ylides.

This type of reaction has also been used in the synthesis of complex biologically active compounds. For example, in 2019, natural immunosuppressant rapamycin was regio- and diastereoselectively modified using substituted isatins **69** and α-aryl-α-diazoacetates **70** to produce a series of forty novel indoline-substituted analogs **71** (Scheme 20) [31]. The obtained compounds were tested against several cancer cell lines and were found to be promising anticancer drug leads.

**Scheme 21:**
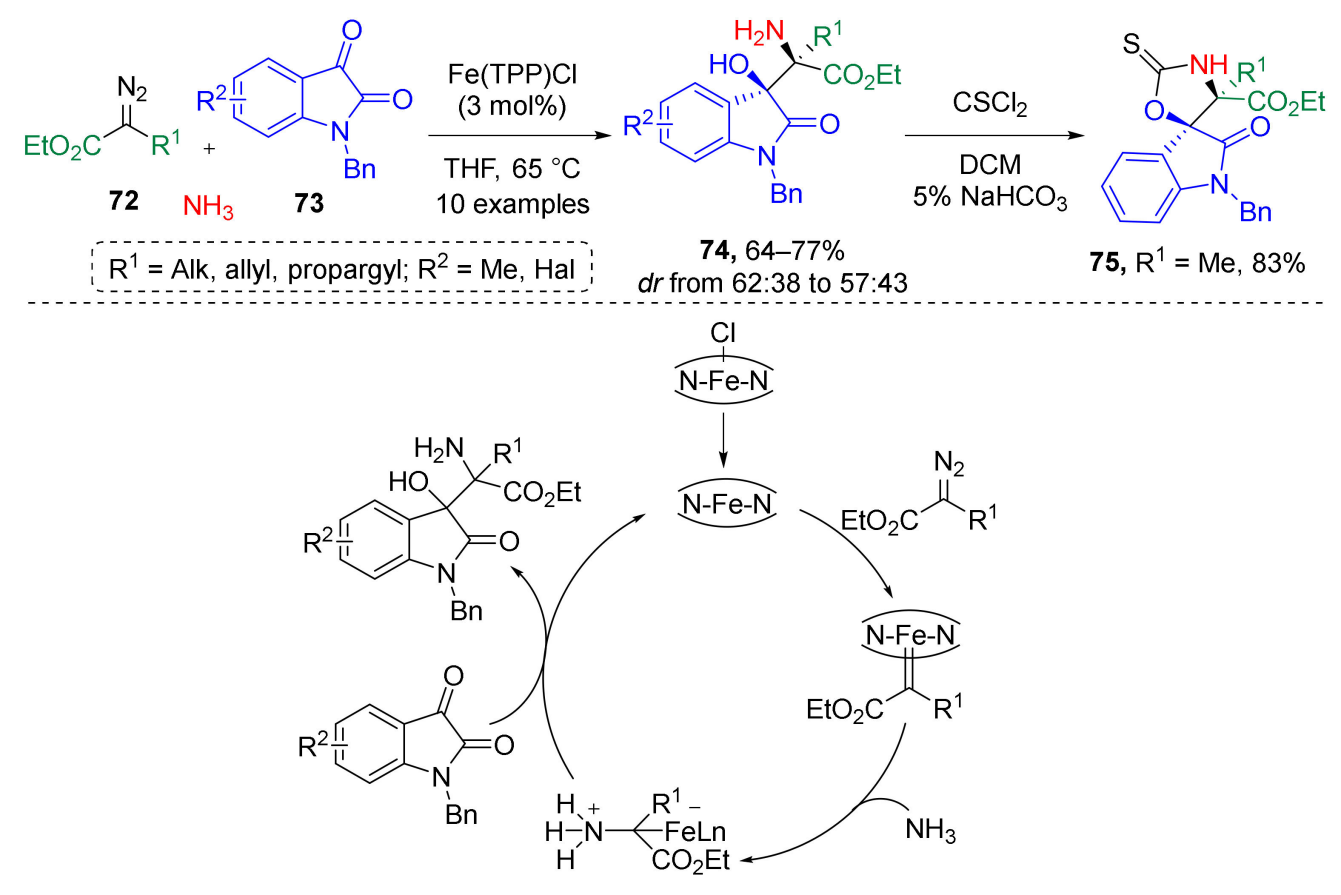
Iron-catalyzed three-component reaction of alkyl diazo esters, isatins, and ammonia.

In addition, several examples of a similar strategy based on the generation of ammonium ylides have been reported recently. In 2017, W. Hu et al. developed an iron-catalyzed three-component reaction of alkyl diazo esters **72**, isatins **73**, and ammonia which provided access to non-protected β-hydroxy-α-aminoesters with adjacent quaternary stereocenters **74** (Scheme 21) [32]. This transformation was achieved via generation of ammonium ylide from a diazo compound, ammonia, and Fe-porphyrin catalyst followed by nucleophilic addition to 3-carbonyl group of isatin. The reaction was conducted in refluxing THF. The authors also showed synthetic application of obtained product in the preparation of 3-spirooxindole **75** via cyclization with thiophosgen.

**Scheme 22:**
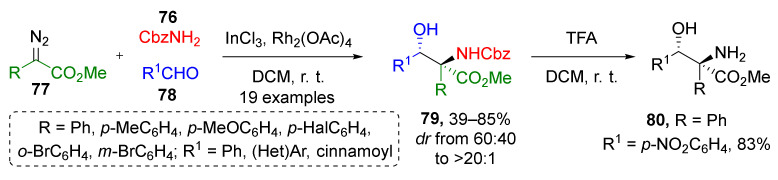
Co-catalyzed diastereoselective trapping of carbamate ammonium ylides with aldehydes.

The same group previously reported another interesting MCR where ammonium ylides were generated from carbamate (Fmoc, Boc or CBz-NH_2_ (**76**)) and diazo esters **77** (Scheme 22) [33]. (Hetero)aromatic aldehydes **78** were used as the third component to provide β-hydroxy-α-amino acid derivatives **79** under dual Rh/In-catalysis. All products were isolated in high yields as single diastereomers under mild reaction conditions (DCM, room temperature). For one of the products, the possibility of *N*-deprotection was confirmed—Cbz group was smoothly removed by TFA to give compound **80**.

### 3.2. Generation of Ylides Followed by Reaction with Imine/Iminium

A substantial improvement of methodology for the preparation of chiral compounds with quaternary carbon asymmetric atoms has recently been made through reactions of ylides generated from diazo compounds with imines. A large variety of structurally diverse and complex α-hydroxy-β-amino-, α-sulfanyl-β-amino-, or α,β-diamino carbonyl compounds were prepared.

**Scheme 23:**
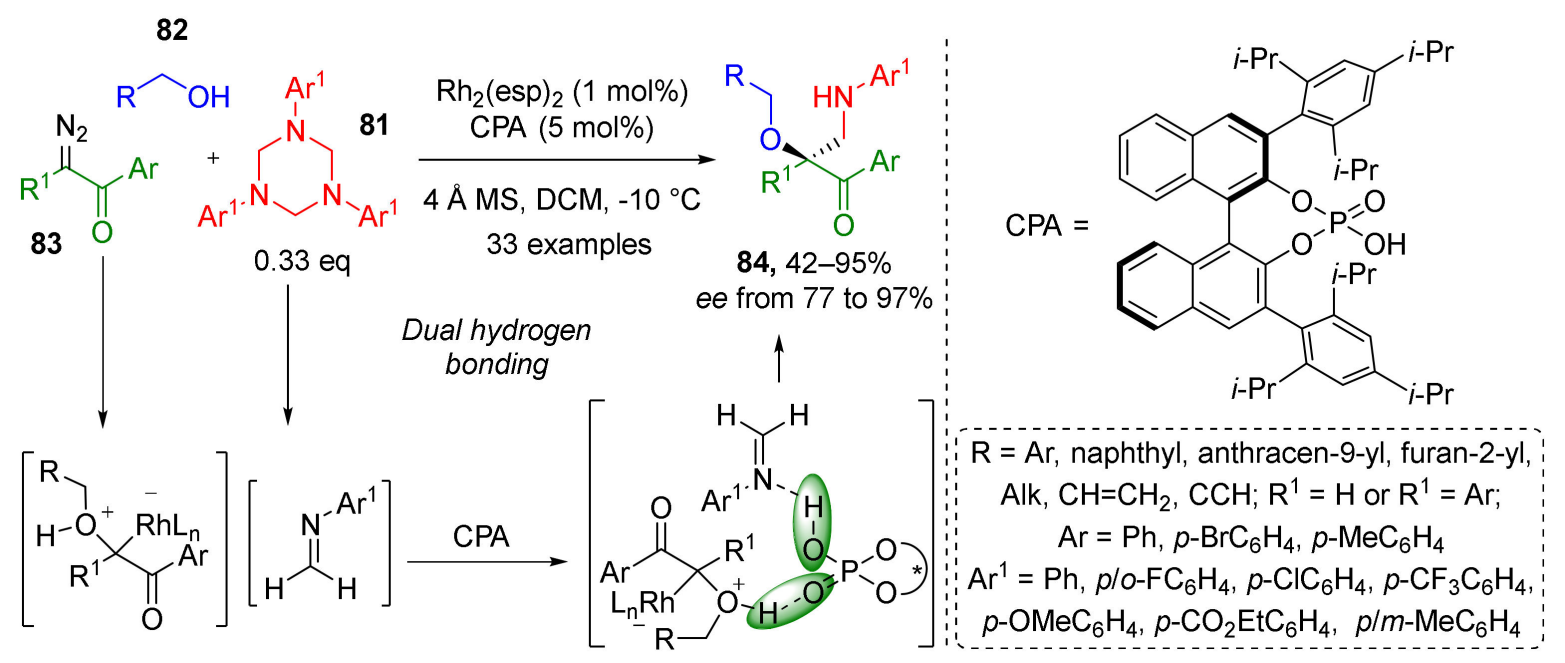
Three-component aminomethylation of α-diazo ketones with alcohols and 1,3,5-triazinanes.

In 2020, 1,3,5-triazinanes **81** were employed by W. Hu et al. as stable trimeric form of formaldehyde imines, which reacted with alcohols **82** and diazo acetophenones **83** to provide a series of substituted α-hydroxy-β-aminoacetophenones **84** (Scheme 23) [34]. A combination of rhodium espinoate and binaphthyl-based chiral phosphoric acid afforded target compounds not only in high yields and mild conditions but also with high enantioselectivity. The key reaction step was Rh-mediated formation of oxonium ylide from alcohol and diazo reagent.

**Scheme 24:**
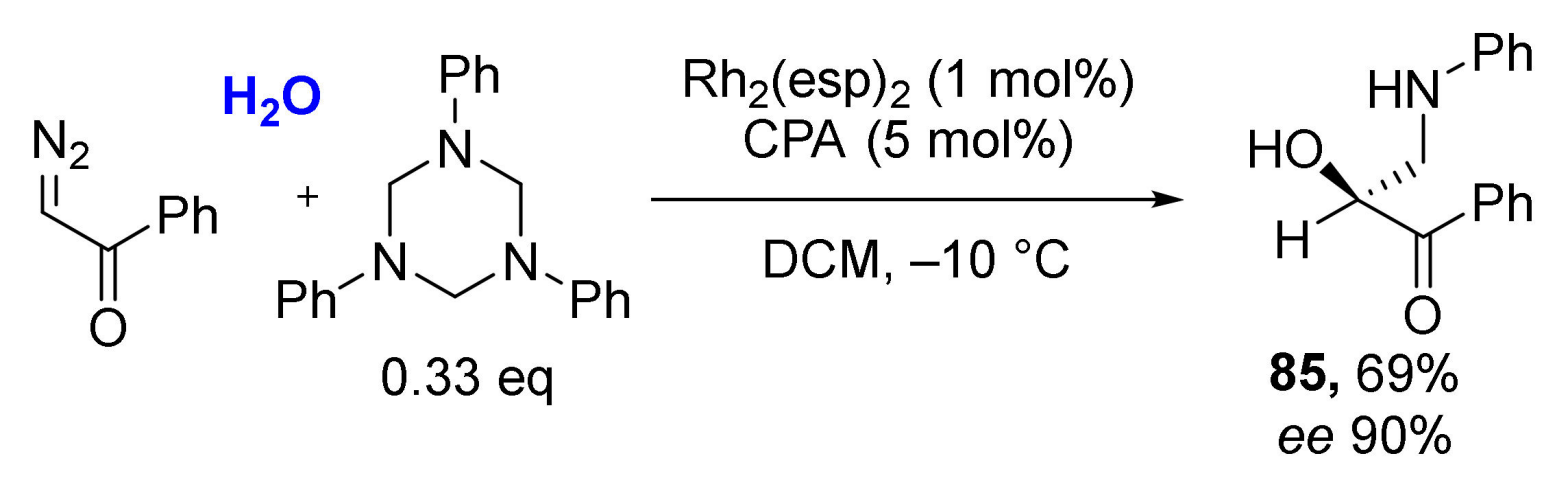
Preparation of *O*-unsubstituted α-hydroxy-β-aminoacetophenones from triazinanes. diazo ketones and water.

Even structurally complex natural alcohols (such as cholesterol, borneol, menthol, farnesol) were successfully involved into this MCR making a valuable instrument for medicinal chemistry (Scheme 24). An interesting observation was also made—that alcohols can be replaced with water molecules providing *O*-unsubstituted products **85** under the same reaction conditions.

**Scheme 25:**
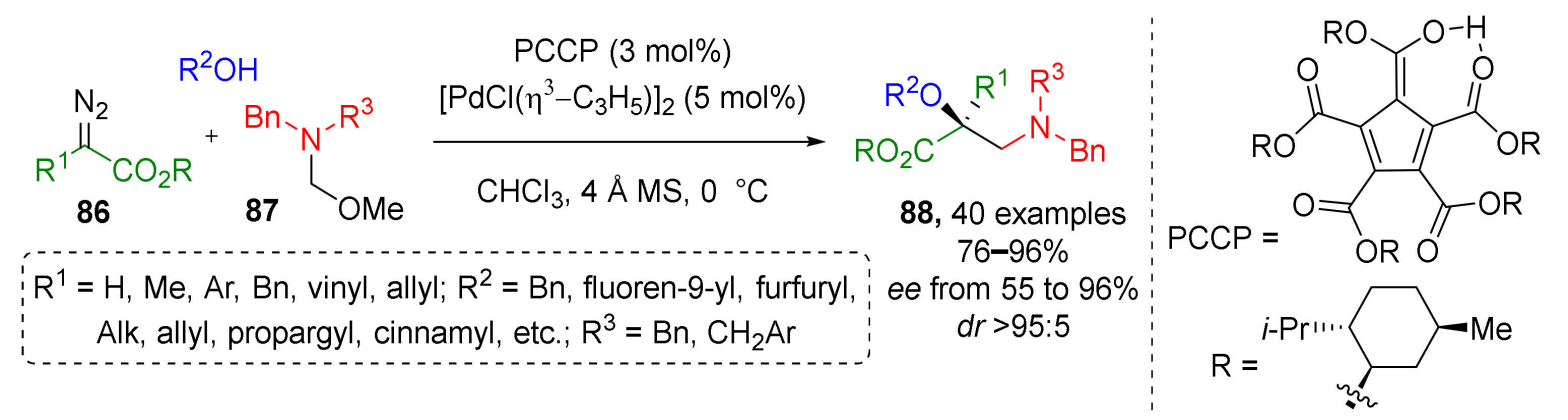
Synthesis of chiral β-amino acids via trapping of an enol intermediate.

One year earlier, the same group showed that structurally related *N*,*N*-disubstituted α-hydroxy-β-aminocarboxylic acids **88** can be also prepared following this strategy (Scheme 25) [35]. The main difference was using α-aminomethyl ether **86** as the methylene iminium ion precursor and diazo esters **87** (not ketones) as the second component. High enantioselectivity in this case required a complex recently (2016) developed ligand—chiral menthol ester of pentacarboxycyclopentadiene (PCCP) acid. Notably, in this work a series of quantum chemical calculations was performed to rationalize the reaction mechanism, choice of chiral ligand, and stereoselectivity.

**Scheme 26:**
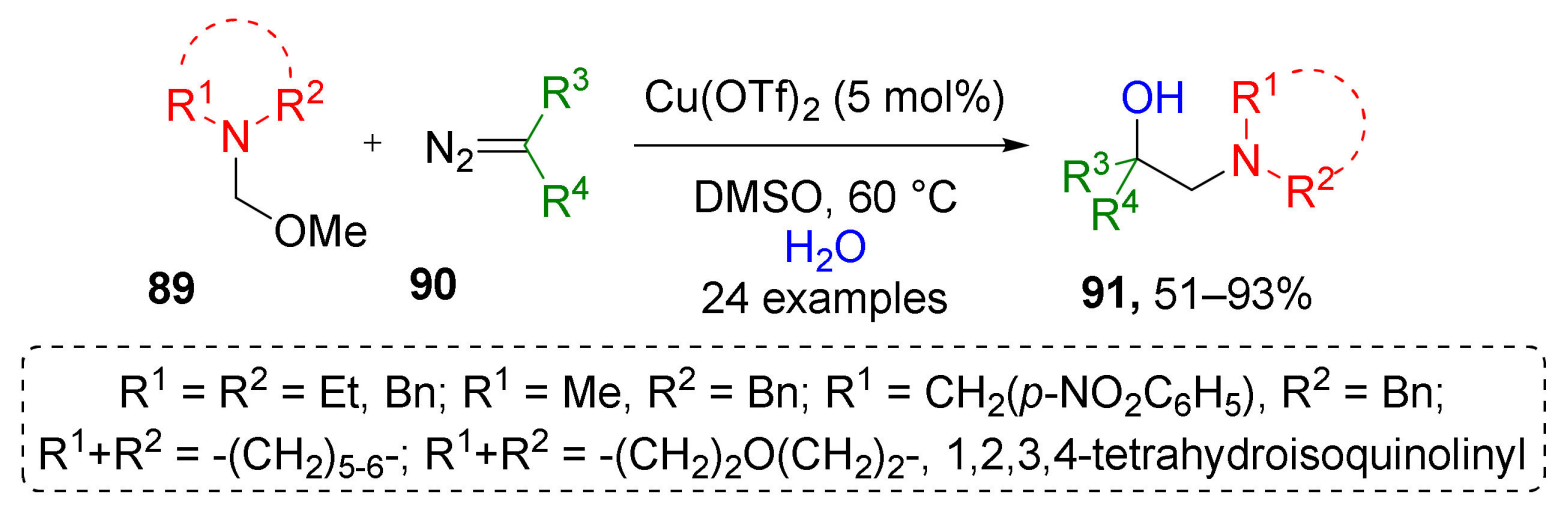
Copper-catalyzed oxy-aminomethylation of diazo compounds with *N*,*O*-acetals and water.

While in the above mentioned approach only one iminium precursor was used (*N*,*N*-dibenzyl), J. Sun et al. reported a copper-catalyzed protocol which expands it by variation of substituents at nitrogen atom in compounds **89** (Scheme 26) [36]. Specifically, different types of cyclic moieties, such as piperidine, morpholine, and dihydroisoquinoline were introduced to product molecules **91** in such a way. Notably, in this case, water was used instead of benzylic alcohol giving direct access to *O*-unprotected products. Additionally, a broader spectrum of diazo compounds **90**, namely, esters, lactones, amides, and ketones were found to be suitable for this reaction.

**Scheme 27:**
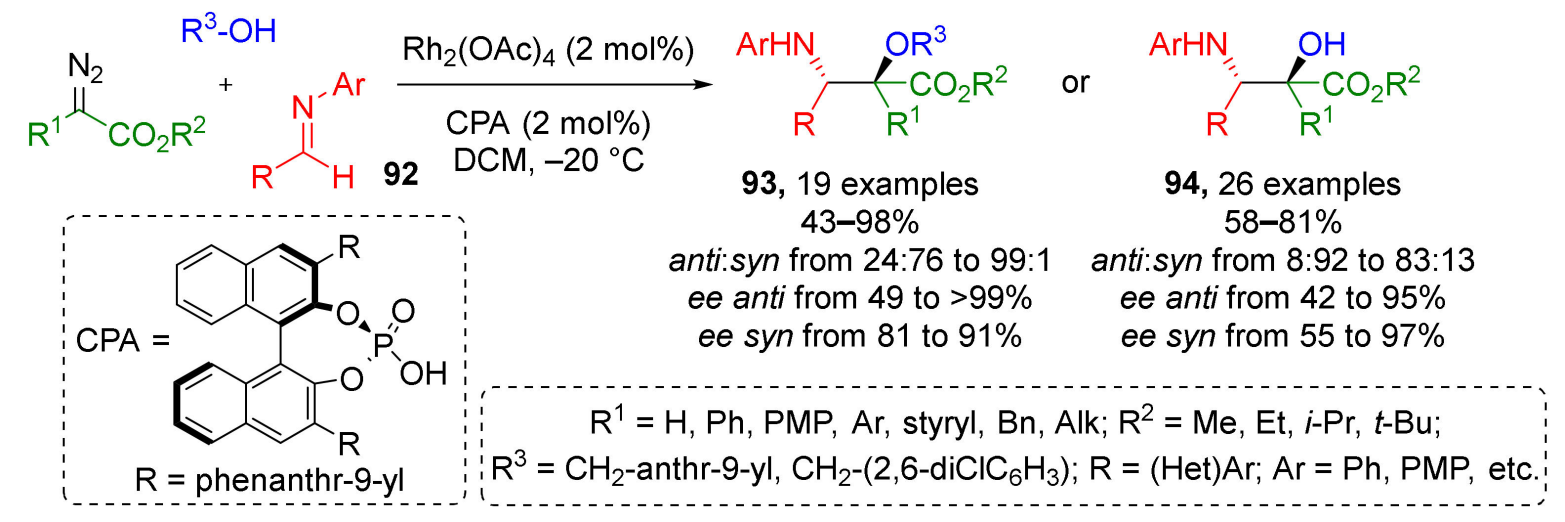
Catalytic enantioselective synthesis of norstatine derivatives.

Another modification of this strategy applied to classic imines **92** has already proven itself to be a useful instrument for medicinal chemistry—in 2019 a compound library consisting of 45 chiral α-hydroxy-β-aminoesters **93** and **94**, and norstatine derivatives, was prepared with excellent enantioselectivity and broad substrate scope in a straightforward fashion (Scheme 27) [37]. Two sets of reaction conditions were identified to direct the reaction selectively to formation of *syn*- or *anti*-diastereomer. Moreover, the authors developed a reaction work-up protocol for reusing of Rh-catalyst making this method part of sustainable chemistry.

**Scheme 28:**
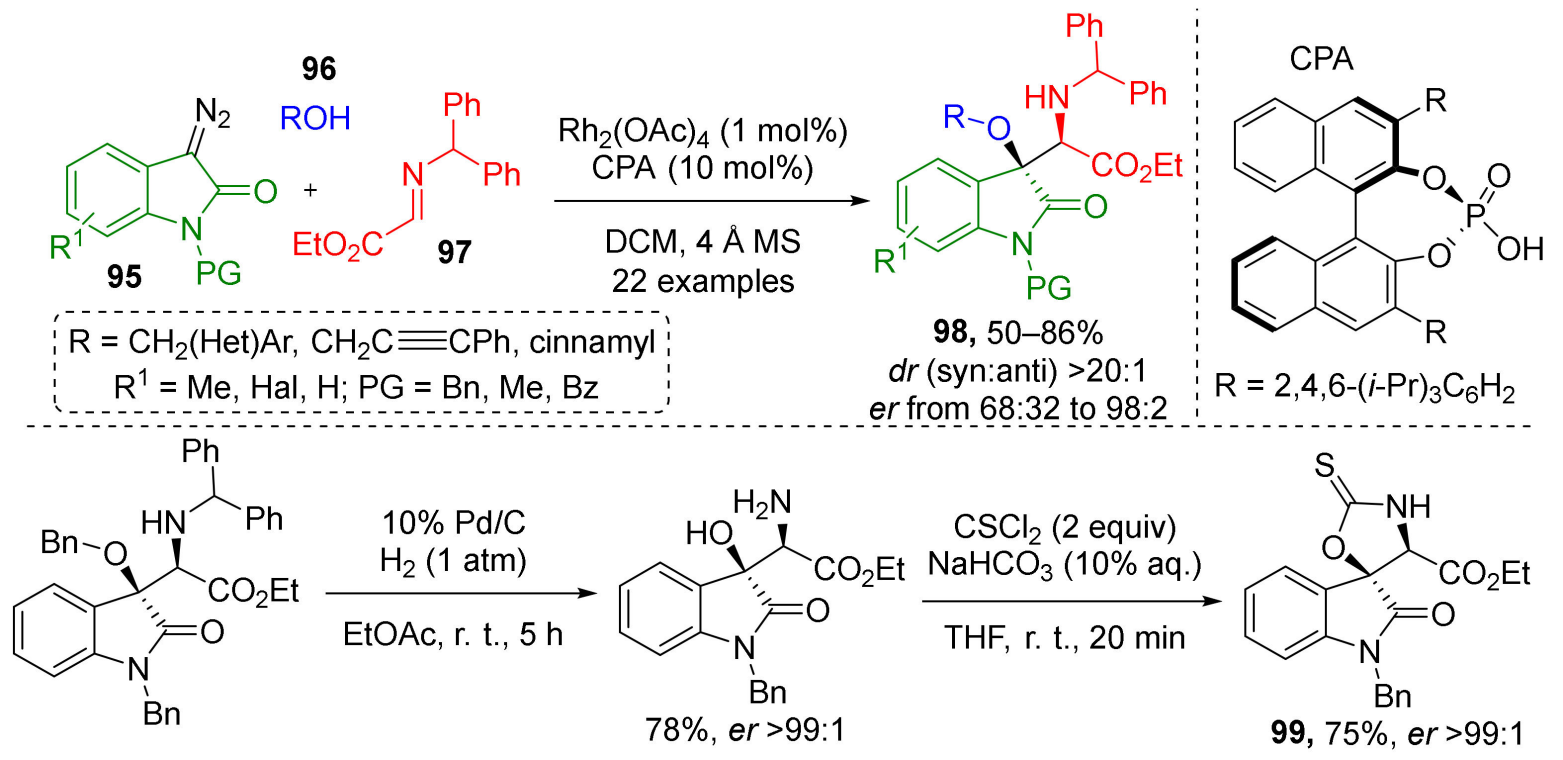
Enantioselective trapping of oxonium ylide intermediates by *N*-benzhydryl-α-imino ester.

Heterocyclic moieties can be also introduced to this type of MCR. One such example published in 2017 implemented *N*-substituted 3-diazoindoline-2-ones **95** (Scheme 28) [38] for the preparation of α-amino-β-hydroxy esters **98**. The other two reaction components were alcohols **96** and imines **97** prepared from ethyl glyoxylate. The scope of the alcohol component was limited to benzylic ones only. Catalytic system consisted of rhodium tetraacetate and the same type of chiral additive—BINOL-derived phosphoric acid (CPA). The authors also demonstrated the application of this methodology to synthesis of spiroheterocyclic compounds **99** via a two-step procedure.

**Scheme 29:**
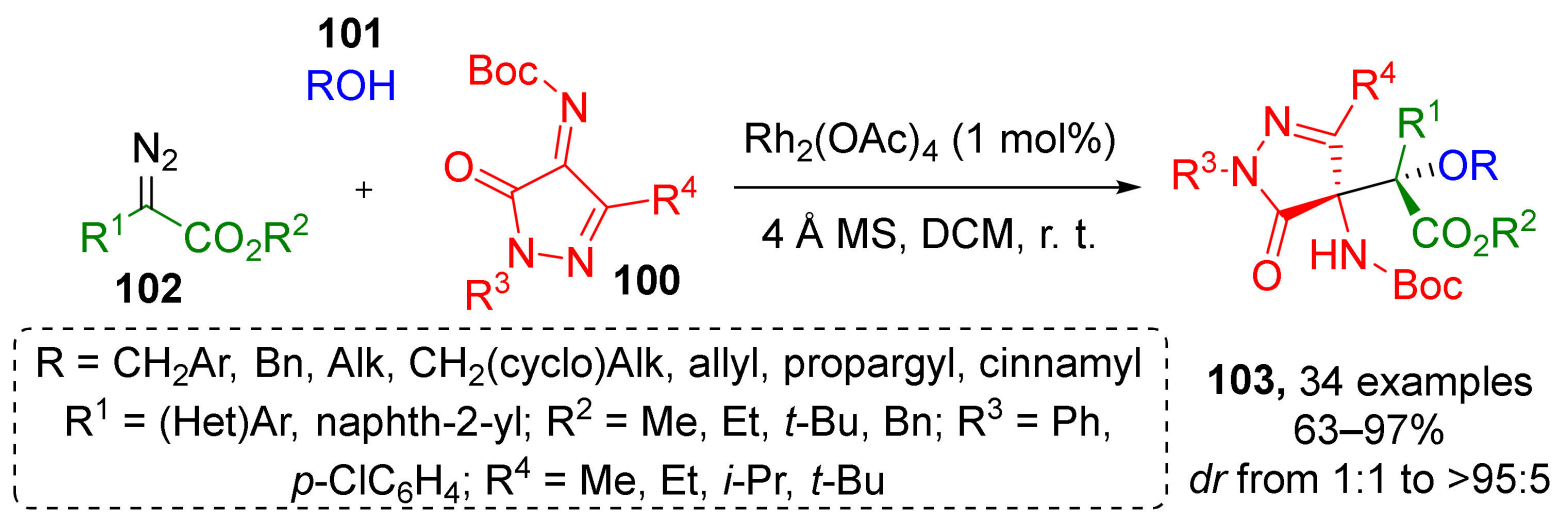
Synthesis of pyrazolone derivatives with contiguous quaternary stereocenters.

Imines can also become a source of heterocyclic substituents for α-hydroxy-β-aminoesters. Thus, in 2020, a series of pyrazolone-derived *N*-Boc-ketimines **100** was involved in Rh-catalyzed reaction with diazo esters **102** and alkyl alcohols **101** to provide compounds **103** (Scheme 29) [39]. The authors proposed regiospecific C-4 Mannich-type addition of an active oxonium ylide intermediate with ketimine as a plausible reaction mechanism. All products were isolated as single diastereomers.

**Scheme 30:**
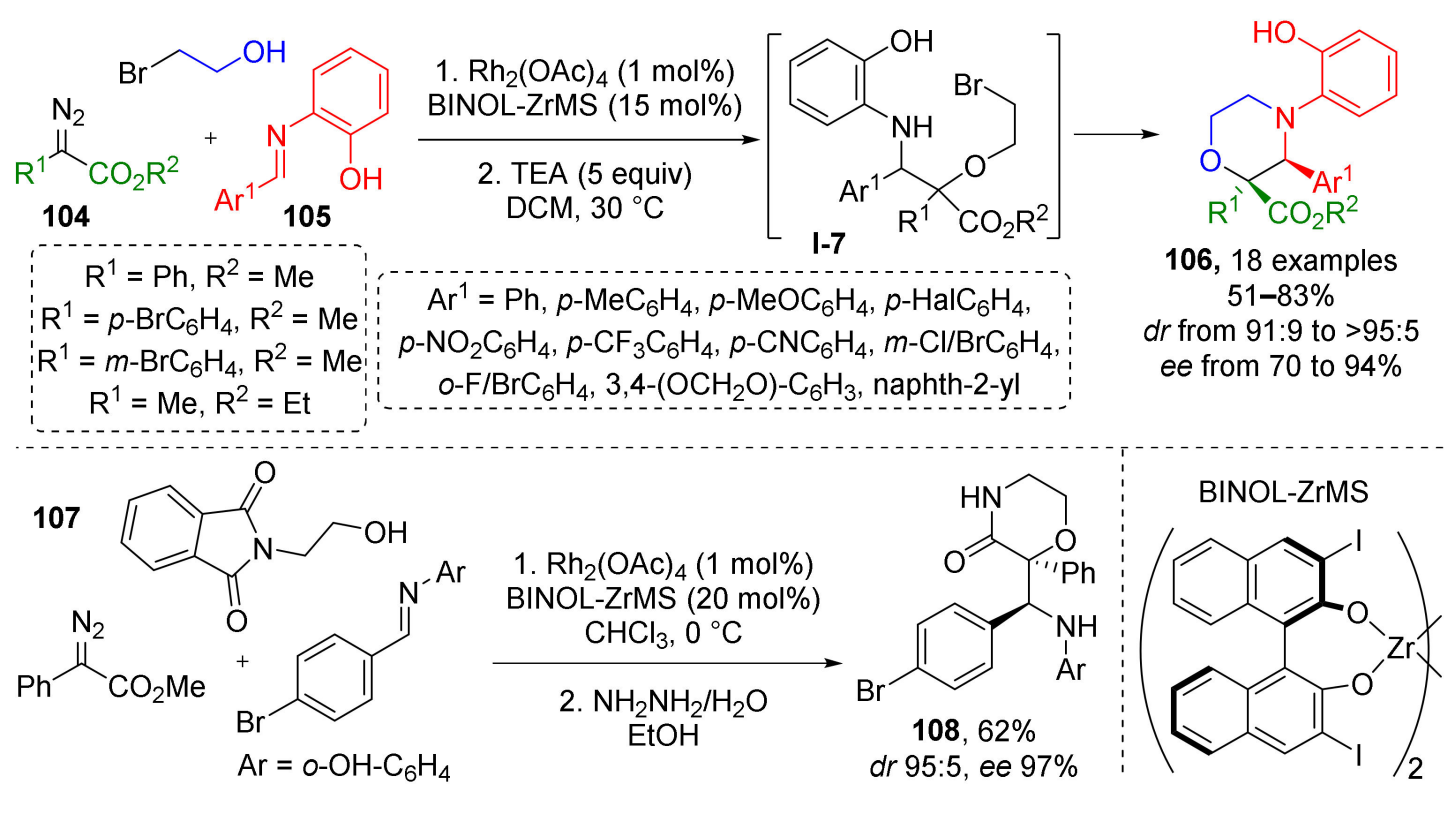
Three-component reactions of alcohols, diazo compounds, and aldimines with one-pot subsequent cyclization to morpholines.

This general methodology can also give access to saturated heterocyclic systems when alcohols with specific substituents are involved. For example, using 2-bromoethanol in Rh/Zn-catalyzed reaction with diazo esters **104** and imines **105** allowed preparation of 2,3-substituted morpholines **106** (Scheme 30) [40]. Initially formed acyclic α-hydroxy-β-amino ester **I-7** undergoes intramolecular *N*-alkylation reaction by bromoalkyl moiety upon addition of base (triethylamine) and mild heating. Another substrate, (*N*-phthalimido)-2-aminoethanol **107** served as amino group donor which was acylated by the ester group (coming from diazo compound) after deprotection with hydrazine hydrate giving substituted 2-disubstituted-morpholine-3-ones **108**.

**Scheme 31:**
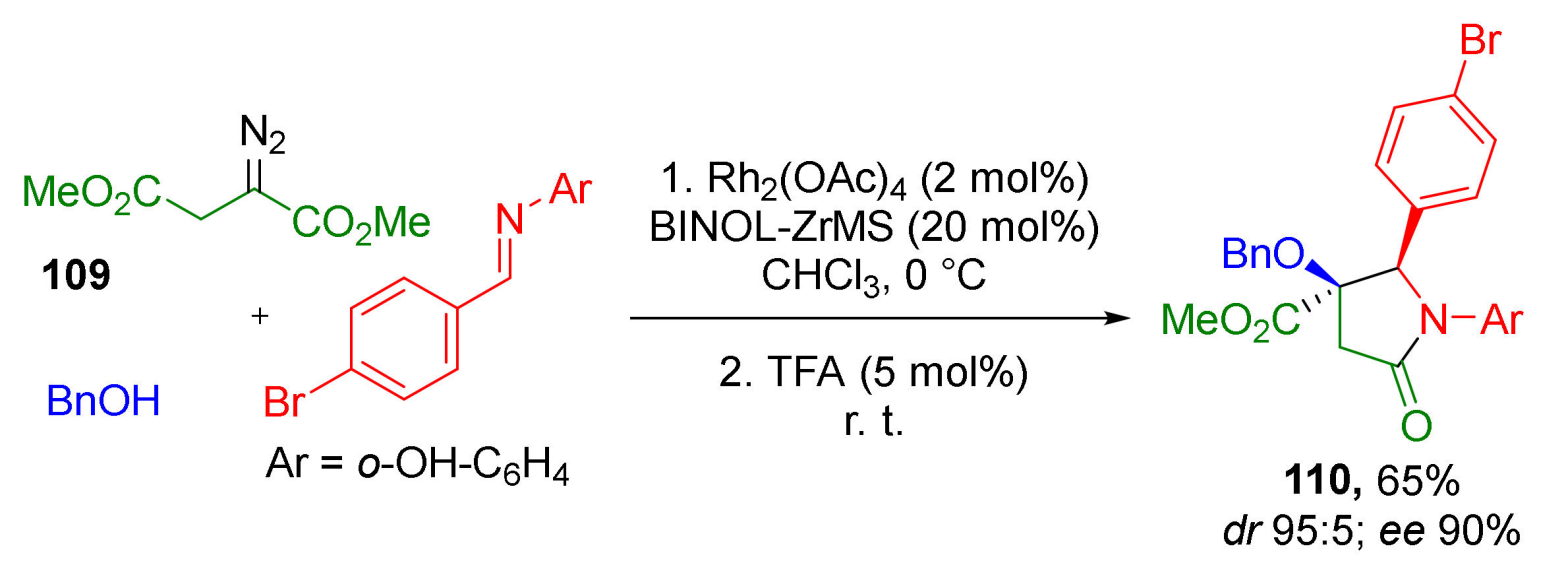
Extension of the methodology to the synthesis of γ-lactams.

The authors also showed that tetrasubstituted pyrrolidine-2-ones **110** can be constructed by the same type of cyclization—*N*-acylation with ester but using groups in other positions (Scheme 31). Thus, the imine nitrogen atom served as nucleophilic component, while the acylating ester group was located in β-position to diazo function (in contrast to α- in previous schemes) in dimethyl diazo succinate **109**. This step was catalyzed by trifluoroacetic acid. Interestingly, in all cases Zr/BINOL complex was used as a co-catalyst (instead of BINOL-derived phosphoric acid) along with rhodium tetraacetate to provide enantioselectivity during the formation of the α-hydroxy-β-aminoester.

**Scheme 32:**
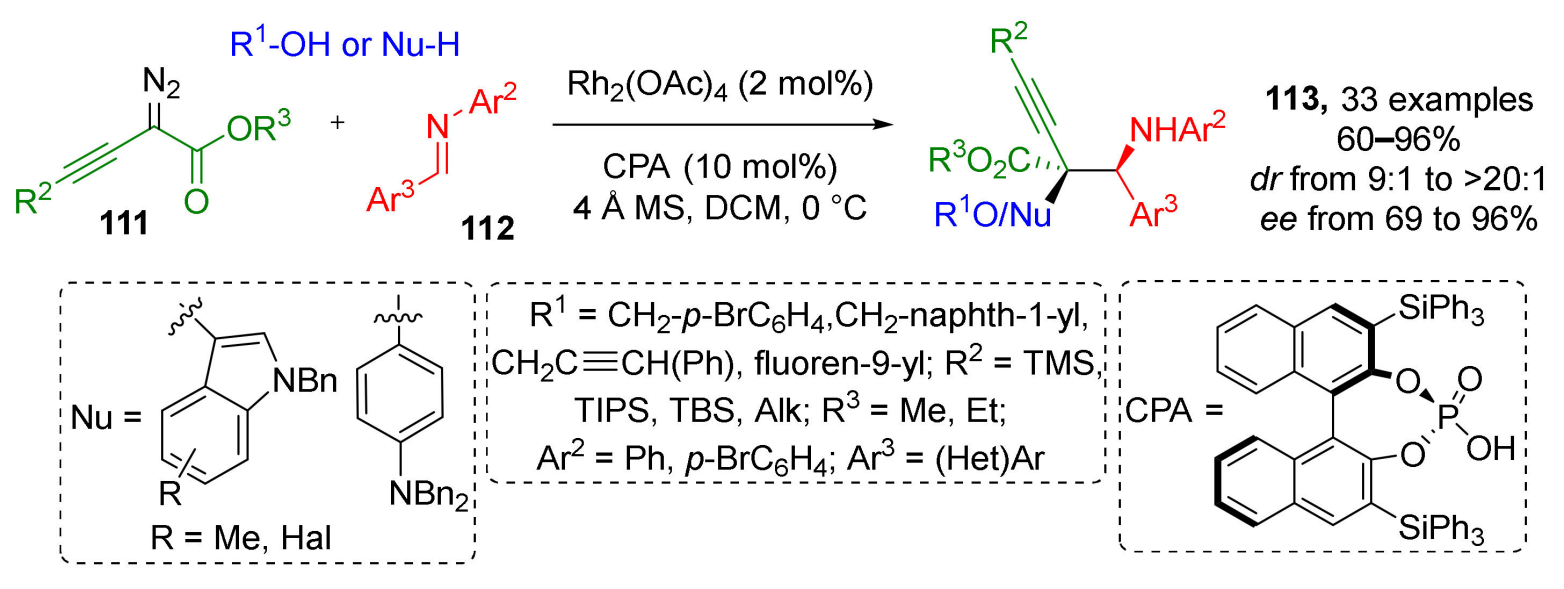
Multicomponent reaction for efficient construction of homopropargyl amine carboxylic esters.

A substantial expansion of diazo component scope for this type of MCR was published in 2019. The authors developed a Rh-catalyzed protocol giving access to α-hydroxy-α-alkynyl-β-aminoesters **113** via the reaction of α-diazo homopropargylates **111**, *N*-arylimines **112**, and alcohols (Scheme 32) [41]. Full stereoselectivity control was achieved by means of chiral phosphoric acid additive. The reaction tolerated a broad spectrum of alkyl and silyl substituents in acetylene moiety. The latter feature opens a new route for post modifications of initial products via deprotection and cycloaddition reactions, e.g., introduction of a triazole moiety via CuAAC. Surprisingly, not only alcohols can be used as the third, nucleophilic, reaction partner (NuH) under the same conditions—a series of indoles or *N*,*N*-disubstituted anilines were also involved in this reaction via CH-activation.

**Scheme 33:**
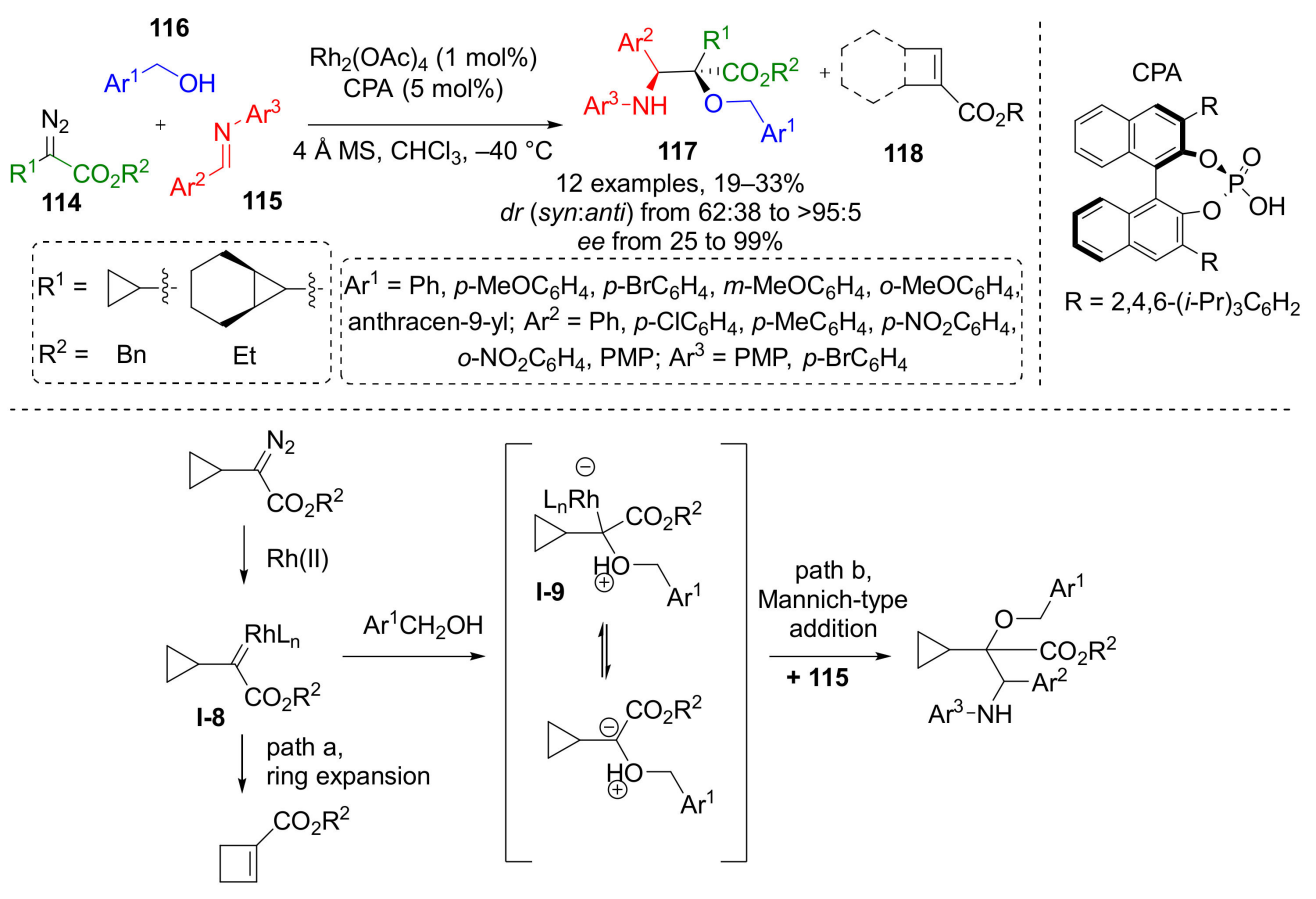
Ring expansion or trapping of cyclopropyl carbene generated from 2-cyclopropyl-2-diazoacetate via oxonium ylide with imine.

An interesting behavior of cyclopropyl substituted diazo esters was reported in 2016: a Rh-catalyzed reaction of the same set of substrates—diazo esters **114**, benzylic alcohol **115**, and imine **116** led to the predominant formation of carboxycyclobutanes **118** instead of α-hydroxy-α-cyclopropyl-β-aminoesters **117** (Scheme 33) [42]. The latter were isolated in 30% yield at maximum and moderate stereoselectivity. Cyclobutanes were formed via ring expansion reaction of Rh-carbenoid **I-8** generated from diazo compound. This intramolecular reaction predominated over the intermolecular formation of oxonium ylide **I-9** with alcohol. The reaction of this ylide with imine lead to formation of products **117** via Mannich-type addition.

**Scheme 34:**
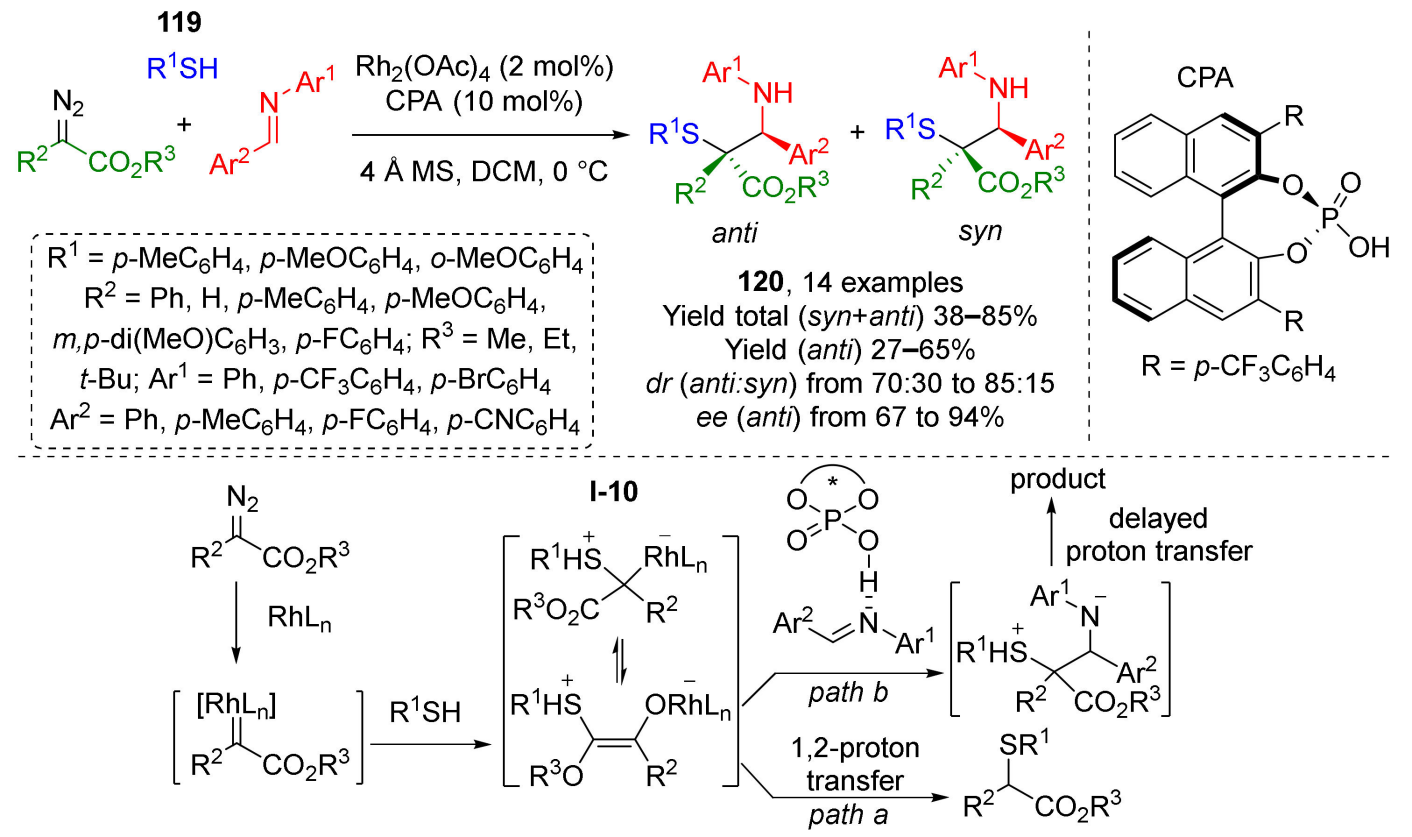
Synthesis of α-mercapto-β-amino esters via the three-component reaction involving aromatic thiols.

Aromatic thiols **119** can also be used as nucleophilic component for the above-mentioned synthetic strategy (Rh-catalyzed MCR of alcohols, diazo compounds, and imines) providing α-mercapto-β-amino esters **120** (Scheme 34) [43]. In this case, a sulfonium ylide **I-10** is generated as reaction intermediate. The product is formed after trapping of this ylide by chiral phosphoric acid-activated imine. Compared to analogous reaction with alcohols it can be noticed that thiols show an advantage of compatibility with aromatic substituents, while among alcohols, only benzylic ones are suitable. At the same time, reaction with thiols provided poorer diastereoselectivity.

**Scheme 35:**
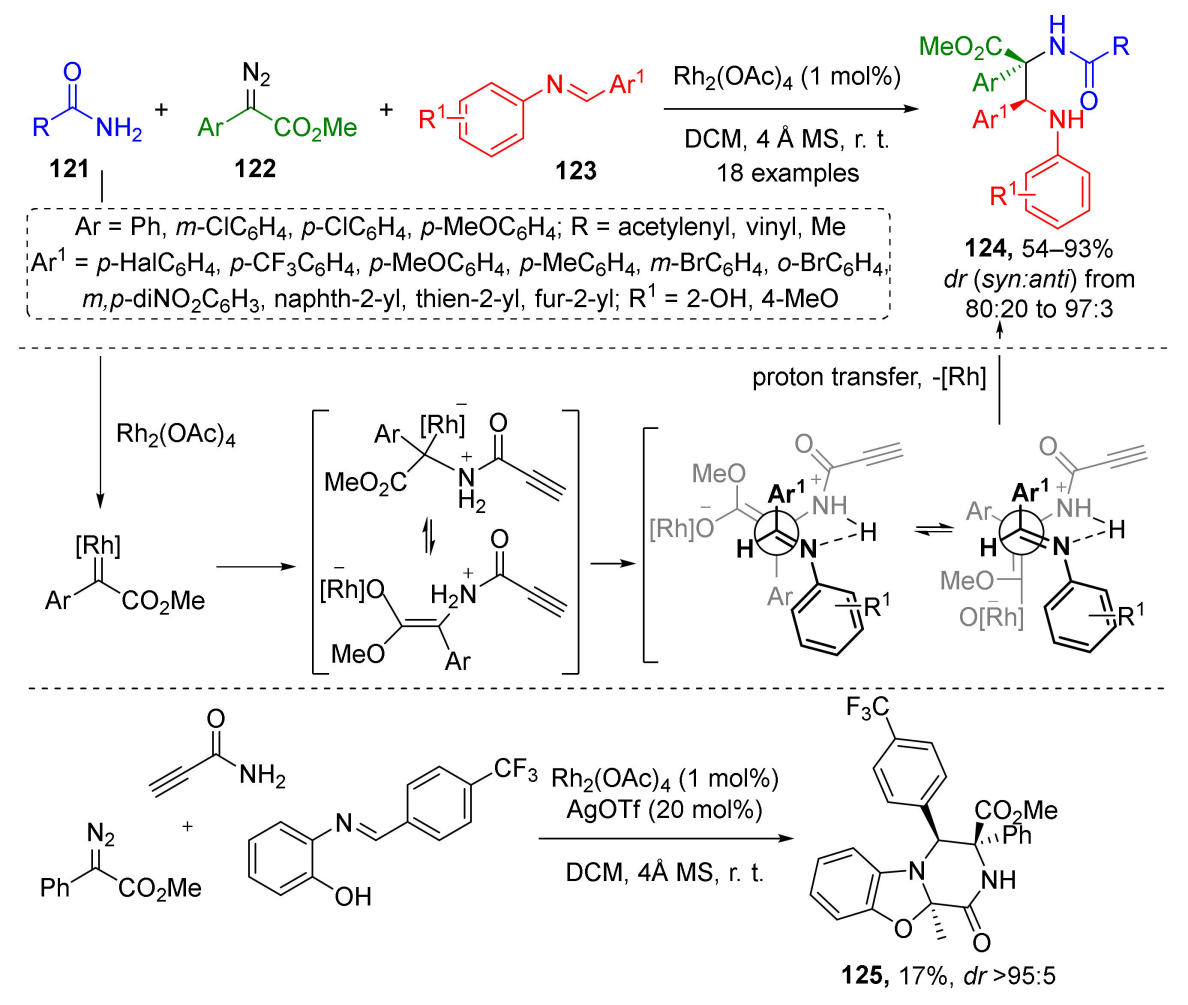
Multicomponent reaction for the construction of alkynylamide-substituted α,β-diamino acid derivatives.

An unusual approach to chiral α,β-diaminocarboxylic acids involving iminium substrates was demonstrated in 2017 by W. Hu et al. Amides **121** were used as *N*-nucleophile for Rh-catalyzed generation of ammonium ylide from diazo esters **122** (Scheme 35) [43]. *N*-Salicylaldimines **123** served as trapping agents giving alkynylamide-substituted α,β-diamino acid derivatives **124**. Noteworthy, the terminal acetylene moiety showed excellent stability under reaction conditions without any need for protection as in many other related reactions. Another surprising feature was inhibition by chiral phosphoric acids, typical co-catalysts for this type of MCR, therefore the reaction was conducted without it.

The obtained products were tested for anticancer activity (against HCT116, BEL7402, and SMMC772 cells) and 16 out of 18 compounds showed promising characteristics. Furthermore, 2-Hydroxy group in *N*-arylimine enabled another potential synthetic application of this strategy—one-pot cascade synthesis of tricyclic tetrahydro-2*H*-benzo[4,5]-oxazolo[3,2-*a*]pyrazines **125** under addition of AgOTf as co-catalyst to the same mixture of reactants.

**Scheme 36:**
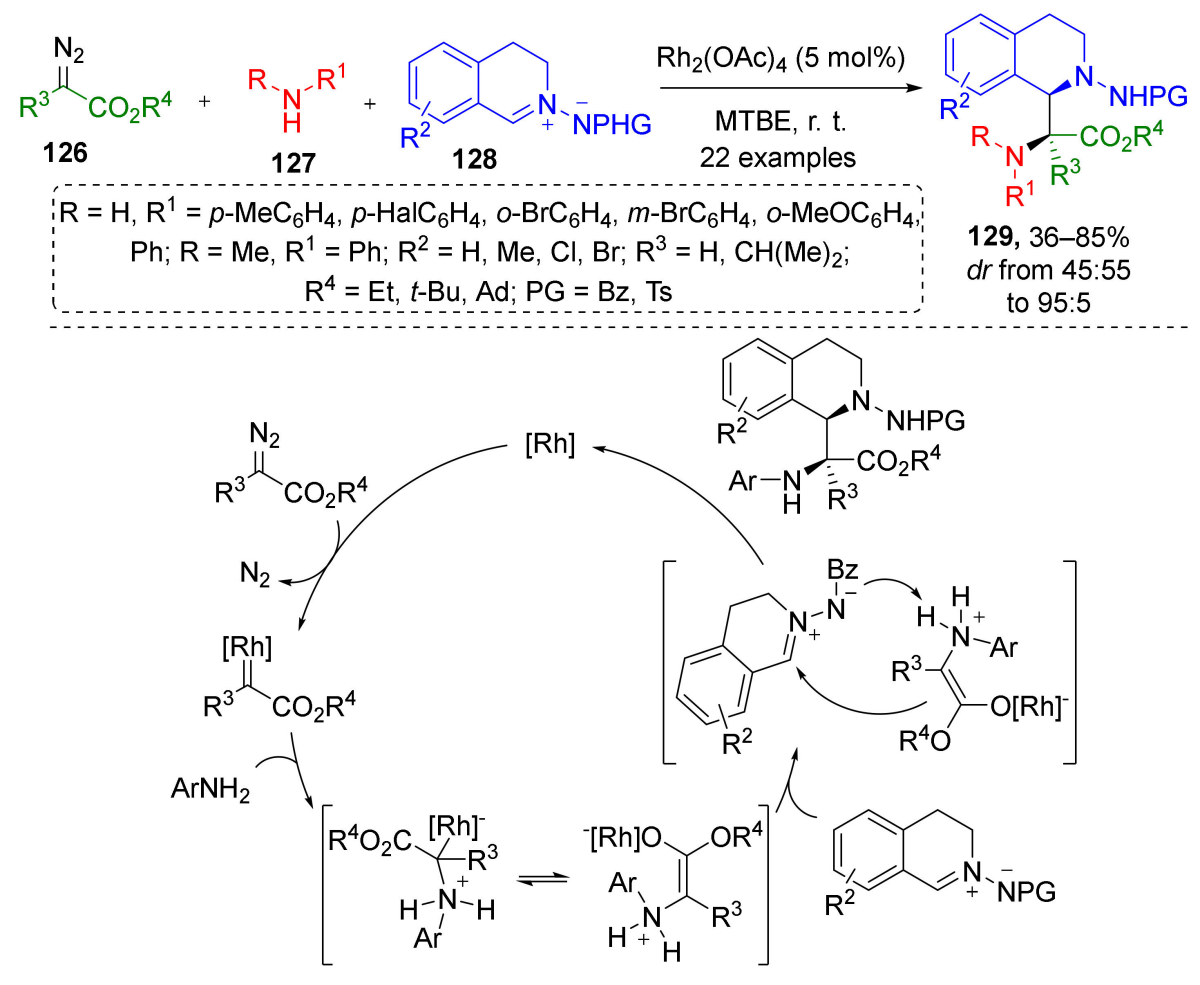
Three-component reaction to access C_1_-substituted tetrahydroisoquinolines.

In 2017, the same group published an extension of this methodology for the preparation of cyclic α,β-diaminocarboxylic acids. A diastereoselective three-component reaction of diazo esters **126**, anilines **127**, and isoquinolinic azomethine imines **128** delivered α-amino-α-(*N*-aminotetrahydroisoquinolin-1-yl) substituted carboxylic acids **129** under mild reaction conditions (Scheme 36) [44]. In this case, Rh-catalyzed generation of ammonium ylide from terminal diazo ester and aniline was followed by nucleophilic addition of ylide to iminium electrophilic carbon atom. This is a rare example of involving azomethine imines as imine component into such MCRs.

### 3.3. Generation of Ylides Followed by Conjugate Addition Reaction

During the last several years, a series of methods based on trapping ylides by α,β-unsaturated compounds has been developed. A-Alkenyl or alkynyl carbonyl compounds as well as nitroalkenes are known as the most common substrates for conjugate addition acting as Michael acceptors.

**Scheme 37:**
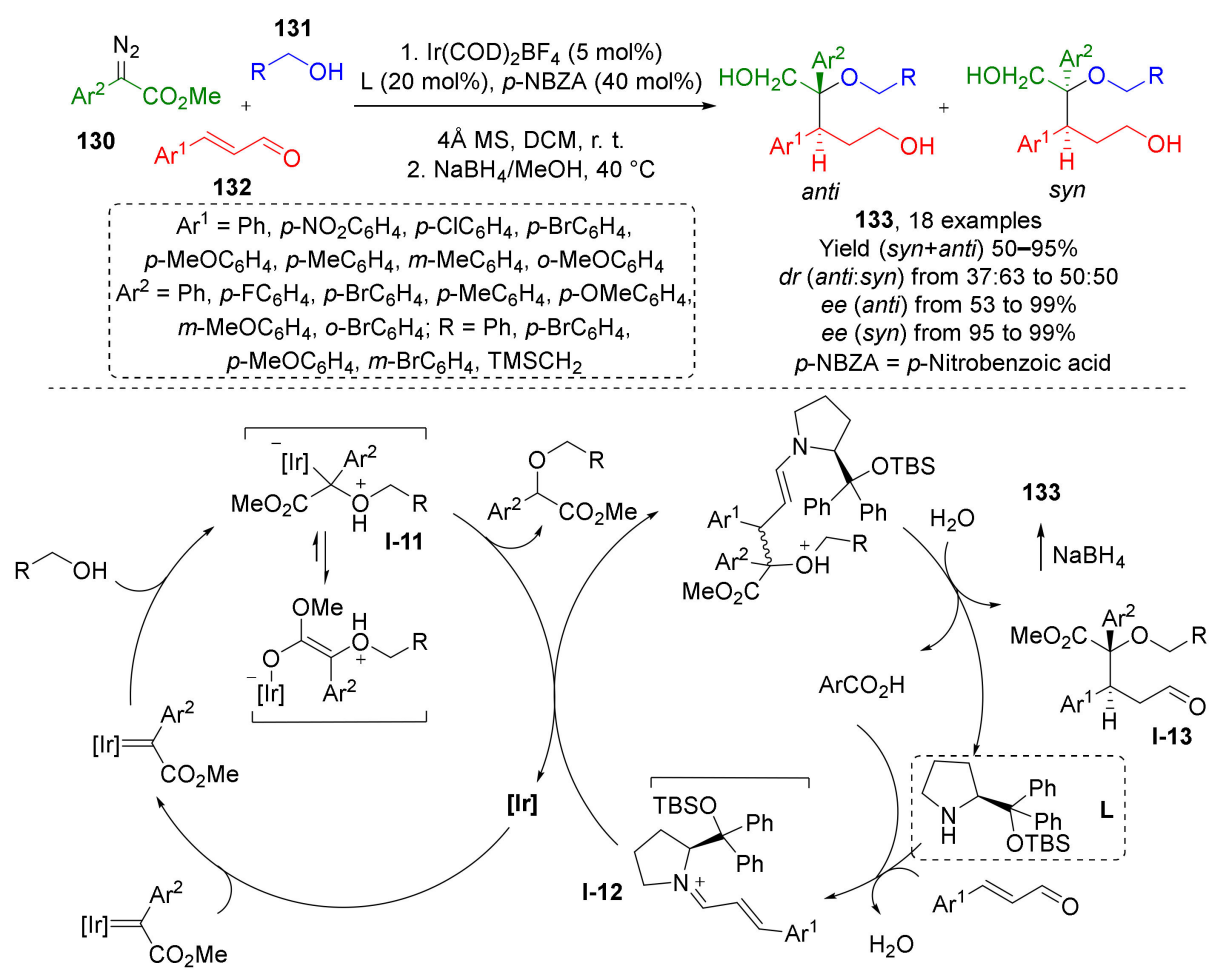
Multicomponent reaction for rapid construction of 1,2,5-triol derivatives.

In 2017, W. Hu et al. reported that an iridium-catalyzed MCR of diazo esters **130**, alcohols **131**, cinnamic aldehydes **132**, and substituted prolinol (L) affords stereoselective preparation 1,2,5-triol derivatives **133** with vicinal chiral centers, including one quaternary (Scheme 37) [45]. The oxonium ylide **I-12** was trapped via conjugate addition reaction with α,β-unsaturated iminium species **I-13** also generated in situ from aldehyde and prolinol derivative. The latter served as chiral auxiliary providing enantioselectivity. Noteworthy, the auxiliary was introduced and cleaved in one-pot fashion. To obtain triols from initial products **I-13** (which can be isolated) the carboxylic and formyl groups were reduced by sodium borohydride.

**Scheme 38:**
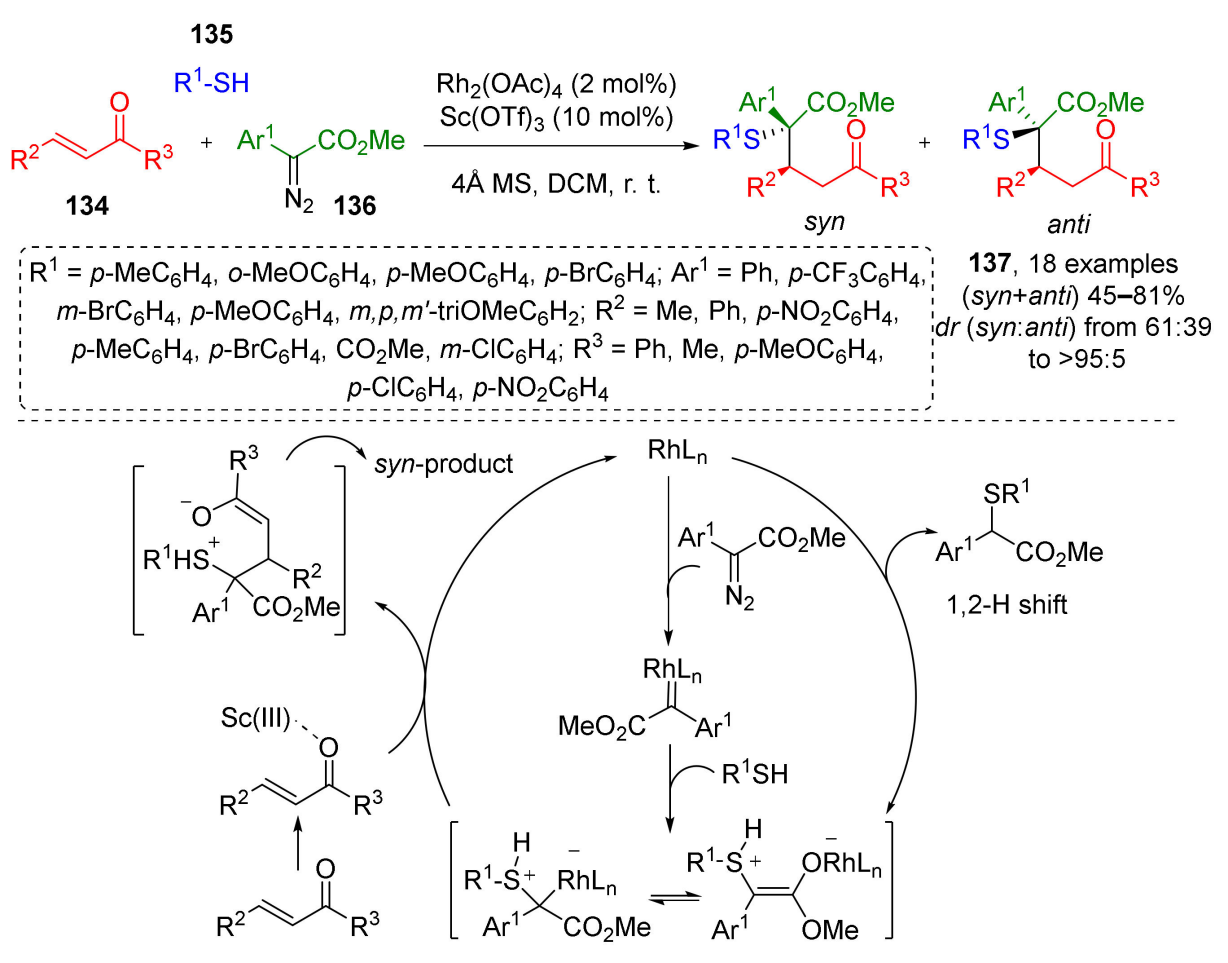
Synthesis of γ-sulfanyl-substituted ketones via Rh(II)/Sc(III)-catalyzed three-component reaction.

Closely related chalcones **134** were used as trapping agents for sulfonium ylide generated from diazo esters **136** and aromatic thiols **135** as a part of novel Rh/Sc-catalyzed MCR (Scheme 38) [46]. This methodology gave diastereoselective access to rare α-sulfanyl-γ-ketoesters **137**; while rhodium tetraacetate was used to transform diazo compound to carbenoid—an ylide precursor, scandium triflate, activated the carbonyl group of ketone facilitating conjugated addition step.

**Scheme 39:**
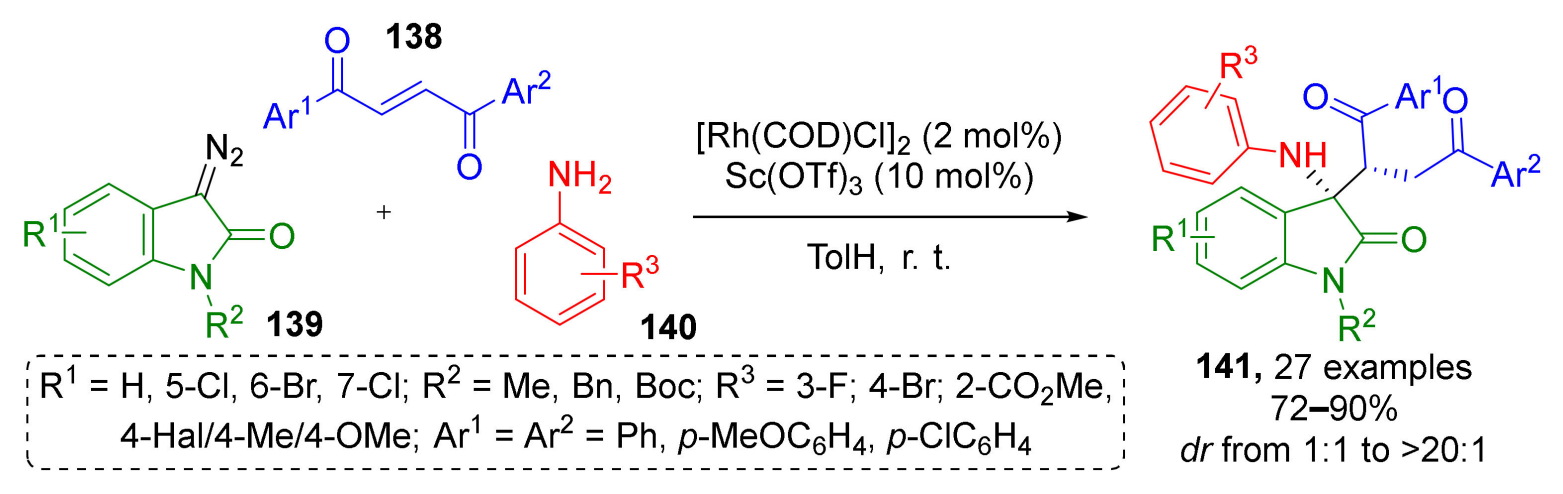
Rh(I)/Sc(OTf)_3_-catalyzed Michael addition of ammonium ylide to (*E*)-1,4-enediones.

In 2019, S. Liu et al. contributed to this approach by developing an MCR using 1,4-enediones **138** as Michael acceptors (Scheme 39) [47]. The reactive intermediate, namely, ammonium ylide was formed via Rh-catalyzed reaction of various *N*-substituted diazo oxindoles **139** and anilines **140**. In many cases the formation of target products **141** proceeded with poor diastereoselectivity. The authors also screened a series of chiral co-catalysts but only moderate ee values were obtained with no increase of diastereoselectivity.

**Scheme 40:**
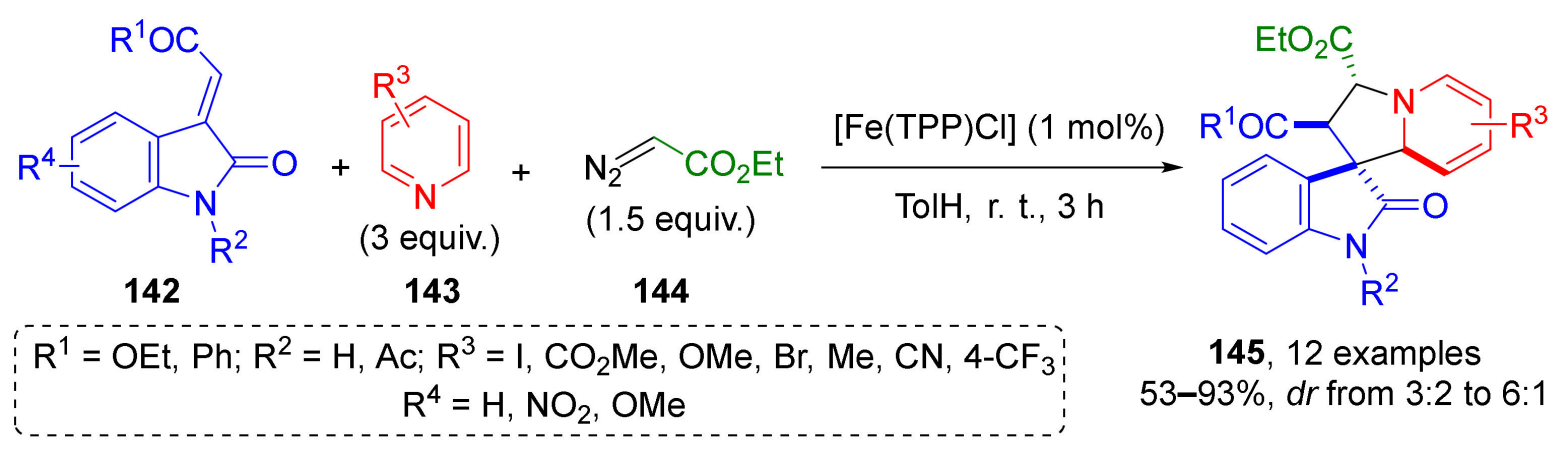
Synthesis of tetrahydroindolizines through the formation of pyridinium ylides.

The next important type of substrate for conjugate addition is carboxylic acid derivatives, such as esters, nitriles, and imides. J. Dowden et al. substantially increased the synthetic potential of unsaturated esters with oxindole periphery **142** by introducing them into a three-component synthesis of alkaloid-inspired tetrahydroindolizidine spirocyclic systems **145** containing four asymmetric atoms (Scheme 40) [48]. This MCR was also based on trapping of ammonium ylide, but of a rare type—where ylides were generated from pyridines **143** and diazocarbonyl compounds **144**. Noteworthy, the authors used non-classical catalyst—iron porphyrinate. The reaction was found to be general and compatible to different types of substitution pattern in pyridine component.

**Scheme 41:**
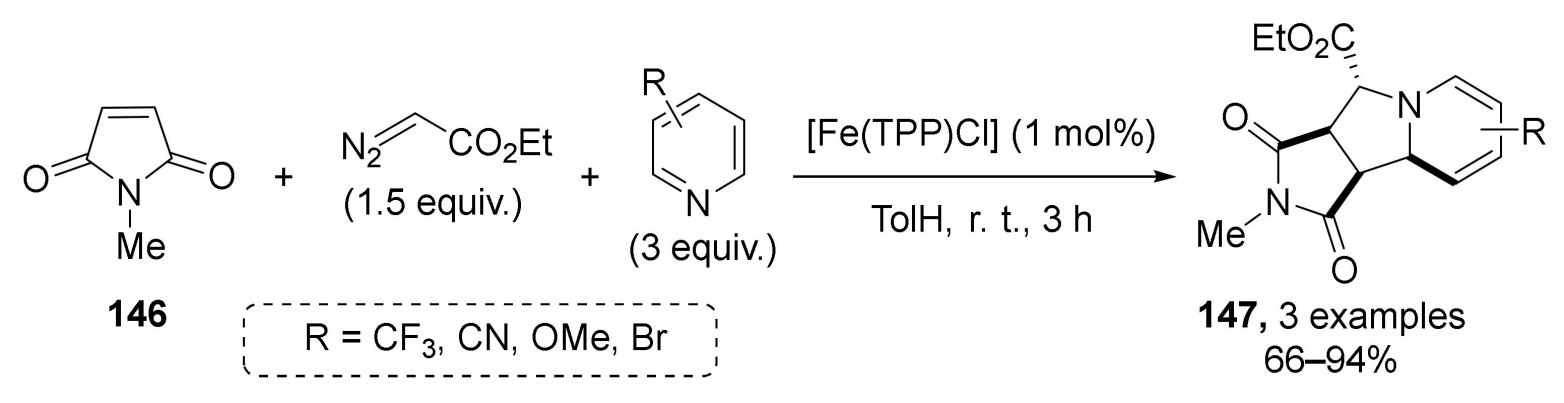
Synthesis of tetrahydroindolizidines from *N*-methylmaleimide.

Additionally, in the same work, the possibility of switching from esters to imide **146** in the role of Michael acceptor component was demonstrated for three examples (Scheme 41). This allowed the preparation of annulated tetrahydroindolizidines **147** in high yields under the same conditions.

**Scheme 42:**
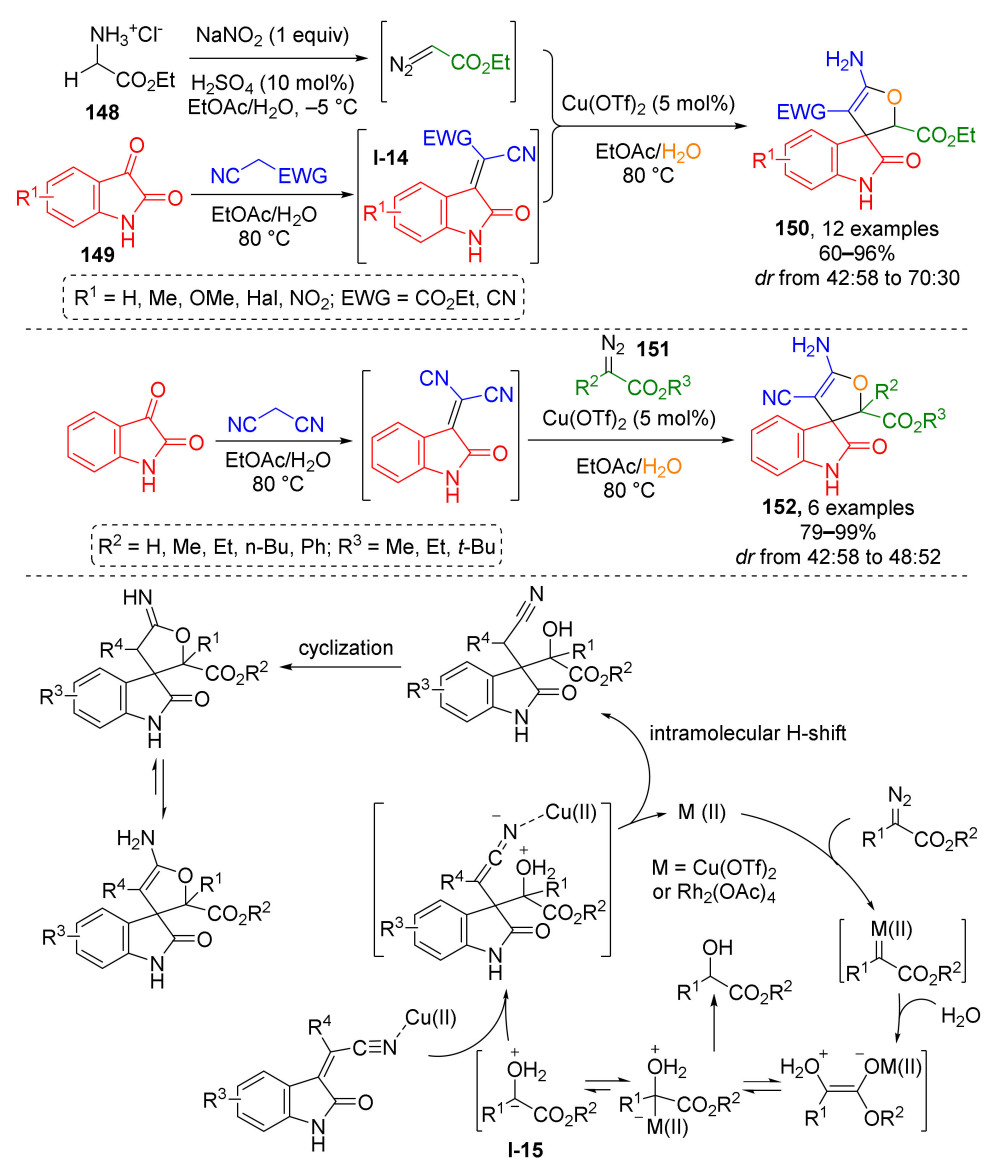
Synthesis of spiro[2,3-dihydrofuran-3,3′-oxindole] via a multi-component cascade reaction.

An approach to another class of spirocyclic compounds, namely, spiro[2,3-dihydrofuran-3,3′-oxindoles] **150**, has been developed on the basis of Cu-catalyzed complex cascade MCR involving glycine ester **148**, isatins **149**, and α-CH_2_-nitriles (malononitrile or cyanoacetate) and water as starting materials (Scheme 42) [49]. At first, the glycine ester was transformed into ethyl diazoacetate via addition of sodium nitrite under acidic conditions in a separate vessel. It was then introduced to a hot mixture of isatin and nitrile, which formed isatylidene nitriles **I-14** in situ via condensation reaction. Meanwhile the diazo compound reacted with water giving oxonium ylide **I-15** which was trapped via conjugate addition to isatylidene nitrile. Another three steps, namely, intramolecular H-shift, cyclization via nucleophilic addition of hydroxy group of the former ylide to cyano group, and imine-enamine tautomerization were required to furnish the final spirocyclic product. The use of environmentally benign solvents and the possibility for catalyst recycling made this methodology meet the requirements of green chemistry. This reaction was also applicable to other diazo esters **151**, which afforded compounds **152**.

**Scheme 43:**
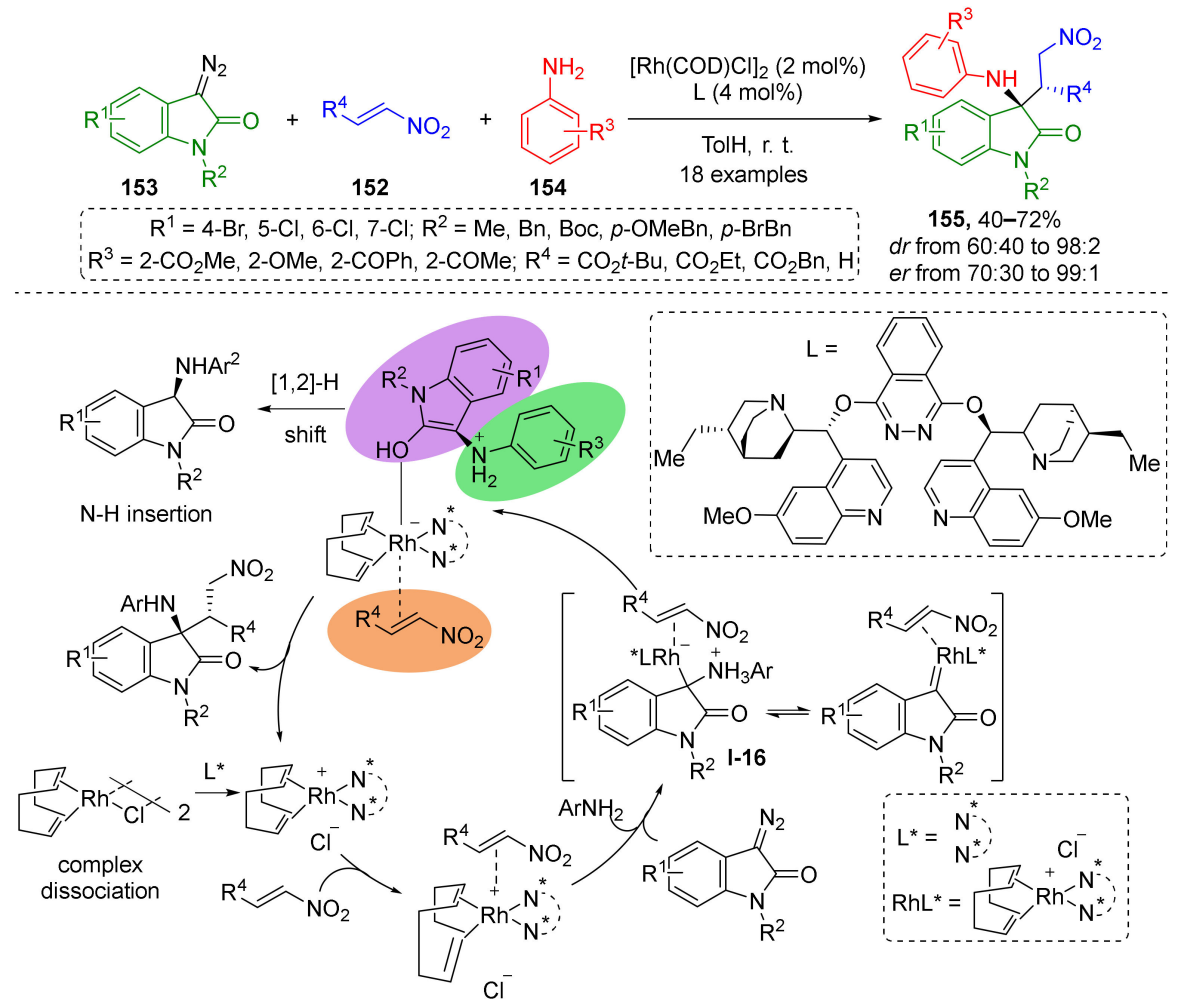
A triple-functionalized metal center-catalyzed enantioselective multicomponent reaction.

Nitroalkenes **152**, which are also regarded as classical substrates for conjugate addition, were successfully involved in the MCR with diazo indolinones **153** and anilines **154** under Rh-catalyzed conditions as was reported in 2018 by S. Liu (Scheme 43) [50]. The presence of quinuclidine/phthalazine chiral ligand (DHQ)_2_PHAL (= L) provided the desired products **155** in enantioselective fashion. In the first step, iminium ylide **I-16** was generated from diazo compound and aniline. This was followed by 1,4-nucleophilic addition to nitroethylene or nitroacrylates. A large series of diazo reagents with different substituents at the nitrogen atom and in the aryl ring was successfully applied. At the same time, the structural diversity of anilines was limited to *ortho*-substituted ones.

**Scheme 44:**
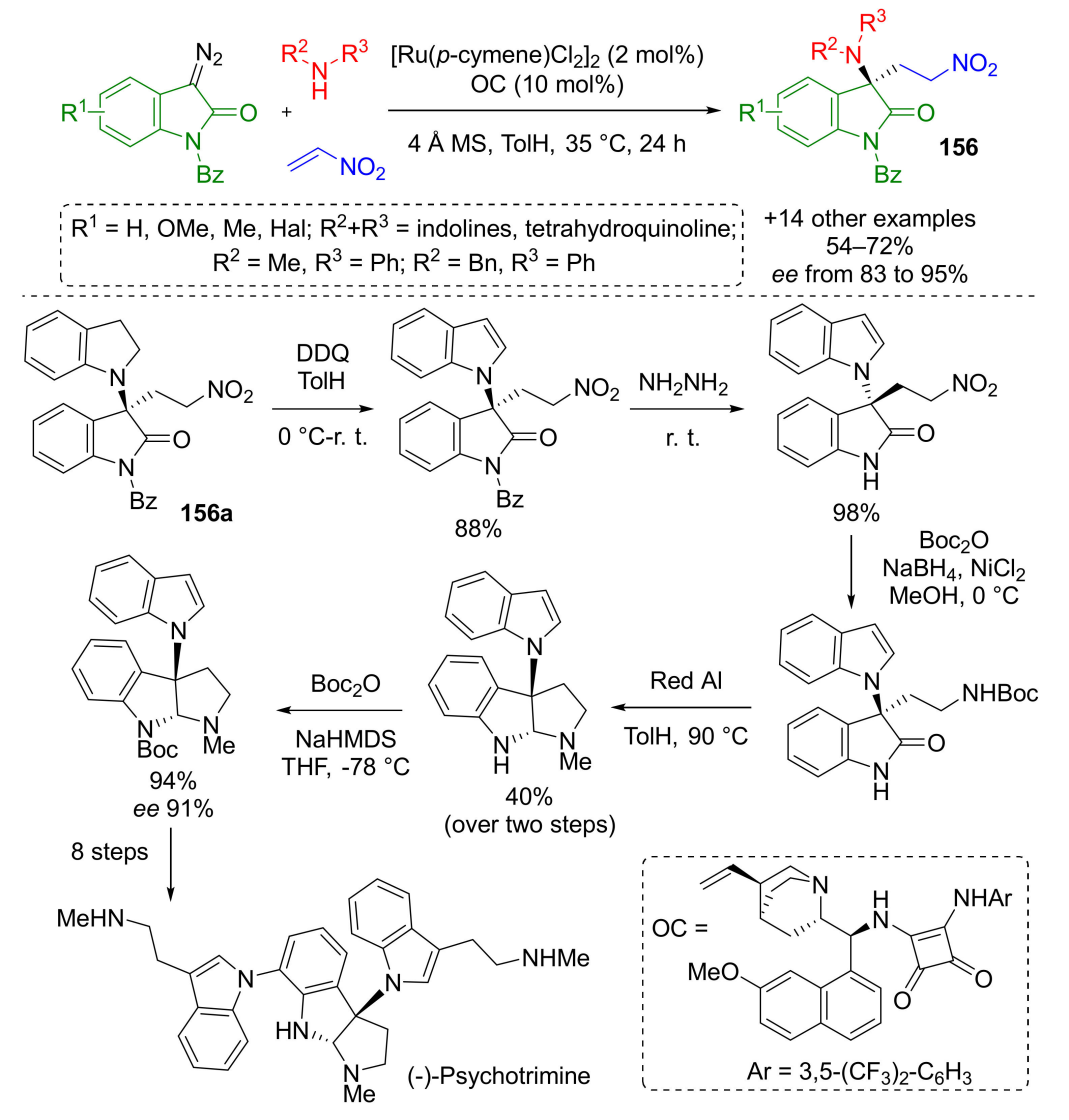
Ru(II)/organo relay catalytic reaction of 3-diazooxindoles, amines, and nitroalkene.

This methodology also found a practical application in the total synthesis of anticancer and antimicrobial alkaloid psychotrimine isolated in 2004 from Malaysian plant, *Psychotria rostrate* (Scheme 44) [51]. In this case, indolines served as specific amino component. Another difference from previous work was using ruthenium π-complex in combination with chiral quinine-based squaramide (OC) to perform reaction in asymmetric fashion. The authors performed a series of transformations with one of the products **156a** to prepare the key intermediate in total synthesis of psychotrimine developed by Takayama et al. [52].

**Scheme 45:**
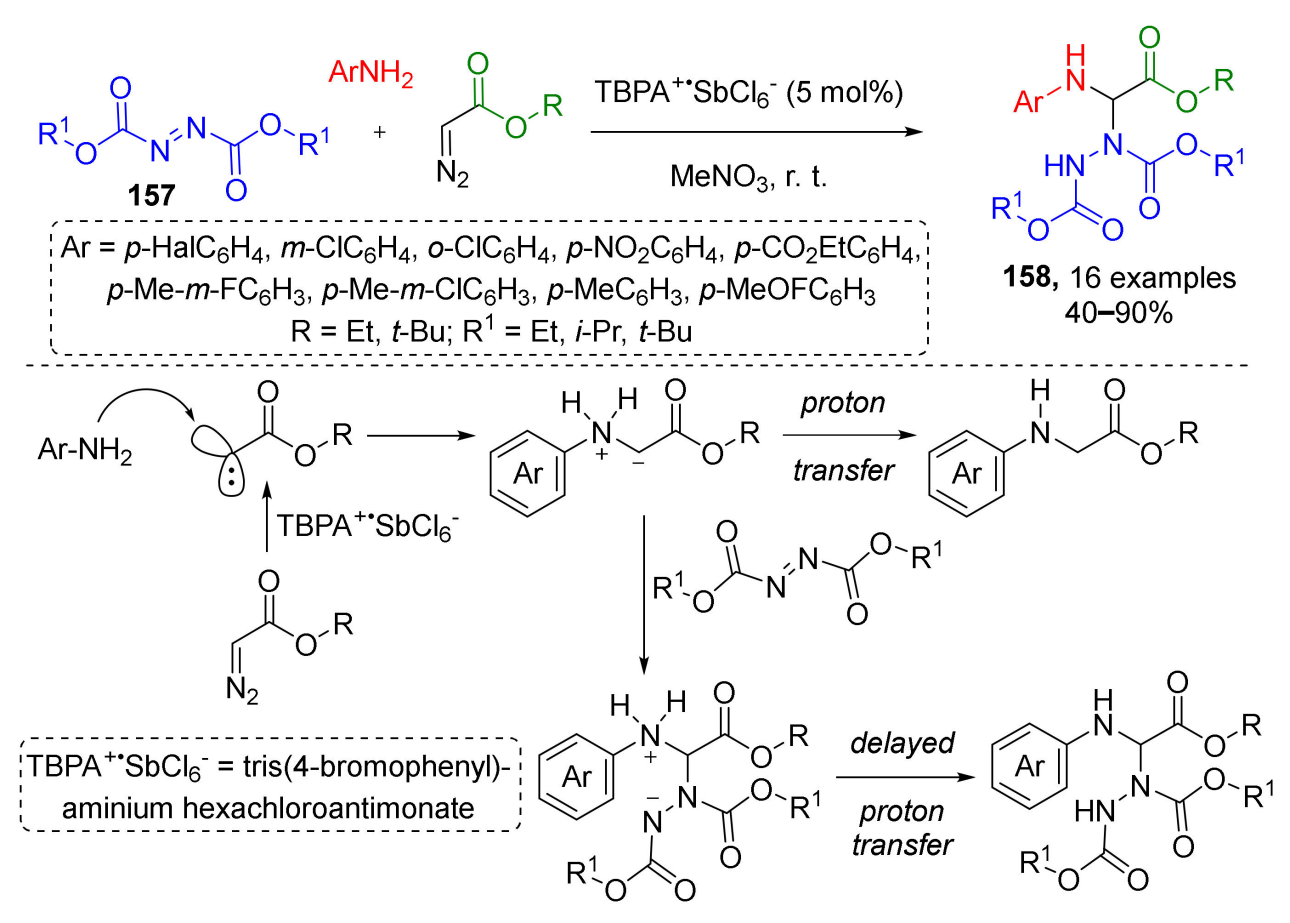
Stable radical-cation salt-initiated *N–H* insertion and related proton-transfer-delay three-component reaction.

In 2016, Y. Wand et al. reported that non-typical substrates—dialkyl azodicarboxylates **157**—can also be involved in this type of MCR and used for the preparation of complex aminals **158** (Scheme 45) [53]. Two other reaction partners were anilines and diazo acetates. One of the remarkable features of this protocol was using a stable cation-radical reagent, namely, tris(4-bromophenyl)ammonium hexachloroantimonate (TBPA^+^SbCl_6_^−^), for generating carbene from diazo compound, which further reacted with aniline to give ammonium ylide. The last step was the conjugate addition to the nitrogen atom in azodicarboxylate. This transition metal-free reaction was found to be rather general. It tolerated both electron rich and electron poor substituents in the anilines. The nature of substituent in the ester group did not show any significant influence on the reaction outcome—even substrates with bulky *t*-butyl groups provided products in good yields.

**Scheme 46:**
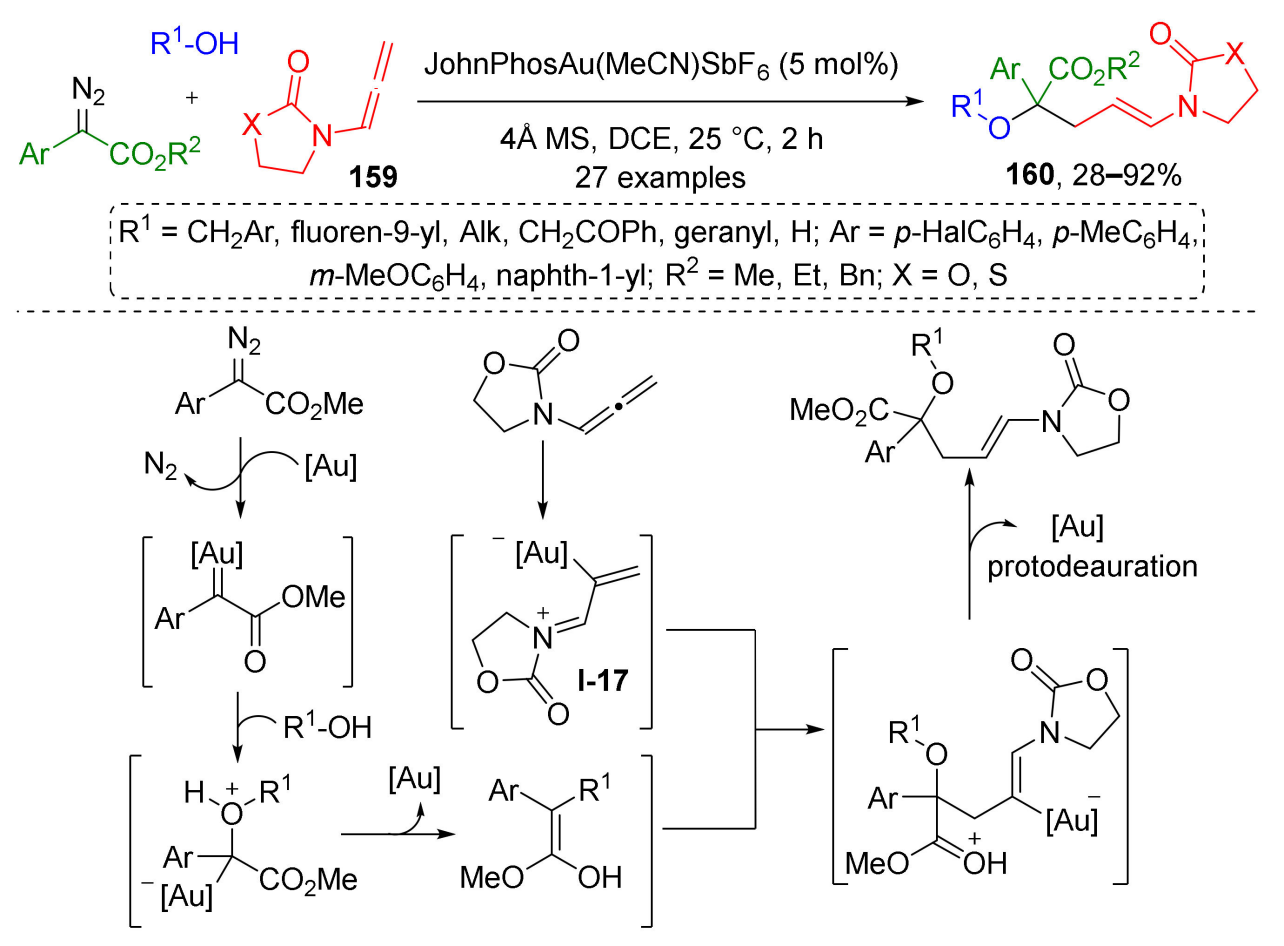
Au(I)-catalyzed chemoselective three-component reaction of alcohols, α-diazocarbonyl compounds, and allenamides.

Recently, this strategy (MCR based on trapping of ylide by conjugate addition) was substantially improved by H. Qui et al. in a non-trivial and elegant way by involving alleneamides **159** as trapping agents for oxonium ylide under Au(I)-catalysis (Scheme 46) [54]. Although alleneamides themselves cannot be regarded as conjugated systems and substrates for Michael addition, the situation changes drastically upon their coordination to gold ions by one of the carbon atoms, which causes the migration of double bonds and formation of positively charged conjugated 1-aza-1,3-diene system of intermediate **I-17**. It further reacts with oxonium ylide generated in situ via gold-promoted reaction of benzylic alcohols and diazo esters. The last step of this sequence is protodeauration, which gives α-hydroxyesters **160** with *N*-(prop-1-en-1-yl)oxa/thiazolidin-2-one moiety and chiral quaternary carbon atom.

**Scheme 47:**
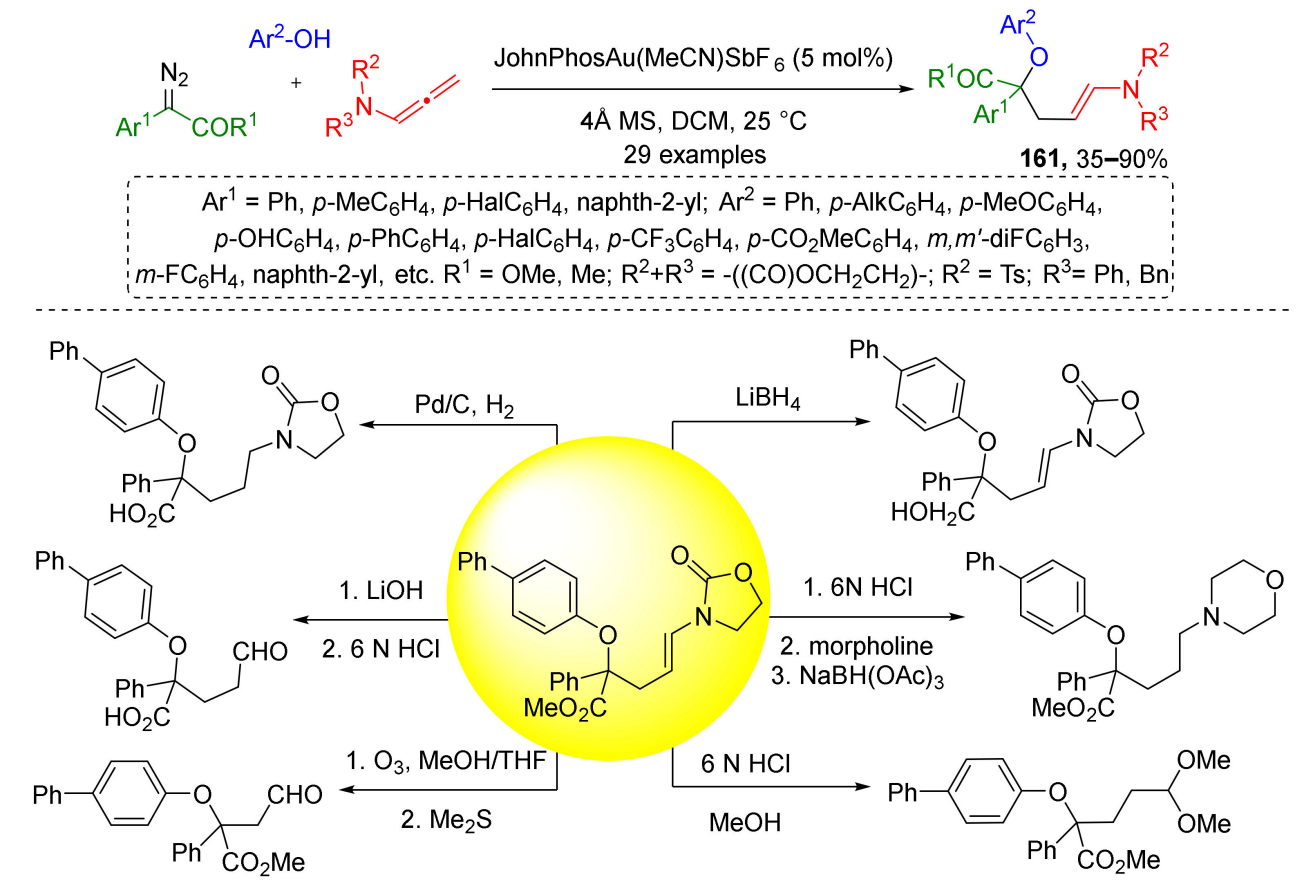
Chemoselective three-component reaction of phenols, α-diazocarbonyl compounds, and allenamides. Synthetic applications of the obtained products.

The same group in 2020 extended this methodology to using phenols as alcohol component (Scheme 47) [55]. Moreover, a series of post-modifications of obtained *N*-alkenyl oxazolidinone products **161** including reduction of olefin moiety, reduction of ester group, and selective cleavage of oxazolidinone to the formyl group was performed to demonstrate their synthetic applications. It should be mentioned that in both works the reactions were conducted in non-asymmetric conditions, therefore, products were obtained as racemates.

### 3.4. Generation of Ylides Followed by [3+2]-Cycloaddition Reactions

Furthermore, 1,3-Dipolar cycloaddition is a powerful tool for construction of various five-membered heterocycles. One of the most studied and frequently employed types of dipoles are ylides. Typically, they are generated in situ, which offers rich opportunities for developing novel types of MCRs including those involving the use of diazo compounds.

**Scheme 48:**
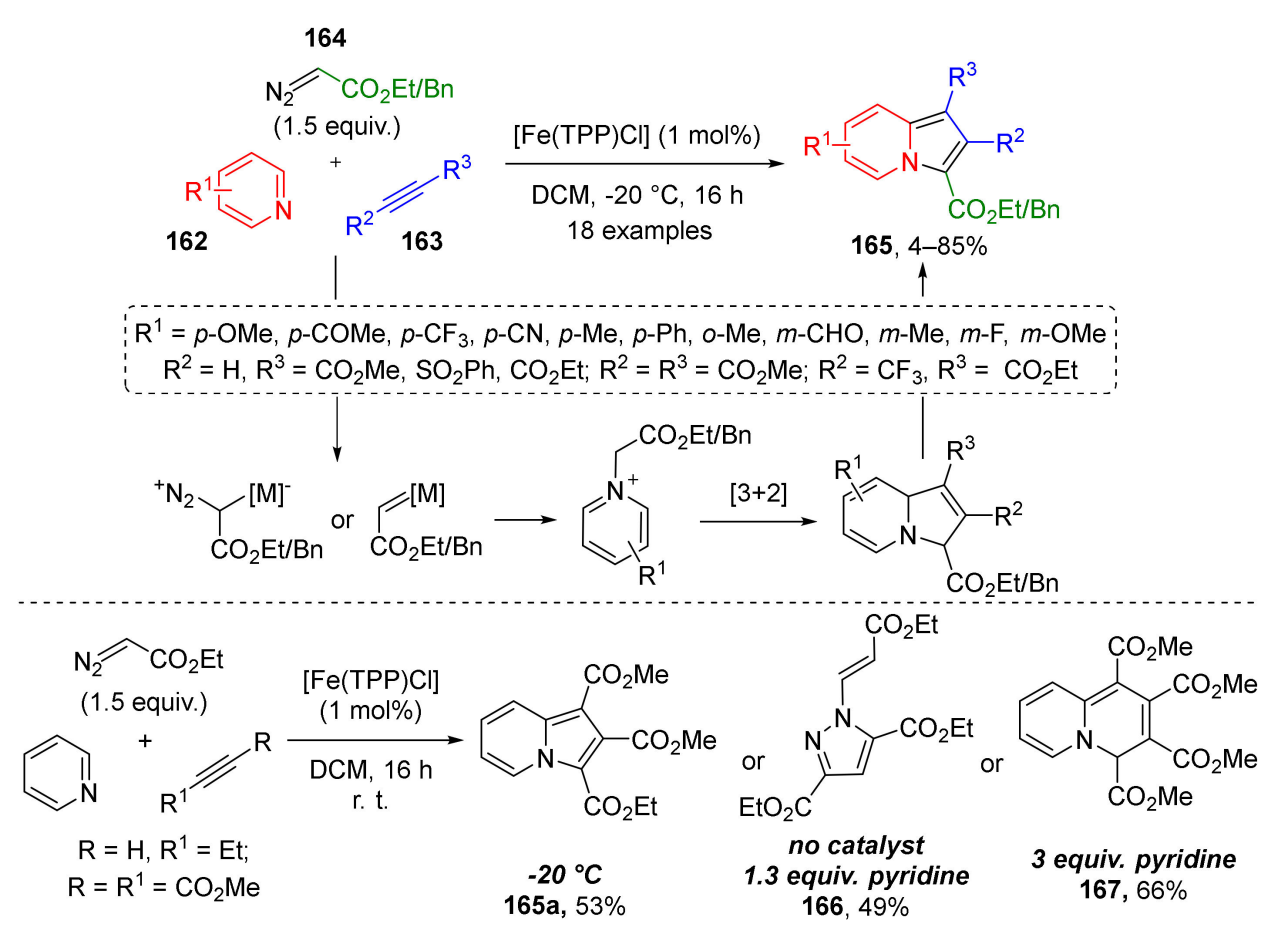
Iron-catalyzed indolizine synthesis from pyridines, diazo compounds, and alkynes.

Pyridinium and quinolinium ylides are well known as versatile building blocks in the construction of indolizines, including partially saturated, annulated, and spirocyclic ones with varying substitution patterns. Recently, a novel approach to indolizines **165** fully substituted at five-membered ring was reported. In 2017, J. Dowden and co-workers developed an iron-catalyzed protocol for the three-component reaction of pyridines **162**, acetylenes **163**, and terminal diazo esters **164** (Scheme 48) [56]. The aromatization of initial cycloaddition products—dihydroindolizines proceeded spontaneously upon standing in the air. The authors not only performed the synthesis of a large series of structurally diverse products (including rare formyl, sulfonyl, trifluoromethyl substituted), but also investigated the influence of reaction conditions on the course of the reaction. Remarkably, depending on the presence of the catalyst, base, and reaction temperature (20 or −20 °C), three different types of heterocycles, namely, indolizine **165a**, pyrazole **166**, and 4*H*-quinolizine **167** were isolated from the same set of substrates.

**Scheme 49:**
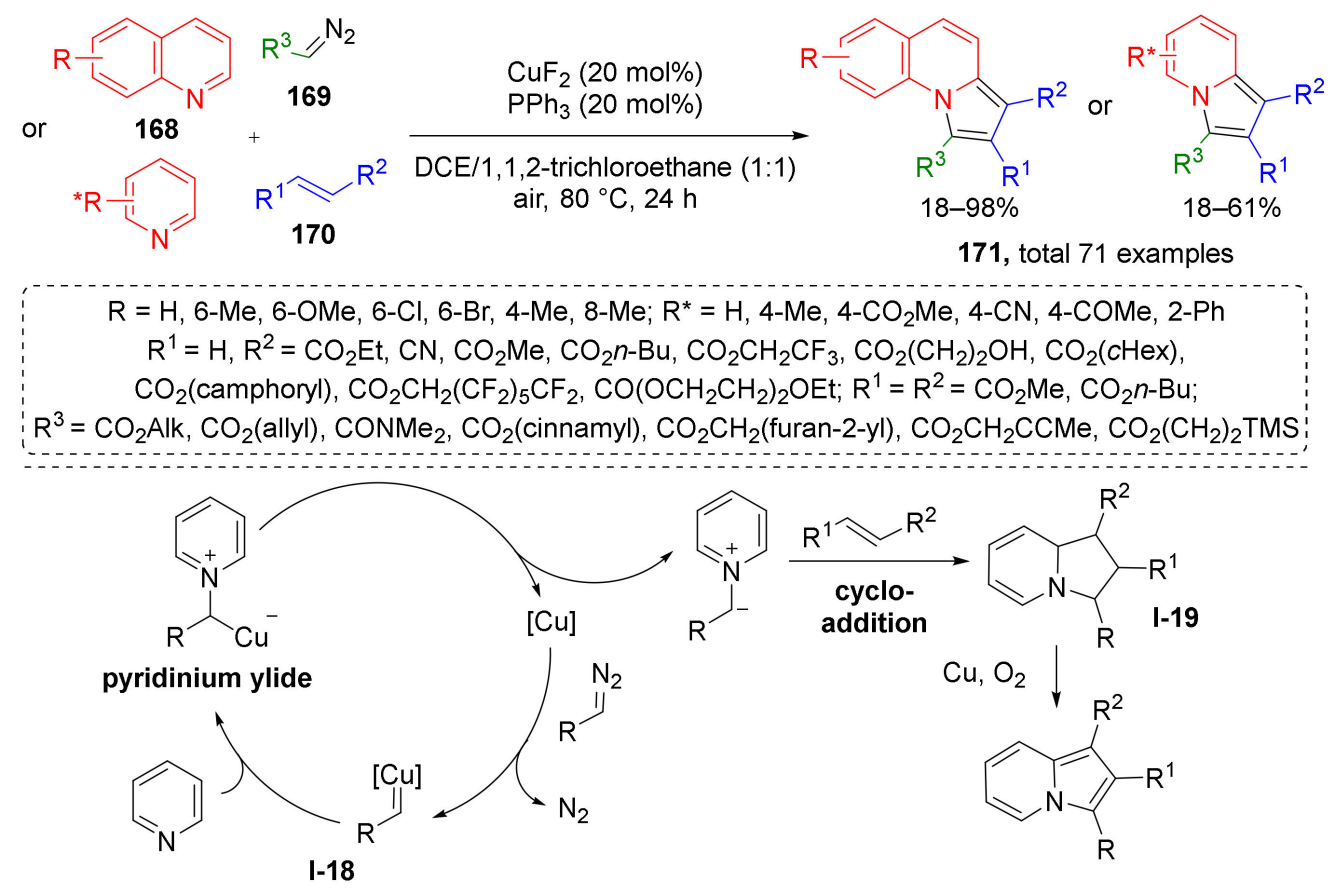
Copper-catalyzed synthesis of indolizines.

In the same year, X. Wan et al. demonstrated that annulated indolizines **171** can be efficiently prepared from quinolones **168**, diazo esters **169**, and alkenes **170** under CuF_2_ catalysis and oxygen atmosphere (Scheme 49) [57]. In contrast to previous methodologies, the reaction was conducted at elevated temperature (80 °C), which is typical for less active dipolarophiles—alkenes. Another difference is that formation of ylide proceeded from copper carbenoid **I-18** and the initial products of cycloaddition were 1,2,3,8a-tetrahydroindolizines **I-19**, which were further oxidized by O_2_.

**Scheme 50:**
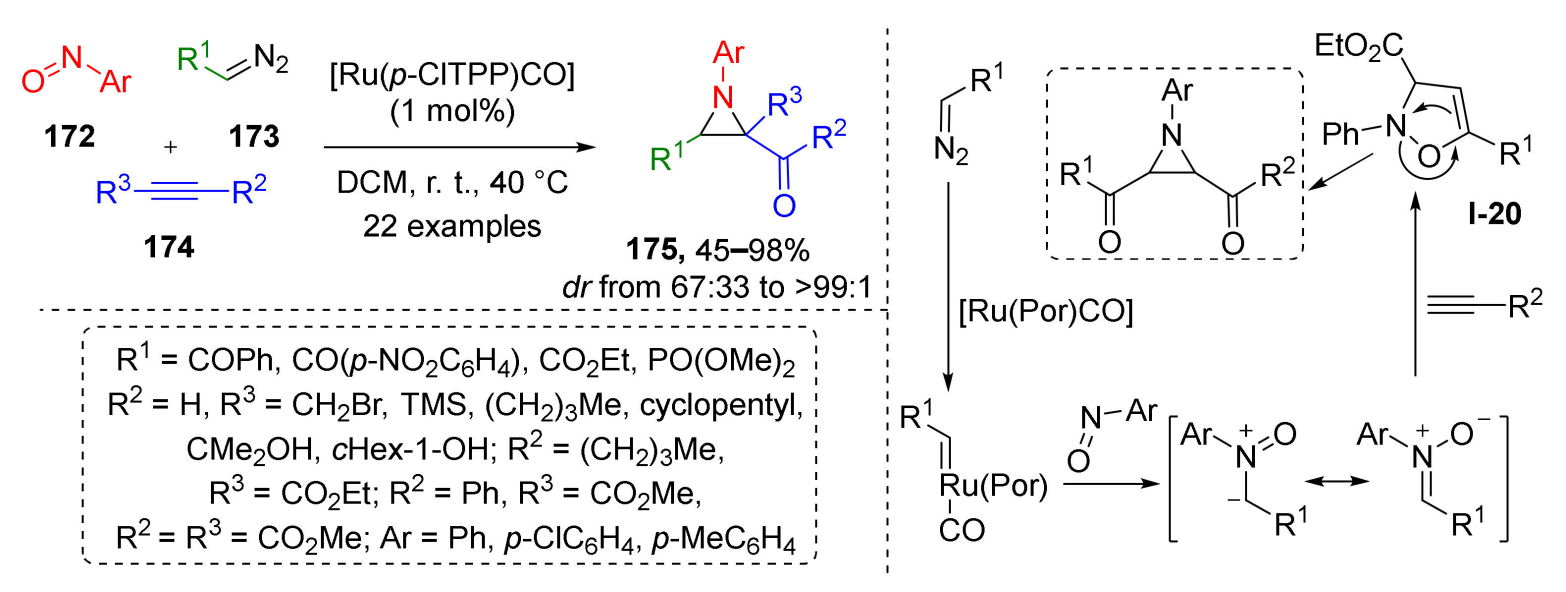
Ruthenium porphyrin catalyzed reaction of diazo compounds, nitrosoarenes, and alkynes.

Although 1,3-dipolar cycloaddition always results in the formation of five-membered rings, its combination with subsequent transformations, such as rearrangements, can be used for the preparation of other classes of heterocyclic compounds. In one such work, the authors demonstrated that specific types of isoxazolines **I-20** undergo isomerization to aziridines **175** (Scheme 50) [58]. The developed three-component protocol included Rh-catalyzed generation of nitrone from nitrosoarene **172** and terminal diazo compound **173** followed by cycloaddition with acetylene **174** to form isoxazoline **I-20**, which underwent thermally-promoted rearrangement to three-membered aziridine cycle **175**. Remarkable reaction scope allowed preparation of a large series of synthetically challenging tri- and tetrasubstituted aziridines in a diastereoselective fashion and high yields. The reaction tolerated the presence of free hydroxy, trialkylsilyl, halogenalkyl, and phosphonate groups. The authors also managed to isolate oxazoline intermediate in one case that supports proposed reaction mechanism.

**Scheme 51:**
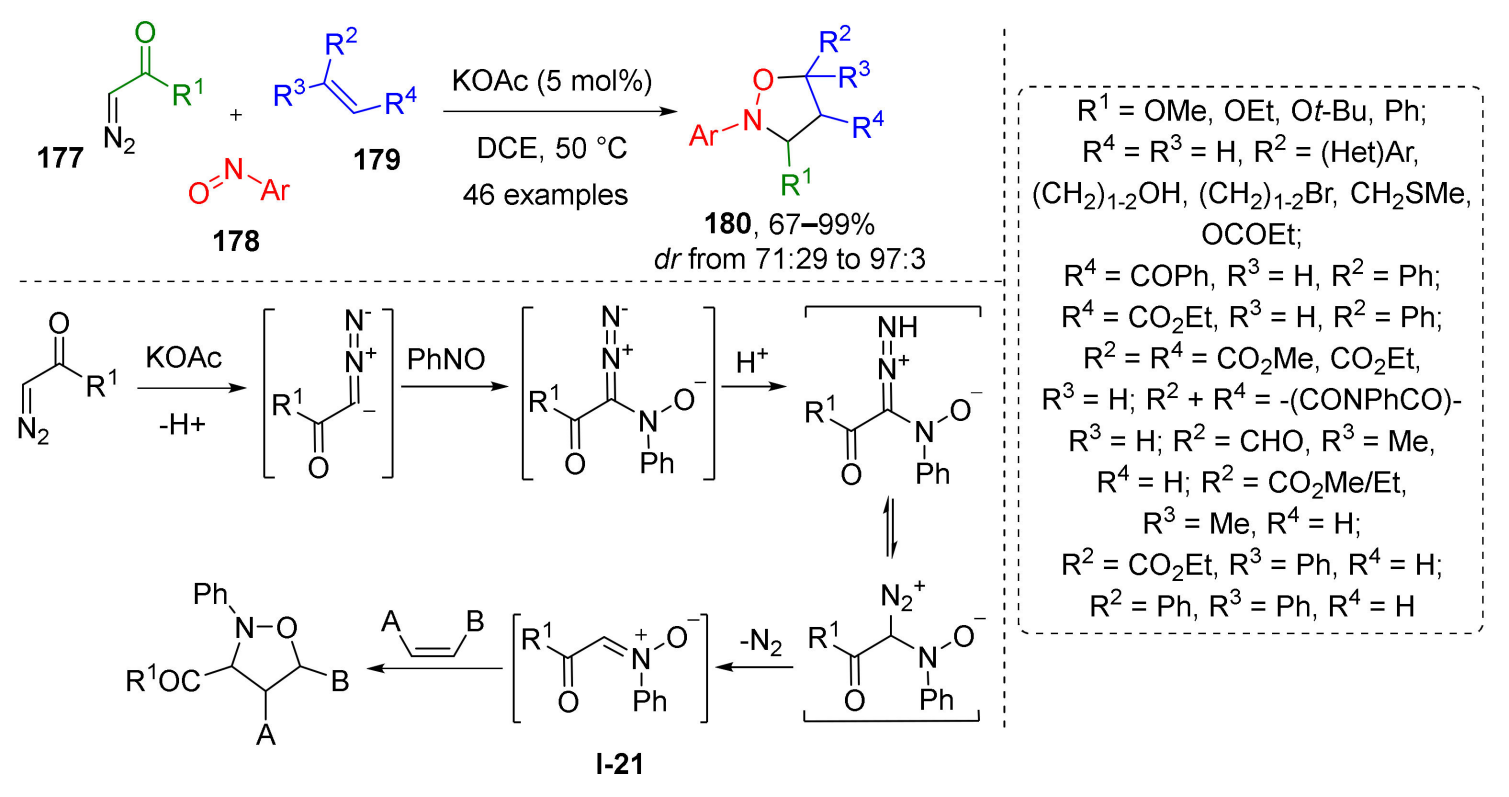
1,3-Dipolar cycloaddition of α-diazo compounds, nitrosoarenes, and alkenes.

1,3-Dipolar addition is also an effective way not only for one-step preparation of isoxazolines, but also for the synthesis of corresponding oxidized and reduced types of heterocycles—isoxazoles and isoxazolidines. To obtain the latter from the same dipole or nitrone, a more saturated dipolarophile is required, namely, an alkene. W. Wei et al. demonstrated an interesting example of transition metal-free [3+2]-cycloaddition of α-alkoxycarbonyl substituted nitrones **I-21** (generated in situ from diazo reagent **177** and nitrosoarene **178**) and mono- or di-substituted alkenes **179** which gave oxazolidines **180** (Scheme 51) [59]. The reaction proceeded under mild conditions and under basic KOAc catalysis in a regio- and diastereoselective fashion. A large scope of alkenes was involved, including both electron-rich and electron-poor ones, as well as those of heterocyclic nature. Remarkably, the reaction was found to be compatible with nucleophilic components, such as free hydroxy groups, and divalent sulfur and pyridine nitrogen atoms.

**Scheme 52:**
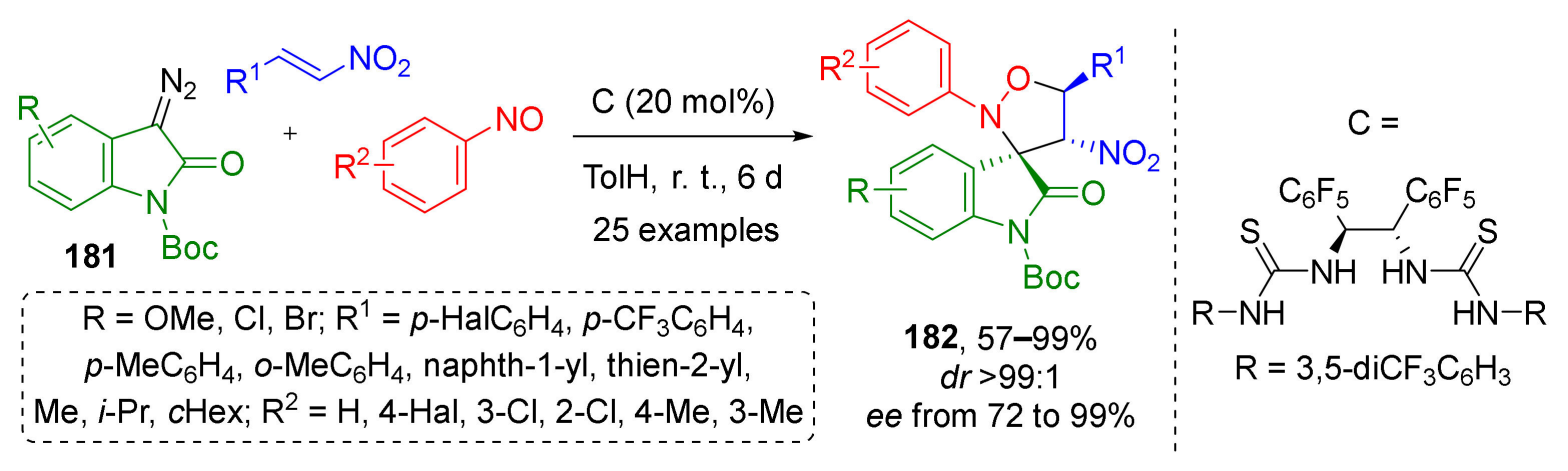
Asymmetric construction of spirooxindoles by multicomponent reactions using diazo oxindoles.

For diazo oxindoles **181** this approach was combined with asymmetric catalysis (Scheme 52) [60]. B. Tan and co-workers identified an effective H-bonding-type bisthiourea organocatalyst C which allowed performing preparation of spirocyclic isoxazolidines **182** with three asymmetric units with excellent ee valuess. In this work, the scope of the olefin component was limited to nitroalkenes with alkyl-, aryl-, or hetaryl groups.

**Scheme 53:**
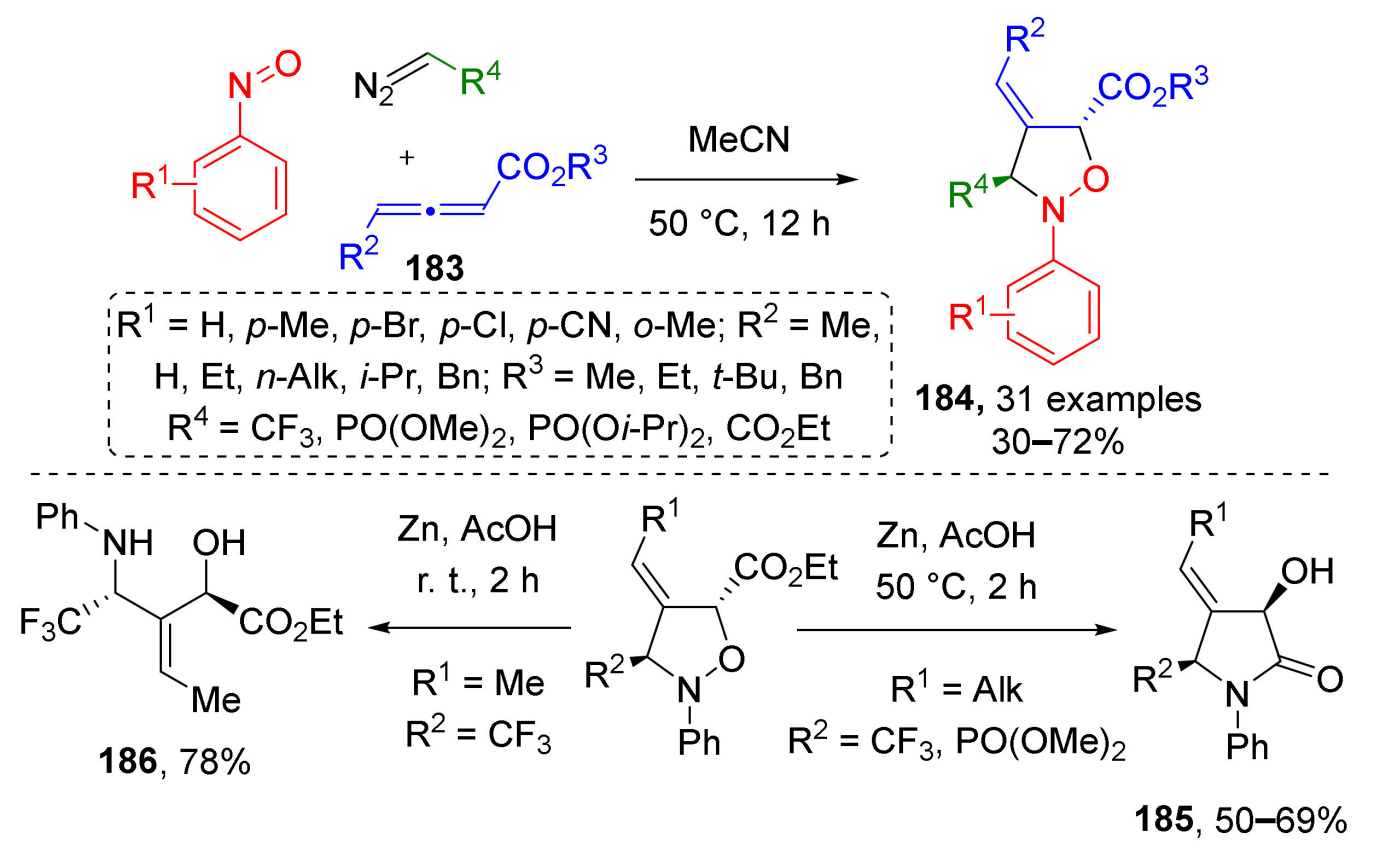
Three-component reaction of diazo compounds, nitrosobenzene, and allenic esters.

A series of isoxazolidines **184** with rare substitution pattern was obtained via the same type of MCR, where dipolarophile was replaced with allene **183** (Scheme 53) [61]. This enabled preparation of products bearing trifluoromethyl or phosphonate group (coming from a diazo compound) combined with an exocyclic double bond (provided by allene). In this case, the reaction required only thermal activation and proceeded without any addition of reagents or catalysts. The authors have also demonstrated that upon reduction with zinc metal under different conditions, the obtained isoxazolidines can serve as platforms for construction of poorly accessible highly functionalized 2-pyrrolidones **185** or α-hydroxy-β-aminoesters **186**. The latter were isolated when reduction was conducted at room temperature, while under heating, they underwent intramolecular amidation to give 2-pyrrolidones.

**Scheme 54:**
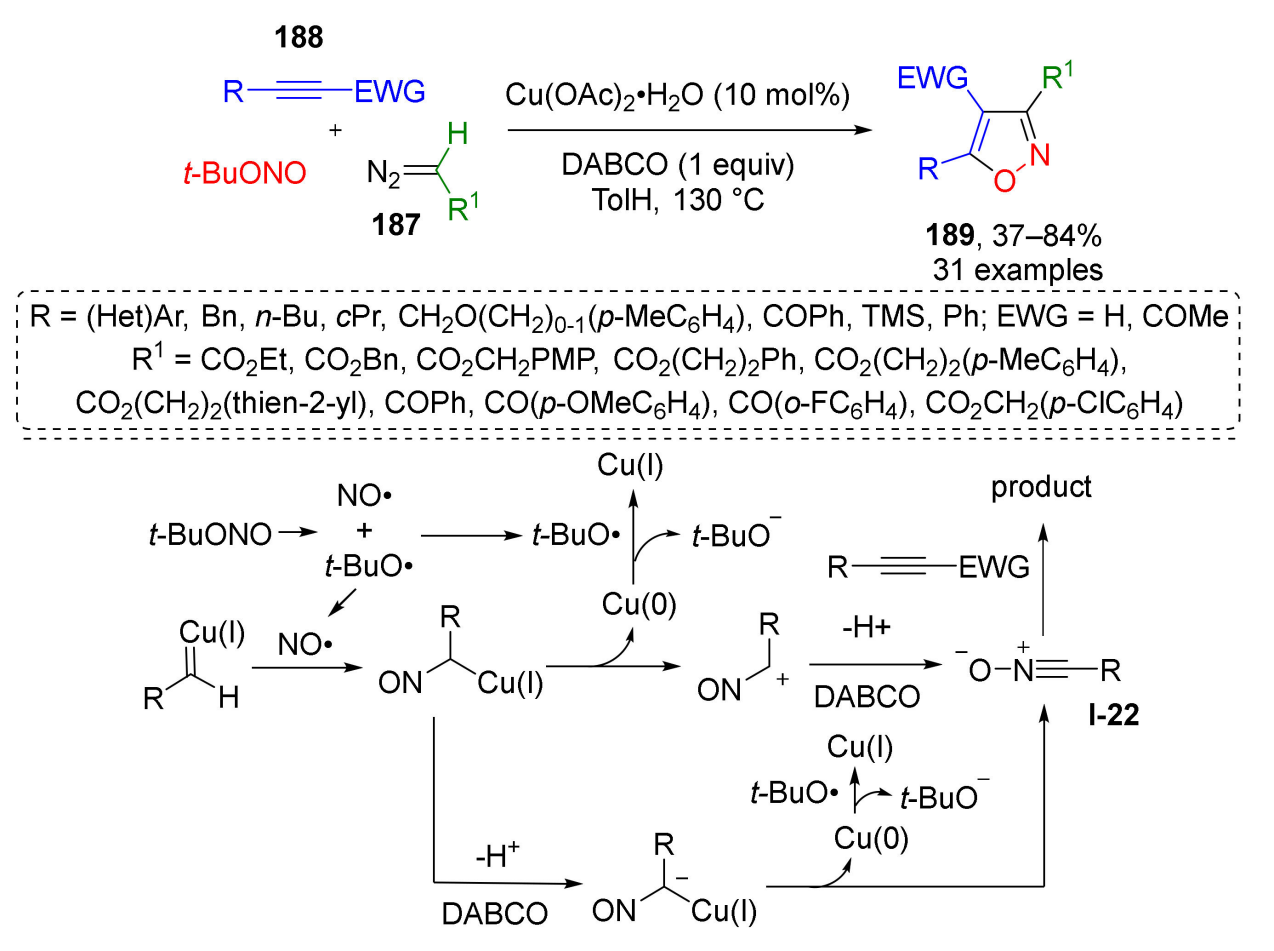
Copper-catalyzed cascade cyclization of diazo compounds with *tert*-butyl nitrite and alkynes.

To achieve direct formation of aromatic isoxazoles via [3+2]-cycloaddition, use of maximum unsaturated substrates is required, which are alkynes and nitrile oxides. The corresponding MCR was developed by Y.-L. Zhao and co-workers in 2019, where 1,3-dipole **I-22** was generated in situ from diazo esters or ketones **187** and *tert*-butyl nitrite under copper catalysis (Scheme 54) [62]. Thus, obtained nitrones further entered regioselective [3+2]-cycloaddition with various acetylenes **188** to afford di- and trisubstituted isoxazoles **189** in a highly regioselective manner.

**Scheme 55:**
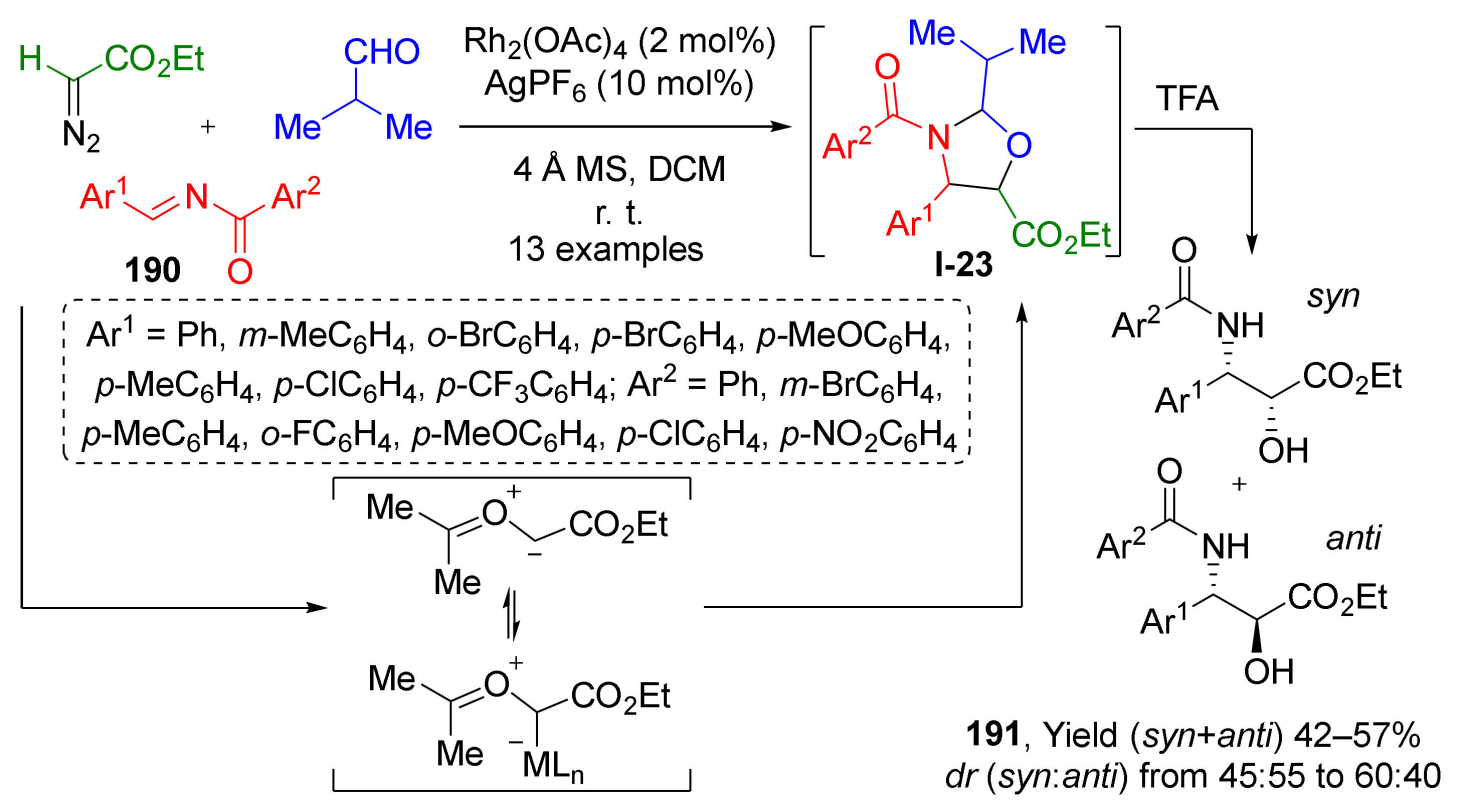
A convenient one-pot approach to Taxol side chain via 1,3-dipolar cycloaddition.

In some cases, [3+2]-cycloaddition strategy can be used for the preparation of acyclic molecules, when initial cyclic products are labile compounds. This principle was employed by W. Hu et al. to access α-hydroxy-β-acylamino esters **191** via Rh/Ag-catalyzed cycloaddition of carbonyl ylides and various aldehydes followed by acid hydrolysis of intermediate oxazolidines **I-23** (Scheme 55) [63]. Ylides were generated in situ from *N*-acylimines **190** and ethyl diazoacetate at room temperature. The hydrolysis was performed in a one-pot fashion upon treatment with TFA after completion of the cycloaddition step. The reaction was found to be rather general and compatible with different types of substituents but suffered from moderate yields and poor diastereoselectivity.

**Scheme 56:**
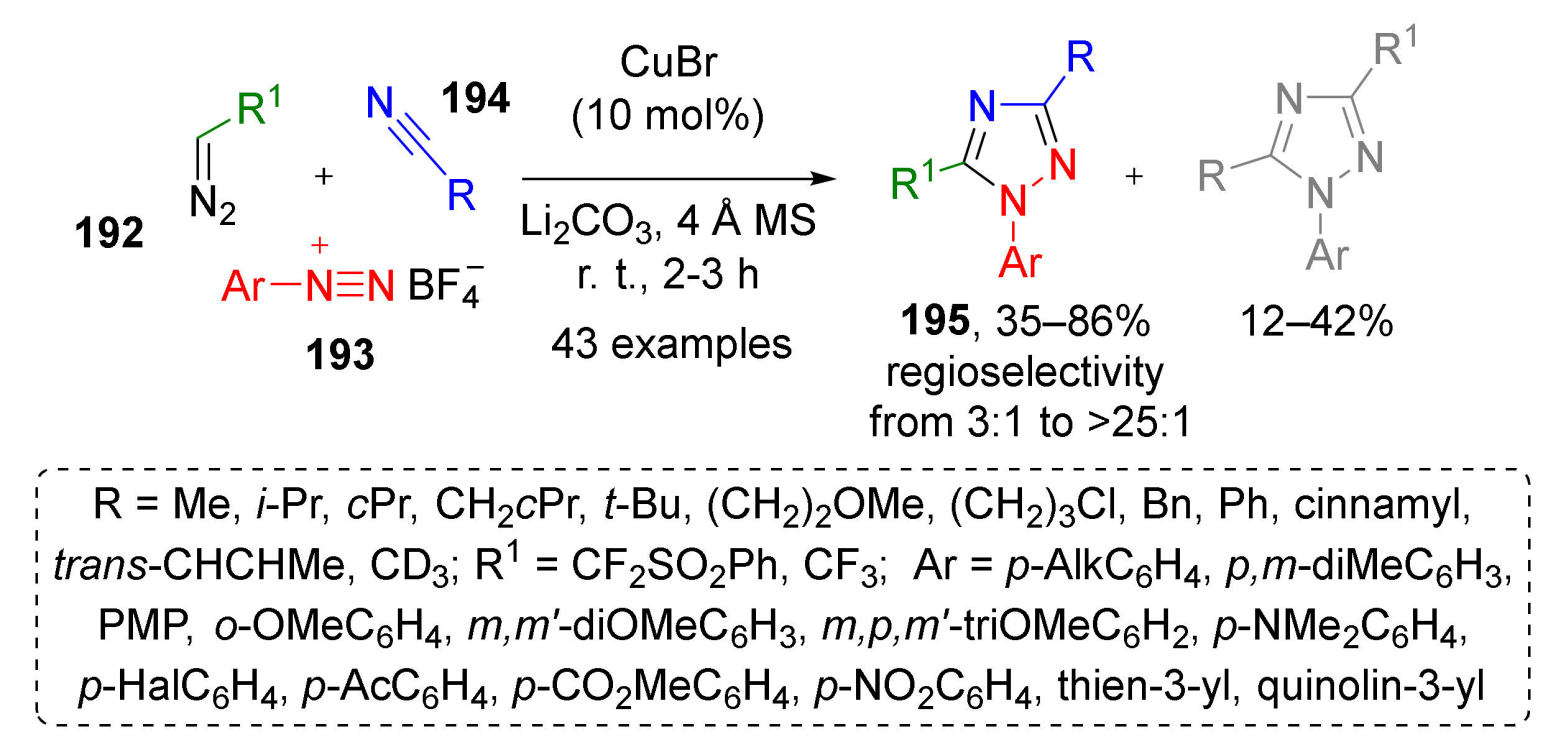
Cu-catalyzed three-component reaction of aryldiazonium salts with fluorinated diazo reagents and nitriles.

Not only carbon containing triple bonds can serve as dipolarophiles for [3+2]-dipolar cycloaddition. An MCR based on corresponding reactivity of diazonium cations was used by J.-A. Ma et al. to develop a novel synthetic approach to specifically substituted triazoles (Scheme 56) [64]. In this Cu-catalyzed protocol, α-di- or trifluoro terminal diazo compounds **192** reacted with arene diazonium salts **193** and aliphatic or aromatic nitriles **194** to form *N*^1^-aryl-1,2,4-triazoles **195** in high yields and moderate regioselectivity. The reaction tolerated a broad scope of substituents, including heterocyclic ones. Mechanistically, it involved generation of dipole from diazo compound and nitrile accompanied by loss of nitrogen molecule followed by cycloaddition with diazonium cation and proton migration.

**Scheme 57:**
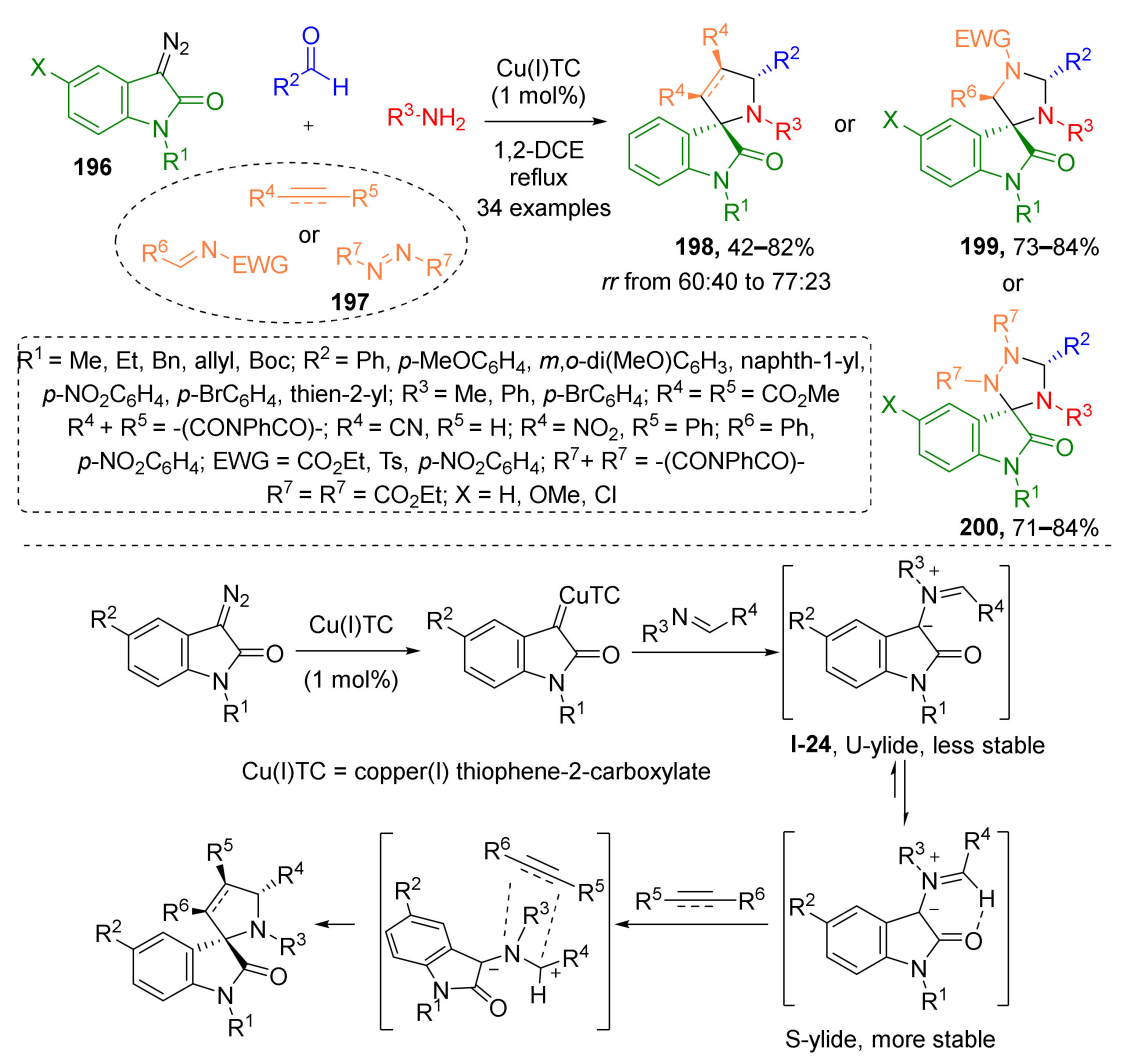
Copper(I)-catalyzed diastereoselective multicomponent synthesis of spiroindolo-pyrrolidines/-imidazolidines/-triazolidines.

Furthermore, 3-Diazooxindoles have proven to be versatile building blocks for construction of various spiroheterocyclic systems via various strategies including [3+2]-cycloaddition. Improvement of such methodologies is in high demand for medicinal chemistry due to multiple examples of structurally related biologically-active compounds being known. In 2016, S. G. Kumar and co-workers developed a copper-catalyzed four component MCR of such diazo compounds **196** with aldehydes, amines and electron poor alkenes, alkynes or diazenes **197** leading to spiroheterocyclic systems **198**–**200** (Scheme 57) [65]. In this reaction sequence, an imine was generated in the first step followed by reaction with diazo compound-derived Cu-carbenoid resulting in formation of azomethine ylide **I-24**. The latter was trapped by dipolarophile—a double or triple bond activated with CN, NO_2_, or carbonyl groups. This cascade afforded three types of indolinone-based tri- and tetracyclic spirocompounds. Corresponding newly constructed spirocyclic moieties include pyrrolidine (products **198**), imidazolidine (products **199**), and or 1,2,4-triazolidine (products **200**).

**Scheme 58:**
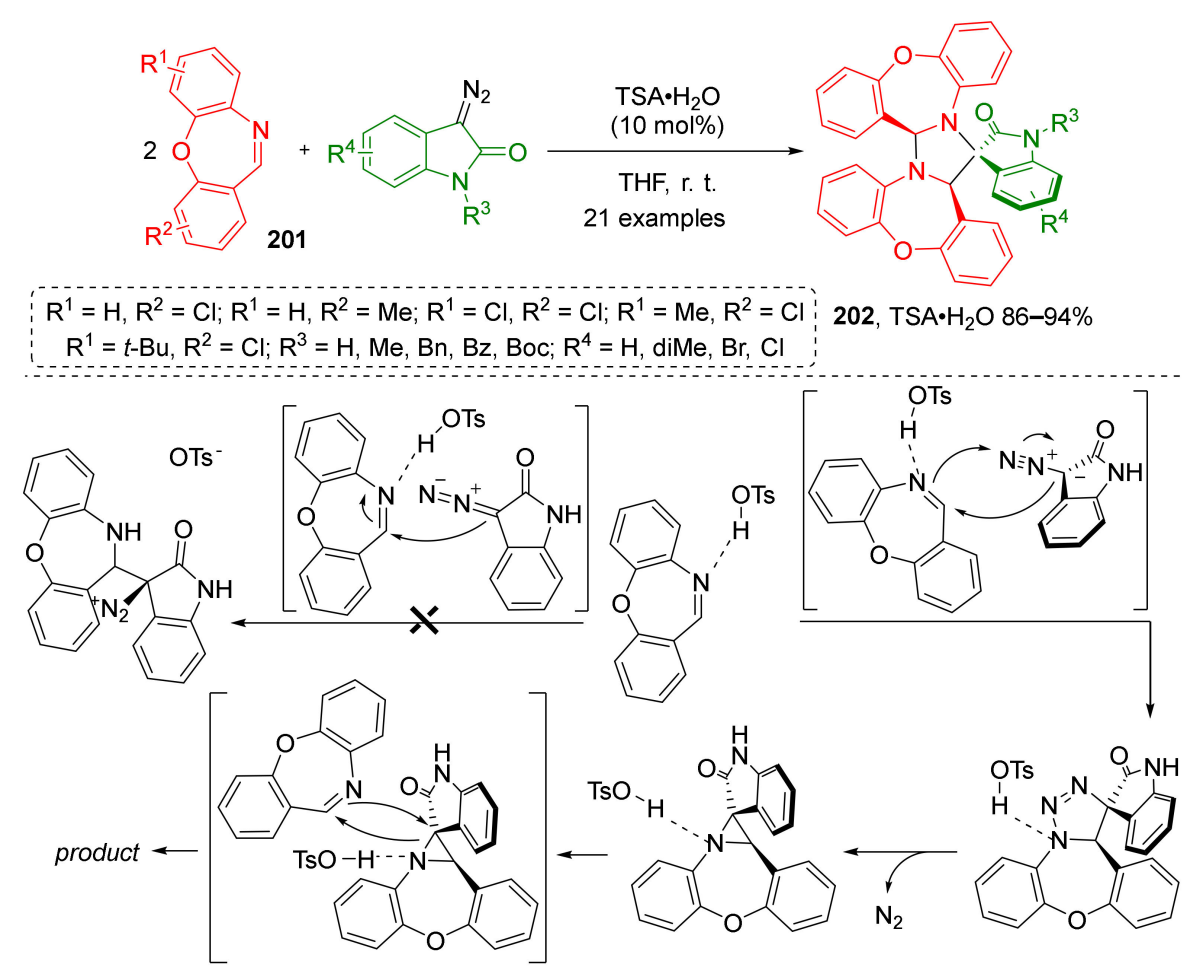
Three-component [2+2+1] cascade reaction of 1,4-oxazepines with diazo-oxindoles.

Cyclic imines were also shown to be suitable dipolarophiles for transformations similar to the ones described above. X.-W. Wang et al. introduced two equivalents of 1,4-oxazepines **201** to this strategy—one to generate ylide and another as a dipolarophile (Scheme 58) [66]. This opened a straightforward route to the construction of complex polycyclic system **202** containing two seven-membered cycles. Remarkably, all reaction products were isolated as single diastereomers despite the presence of three asymmetric centers. Another advantage of this methodology was the use of a Brǿnsted acid (*p*TSA) instead of a transition metal catalyst. The authors also proposed the reaction mechanism supported by quantum chemical calculations.

**Scheme 59:**
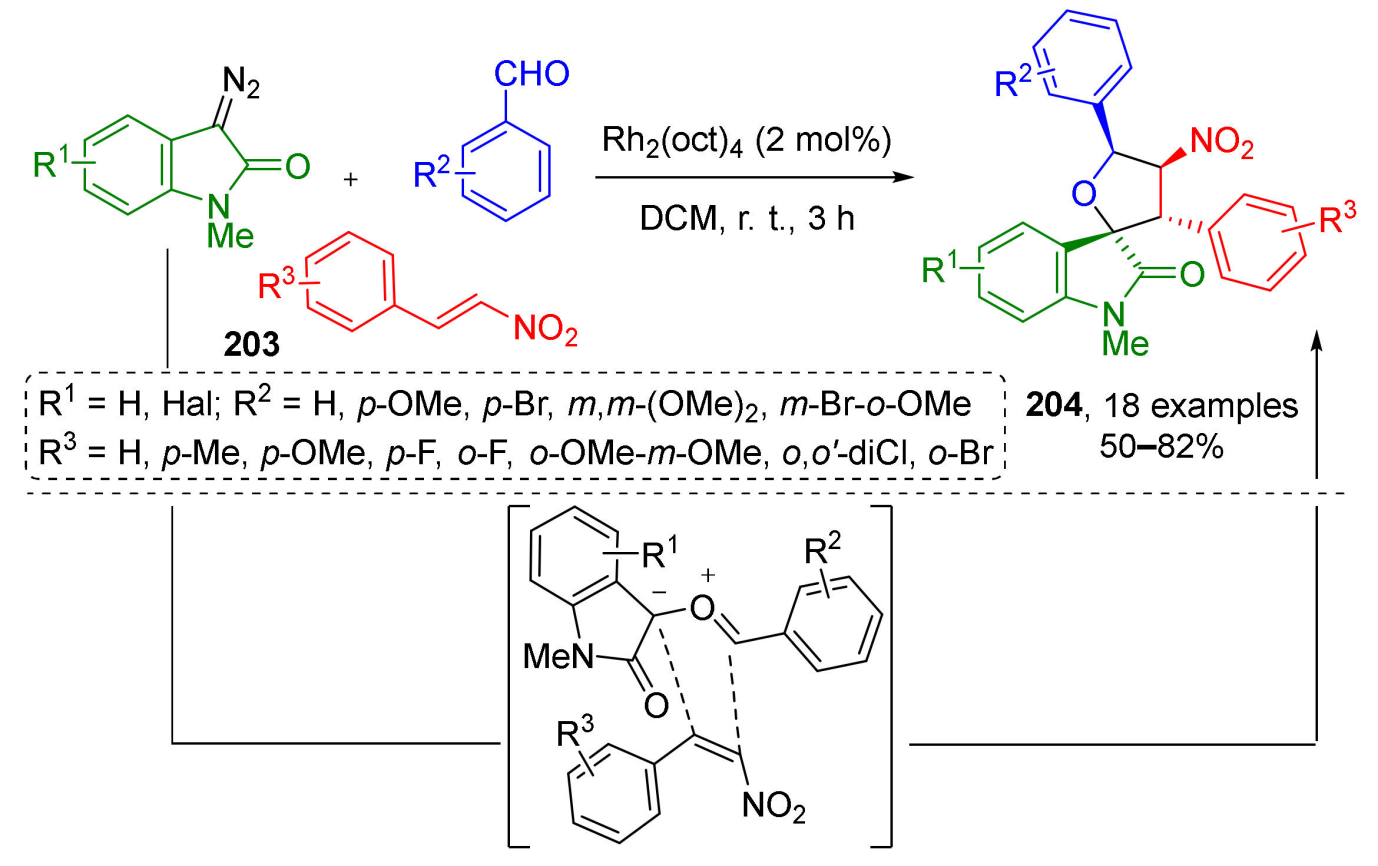
Biology-oriented synthesis of 3,3-spiro(2-tetrahydrofuranyl)oxindoles.

Furthermore, [3+2]-cycloaddition is an effective way for the construction of not only aza-, but oxaheterocycles. A series of works on the synthesis of spiro-annulated tetrahydrofuranes (THFs) was published recently. In such MCRs, diazo oxindole reacts with aldehydes to produce carbonyl ylide which further enters formal cycloaddition with dipolarophile forming a five-membered ring (Scheme 59) [67]. In 2016, H. Waldmann et al. published an approach to nitro substituted spiro-THFs **204** based on using nitrostyrenes **203** as dipolarophiles. This Rh-catalyzed MCR was found to be general and tolerated a wide scope of substituents in all three components. The products containing four asymmetric carbon atoms were isolated as single diastereomers.

**Scheme 60:**
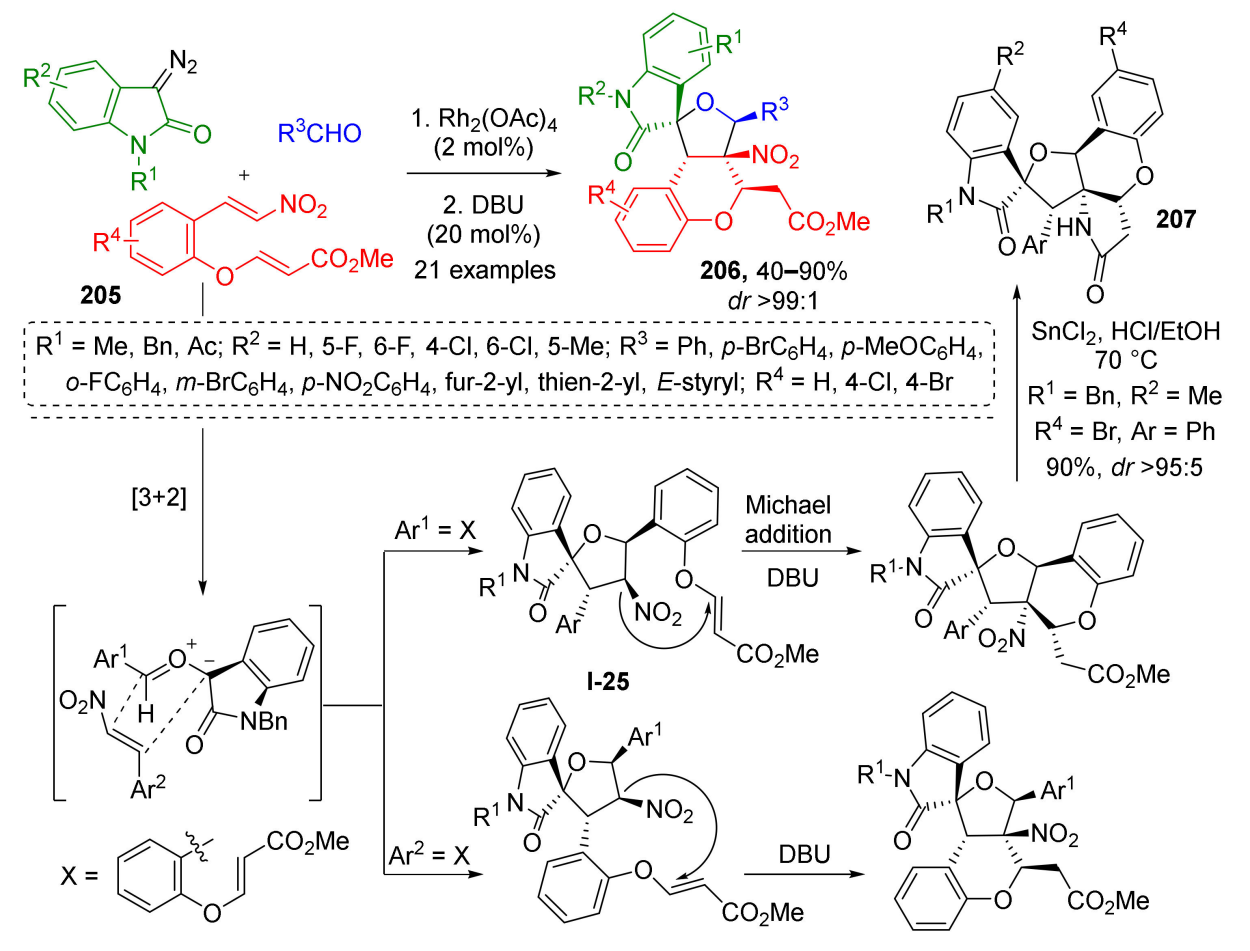
Three-component cascade reaction to construct oxindole-fused spirotetrahydrofurochroman scaffolds.

An interesting improvement of this methodology was developed by W. Hu et al. by introducing a Michael acceptor in the *ortho*-position of aldehyde **205** (Scheme 60) [68]. This enabled annelation of chromane system to the same scaffold via DBU-promoted conjugate addition at the last step of cascade resulting in formations of compounds **206**. The reaction demonstrated excellent diastereo-, regio-, and chemoselectivity. The latter corresponds to the fact that only nitrostyrene double bond entered cycloaddition and the acrylate moiety remained intact. To prove the sequence of transformations in this cascade the authors conducted the reaction without base and corresponding non-annelated spiroTHF **I-25** was isolated, thus identifying Michael addition as the final step. The obtained compounds were also evaluated [69] as protein tyrosine phosphatase 1B inhibitors—promising agents for treatment of type II diabetes and obesity. The authors also showed that simple reduction of nitro group with SnCl_2_ followed by spontaneous intramolecular amidation opens access to even more structurally complex polycyclic systems **207**.

**Scheme 61:**
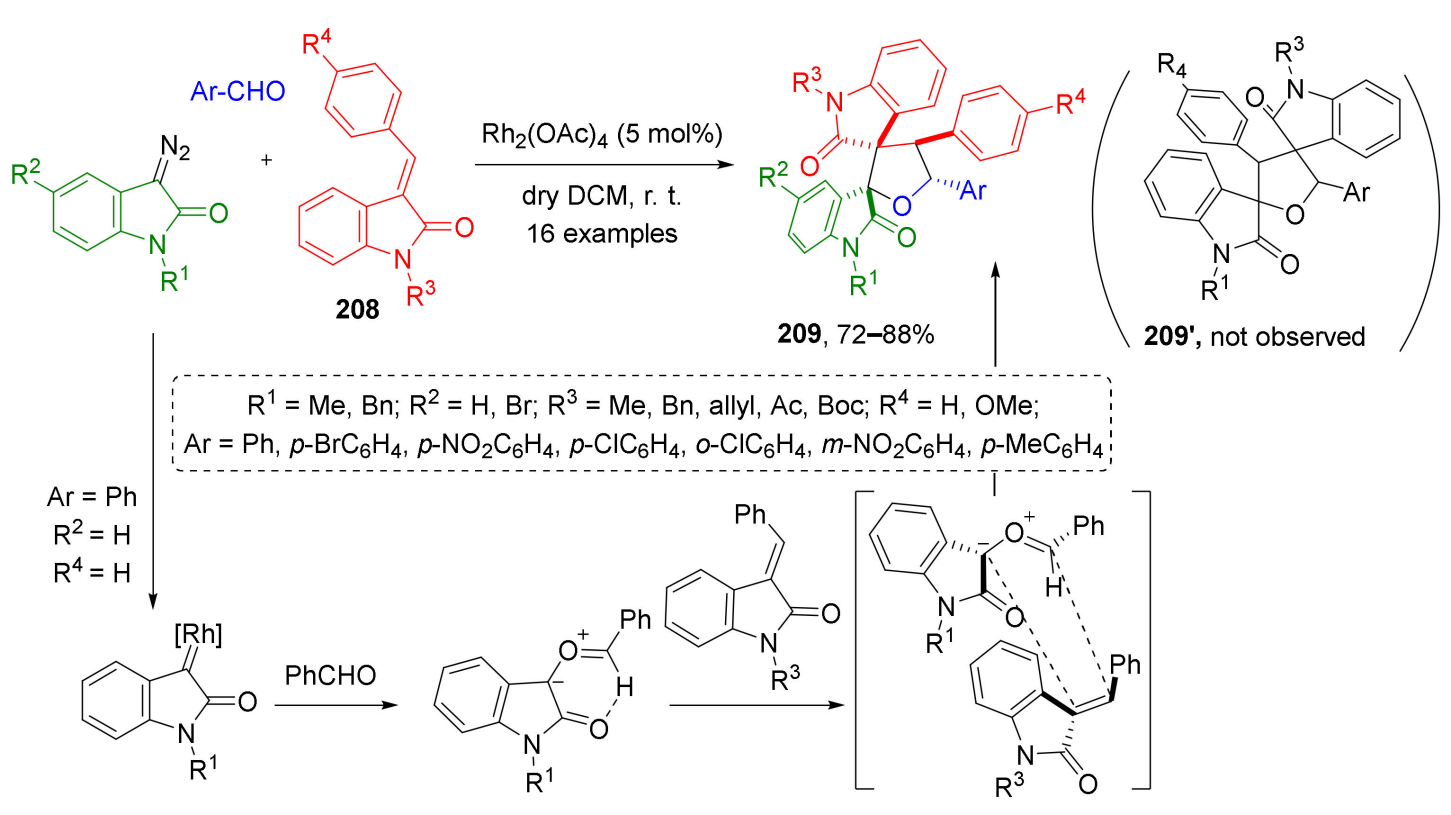
[3+2]-Cycloaddition of carbonyl ylides to 3-arylideneoxindoles.

The use of isatin-derived 1,1-disubstitued alkenes **208** as dipolarophiles in the discussed strategy gives access to complex THF-based systems with two spirocyclic moieties **209** (Scheme 61) [70]. In this case, the reaction proceeded with complete but unexpected regioselectivity—products **209** were formed instead of **209′** due to attack of nucleophilic carbon center of carbonyl ylide to disubstituted atom at CC-double bond of dipolarophile.

**Scheme 62:**
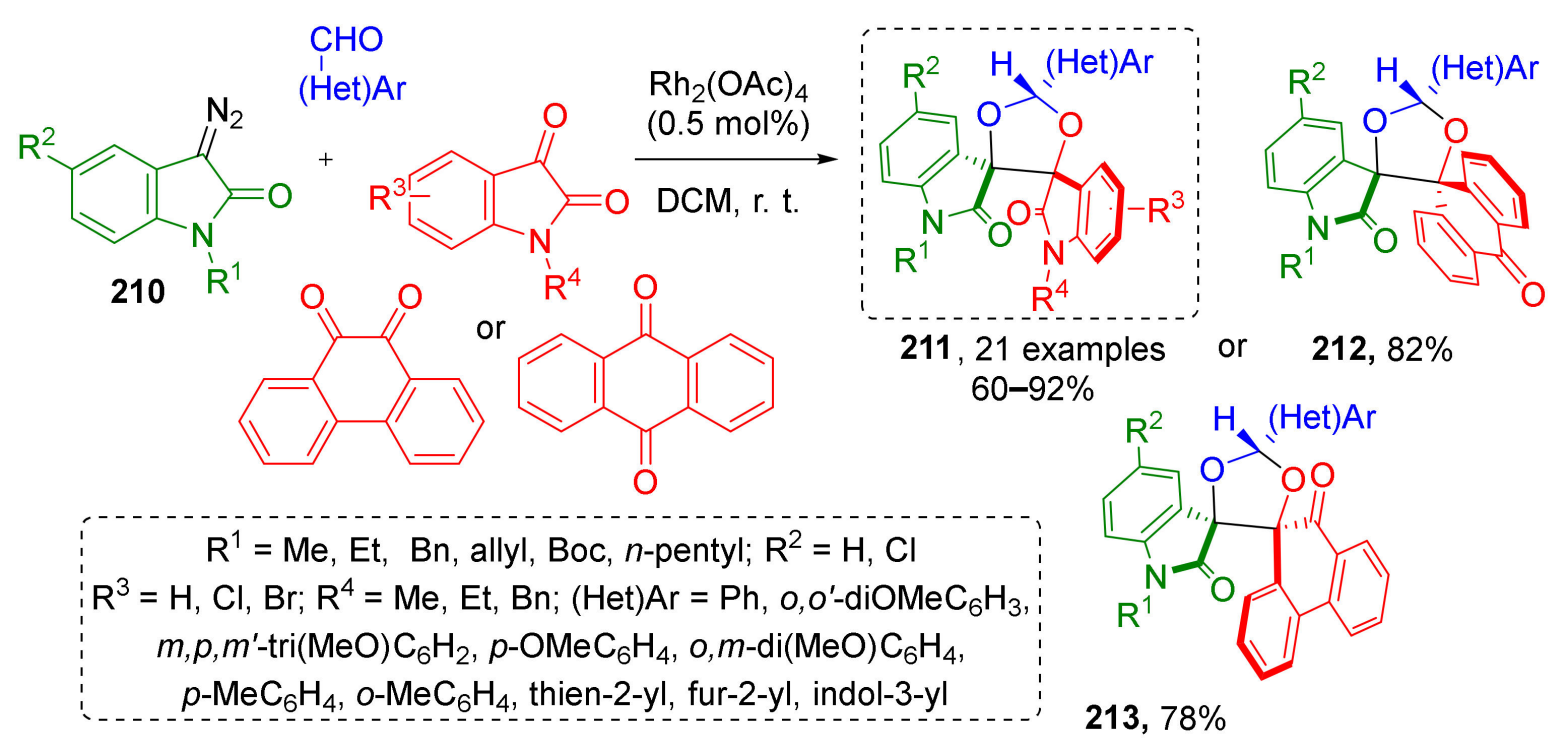
Highly diastereoselective synthesis of conformationally restricted dispiro[1,3-dioxolane]bisoxindoles.

An interesting example of diazo oxindole-based MCR was demonstrated by R. Ramkumar and co-authors. This MCR allowed facile preparation of polyheterocyclic systems **212**–**213** with 1,3-dioxolane core and two spirocyclic centers (Scheme 62) [71]. Diazo compounds **210** reacted with aromatic or heteroaromatic aldehydes and cyclic diketones under Rh-catalysis in DCM at room temperature. The scope of diketones included various isatins, anthraquinone, acenaphthenequinone and phenanthrenequinone. Remarkably, this reaction also worked with dialdehydes giving even more complex dimeric systems. All reactions proceeded with high diastereoselectivity.

### 3.5. Generation of Ylides Followed by Other Types of Reactions

**Scheme 63:**
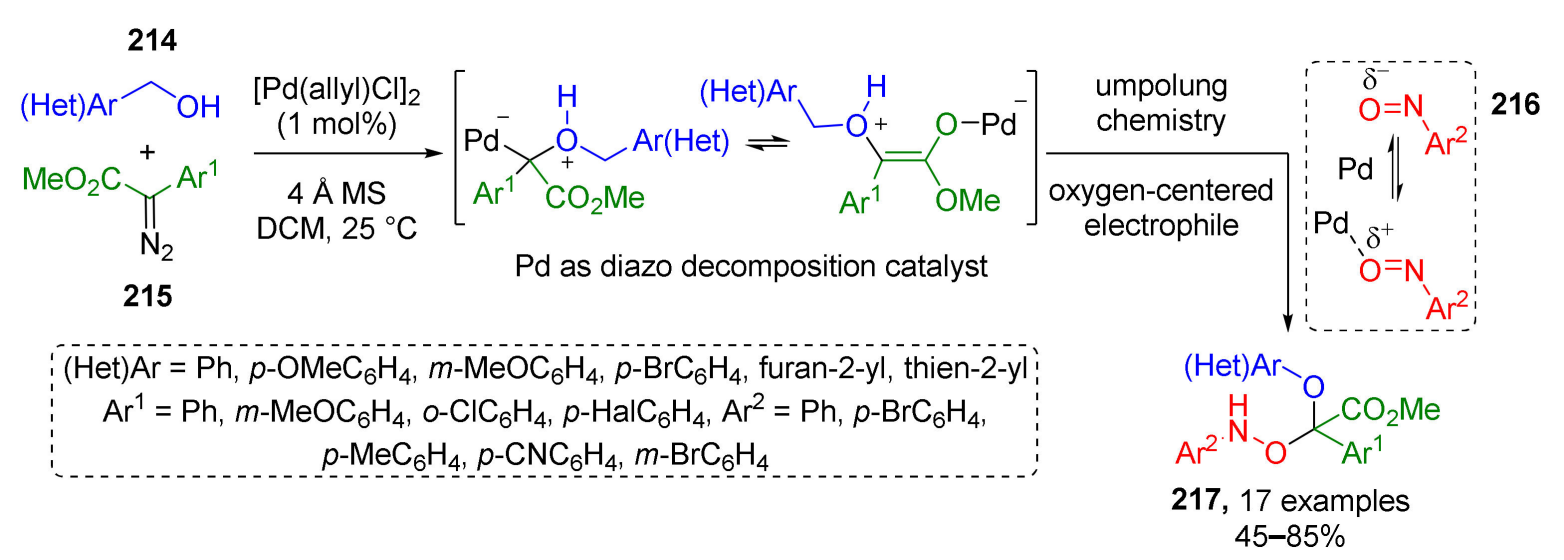
Pd-catalyzed multicomponent reaction of oxonium ylide with nitrosobenzene.

In previous sections, four main types of agents for trapping oxonium ylides were discussed, but recently some additional types have also been discovered, e.g., nitroso compounds. In 2021, S. Liu et al. described such a novel MCR which was based on interaction of benzylic alcohol **214**, diazo esters **215**, and nitrosoarenes **216** (Scheme 63) [72]. This MCR provided complex *O*-substituted *N*-arylhydroxylamines **217** via Pd-promoted umpolung chemistry. Oxonium ylide was first generated from alcohol and diazo compound followed by trapping with nitrosobenzene. One of the reaction features is nitroso compound acting as oxygen-centered electrophile. Such reversed polarity of oxygen atom was explained by its coordination to Lewis acid (Pd(II)). Noteworthy, the obtained compounds exhibited anti-osteosarcoma activity.

**Scheme 64:**
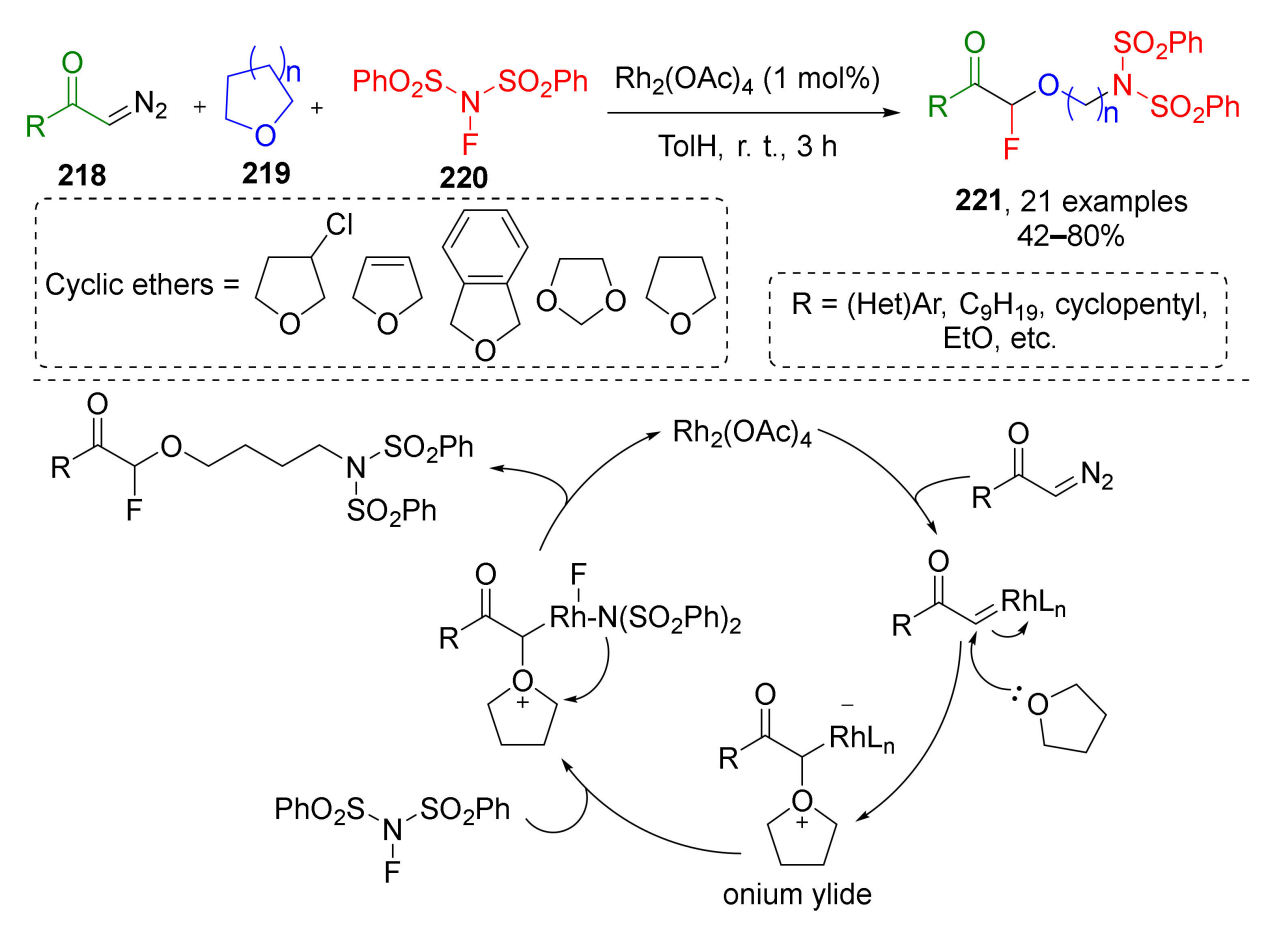
Oxy-aminofluorination of diazo ketones with cyclic ethers and NFSI.

Oxonium ylides derived from cyclic ethers often undergo another type of transformations—ring-opening reactions. K. J. Szabo et al., in 2016, utilized this approach for Rh-catalyzed oxy-aminofluorination of diazo ketones **218** (Scheme 64) [73]. Two other reactants for this MCR were four- or five-membered cyclic ethers **219** (including oxetanes, di- and tetrahydrofuranes, and 1,3-dioxolanes) and *N*-fluorobenzenesulfonimide **220**. The latter provided two components of the product molecule **221**—fluorine atom at α-position to carbonyl group and N(SO_2_Ph)_2_ to ω-. The scope of diazo ketones included both cyclic and acyclic molecules with alkyl or aryl groups at carbonyl atom. The authors also attempted to expand the reaction to diazo esters, but they showed insufficient reactivity.

**Scheme 65:**
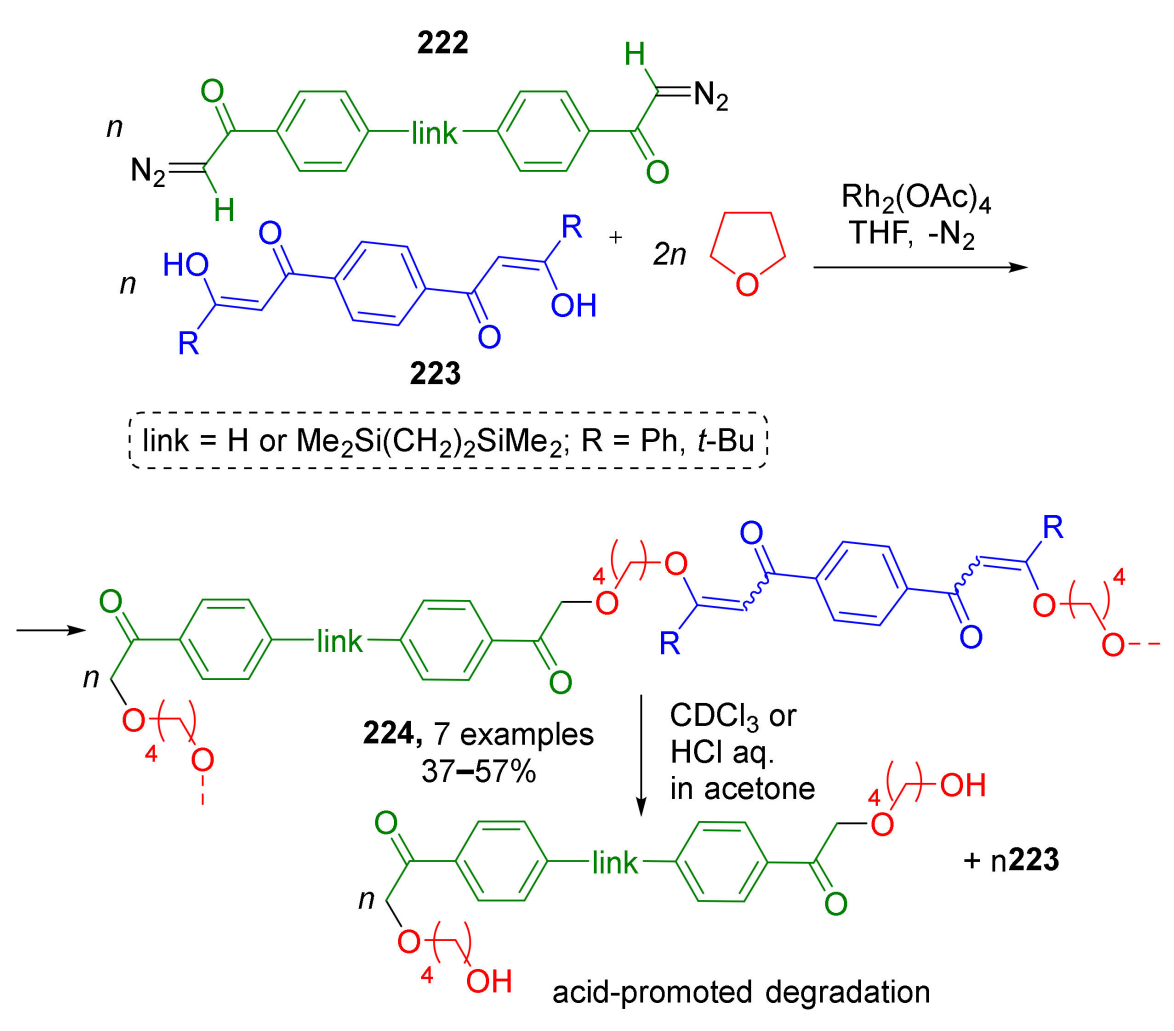
Poly(β-keto enol ether) prepared by three-component polycondensation of bis(diazo ketone), bis(1,3-diketone), and tetrahydrofuran.

A similar strategy based on Rh-catalyzed ring-opening transformations of ylides derived from diazo ketones and THF was applied to synthesis of polymeric compounds by E. Ihara et al. in 2019 (Scheme 65) [74]. Polymerization was achieved by using difunctional building blocks—bis(diazo ketones) **222** and bis(1,3-diketones) **223**. The latter reacted as nucleophilic component due to the presence of enol form. Not every polymerization can be referred to as MCR but in this case the term is applicable due to the structure of monomer as it is composed of three reactants. The authors also found the obtained polymeric products to be acid-degradable with elimination of 1,3-diketone blocks due to protonation of enolic oxygen atom.

**Scheme 66:**
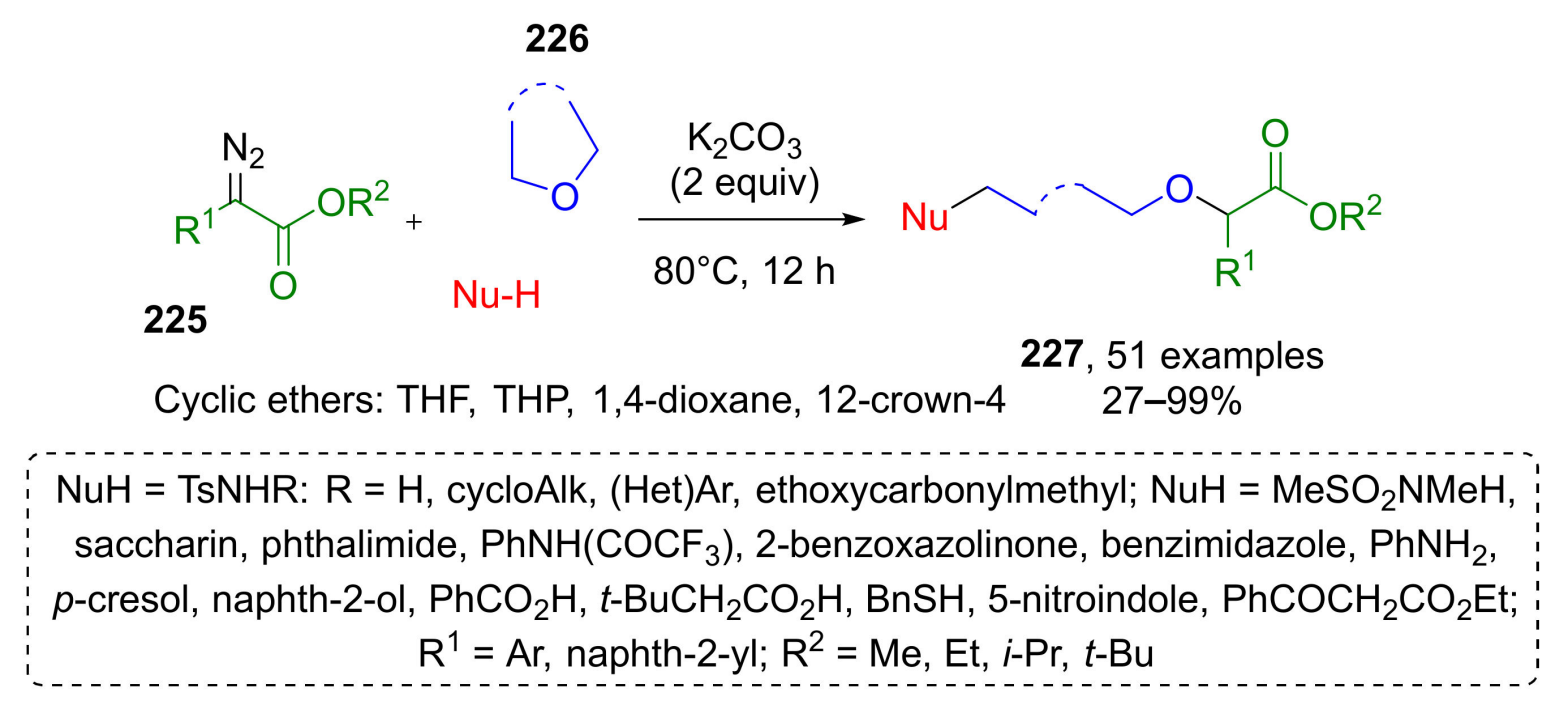
Three-component ring-opening reaction of cyclic ethers, α-diazo esters, and weak nucleophiles.

α-Diazo esters were also modified via ring-opening transformation in another MCR developed by B. Yin and co-workers (Scheme 66) [75]. In this case, the reaction was base-promoted. A large scope of α-aryl-α-diazoacetates **225** and cyclic ethers **226** reacted to form oxonium ylides followed by nucleophilic ring-opening reaction. The authors demonstrated a remarkable scope of suitable nucleophilic components—more than fifteen different *O*-, *N*-, *S*- and *C*-centered nucleophiles, even poorly reactive ones such as sulfonimides, carbamates, and carboxylates, gave corresponding products **227** in high yields. Various cyclic ethers were also tested in this reaction, namely, tetrahydrofuran, 1,4-dioxane, oxetane, and 12-crown-4, but only the first two succeeded.

**Scheme 67:**
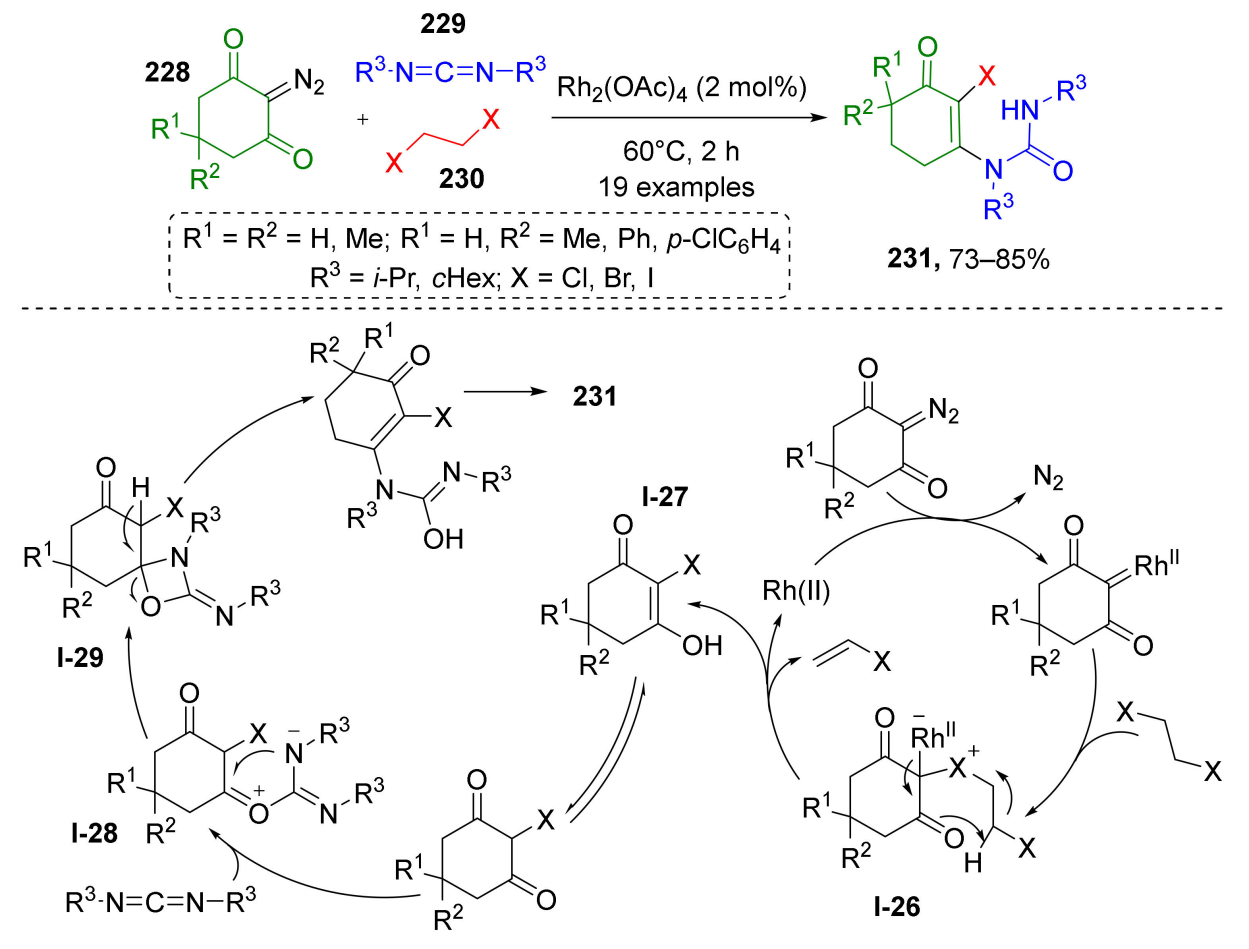
Three-component reactions of cyclic 2-diazo-1,3-diketones, carbodiimides, and 1,2-dihaloethanes.

A quite rare type of MCR based on the formation of halonium ylide was developed recently by Y. Shang and co-workers (Scheme 67) [76]. The reaction involved 2-diazo-1,3-diketones **228**, dialkyl carbodiimides **229**, and 1,2-dihaloethanes **230** to produce complex unsymmetrical halogenated ureas **231**. Interestingly, dihaloethanes served only as halogen donor (Cl, Br or I)—no carbons were incorporated into the product structure. The reaction was found to be compatible with H, alkyl, or aryl substituents in position 4 of the diazo compound. The proposed reaction mechanism starts with the formation of Rh-carbenoid from diazo reagent followed by its reaction with dihaloethane which results in formation of halonium ylide **I-26**. The next step is elimination of vinyl halogenide and formation of intermediate **I-27** which undergoes *O*-alkylation with carbon electrophilic center of carbodiimide. The resulting zwitter-ionic intermediate **I-28** is then transformed into spiro 4-oxazetidine **I-29**, which after ring-opening and tautomerization, gives products **231**. Interestingly, the initial binding of carbodiimide to halo-diketone substrate occurs via C-O bond, but in the final product these moieties are connected via C-N bond and one of the keto-groups is transferred to ex-carbodiimide component.

**Scheme 68:**
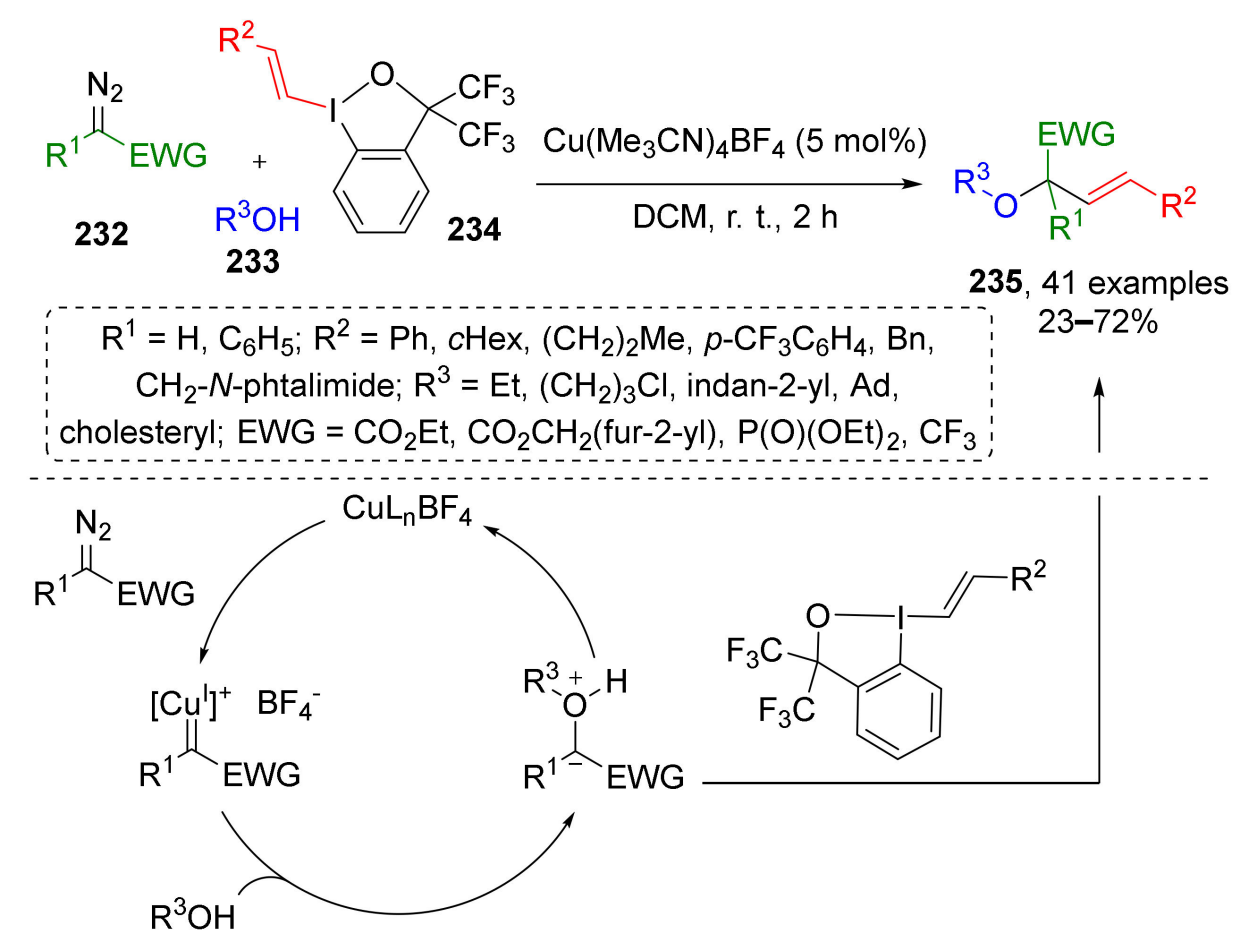
Copper-catalyzed oxyvinylation of diazo compounds.

In 2020, J. Waser and co-workers reported a three component Cu-catalyzed reaction of terminal diazo compounds **232** (CO_2_R, CF_3_ or P(O)OEt_2_ substituted), aliphatic alcohols **233** and vinylbenziodoxolones **234**, which allowed preparation of structurally diverse allylic ethers **235** (Scheme 68) [77]. The above-mentioned hypervalent iodine compounds served as vinyl moiety donors. The key steps of this transformation were the generation of Cu-carbenoid from diazo compound, formation of oxonium ylide with alcohol, followed by the transfer of the vinyl group from iodine reactant.

**Scheme 69:**
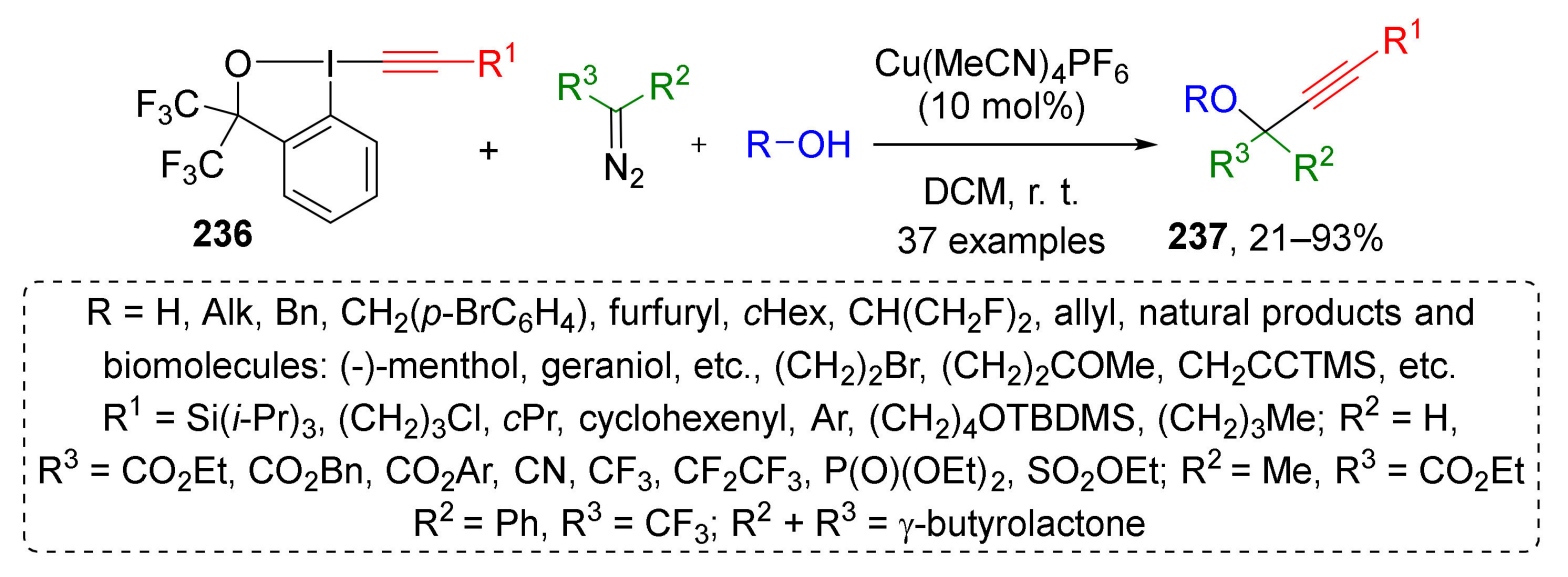
Three-component reaction for the synthesis of highly functionalized propargyl ethers.

This approach was also extended to the synthesis of highly functionalized propargylic ethers **237** by the same group (Scheme 69) [78]. Substituted acetylene moiety was introduced to product molecule using structurally related hypervalent iodine reactant **236**. The reaction proceeded under the same conditions—Cu(CH_3_CN)_4_PF_6_ in DCM at room temperature. In this work, the scope of diazo component was extended using diazo acetonitrile and terminal diazo sulfonic ester along with disubstituted diazo compounds. In the acetylene component, the silyl, alkyl, and aryl groups were well tolerated. The authors also showed that complex natural alcohols, such as hydroxy amino acids, terpenols, sterols, and nucleosides can be successfully involved in the developed MCR, thereby making it very attractive for the design of biologically active compounds.

**Scheme 70:**
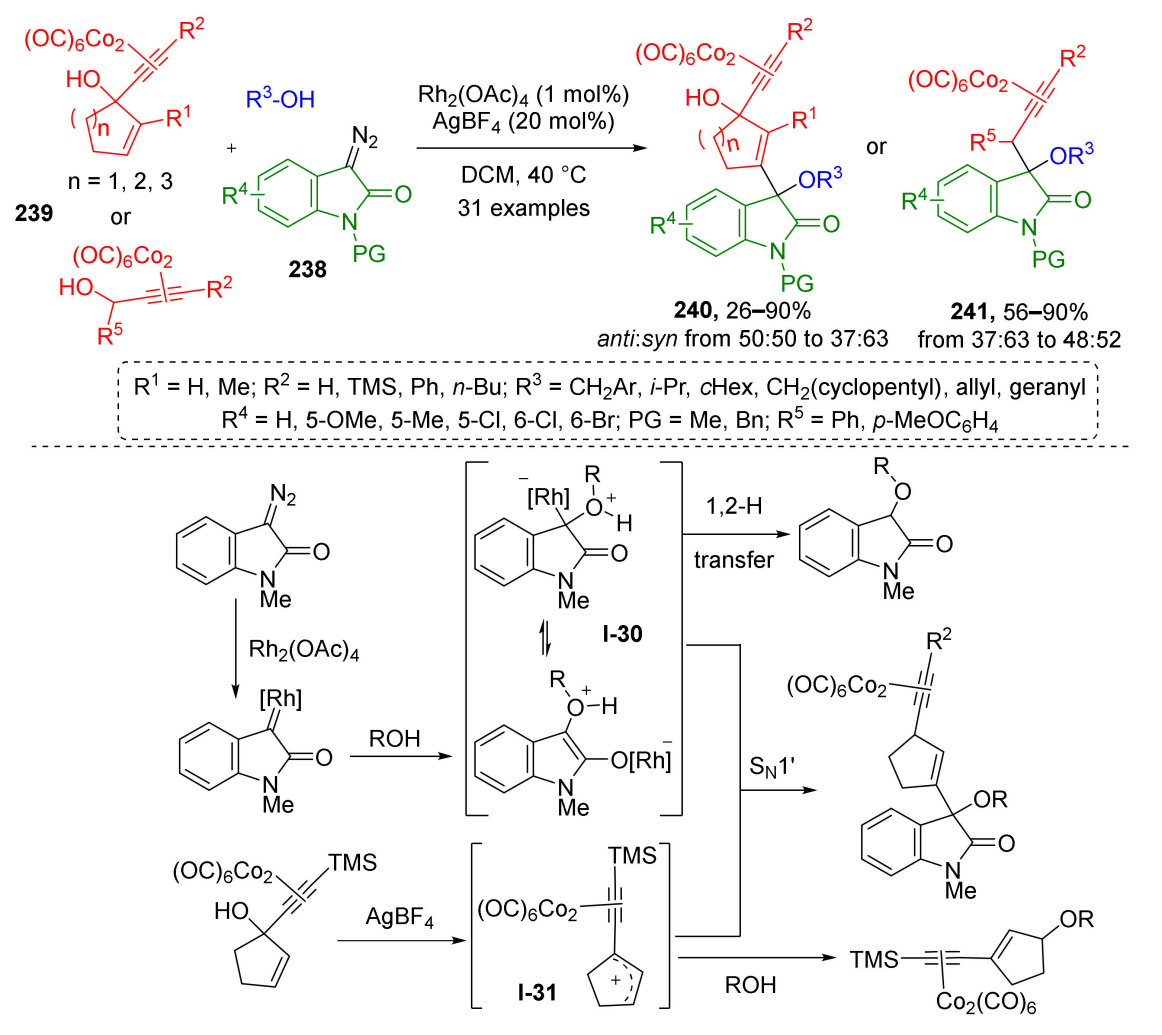
Rh(II)/Ag(I) co-catalyzed trapping of oxonium ylide with Nicholas intermediate.

Several works on trapping of oxonium ylides by allylic cations were recently published, which revealed novel remarkable methods for modification of indolone core. W. Hu et al., in 2020, showed that oxonium ylides **I-30** generated from diazo oxindoles **238** and aliphatic alcohols can enter nucleophilic substitution with enyne-type organocobalt compounds **239** under dual Rh/Ag catalysis providing indolones **240** and **241** with quaternary carbon atom at position 3 (Scheme 70) [79]. The reaction proceeds via the formation of cation **I-31**, which is also a typical intermediate for Nicholas reaction. The developed reaction was found to be general regarding the scope of tolerated substituents at alkoxy and enyne moieties, but suffered from low diastereoselectivity.

**Scheme 71:**
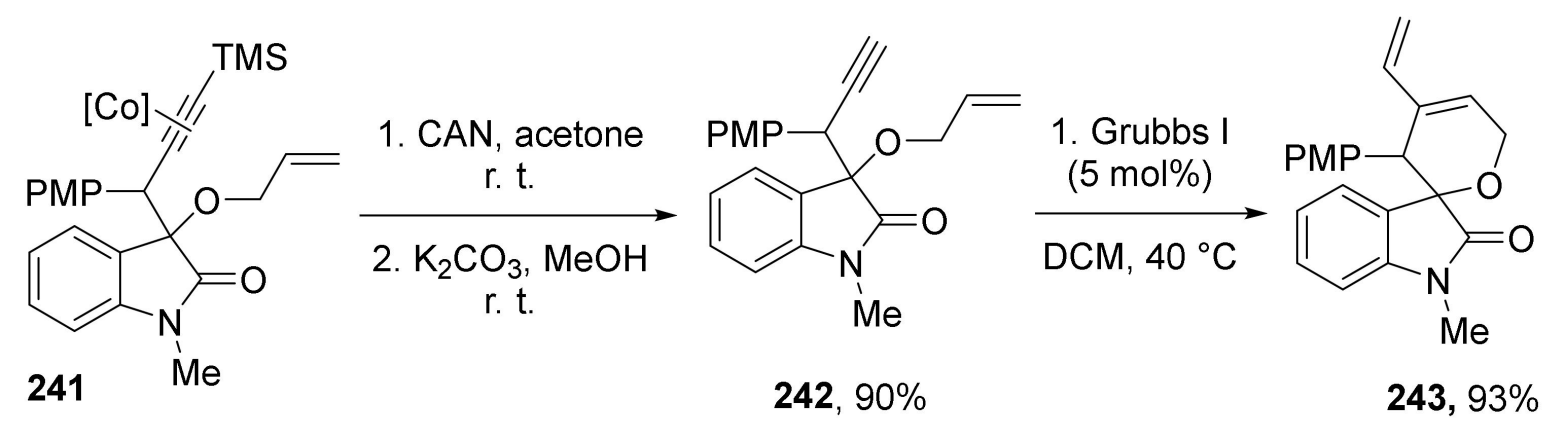
Synthetic utility of the obtained protected acetylenes.

**Scheme 72:**
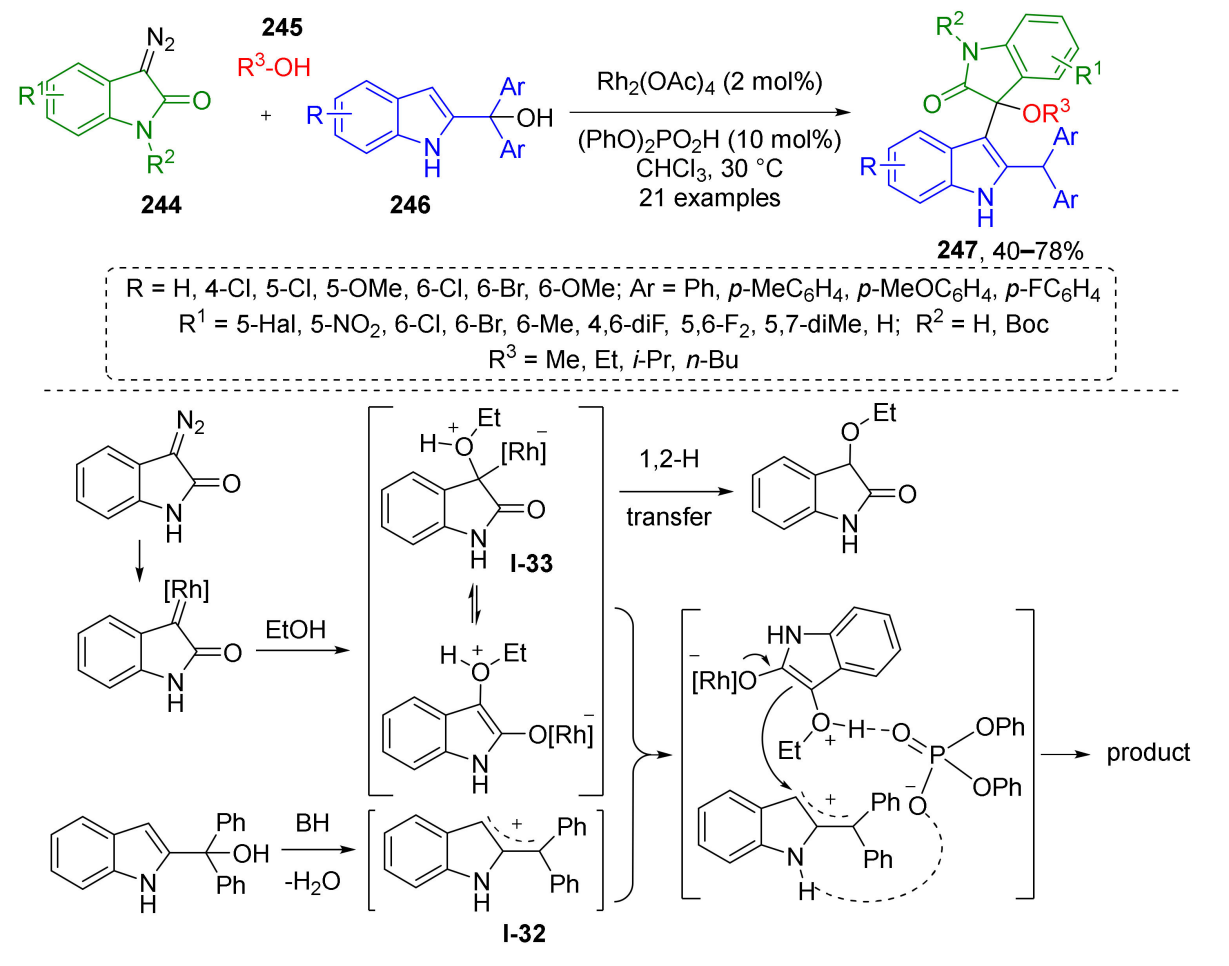
A synergistic catalysis-enabled reaction of 2-indolylmethanols with oxonium ylides.

The authors also demonstrated that initial reaction products—TMS protected and Co-coordinated acetylenes **240** and **241** can be smoothly transformed to terminal acetylenes **242**, which, in combination with a specific alcohol component (allylic moiety), opens access to spirocyclic vinyldihydropyran derivatives **243** via intramolecular enyne methatesis (Scheme 71):

A similar approach was used by F. Shi et al. to construct bis-indolic systems **247** via MCR of diazo oxindoles **244**, alcohols **245**, and 2-indolyl carbinols **246** (Scheme 72) [80]. The latter generated allylic cations **I-32** under treatment with diphenylphosphoric acid and further reacted with in situ generated oxonium ylides (under Rh-catalysis) **I-33** via nucleophilic addition to provide bis-indolic compounds **245**. The authors discovered that this reaction is sensitive to substitution pattern at quaternary C_sp3_-atom of indolyl carbinol—two aryl groups were required for formation of stable cation and successful interaction with ylide. It was also shown that using of BINOL-derivatives as chiral acid allowed performing the reaction in enantioselective fashion, although with low ee (18%).

### 3.6. Generation of Ketene/Ketenimine

Diazo carbonyl compounds display different types of reactivity and are also well-known as ketene source via the Wolff rearrangement, which can be induced thermally, photochemically, or catalytically. Both terminal and disubstituted diazo compounds undergo this rearrangement. Ketenes are very reactive intermediates which are most often trapped by nucleophiles giving both cyclic or acyclic products.

**Scheme 73:**
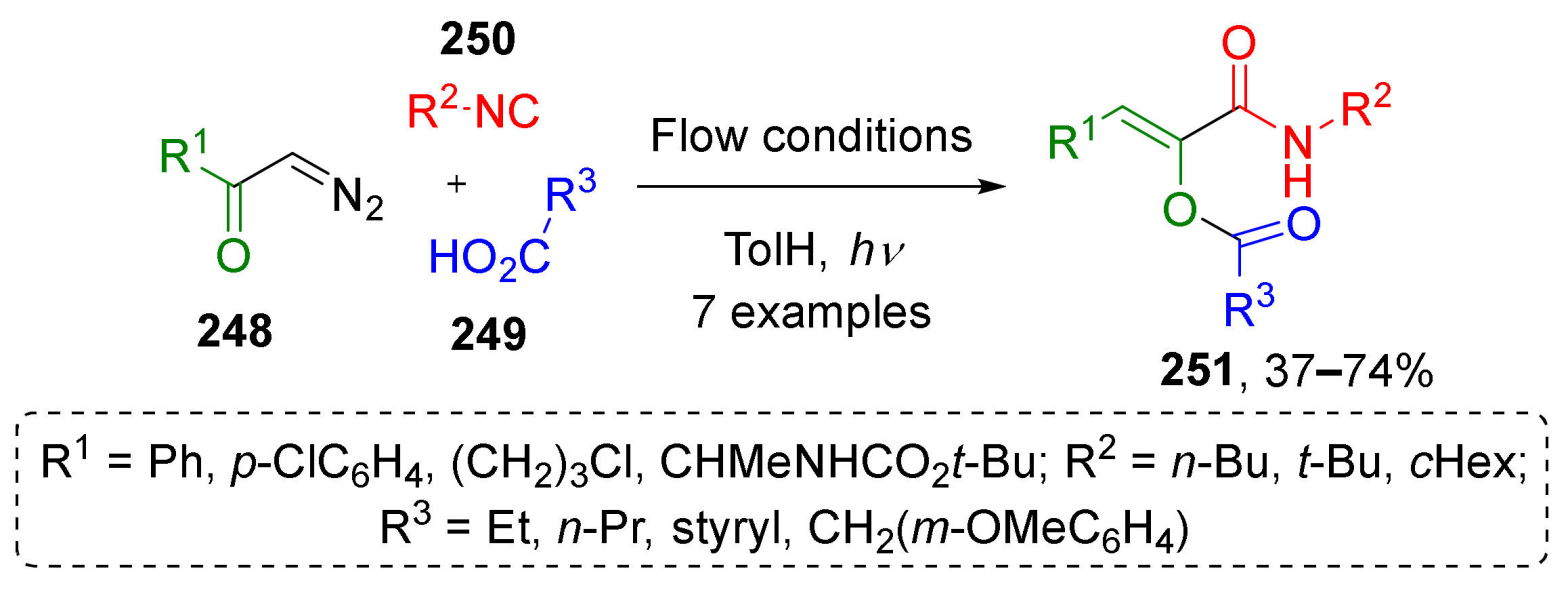
Photoinduced three-component reaction of diazo ketones, carboxylic acids, and isocyanides.

In one of the recent works, A. Basso et al. developed a protocol for introducing photochemically generated ketenes into formal Passerini reaction as carbonyl component (Scheme 73) [81]. Terminal aromatic and aliphatic diazo ketones **248** reacted with carboxylic acids **249** and alkyl isocyanides **250** to give polysubstituted α-acyloxyacrylamides **251** as *Z*-isomers. The reaction was performed in toluene under irradiation at 364 nm in a flow reactor making this approach attractive for sustainable chemistry. The obtained compounds were applied by authors to perform base-mediated rearrangements affording pyrrolones and pyrrolidinediones with antitumor activity.

**Scheme 74:**
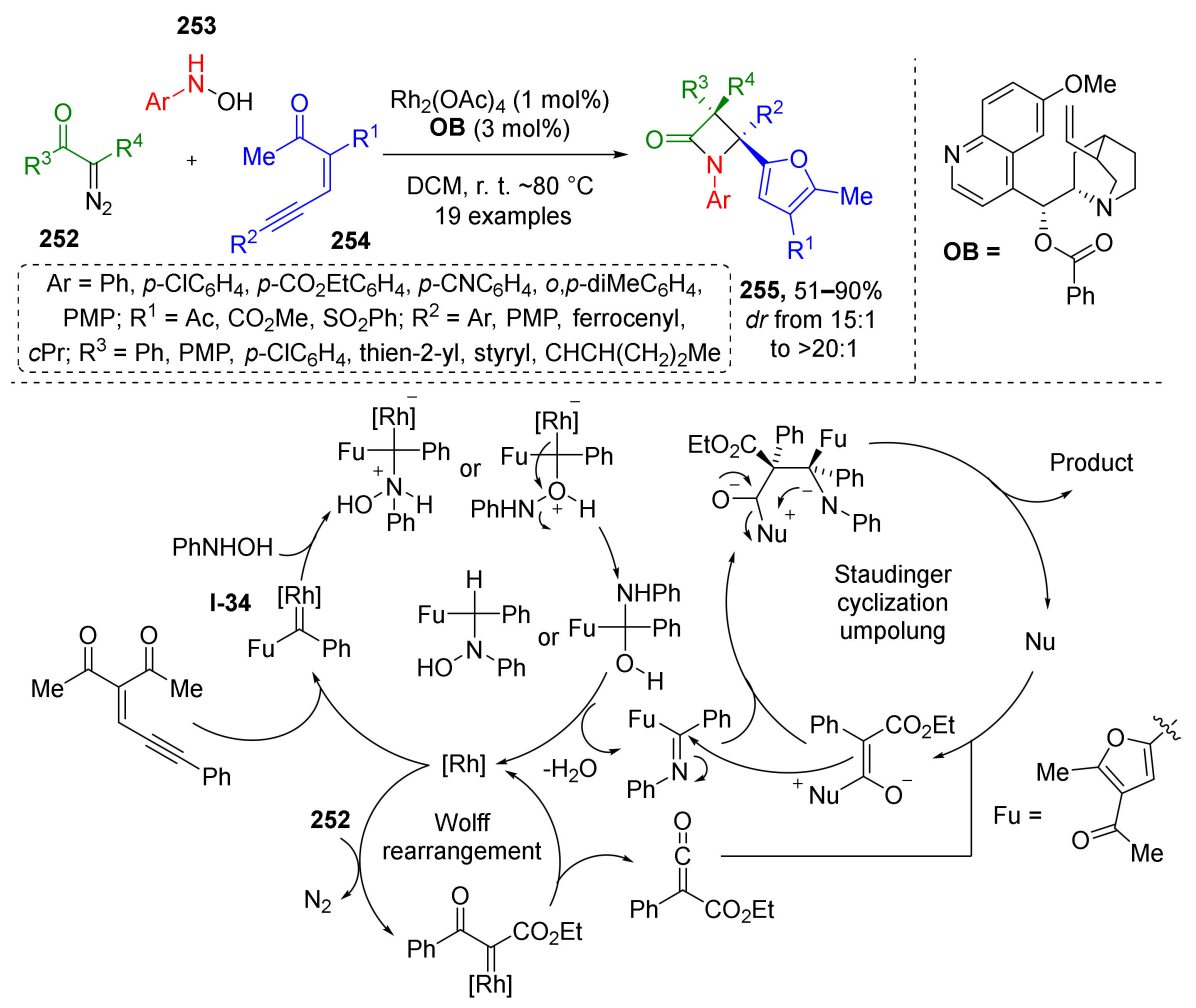
Stereoselective synthesis of fully substituted β-lactams via metal−organo relay catalysis.

Traditionally ketenes are viewed as useful building blocks for beta-lactam synthesis also known as the Staudinger synthesis. This methodology was recently improved by discovering a novel protocol for the preparation of complex fully substituted β-lactams **255** containing α-furyl moiety in position 4 of the ring (Scheme 74) [82]. In this case, the ketene was generated from diazo ketoesters **252** via Rh-catalyzed Wolff rearrangement while imine was formed through unusual reaction of aromatic hydroxylamines **253** and enynones **254**. Interestingly, generation of imine also proceeded under Rh-catalysis including formation of furane ring from acyclic starting materials. Mechanistic studies showed that enynone undergoes cyclization to furane Rh-carbenoid **I-34** first, which is then trapped by nucleophilic addition of hydroxylamine followed by water elimination. The obtained imine then enters diastereoselective Staudinger cyclization with ketene to produce β-lactam.

**Scheme 75:**
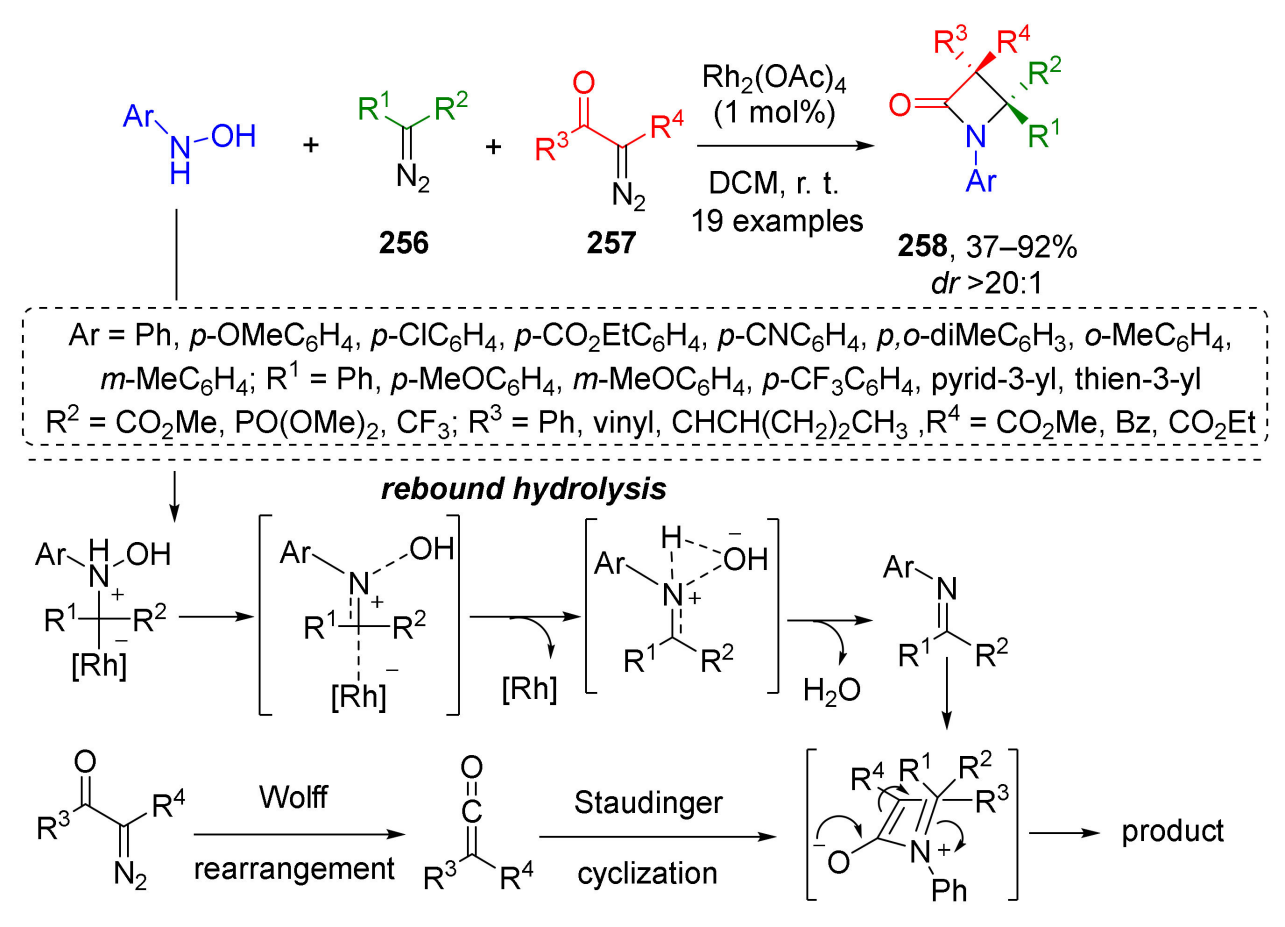
Rhodium-catalyzed C=N bond formation through a rebound hydrolysis and application in β-lactam synthesis.

The latter approach was limited to furyl substituents at position 4 of β-lactam, therefore in 2019 the same group (J. Sun and co-workers) developed its significantly more general modification allowing different aryl, alkyl, alkoxycarbonyl, phosphoryl, and trifluoromethyl groups instead (Scheme 75) [83]. The main difference was that imines were generated from hydroxylamines and diazo compounds **256**, not enynones via Rh-catalyzed reaction. In the next step, these imines entered a formal [2+2]-cycloaddition with ketenes generated from diazo carbonyl compounds **257** via Rh-catalyzed Wolff rearrangement. This MCR belongs to a rare type involving two different molecules of diazo compounds as reactants. Remarkably, in all cases, the reaction proceeded regioselectively although all components were mixed at once. The reaction proceeded also with excellent diastereoselectivity.

**Scheme 76:**
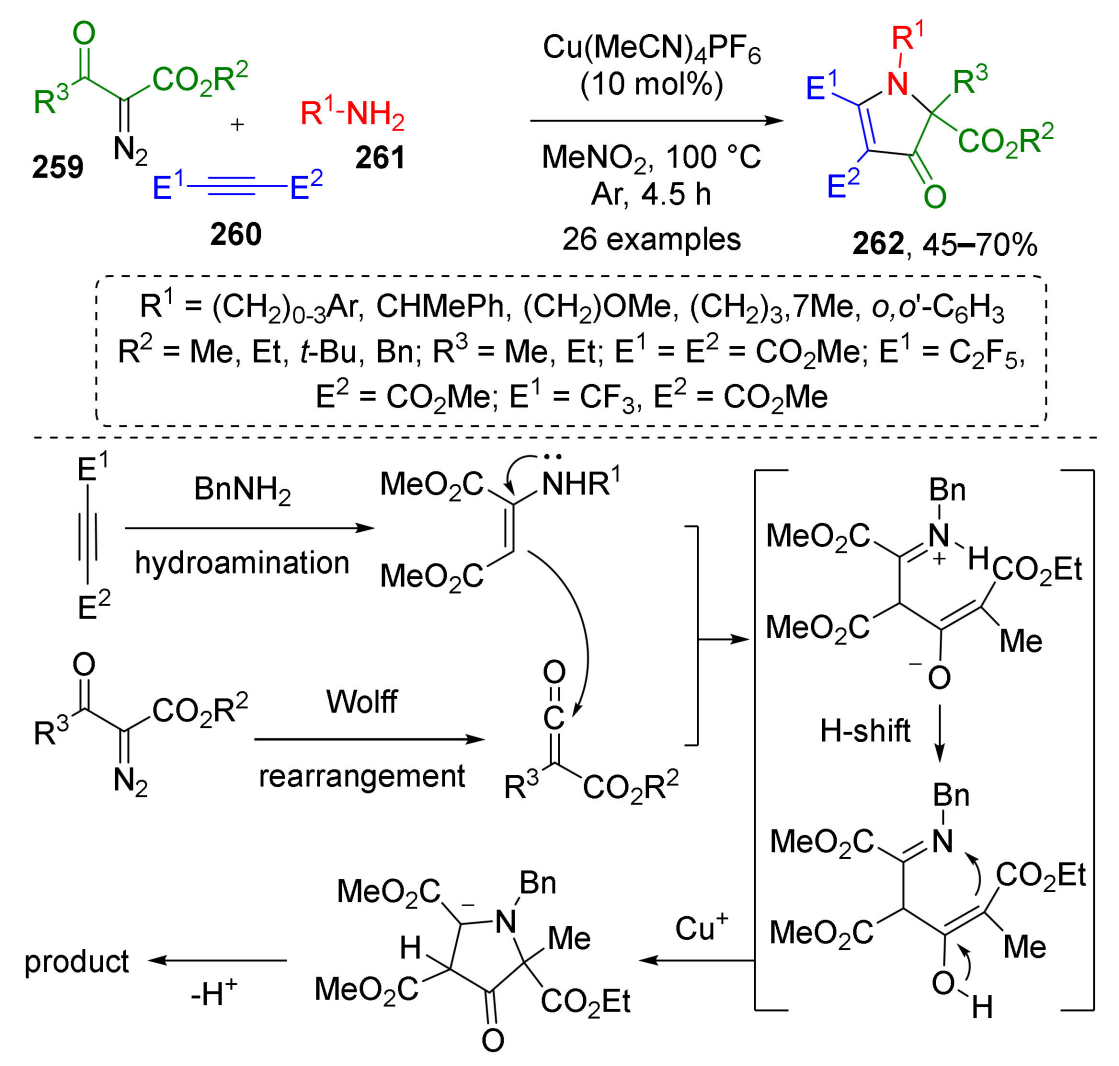
Copper-catalyzed three-component synthesis of polysubstituted β-pyrrolinones.

Ketenes can serve as building blocks for the construction of azoles as well. In 2021, N. Jiao et al. developed a novel regioselective approach towards fully substituted β-pyrrolinones **262** from diazo ketoesters **259**, electron poor acetylenes **260**, and primary amines **261** based on unusual reaction of *C*-nucleophilic addition to ketene (Scheme 76) [84]. This copper-catalyzed cascade included hydroamination of alkyne followed by nucleophilic attack of thus generated enamine to ketene obtained via the Cu-catalyzed Wolff rearrangement of diazo ketoester.

**Scheme 77:**
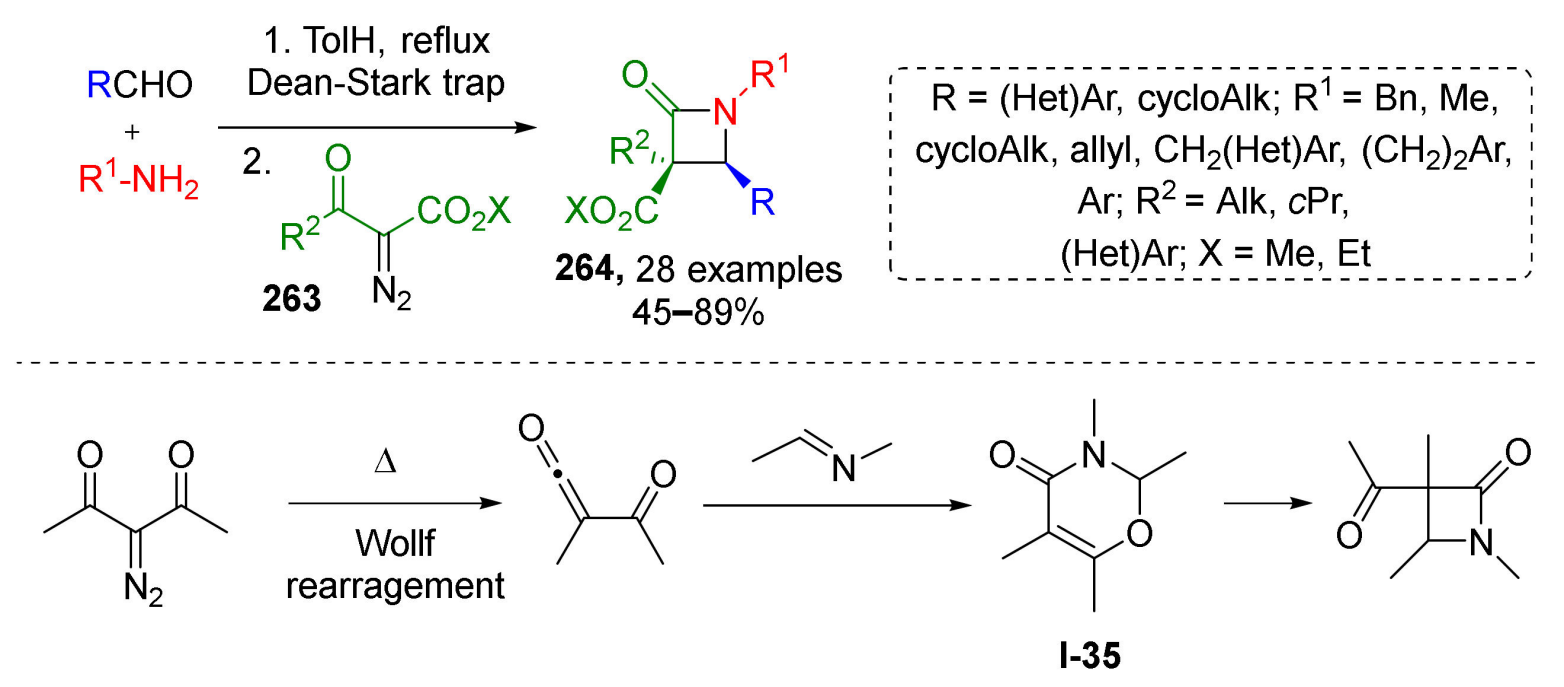
Thermally promoted reaction of α-acyl-α-diazoacetates with imines.

Economically efficient and environmental-friendly metal-free protocols are attracting more and more attention these days. In 2019, it was demonstrated that the thermal Wolff rearrangement involving diazo ketoesters **263** can be effectively coupled with imine generation from amine and carbonyl compound under azeotropic distillation conditions to prepare tetrasubstituted β-lactams **264** (Scheme 77) [85]. This novel protocol of Staudinger synthesis showed general scope and excellent diastereoselectivity. Various (hetero)aromatic and aliphatic groups were well tolerated in aldehyde and amine components as well as R^2^ substituent in diazo compound. According to DFT calculations the reaction is likely to proceed with interim formation of 1,3-oxazinone **I-35** as initial product which further undergoes recyclization to β-lactams.

**Scheme 78:**
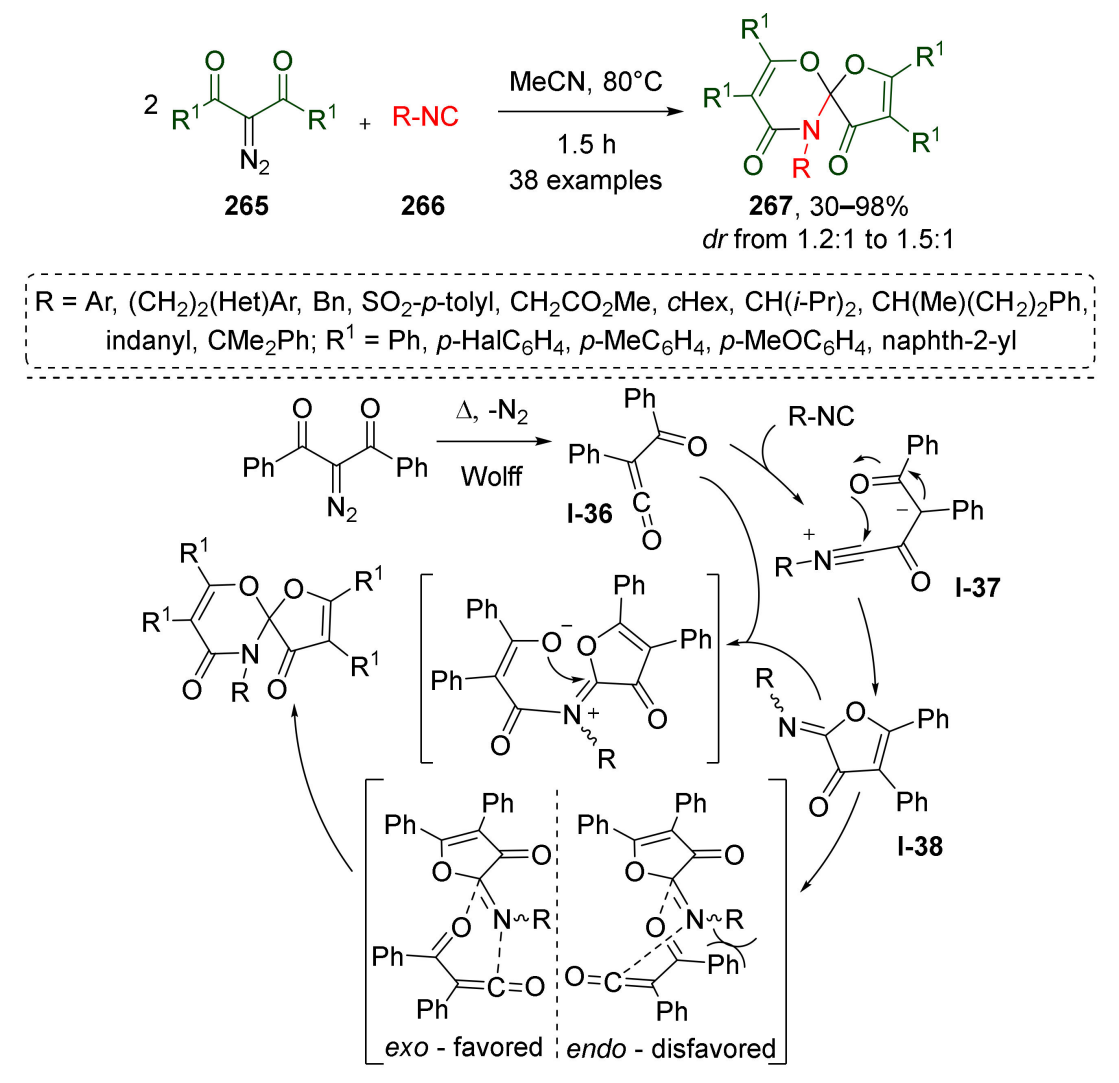
Catalyst-free formal [4+1]/[4+2] cyclization cascade sequence of isocyanides with two molecules of acylketene.

Another interesting application of metal-free thermally promoted Wolff-rearrangement was demonstrated by Y.-L. Liu et al. in 2019 (Scheme 78) [86]. In this study, two molecules of diazo 1,3-diketones **265** reacted with aliphatic or aromatic isocyanides **266** giving access to synthetically challenging spiroheterocyclic systems **267**. This unexpected reaction included generation of α-acylketene **I-36**, nucleophilic addition of isocyanide to C*_sp_* of ketene, cyclization of corresponding intermediate nitrilium ion **I-37** to iminofuranone **I-38**, and [4+2]-cycloaddition with the second molecule of ketene. It was demonstrated that this reaction tolerated various types of isocyanide but was very sensitive to the substituents in the diazo compound. Into this reaction, the authors successfully involved mono-, di-, and tri-α-substituted isocyanides with different functional groups, while only diazo compounds with two phenyl groups showed sufficient reactivity.

**Scheme 79:**
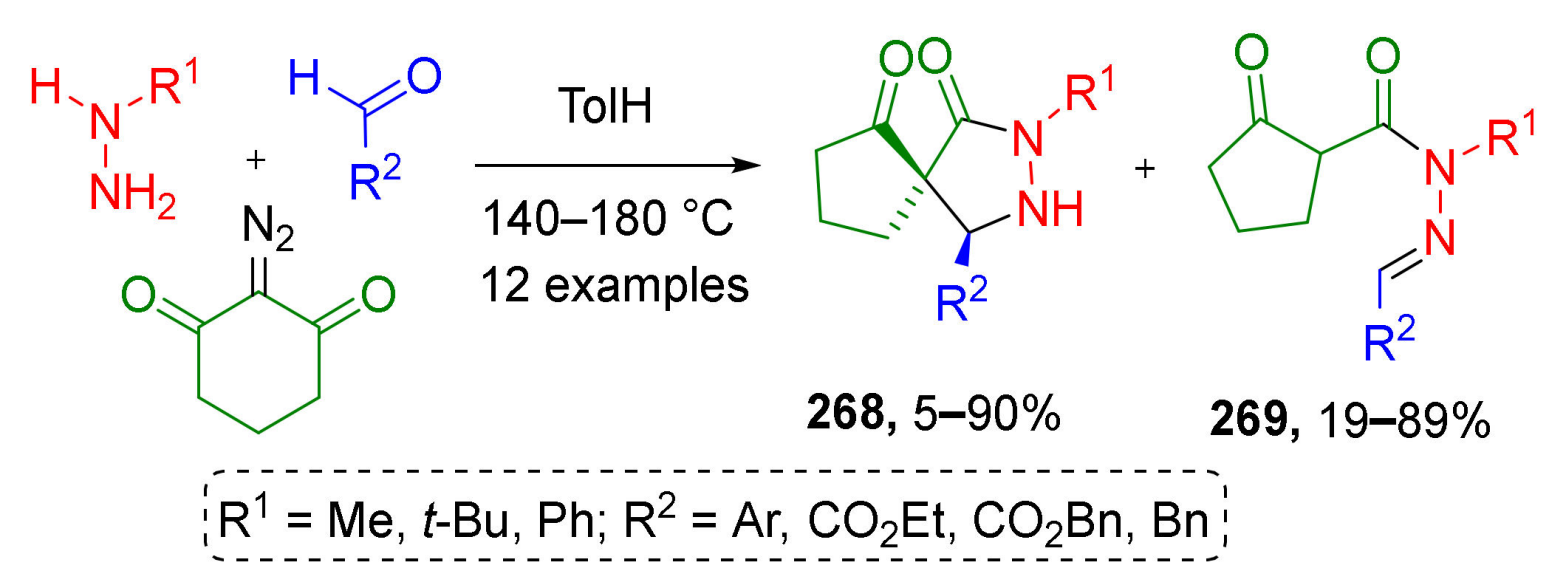
Three-component [3+2]-annulation of aldehydes, hydrazines, and α-oxoketenes.

Spirocyclic *N*-heterocycles can also easily be accessed from ketenes when cyclic diazo compounds are employed. In such cases, the Wolff rearrangement is accompanied by ring contraction. Y. Coquerel et al. applied this thermally induced (MW irradiation) transformation to 2-diazo-1,3-cyclohexanedione and successfully combined it with nucleophilic addition of in situ generated hydrazones and subsequent cyclization to prepare a series of spirocyclic pyrazolidinones **268** (Scheme 79) [87]. In the case of alkyl substituted hydrazines, simple acylhydrazones **269** were isolated as major products. Elevating reaction temperature to 200 °C facilitated the cyclization step and allowed increasing the yield of the target spirocyclic compounds.

**Scheme 80:**
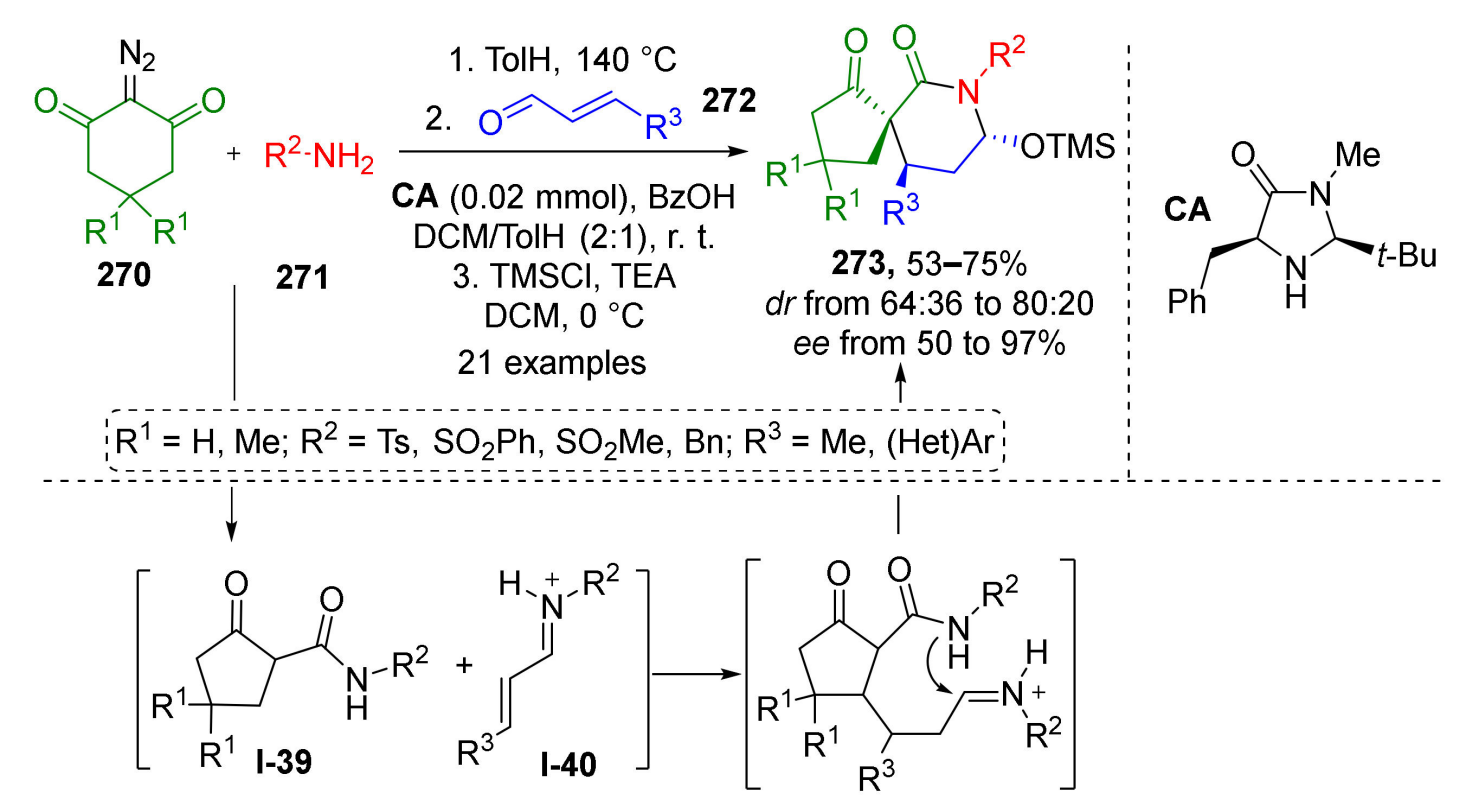
One-pot organocatalytic synthesis of spirocyclic piperidones.

In 2017, W. Huang and co-workers showed that using cinnamic imines in a similar strategy gave access to spirocyclic systems with six-membered piperidine moiety **273** (Scheme 80). The authors used diazo compounds **270**, sulfonamides **271**, and cinnamic aldehydes **272** as building blocks. Another sequence of adding reactants was applied, therefore this reaction followed a different mechanistic pathway. At first, ketene was generated thermally from diazo compound in the presence of sulfonamide which led to its acylation giving intermediate **I-39**. After the consumption of these reactants, cinnamic aldehyde and the catalyst, secondary amine, were added. Amine additive facilitated the next two steps, namely, conjugate addition and cyclization, through the formation of highly reactive iminium intermediate **I-40**. Applying chiral proline-derived amine **CA** as the catalyst allowed obtaining the desired spirocyclic products with high optical purities.

**Scheme 81:**
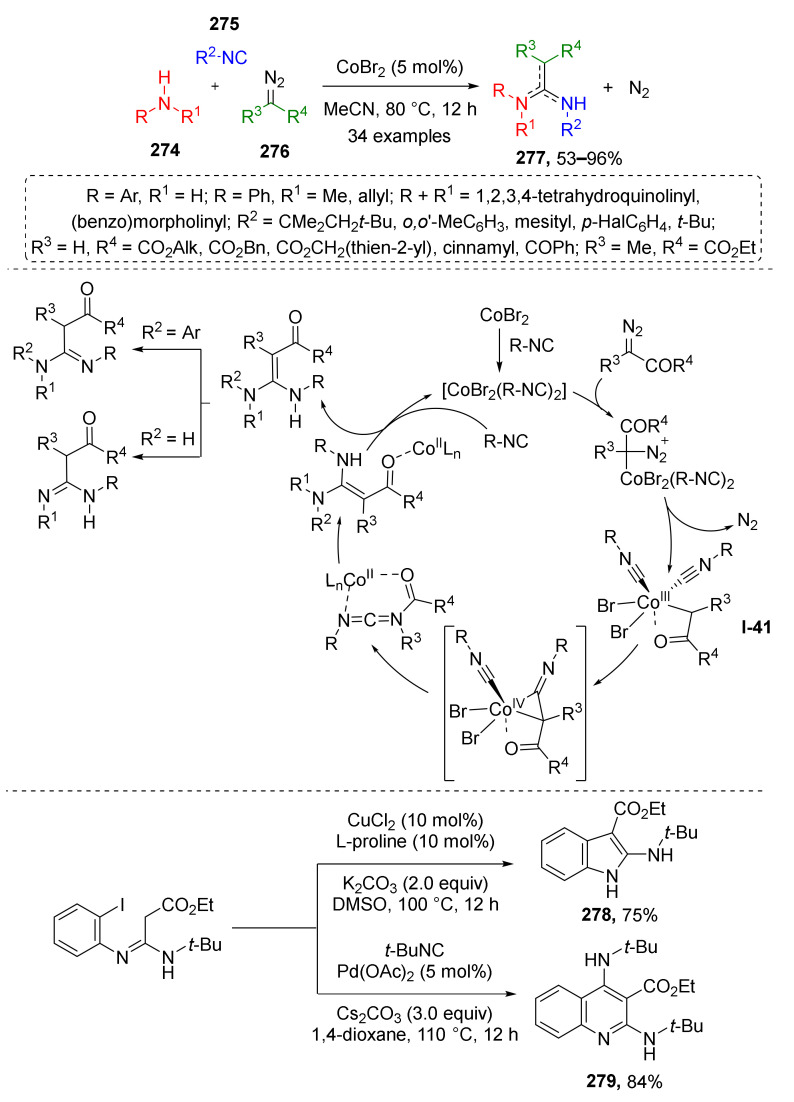
Synthesis of amidines via Co-carbene radical coupling with isocyanides and amines.

While ketenes are generally obtained via the Wolff rearrangement, formation of ketene imines requires reaction of metal carbenoid derived from diazo compound with isocyanide. In 2021, J.-B. Xia and co-workers reported a novel Co-catalyzed amidine synthesis from secondary amines **274**, aromatic or aliphatic isocyanides **275**, and diazo esters **276** (Scheme 81) [88]. The investigation of the reaction mechanism showed that a non-classical Co(III)-carbenoid **I-41** is formed from catalyst CoBr_2_, diazo compound, and two molecules of isocyanide. Next, one molecule of isocyanide migrates to the carbenoid carbon atom, forming a ketenimine ligand, while the second isocyanide molecule leaves coordination sphere via reductive elimination giving a Co(II) species. Coordinated ketenimine then undergoes nucleophilic addition of amine forming a coordinated molecule of amidine **277**. The latter is released from the complex via ligand exchange with another two isocyanide molecules. It was also demonstrated that the obtained products can be useful building blocks for the preparation of indoles **278** and quinolines **279**.

### 3.7. Generation of Organic Radical Species

It is a general trend that in organic synthesis, most approaches are based on ionic or concerted reactions, while radical processes are quite rare, especially in the development of MCRs. Many recent studies in this field involve reactions of hypervalent iodine compounds.

**Scheme 82:**
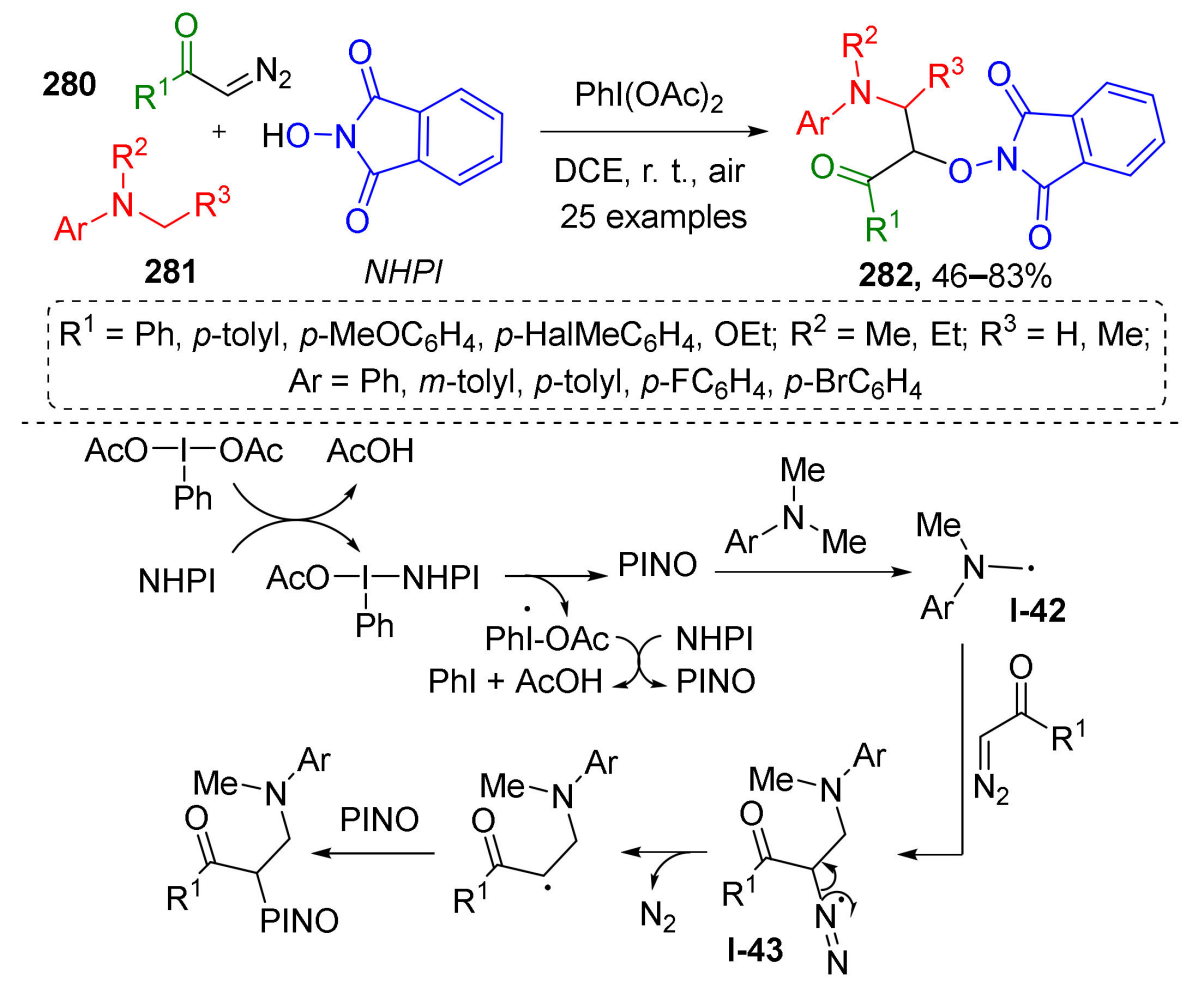
Oxidative carbooxygenation of α-diazo carbonyls via radical dediazotization.

One such novel approach was developed by B. Jiang et al. in 2016, and is aimed at preparation of α-aminooxy-β-amino ketones **282** from terminal diazo etones **280**, *N*-methylanilines **281** and *N*-hydroxyphthalimide in the presence of PIDA as radical initiator (Scheme 82) [89]. This reaction involves the formation of radical from *N*-hydroxyphthalimide (PINO) as the key step. This reactive intermediate then interacts with *N*-methyl aniline via H-abstraction which generates second type of radical species **I-42**. The latter reacts with diazo compound forming intermediate **I-43**, which undergoes radical elimination of dinitrogen. The product of this step is further trapped by *N*-hydroxyphthalimide giving stable α-aminooxy-β-amino ketone **282**. It should be noted, that this is an example of metal-free C(sp_3_)−H functionalization, a very attractive type of MCR.

**Scheme 83:**
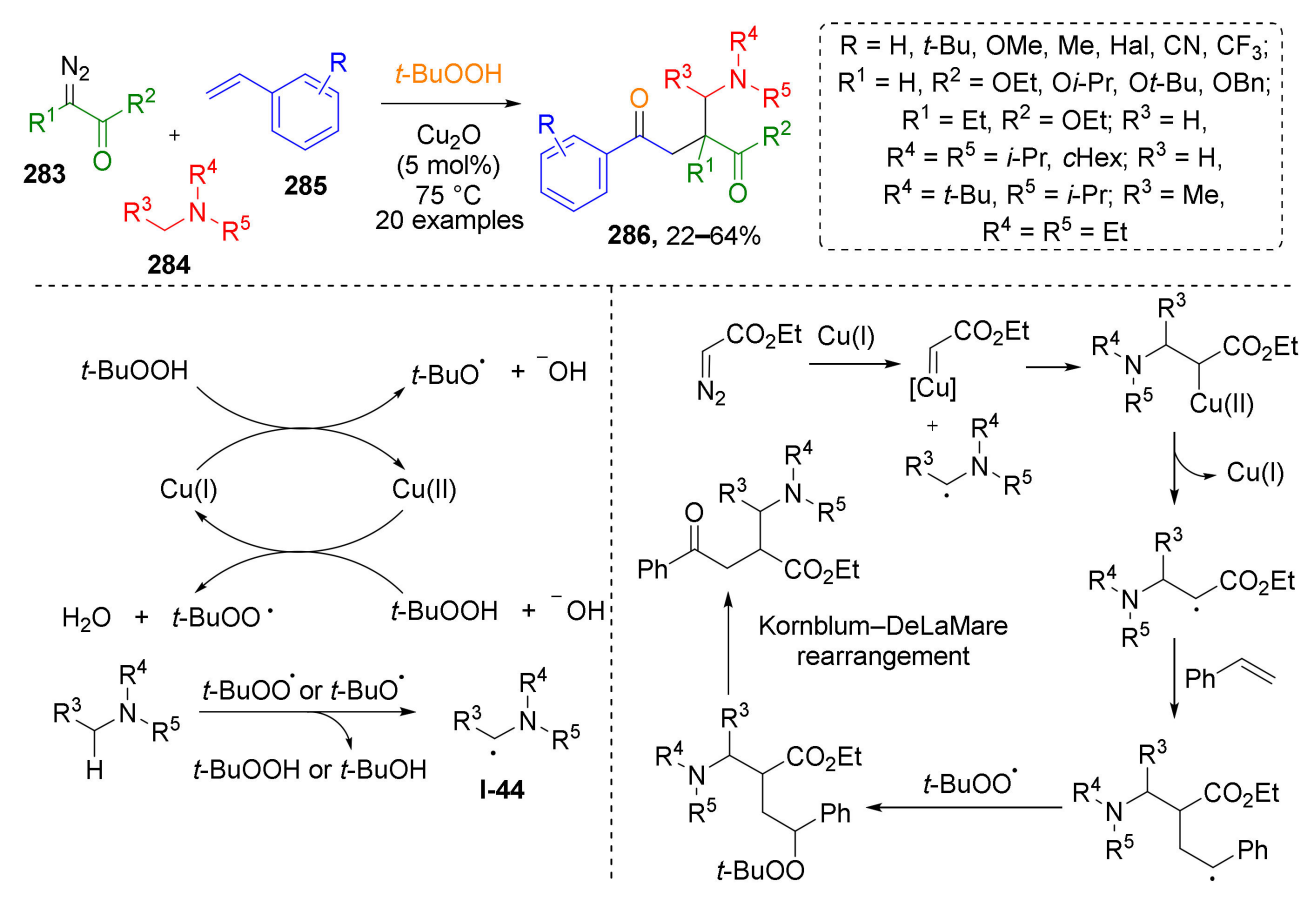
Cu-catalyzed four-component cascade reaction of styrenes, diazo compounds, amines, and *tert*-butyl hydroperoxide.

Several metal-catalyzed radical MCRs involving diazo compounds have also been developed recently. It was demonstrated that terminal diazo esters **283** can be functionalized by tertiary aliphatic amines **284** and styrenes **285** under treatment with TBHP in the presence of Cu_2_O (Scheme 83) [90]. In this case, initiator, a *t*-BuO-radical, was generated from TBHP and Cu_2_O followed by radical transfer to amine molecule producing intermediate **I-44**. This radical further attacks Cu-carbenoid obtained from diazo compound and copper catalyst. The target product β-alkoxycarbonyl-γ-amino ketone **286** is formed after sequential addition of styrene and t-BuO-radical to the abovementioned intermediate and Kornblum–DeLaMare rearrangement.

**Scheme 84:**
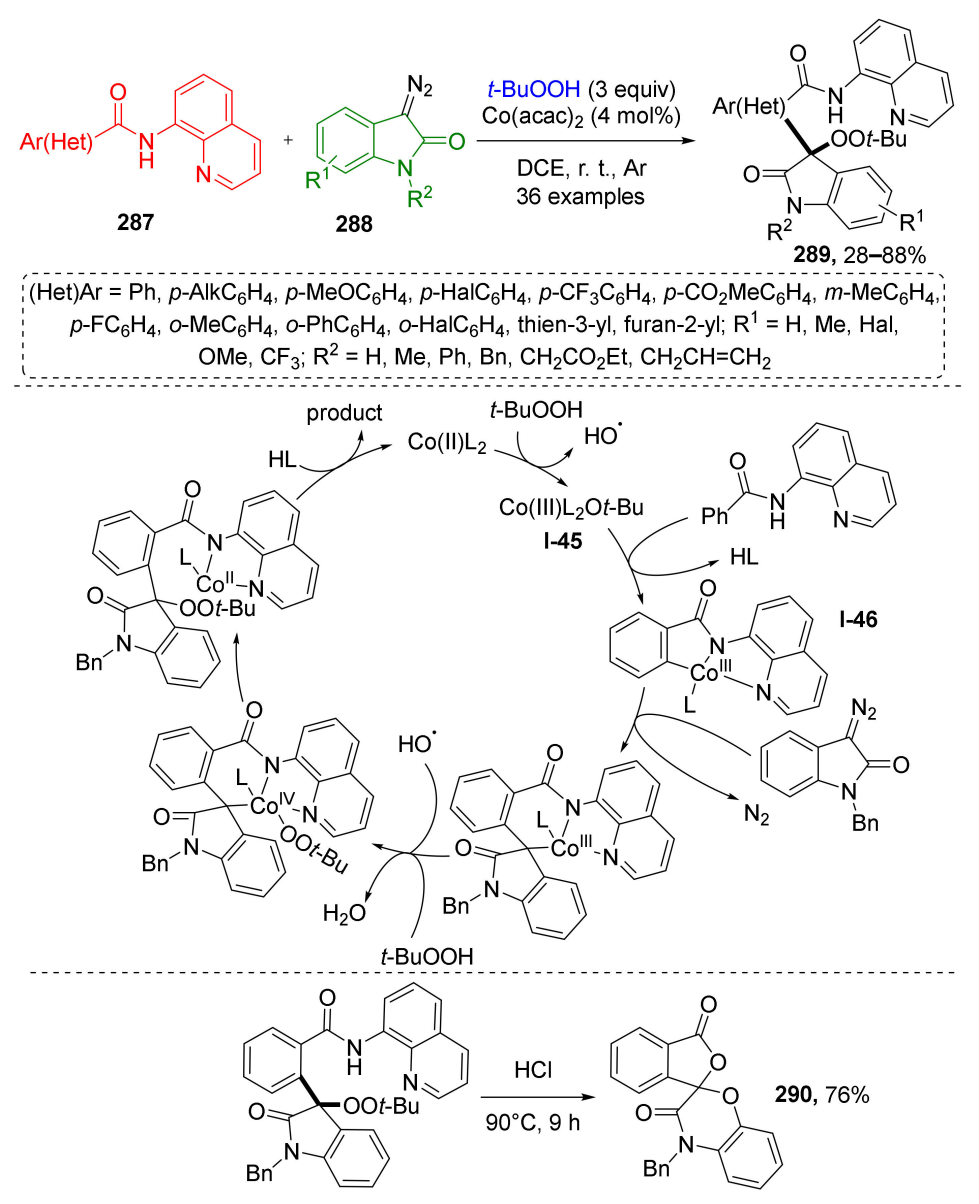
Three-component coupling of benzamides, diazo compounds, and *tert*-butyl hydroperoxide.

The methodology of radical MCRs has been extended to diazo oxindoles as well. M.-P. Song et al., in 2021, reported a novel Co-catalyzed method for the functionalization of diazo compounds **287** with TBHP and aromatic amides **288** (Scheme 84) [91]. Interestingly, in this case TBHP acted as a building block and not only as radical initiator. Corresponding reaction products, 3,3-disubstituted indolin-2-ones **289** were formed via a multi-step process including dissociation of TBHP, oxidative addition of *t*-BuO-radical to catalyst Co(acac)_2_ with the formation of Co(III)-species **I-45**. The latter reacted with the aromatic amide, which bears an extra chelating quinoline moiety giving an intermediate **I-46** via CH-activation. The next step was ligand exchange and denitrogenative migratory insertion reaction with diazo compound with formation of a C-C-bond between indoline and amide building blocks. This cascade ended with addition of the peroxide radical (*t*-BuOO) to the Co-center followed by transfer of the t-BuOO moiety from metal to indoline ring and cleavage of product **289** from the complex. The authors also demonstrated that the obtained products can be used for the preparation of spirocyclic lactones **290** via acid-promoted cyclization and elimination of t-BuOO and chelating aminoquinoline group.

**Scheme 85:**
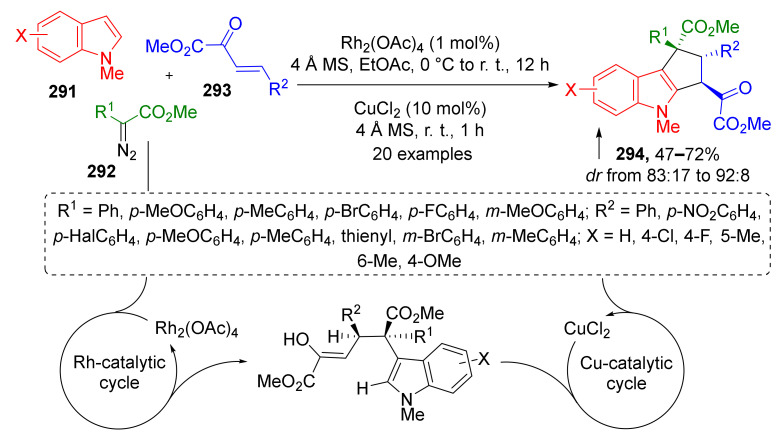
Double C-H functionalization of indoles via three-component reactions/CuCl_2_-catalyzed aerobic dehydrogenative coupling.

A remarkable example of radical MCR was reported by W. Hu in 2016. The authors discovered a double CH-activation/annulation reaction of substituted *N*-methylindoles **291** providing complex cyclopenta[*b*]indoles **294** in a regio- and diastereoselective fashion (Scheme 85) [92]. Indole functionalization was achieved by using diazo esters **292** and 2-oxobutenoates **293** under dual Rh/Cu catalysis.

**Scheme 86:**
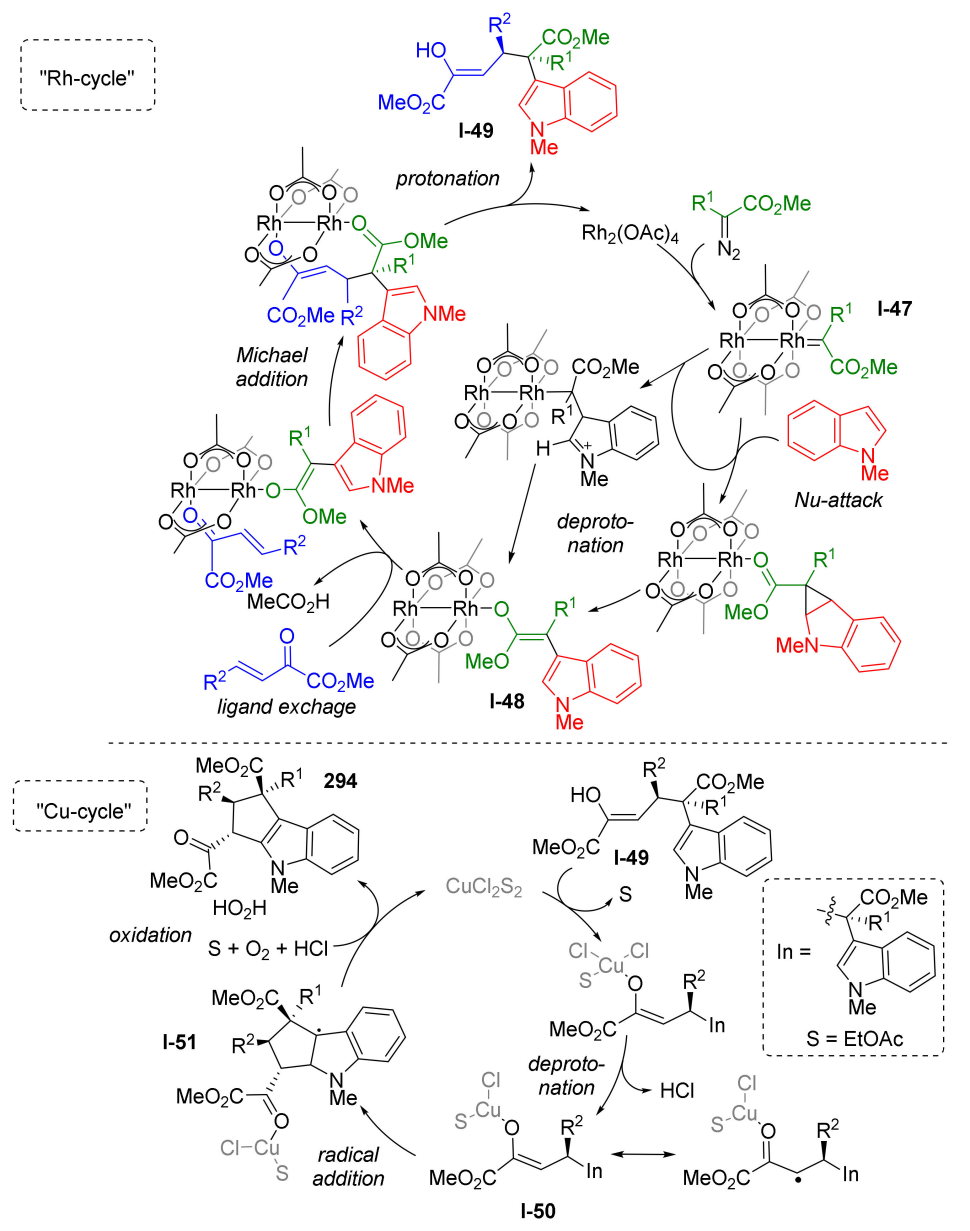
Mechanistic insights into the rhodium/copper cascade catalyzed dual C-H annulation of indoles.

The reaction mechanism was investigated by DFT calculations by Y. Lan and co-workers in 2020 (Scheme 86) [93]. They identified two separate catalytic cycles, one for each CH-activation step. During the first one, “Rh-cycle”, a metal carbenoid **I-47** was generated from the diazo compound followed by nucleophilic addition of indole leading to formation of the enolic intermediate **I-48**. The latter entered 1,4-conjugate addition to the third reactant—2-oxobutenoate, giving non-annulated half-product **I-49** with quaternary asymmetric carbon atom. Therefore, the first cycle enabled CH-activation in position 3 of indole. The second catalytic cycle, “Cu-cycle”, was employed to achieve annulation via CH-activation in position 2 of indole. The key step of this cycle was radical addition of Cu-coordinated side chain of half-product **I-50** to indole five-membered ring leading to its dearomatization and intermediate **I-51**. The aromaticity of indole was restored at the last step of the cycle by aerobic oxidation.

### 3.8. Generation of Dearomatized Species

Electron rich aromatic systems such as anilines and indoles can be involved in specific transformations occurring via formation of dearomatized zwitter-ionic intermediates after addition of electrophilic species. The latter are often Rh-carbenoids generated from diazo compounds in situ. These zwitter-ionic intermediates are further trapped by other electrophilic species such as proton or carbonyl groups followed by rearomatization. Such transformations of electron rich aromatics are often regarded as Friedel–Crafts-type alkylations. This approach has been widely applied for functionalization of 3-diazooxindoles.

**Scheme 87:**
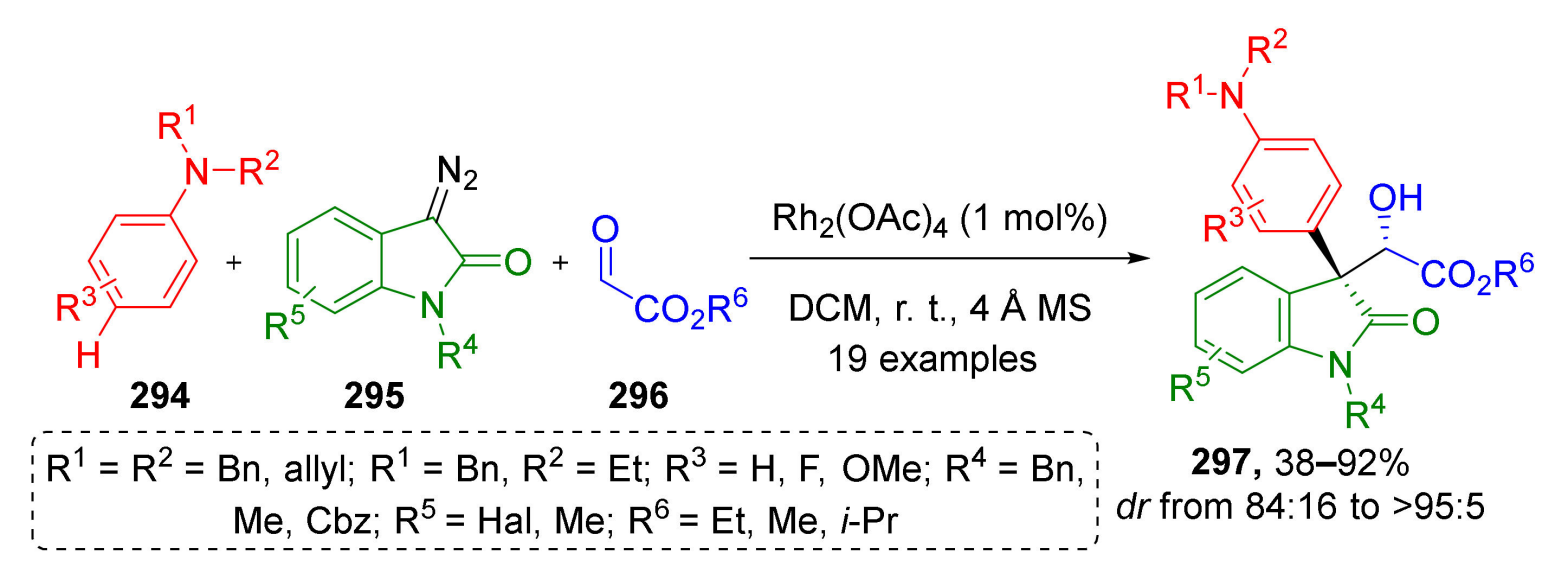
A Rh(II)-catalyzed three-component reaction of 3-diazooxindoles with *N*,*N*-disubstituted anilines and glyoxylates.

In 2016, W.-H. Hu and co-workers reported a Rh-catalyzed three-component reaction of diazo compounds **294**, *N*,*N*-dialkylanilines **295**, and α-ketoesters **296**, which provided indolin-2-ones **297** with quaternary carbon atom and α-hydroxy carboxylic acid moiety at position 3 (Scheme 87) [94]. The reaction products **297** were obtained in high yields and diastereoselectivity.

**Scheme 88:**
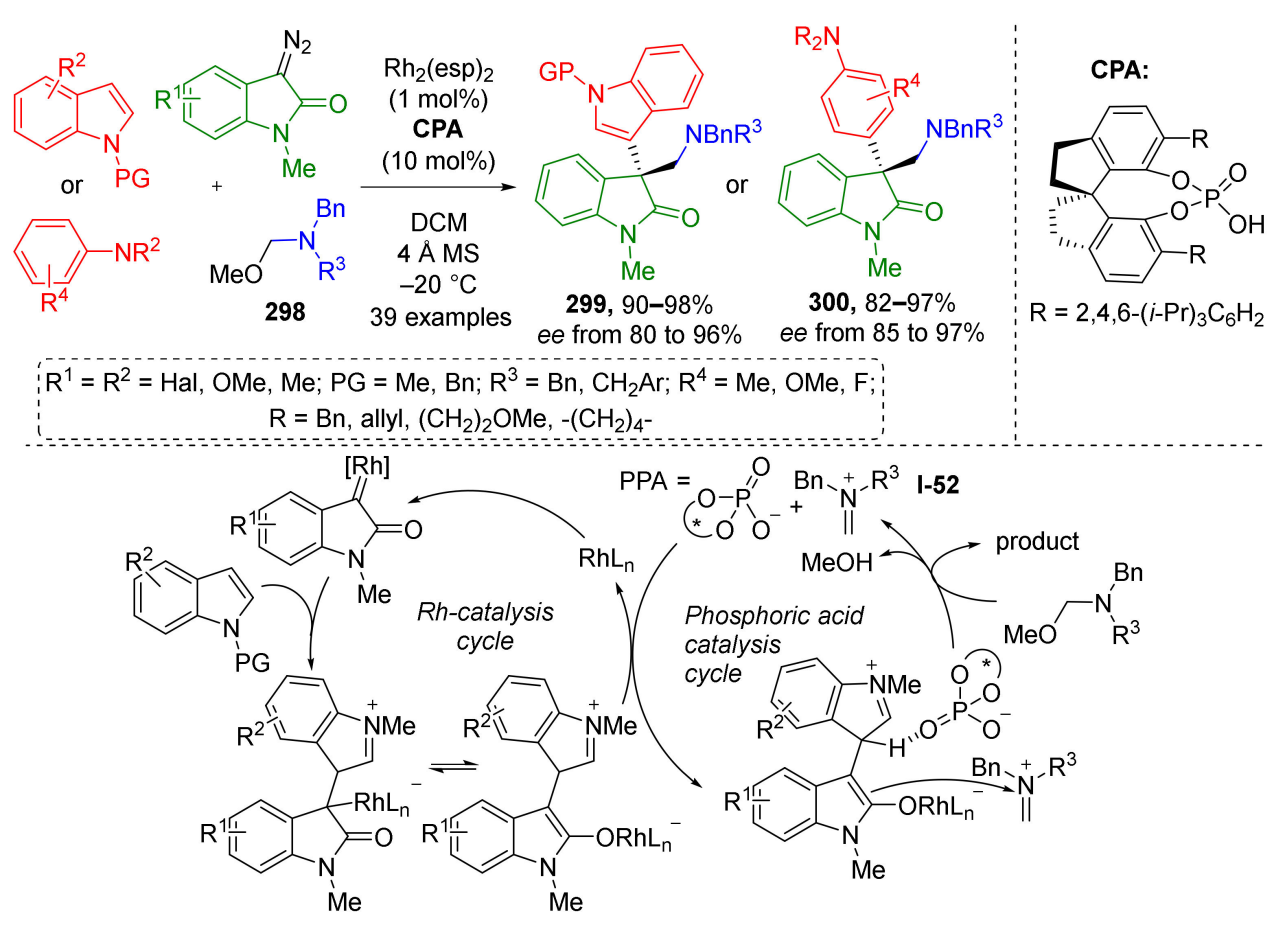
Three-component asymmetric aminomethylation involving diazo oxindoles.

Further exploration of the potential of this type of MCR concerns involving other electrophilic reagents in the last step. For example, in 2019 the same group showed that using iminium species **I-52** as trapping agent afforded aminomethylated indoline-2-ones **299** or **300** via Mannich addition (Scheme 88) [95]. Iminium reagent was generated in situ from common stable precursor—methoxymethyl amine **298**. Several other substantial improvements have also been made by the authors—identifying suitable chiral additive (CPA, a BINOL-derived phosphoric acid) for performing the reaction enantioselectively and expanding the scope of the electron rich aromatic component to indoles in addition to anilines.

**Scheme 89:**
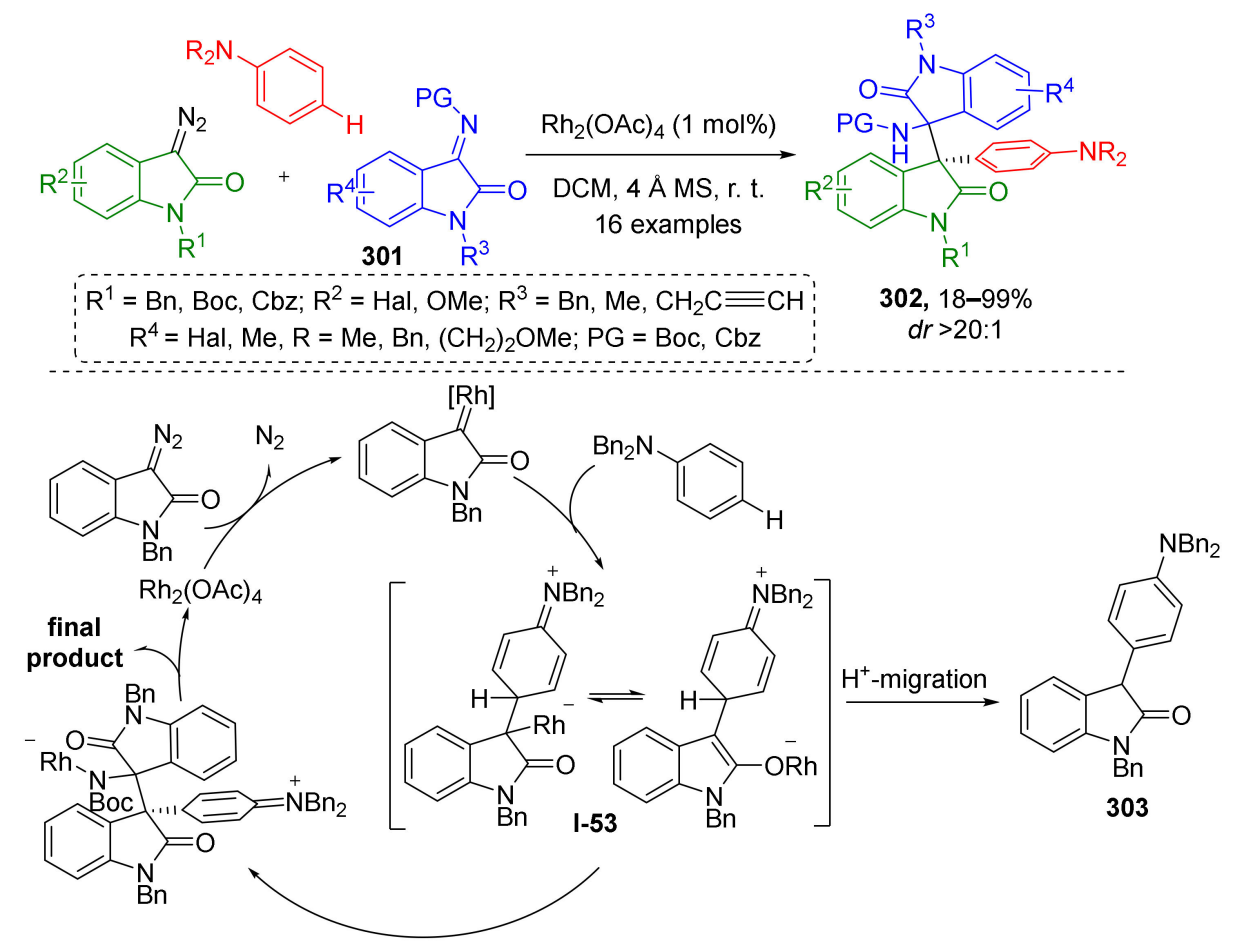
Rh(II)-catalyzed three-component reaction of *N*,*N*-disubstituted anilines, 3-diazooxindoles, and isatin ketimines.

Ketimines were also found to be suitable substrates for this multicomponent Fridel–Crafts/Mannich addition approach. In 2019, L. Shunying and co-workers reported a construction of complex bis-indolonic systems **302** with two newly formed quaternary carbon asymmetric centers from isatin 3-(*N*-Boc)ketimines **301** (Scheme 89) [96]. The authors also managed to isolate side-product **303** arising from undesired intramolecular 1,2-proton transfer within zwitter-ionic intermediate **I-53**.

**Scheme 90:**
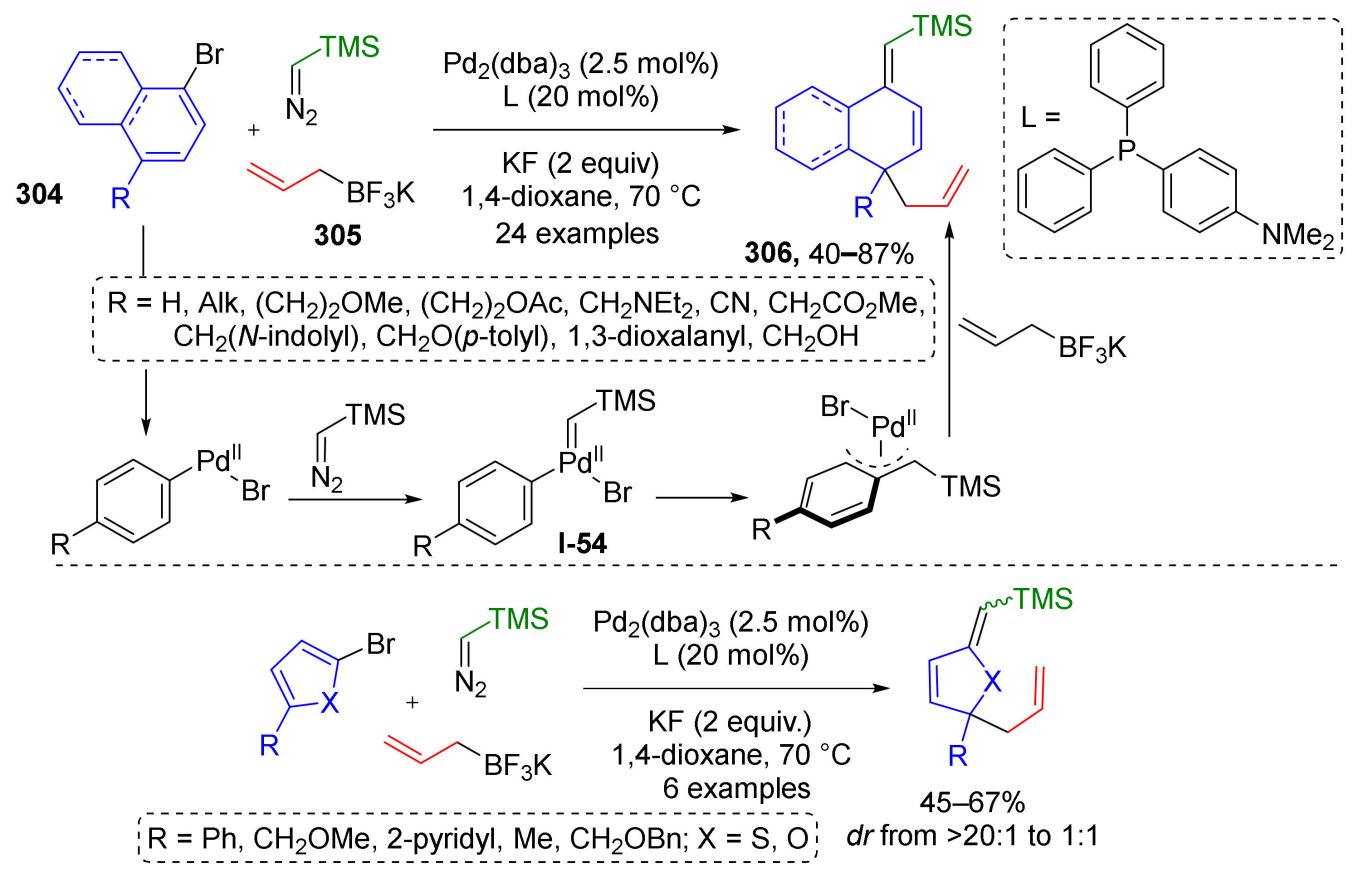
Pd-catalyzed dearomative three-component reaction of bromoarenes with diazo compounds and allylfluoroborates.

This concept of functionalization through dearomatization can be expanded to electronically unbiased substrates such as haloarenes by using palladium catalysis. In 2019, J. Yamaguchi and co-workers reported such MCRs based on generation of palladium-π-benzyl complexes from 4-substituted bromoarenes **304**, TMS-diazomethane, and Pd^0^-catalyst, namely, Pd_2_(dba)_3_ (Scheme 90) [97]. This π-complex was further trapped by nucleophilic allyl borate **305** giving stable dearomatized 3,6-disubstituted 1,4-cyclohexadienes **306**. Mechanistic studies showed that palladium-π-benzyl complex is generated from Pd-carbenoid **I-54** via migratory insertion reaction. The scope of bromoarene component included benzenes, naphthalines, furans, and thiophenes. The reaction also tolerated a wide range of substituents in bromoarene, such as dialkylamine, alkoxy, acetoxy, alkoxycarbonyl, allyl, and heteroaryl providing a general method for preparation of functionalized 1,4-cyclohexadienes.

**Scheme 91:**
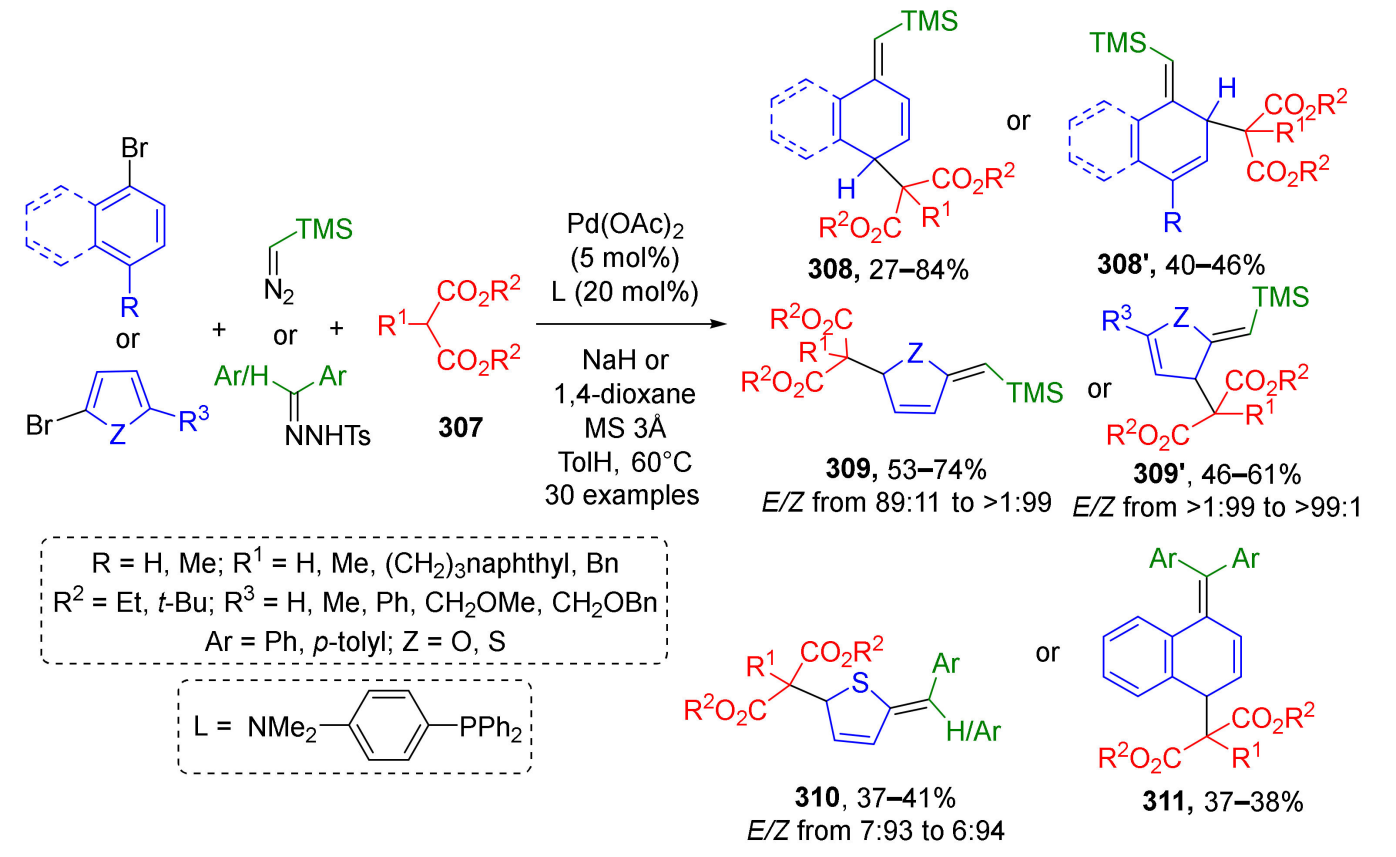
Catalytic three-component C-C bond forming dearomatization of bromoarenes with malonates and diazo compounds.

Not only allyl borates can be employed as nucleophilic reagents in this MCR, which was further demonstrated by the same group. CH-acidic compounds such as malonates **307** can serve as trapping agent for the abovementioned palladium-π-benzyl complexes (Scheme 91) [98]. Depending on reaction conditions, the authors isolated compounds **308–311** or their regioisomers **308′/309′**. To avoid oxidation to 1,4-quinoid structures, the authors used C-monoalkylated malonates. In this work, the scope of diazo component was also expanded to mono or diaryl substituted ones.

## 4. MCRs Where Reaction of Diazo Compound and One of the Components Generates a New Stable Compound, Which Further Reacts with the Third Component of the MCR

The Hooz reaction is based on coupling of organoboranes, diazocarbonyl compounds, and electrophiles. It has already proven itself a useful tool for the synthesis of α,α-bifunctionalized carbonyl compounds.

**Scheme 92:**
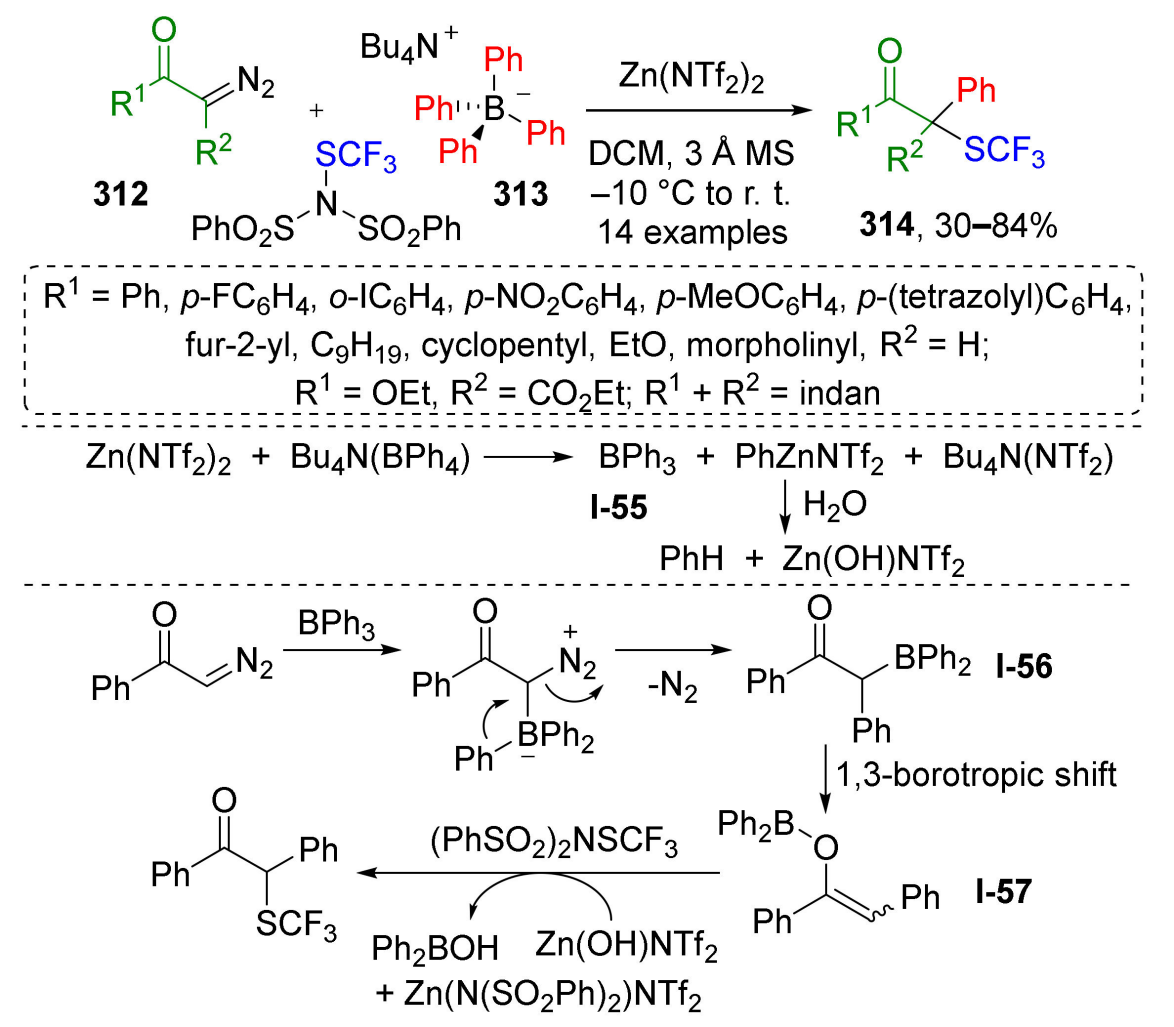
Trifluoromethylthiolation—arylation of diazocarbonyl compounds by modified Hooz multicomponent coupling.

Recently, the scope of nucleophilic component for this MCR was extended with SCF_3_ group by K.J. Szabo and co-workers (Scheme 92) [99]. The authors successfully involved aliphatic and aromatic terminal diazo ketones **312**, tetraaryl borates **313**, and (PhSO_2_)_2_NSCF_3_ into a coupling reaction catalyzed by Zn-bistriflimide affording α-aryl,α-SCF_3_-ketones **314**. One of the key steps of this transformation was generation of triarylborane **I-55** by zinc-boron transmetallation. Triarylborane **I-55** further attacked α-carbon atom of diazo compound via denitrogenative nucleophilic addition forming intermediate borane **I-56**, which further underwent 1,3-borotropic shift to vinyloxy-boronate **I-57** detected by NMR. Subsequent addition of (PhSO_2_)_2_NSCF_3_ and elimination of Ph_2_BOH gave the final product **314**.

**Scheme 93:**
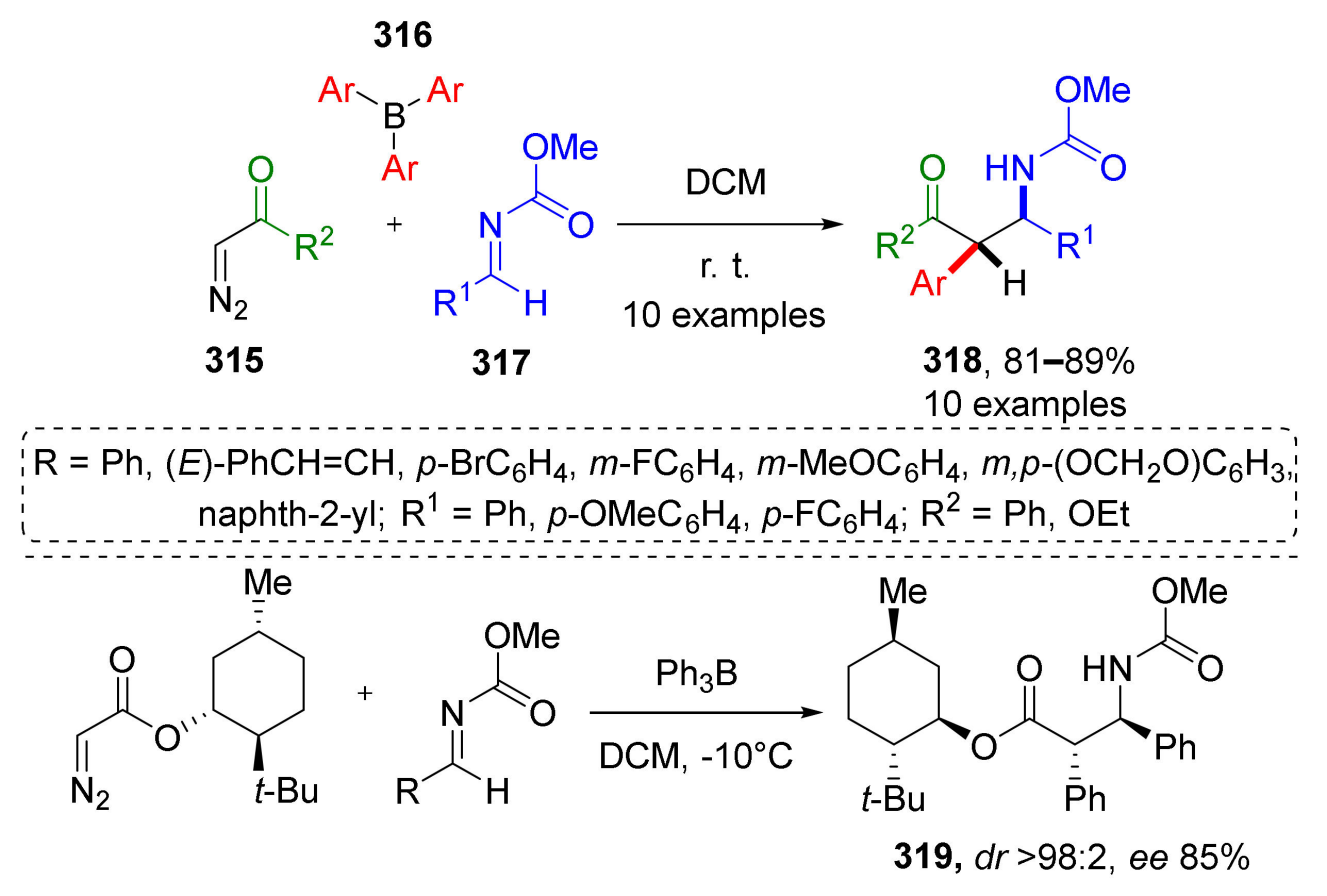
Diastereoselective three-component synthesis of β-amino carbonyl compounds using diazo compounds, boranes, and acyl imines.

G. Wang et al. published another variant of Hooze-type reaction of terminal diazo ketones or esters **315**, pre-synthesized triarylboranes **316**, and *N*-acylialdmines **317** as electrophilic component (Scheme 93) [100]. The reaction proceeded under mild conditions (at room temperature) without any addition of catalyst providing α,β-diaryl-β-acylaminocarbonyl compounds **318** as single diastereomers. The authors also demonstrated the possibility of enantioselective preparation of products **319** by introducing chiral auxiliary (menthol) to the diazo compound molecule.

**Scheme 94:**
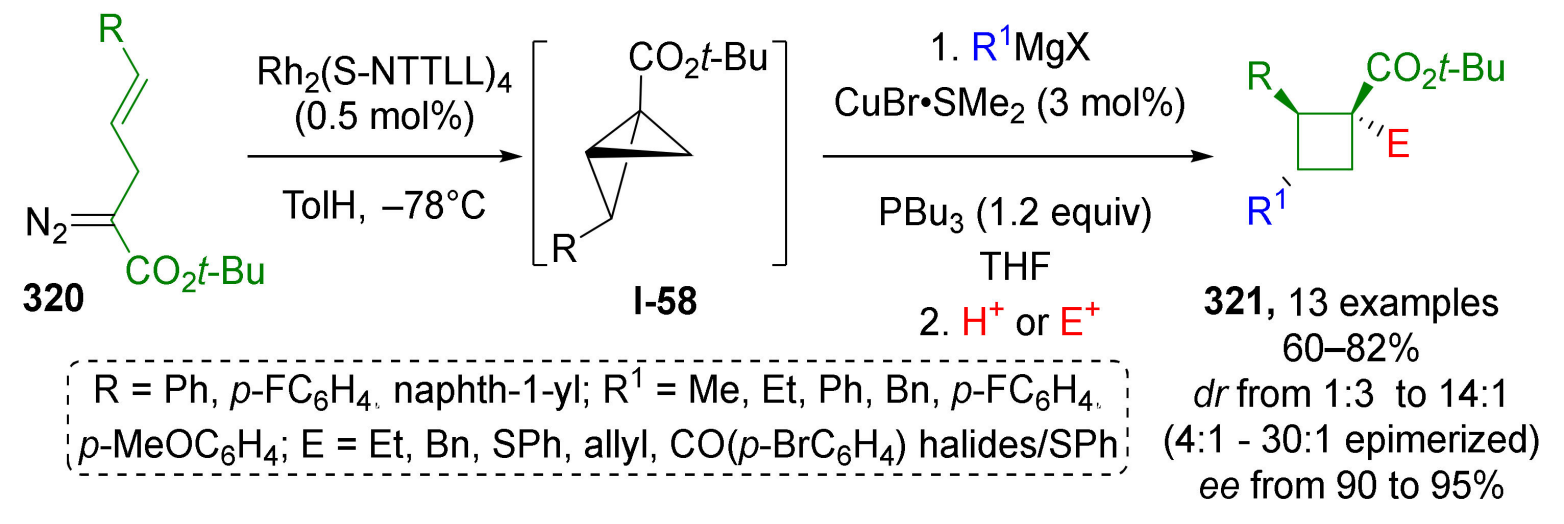
Synthesis of cyclobutanes via sequential Rh-catalyzed bicyclobutanation/Cu-catalyzed homoconjugate addition.

A rare example of carbocycle construction through a diazo-MCR was reported by J. M. Fox et al. (Scheme 94) [101]. Furthermore, 2-diazo-5-arylpent-4-enoate **320** reacted with alkyl or aryl organomagnesium compounds and electrophilic reagents under Rh-catalysis to provide tetrasubstituted cyclobutanes **321**. Brǿnsted acids, alkyl, or acyl halogenides were used as electrophiles. Applying chiral Rh-complex allowed performing synthesis with full stereocontrol and achieving high optical purity of products. Mechanistic studies showed that the first step of this MCR is Rh-catalyzed formation of bicyclobutanes **I-58**, which can be isolated. Bicyclobutanes were transformed into target products via nucleophilic ring-opening reaction with Grignard reagents.

**Scheme 95:**
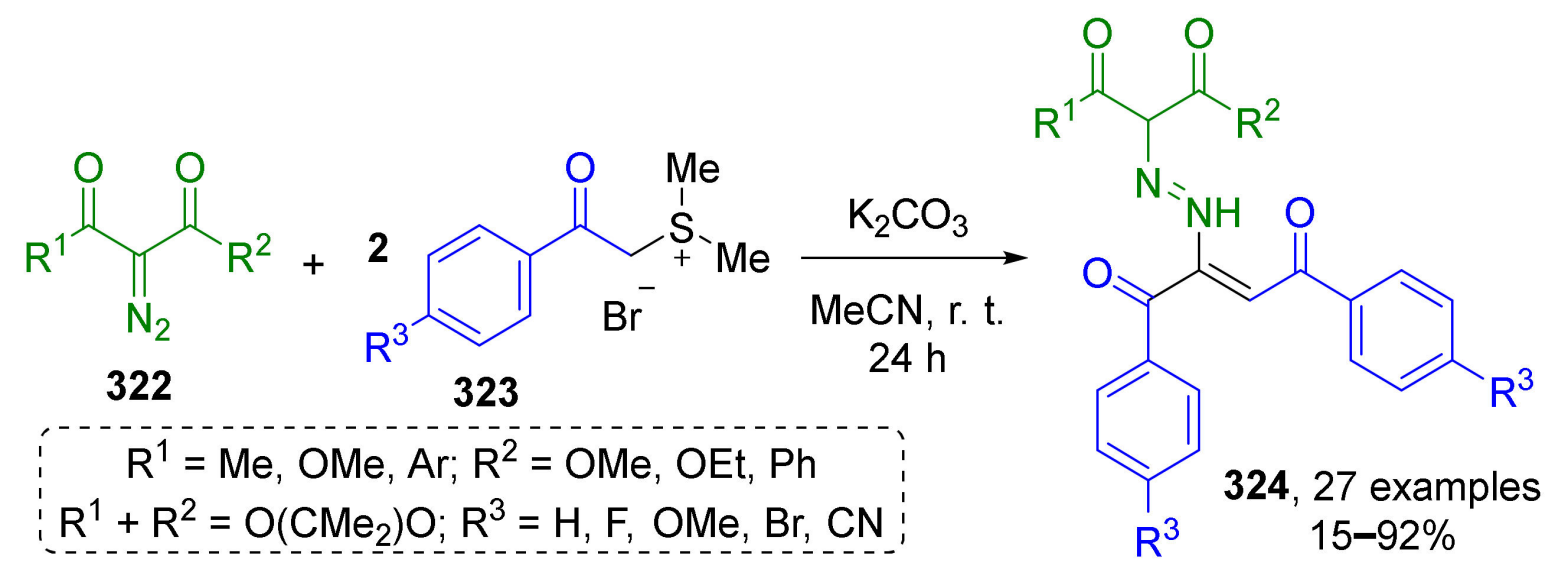
Functionalization of diazo groups by the nucleophilic addition of two molecules of a methylsulfonium ylide.

The arsenal of synthetic applications of nucleophilic addition to the diazo terminal nitrogen atom has recently been extended by a series of works on reactivity of diazo compounds towards sulfonium ylides or sulfoxides. In 2019, X. Xu and coworkers described a transition metal-free MCR reaction of diazo dicarbonyl compounds **322** and two molecules of aroyl sulfonium ylides **323** affording acyclic products—highly functionalized hydrazones **324** (Scheme 95) [102]. The authors successfully introduced various diazo components—ketoesters, malonates, and Meldrum’s acid into this reaction. Remarkably, this is a rare example of generation of functionalized hydrazones from diazo compounds. Normally their roles of starting material and product are reversed—for example, *N*-arylsulfonyl hydrazones are widely used as precursors for diazo compounds (vide infra).

**Scheme 96:**
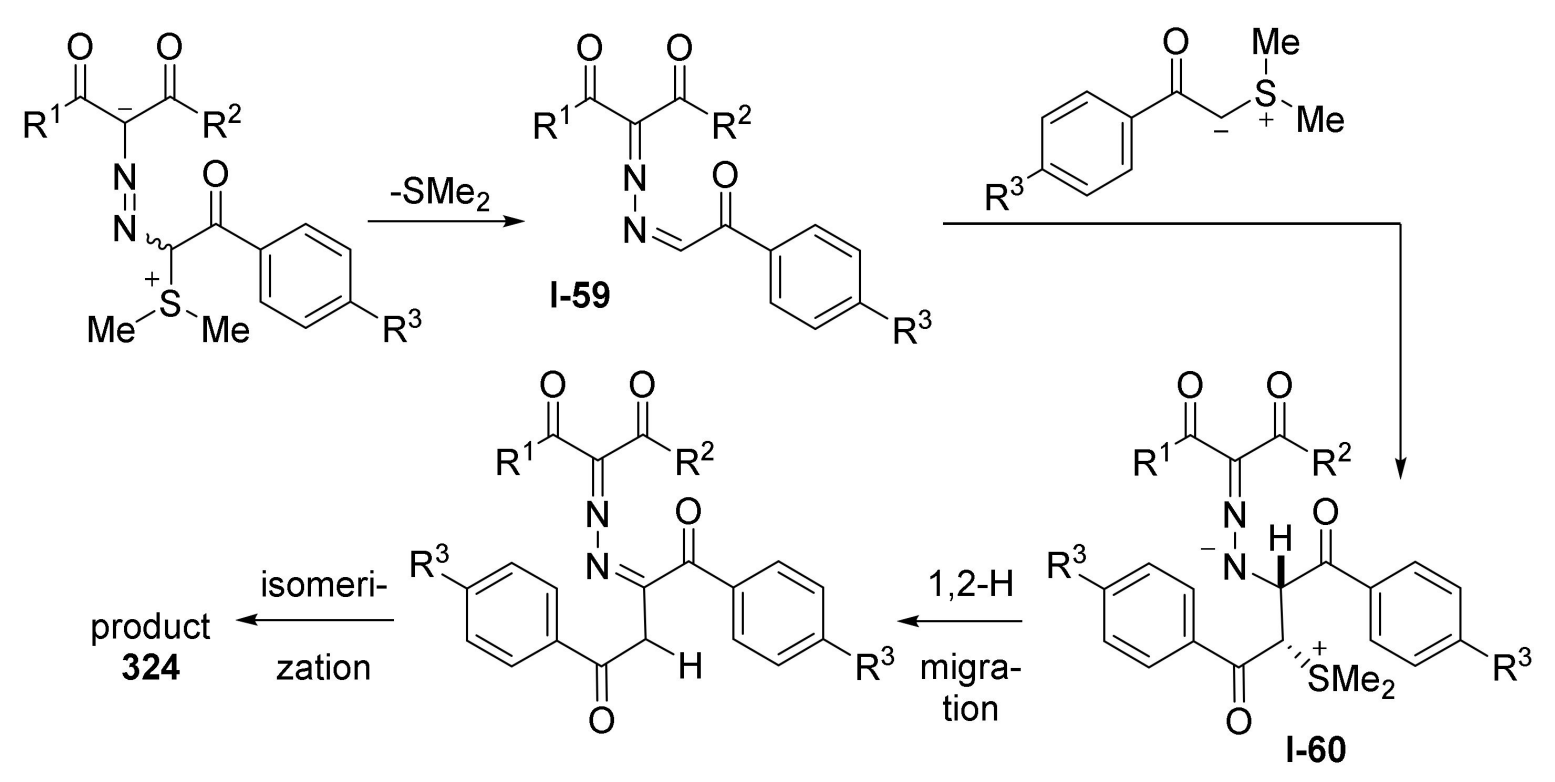
Proposed mechanism for the nucleophilic addition of two molecules of a dimethylsulfonium ylide to α-diazo-β-ketoesters/ketones.

Mechanistically this MCR has following key steps (Scheme 96): first, nucleophilic addition of ylide to terminal nitrogen atom at diazo group, accompanied by elimination of dimethyl sulfide and formation of mixed ket-/aldazine intermediate **I-59**; second, nucleophilic addition of ylide to double CN-bond in aldazine moiety giving zwitter-ionic intermediate **I-60**, followed by SMe_2_ elimination, hydride migration, and imine-enamine tautomerization to isolated products **324**. The authors also suggested that aziridination can be involved as alternative route after second nucleophilic-addition step.

**Scheme 97:**
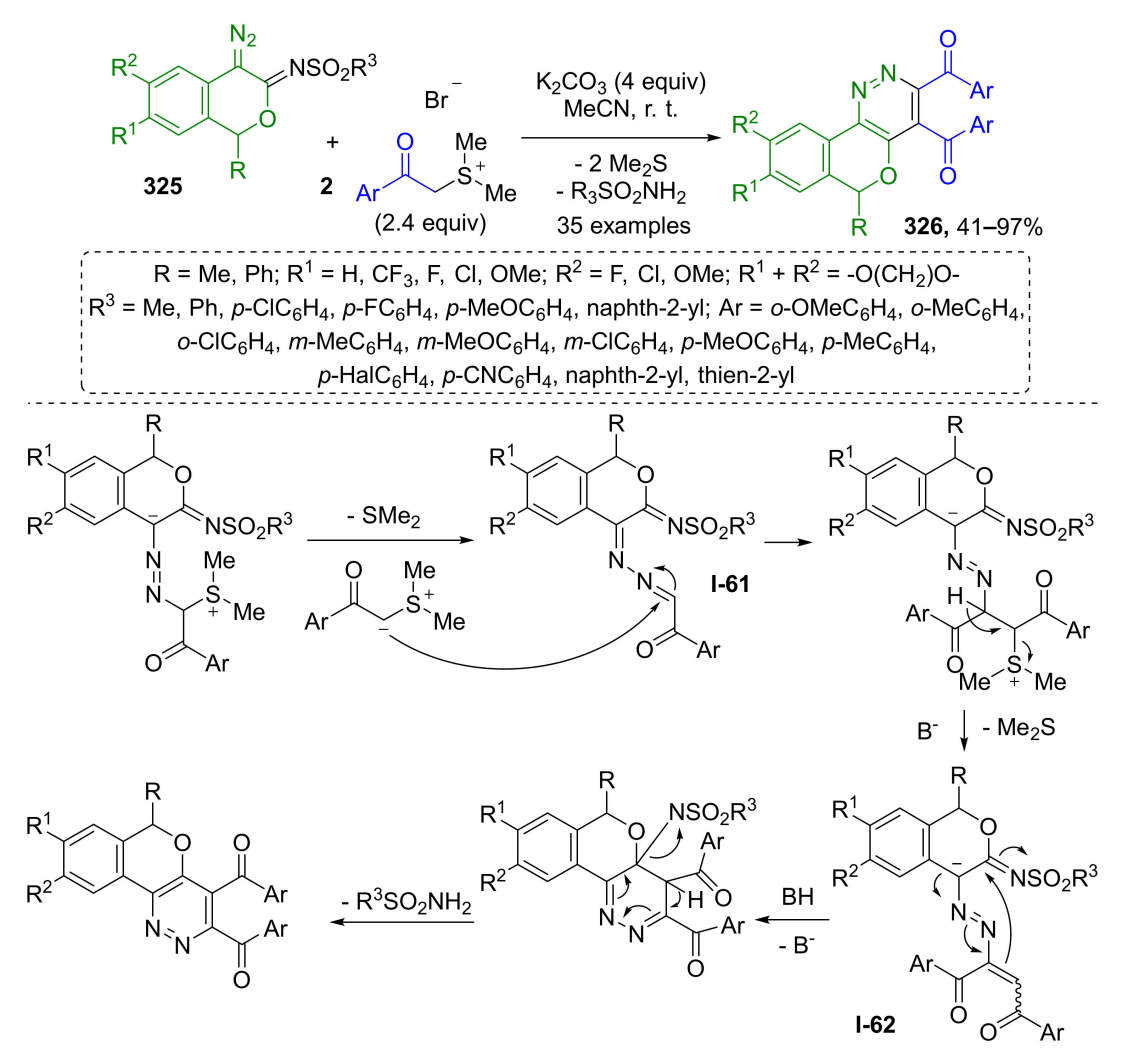
Three-component annulation of 4-diazoisochroman-3-imines with dimethylsulfonium ylides.

Later it was shown that different types of carbo- or heterocyclic products can also be obtained following this strategy. Y. Wang and co-authors described the reaction of the same type of sulfonium ylides with sulfonylimino diazo compounds **325**, derived from isocoumarines (Scheme 97) [103]. In this case, the MCR followed the same mechanistic pathway to azines **I-61**, which further entered an additional cyclization involving adjacent electrophilic imino carbon atom with elimination of NHTs as leaving group giving isochromeno[4,3-*c*]pyridazines **326** as final products. It should be noted that when authors used three equivalents of base instead of four, intermediate **I-62** was isolated and the annulation did not take place.

**Scheme 98:**
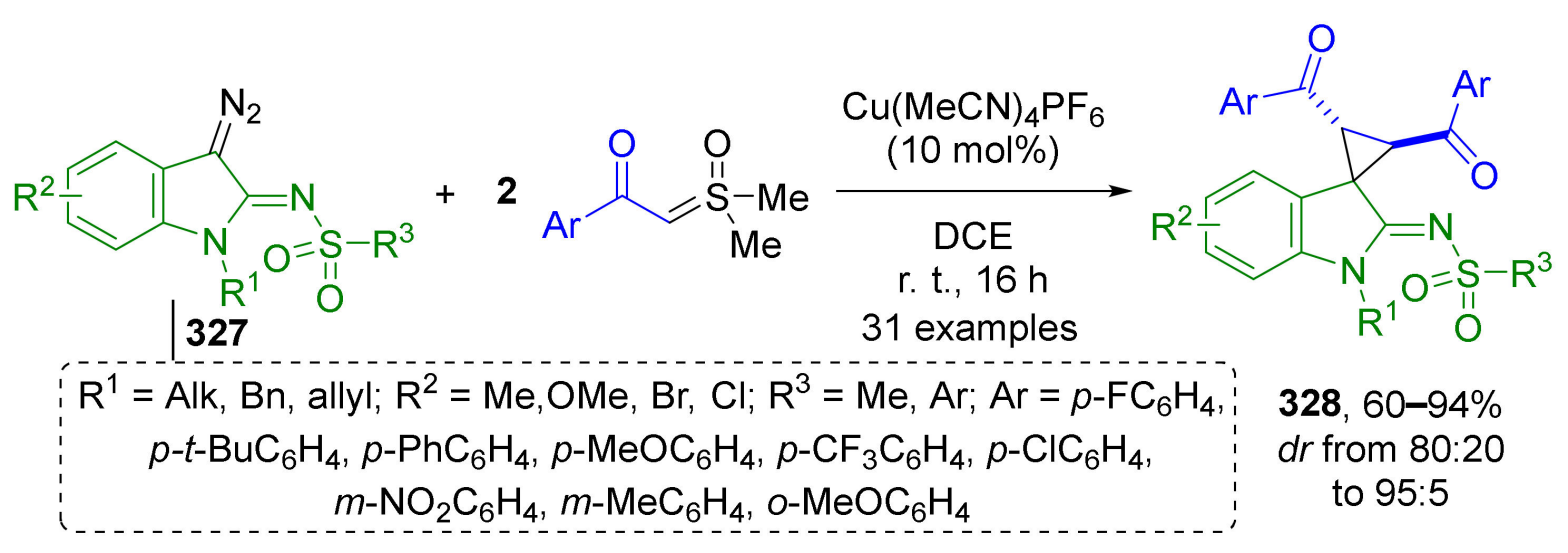
Cu-catalyzed dimerization of sulfoxonium ylides with 3-diazoindolin-2-imines.

The same group also developed a novel approach to spiroheterocyclic cyclopropanes using similar setting (Scheme 98) [104]. In this case, a three-component reaction of sulfonylimino diazo compounds **327** with indoline backbone and sulfoxonium ylides as second reaction partner was employed. This copper-catalyzed reaction delivered complex bisaroyl spiro[cyclopropane-1,3′-indolin]-2′-imines **328** in high yields, broad substituent variability, and diastereoselective fashion despite the formation of three asymmetric units.

**Scheme 99:**
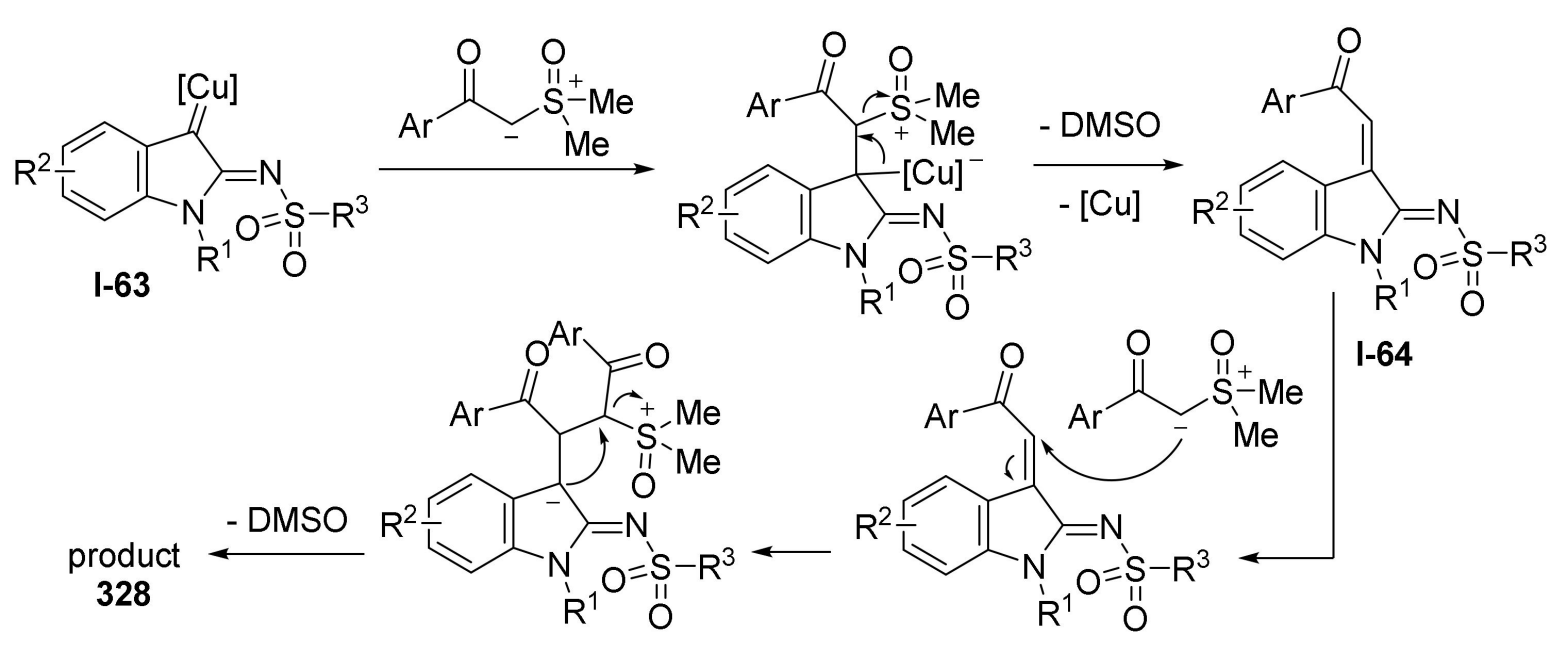
Proposed mechanism of spiro[cyclopropane-1,3′-indolin]-2′-imines formation.

In contrast to two above mentioned works, diazo compounds **327** here react via denitrogenative formation of metal carbenoid **I-63** (Scheme 99). The latter is attacked by the first molecule of nucleophilic ylide which results in formation of isolable intermediate alkene **I-64** after elimination of SMe_2_. Being a part of the reactive 1,4-azadiene system, the newly formed double CC-bond is readily involved into conjugate addition with the second molecule of ylide. Noteworthy, at this step, elimination of SMe_2_ leads to cyclopropanation and the formation of compounds **328** instead of formation of alkenes.

## 5. MCRs with In Situ Generation of Diazo Compound

Certain types of diazo compounds can be successfully replaced with more stable precursors, such as 1,2,3-triazoles or hydrazones bearing a good leaving group (more often, sulfonyl hydrazones). *N*-Tosyl hydrazones is one of the most studied type of diazo compound precursors. Their transformation into diazo compound requires treatment with strong base.

**Scheme 100:**
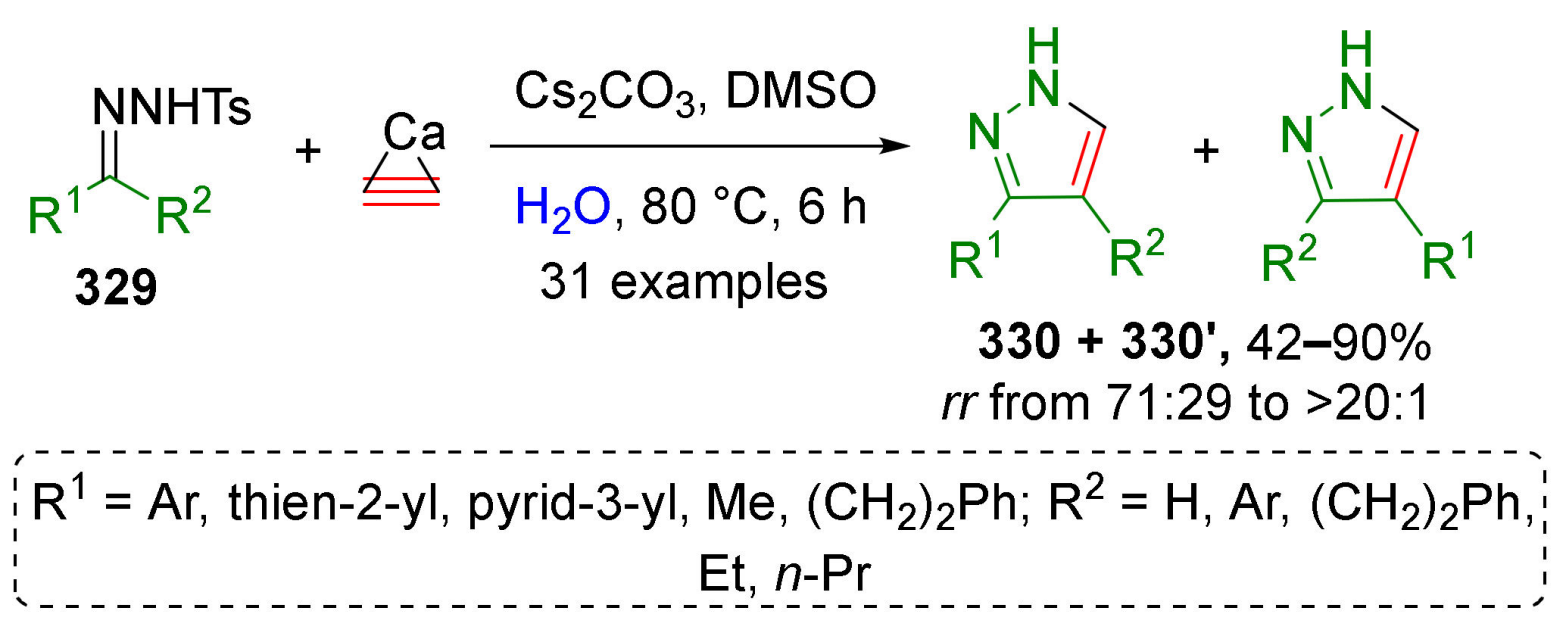
Three-component reaction of calcium carbide, water, and *N*-tosylhydrazones.

In 2016, H. Jiang et al. employed this approach to access mono- and disubstituted pyrazoles **330** and **330′** from *N*-tosylhydrazones **329** and acetylene gas generated in situ from calcium carbide and water (Scheme 100) [105]. The reaction was carried out under heating in DMSO in presence of equimolar amount of cesium carbonate. Both aromatic and aliphatic substituents were well tolerated in hydrazone component. Notably this is an example of diazo compound MCR proceeding without denitrogenation.

**Scheme 101:**
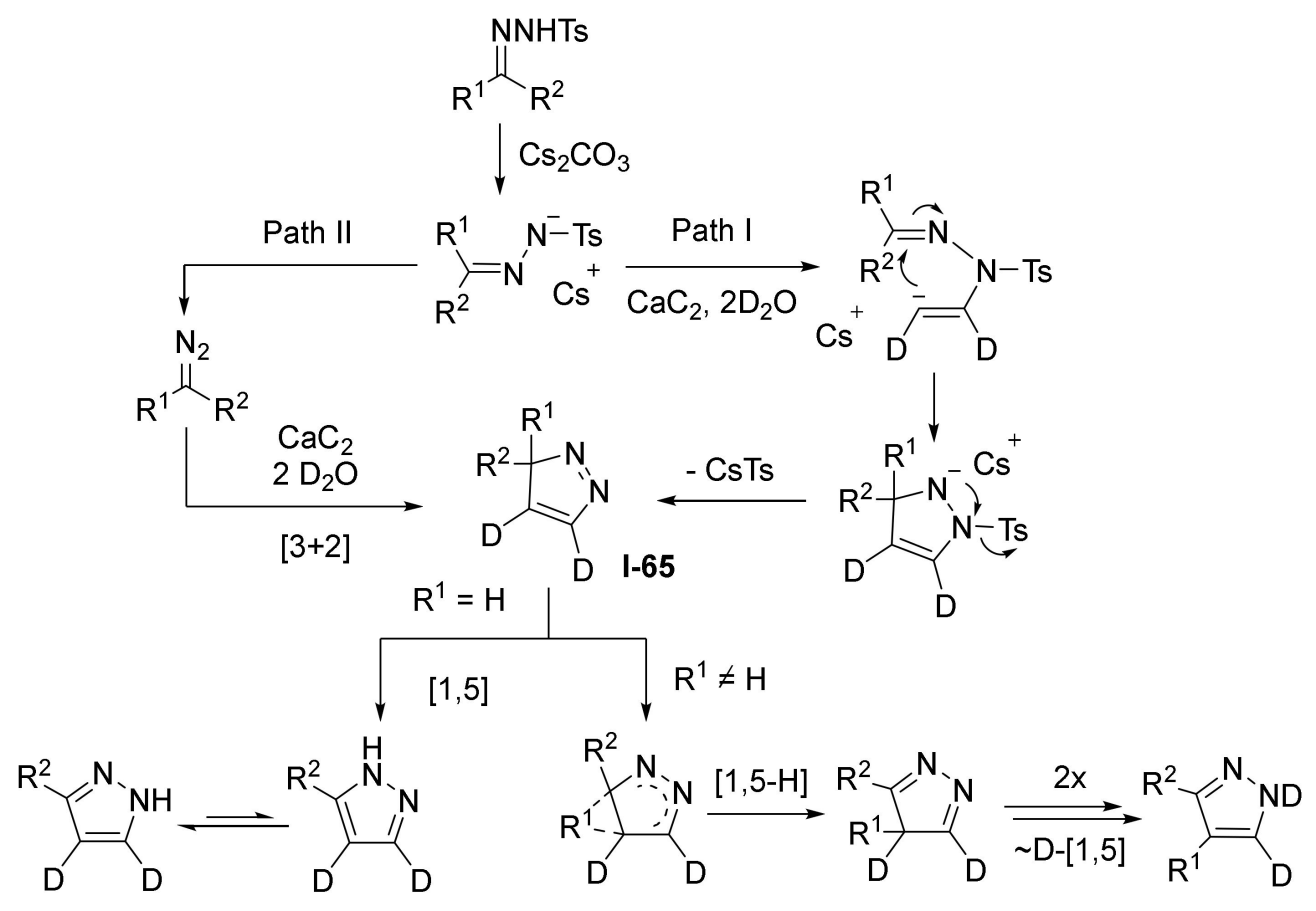
Proposed mechanism of the pyrazole’s formation.

The authors proposed two mechanistic pathways (Scheme 101)—the first one included direct stepwise reaction of tosylimine-anion with acetylene involving nucleophilic addition and cyclization. The second pathway involved formation of diazo compound in the first step followed by [3+2]-dipolar cycloaddition. Both ways lead to formation of 3- or 3,3-substituted pyrazolines **I-65**, which undergo [1,5]-H- or alkyl shifts to provide 3- or 3,4substituted pyrazoles. In the case of ketimines, mixtures of regioisomers were isolated arising from insufficient selectivity of the [1,5]-sigmatropic rearrangement step. The migration of substituents from position 3 to position 4 was supported by isotopic labeling experiments.

**Scheme 102:**
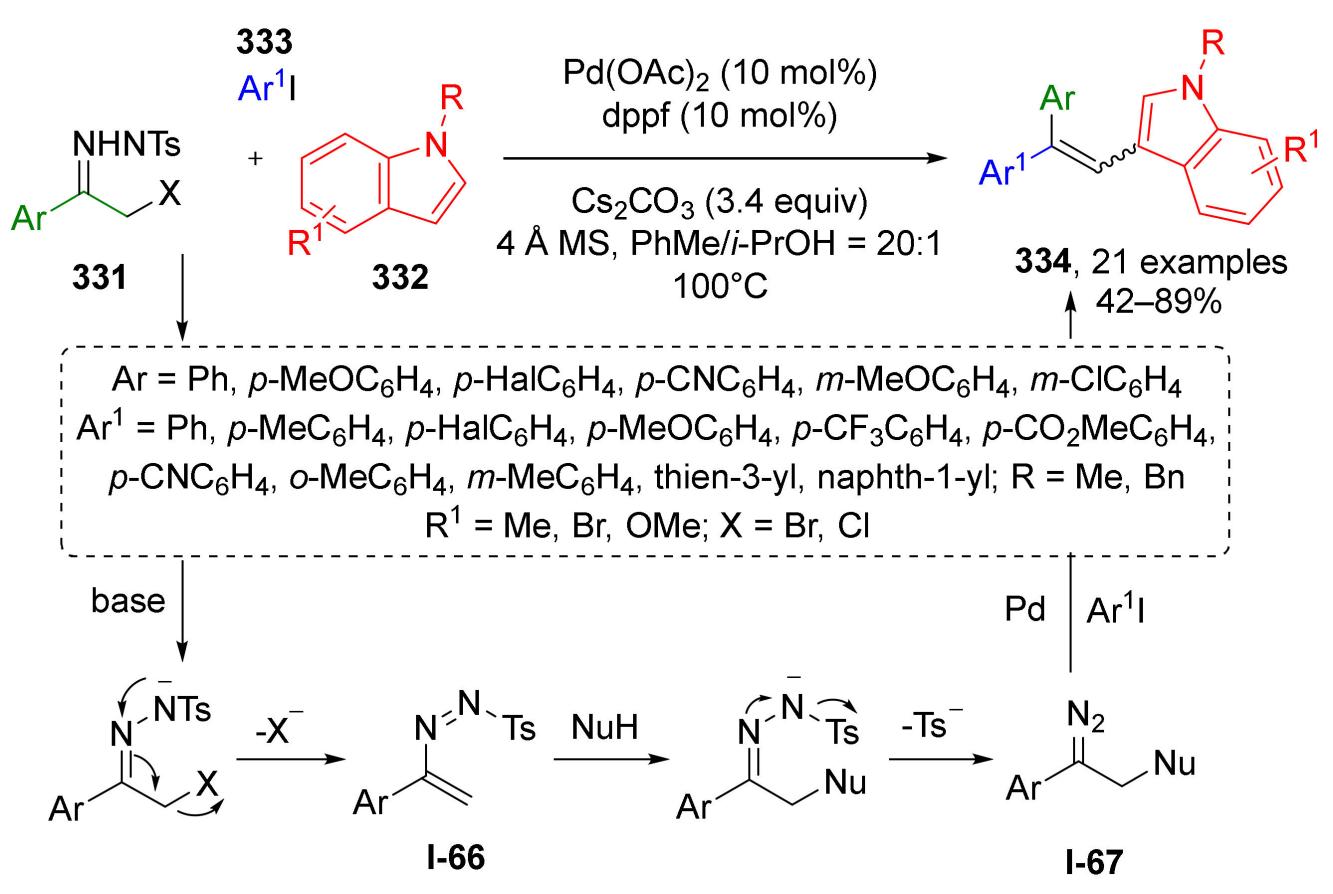
Pd-catalyzed cascade reactions of α-halo-*N*-tosylhydrazones, indoles, and aryl iodides.

In one of the recent works reported by J. Wang and co-authors, this method of in situ generation of diazo compounds was applied for the introduction of diarylvinyl moiety to indoles (Scheme 102) [106]. In this MCR, α-halomethyl *N*-tosyl hydrazone **331** was first transformed into azoalkene **I-66** under treatment with base. This intermediate was then trapped by *C*-nucleophile, *N*-methylindole **332**, and transformed into diazo compound **I-67** after elimination of tosyl group. The olefinic moiety of final product **334** was constructed after Pd-catalyzed coupling of this diazo intermediate with aryl iodides **333**. The authors varied substituents only in one component, aryl iodide, and did not observe any significant influence of electronic nature of substituent on reaction yield.

**Scheme 103:**
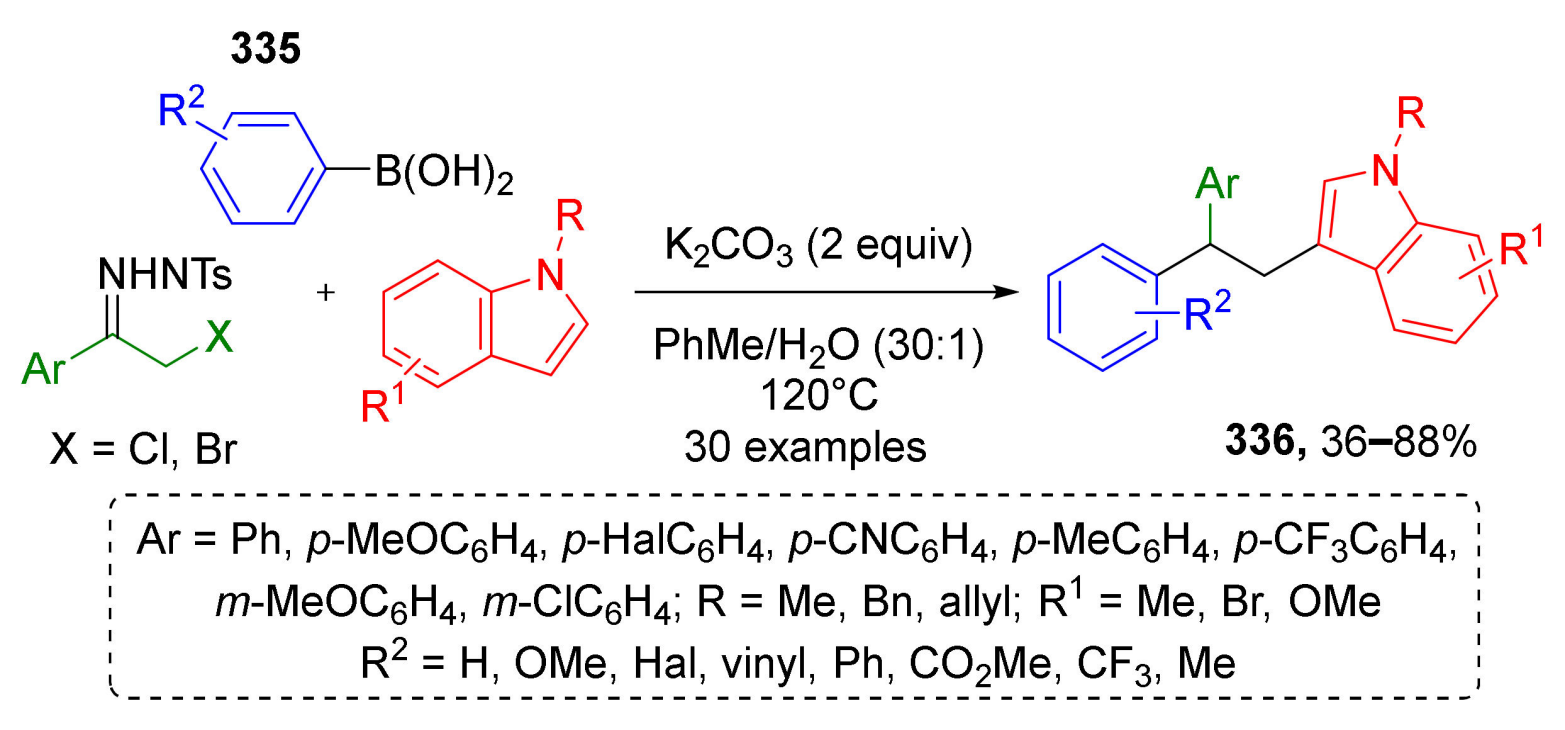
Transition-metal-free cascade reaction of α-halo-*N*-tosylhydrazones, indoles, and arylboronic acids.

Structurally related indole derivatives **336** with reduced side chain can be prepared via the same strategy, switching from aryl iodides to boronates **335** (Scheme 103) [107]. In this case, substituents in all reactants were varied, proving the general scope of this reaction. This approach offers an advantage of transition metal-free protocol.

**Scheme 104:**
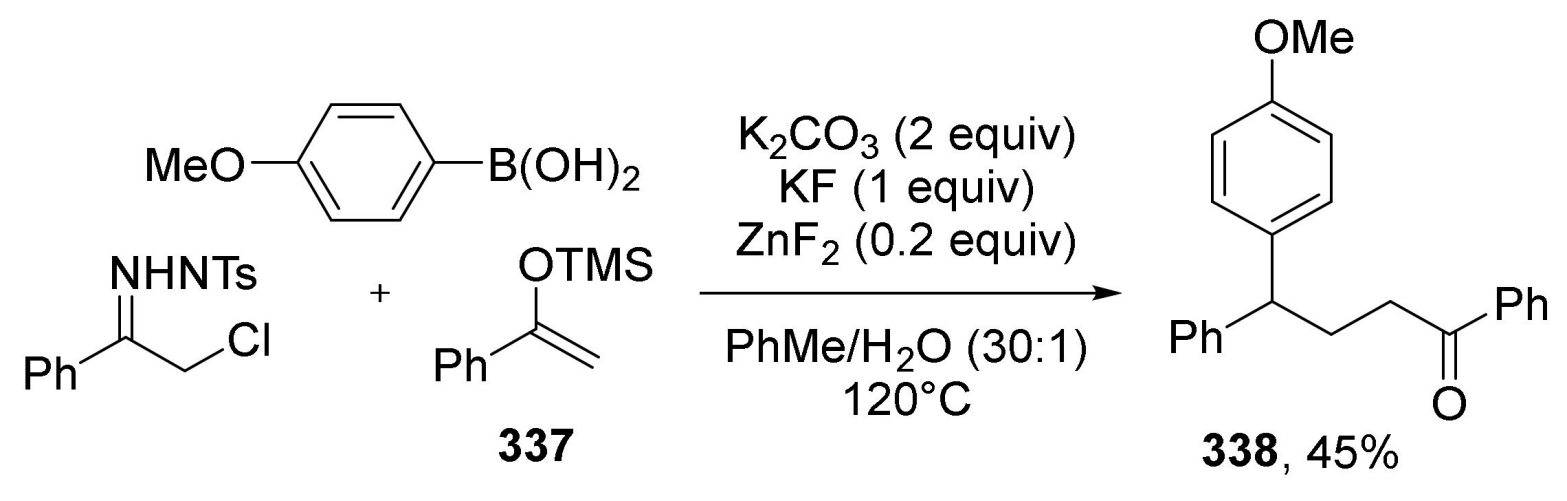
Cascade reaction of α-halo-*N*-tosylhydrazone, silyl enol ether and arylboronic acid.

The authors also showed that *N*-methyl indole can be replaced with acyclic substrate—enolate **337**, which allowed preparation of acyclic product **338** (Scheme 104). In this case, an additional catalyst, ZnF_2_, was required to perform the reaction.

**Scheme 105:**
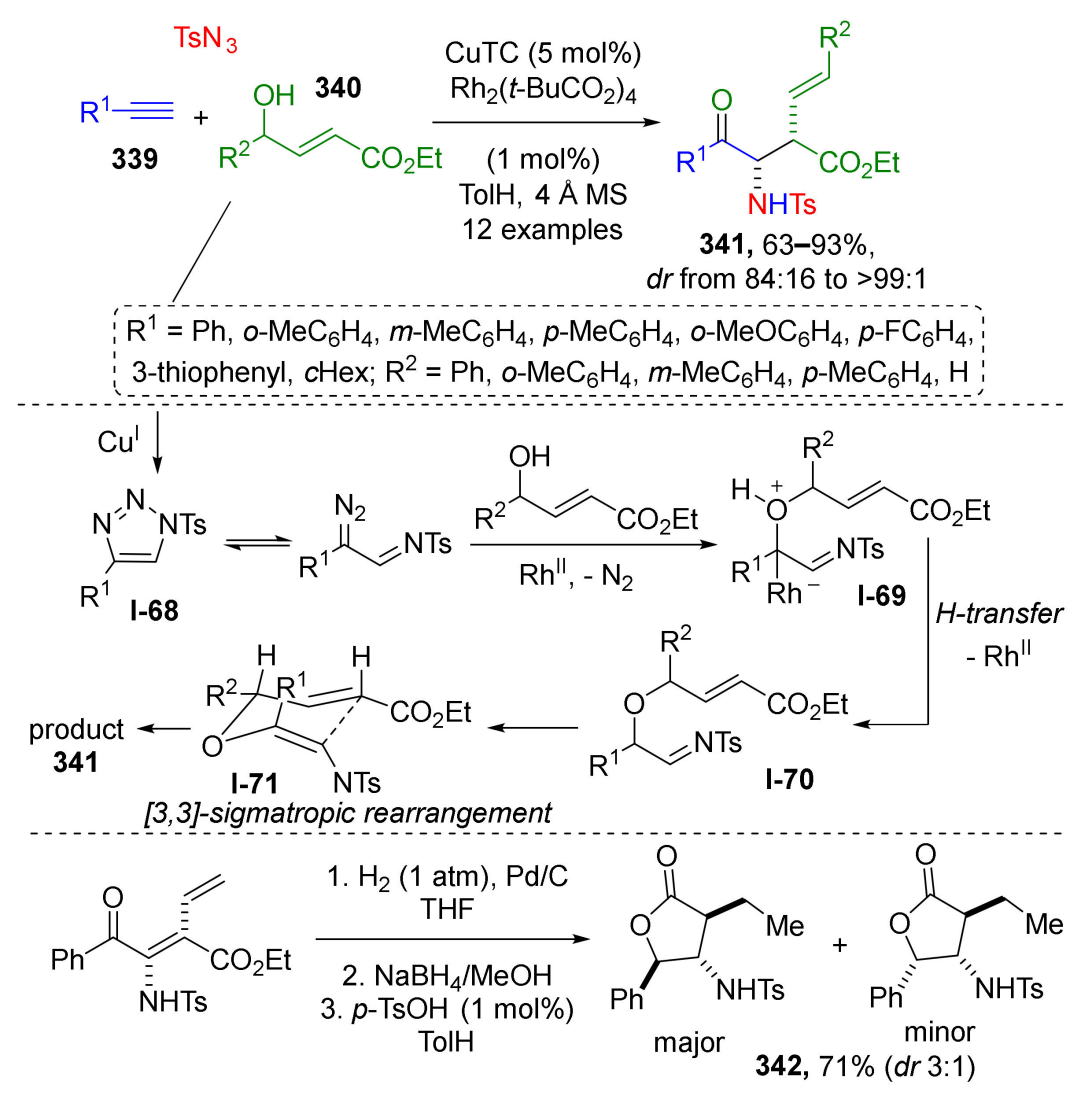
Cu^I^/Rh^II^-catalyzed tandem multicomponent reaction for the regio- and stereocontrolled synthesis of γ-oxo-β-amino esters.

Diazo compounds with α-sulfonylimino group can be prepared in situ via click reaction of terminal acetylenes **339** and tosyl azide, which give *N*-sulfonyl triazoles **I-68** as initial products (Scheme 105) [108]. The latter have equilibrium with acyclic diazo form. Addition of Rh-catalyst generates carbenoid and therefore shifts equilibrium to acyclic form. Recently this reaction was applied for modification of β-hydroxymethyl acrylates **340**, allowing preparation of complex γ-oxo-β-amino esters **341** in a regio- and stereoselective fashion. The reaction mechanism includes generation of carbenoid **I-69** followed by its reaction with hydroxy group of compound **340** to produce oxonium ylide **I-70**, which further undergoes an H-shift to give vinyl ether **I-71**. The final step leading to products **341** is a [3,3]-sigmatropic rearrangement of intermediate **I-72**. The authors also demonstrated that the obtained compounds can serve as useful building blocks for construction of polysubstituted γ-lactones **342**.

**Scheme 106:**
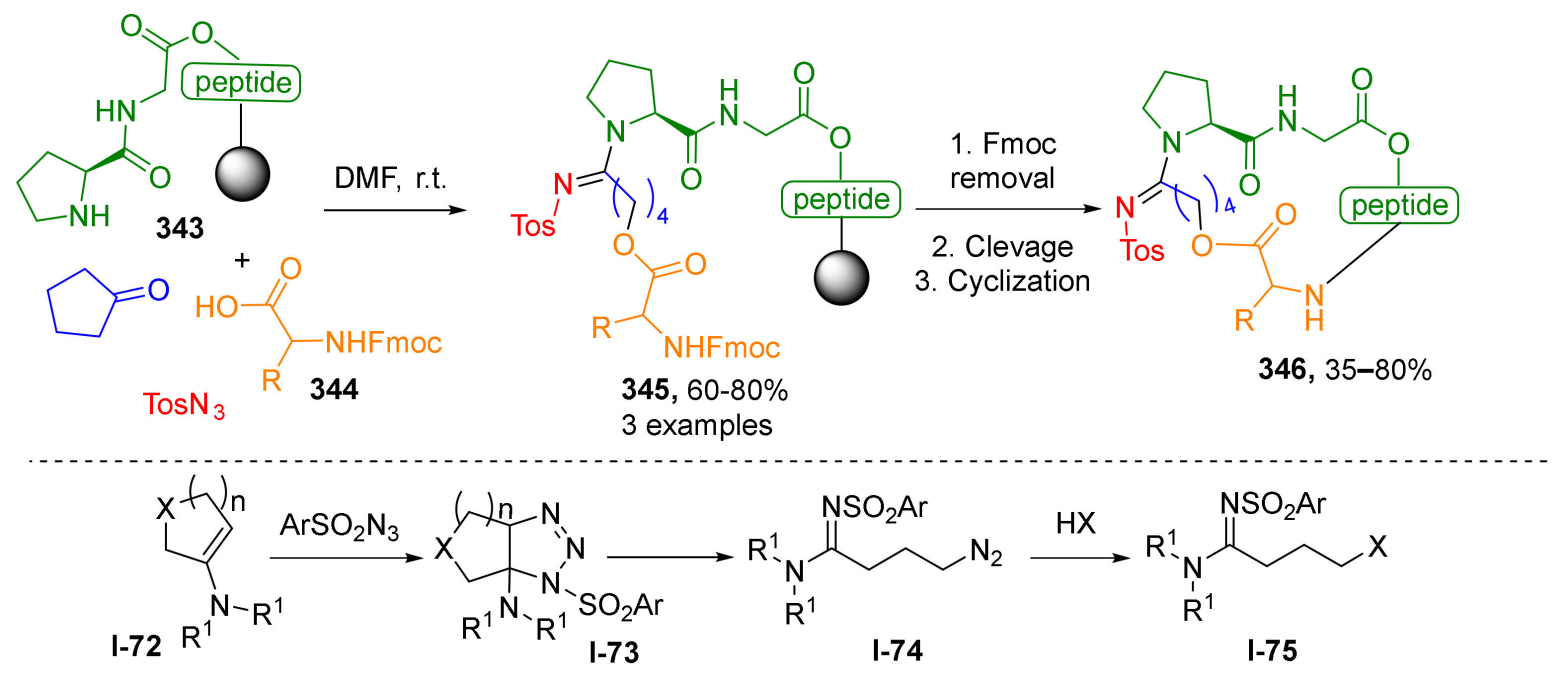
On-resin multicomponent 1,3-dipolar cycloaddition of cyclopentanone–proline enamines and sulfonyl azides.

Another interesting and elegant application of this approach was reported by S. Pellegrino and co-workers (Scheme 106) [109]. A four-component metal-free reaction of peptide with *N*-terminal proline **343**, tosyl azide, cyclopentanone, and *N*-protected amino acids **344** was used for construction of cyclic depsipeptides **346** with incorporated *N*-sulfonyl amidine moiety. In the first step, proline and cyclopentanone reacted to give corresponding cyclic enamine **I-72** via condensation reaction, followed by click reaction with sulfonyl azide. The product of this step, bicyclic amino triazoline **I-73**, was then transformed into diazo intermediate **I-74** via a ring-opening reaction, followed by denitrogenative OH-insertion to amino acid giving compound **I-75**. The latter was transformed into a cyclic peptide **346** after a series of deprotections and intramolecular amidation.

## 6. Miscellaneous Reactions

In this section we will discuss MCRs based on various principles which are difficult to be gathered in a larger group.

**Scheme 107:**
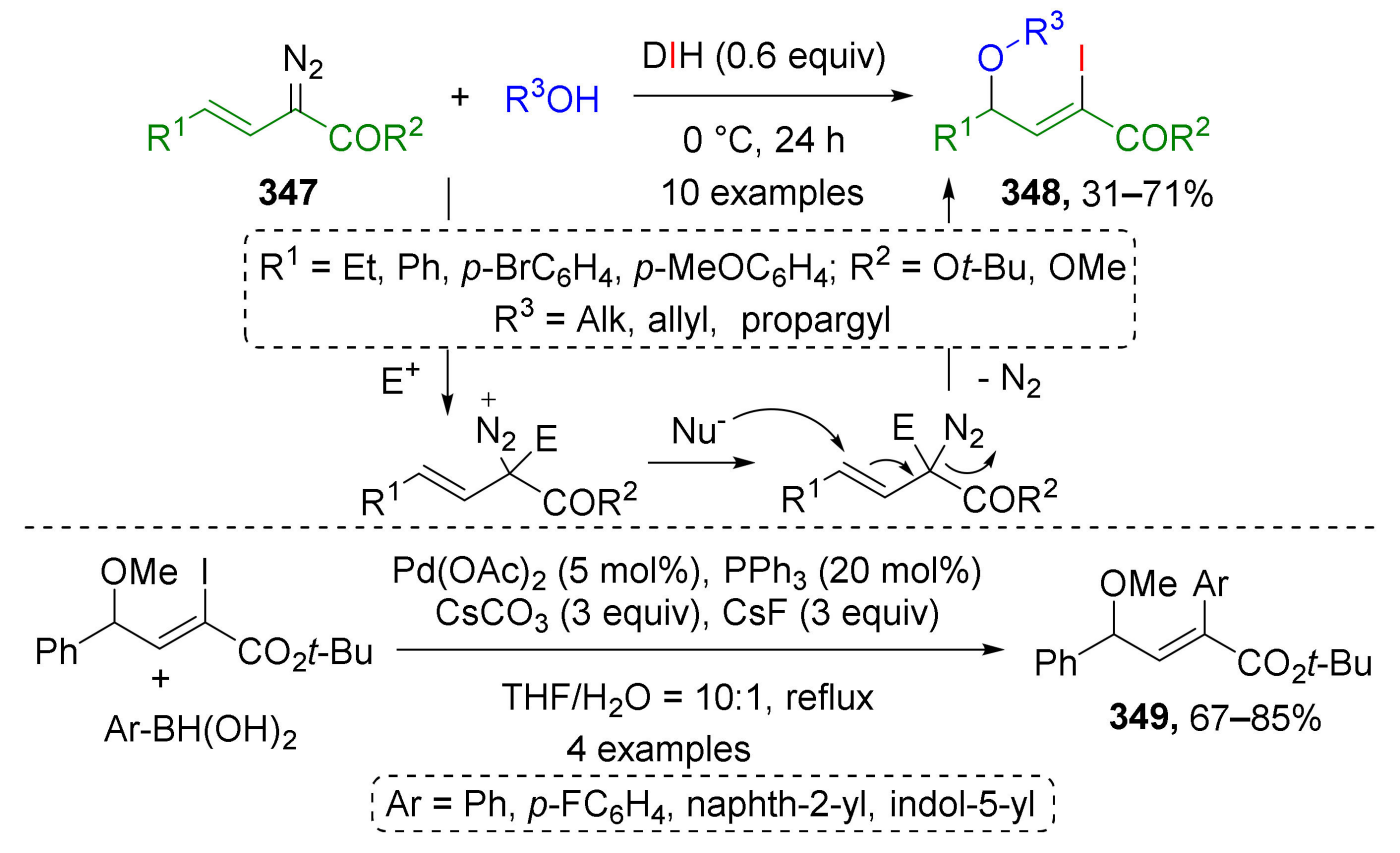
Synthesis of functionalized iodoalkenes using a multicomponent reaction triggered by electrophilic iodination of alkenyl diazoacetates.

While most MCRs described above include formation of at least one new C-C bond, a protocol developed by T. Nemoto and co-workers describes introduction of two functional groups, namely, alkoxy and iodo, to α-alkenyl diazoacetates **347** allowing preparation of iodinated allylic alcohols **348** (Scheme 107) [110]. The reaction mechanism includes bonding between nucleophilic diazo carbon atom and electrophilic iodine (coming from diiododimethylhydantoin, DIH) followed by nucleophilic addition of alcohol molecule and loss of dinitrogen. Notably, different types of aliphatic alcohols, including tertiary ones, are suitable for the reaction and it is catalyst free. It was also shown that this protocol can be coupled with Suzuki reaction by adding aryl boronate and palladium catalyst, which gives compounds **349**.

**Scheme 108:**
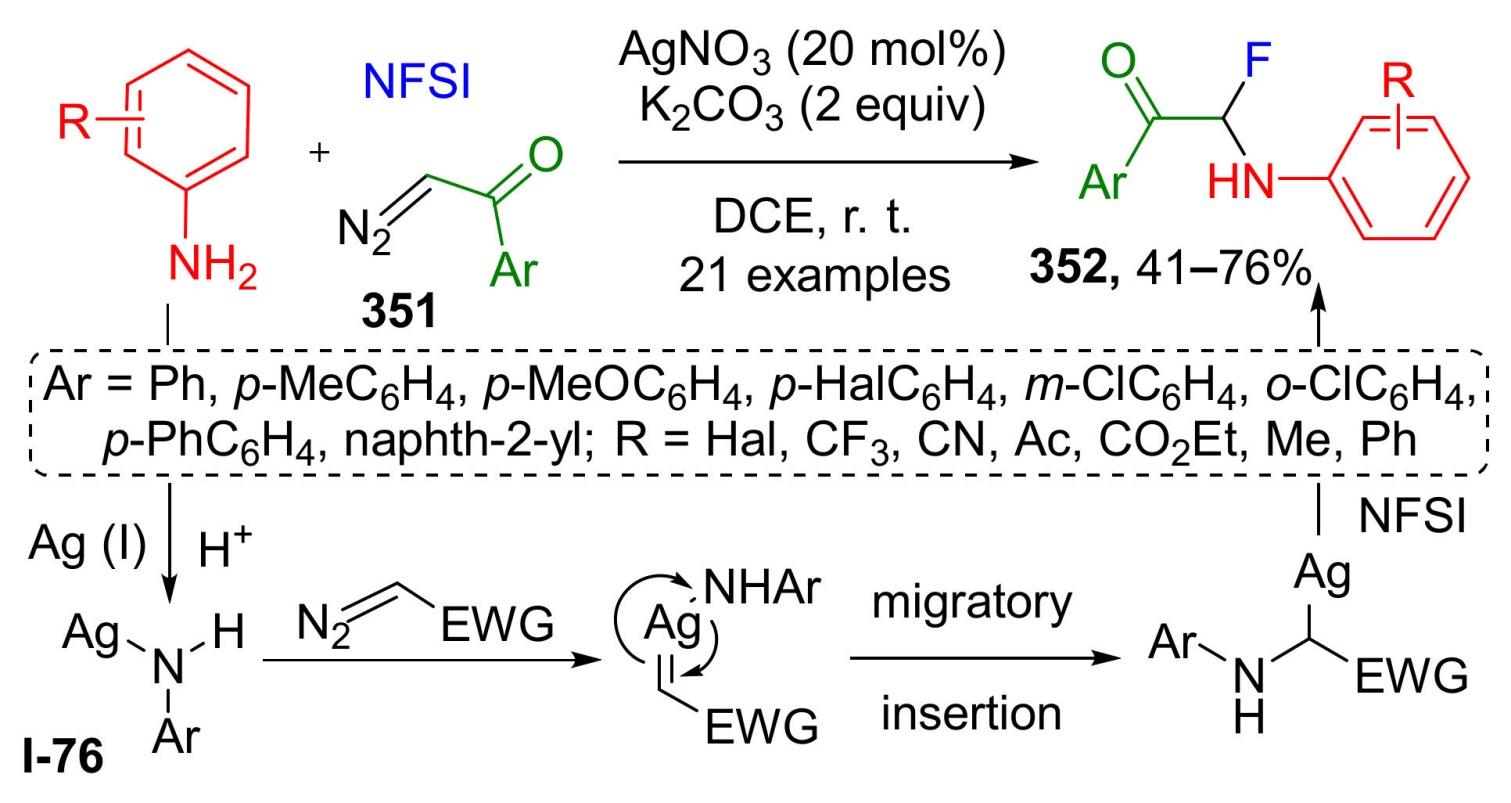
Silver-catalyzed aminofluorination of diazo compounds involving amine ligand migratory insertion.

An interesting example of diazo compound double functionalization occurring at α-position has also recently been reported. In 2017, L. Zhou et al. developed a protocol for *gem*-aminofluorination of diazo acetophenones **350** (Scheme 108) [111], which opens a way to α-fluoro-α-aminoacetophenones **352**. This MCR is based on silver-catalyzed NH-insertion to anilines followed by introduction of electrophilic fluorine, which is provided by *N*-fluorobenzenesulfonimide (NFSI). Control experiments performed by the authors proved the first step of the reaction to be the generation of a Ag(I)/aniline complex **I-76**, rather than the direct decomposition of diazo compound by silver nitrate with formation of an silver carbenoid intermediate. The main difference of this approach from previously published ones is *gem*-aminofluorination with separated nitrogen and fluorine sources combined with the unusual mechanism of amine ligand migratory insertion of Ag carbene.

The development of novel MCRs based on reactivity of metal acetylides has gained much attention recently due to the broad spectrum of valuable transformations for biomedical applications offered by triple C-C bond when introduced to natural product-like scaffolds. Many of these approaches involve the formation of copper acetylides as key intermediates acting as *C*-nucleophiles towards diazo compounds. This interaction results in formation of isolable disubstituted acetylene, which is further trapped by the second electrophilic reagent.

**Scheme 109:**
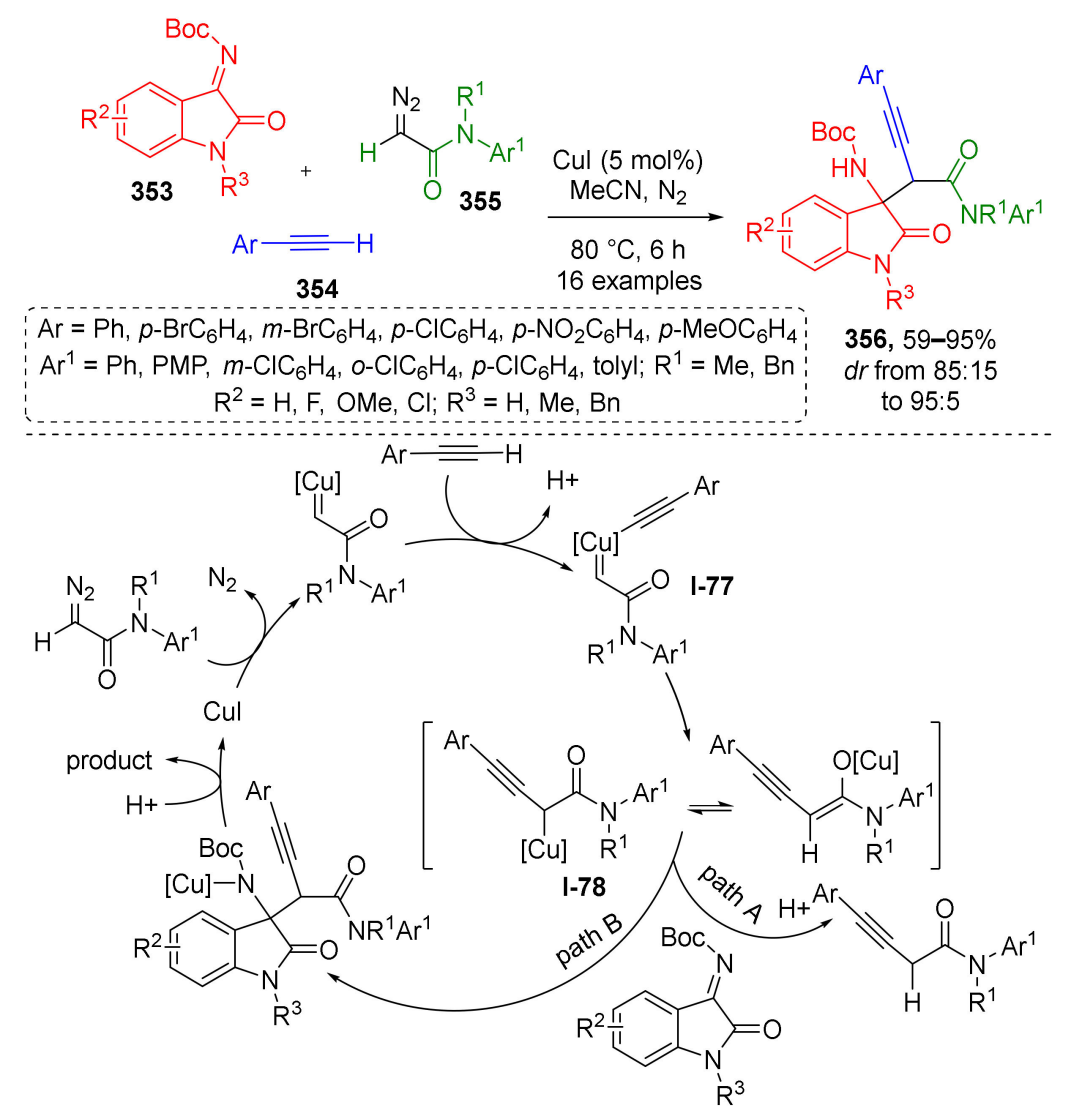
Cu(I)-catalyzed three-component reaction of α-diazo amides with terminal alkynes and isatin ketimines.

In 2019, W. Hu and co-workers reported a related method for the modification of isatin imines **353** with phenylacetylenes **354** and terminal diazo acetamides **355** (Scheme 109) [112]. During the reaction, the copper catalyst brought together carbenoid and acetylide which formed α-alkynyl acetamide **I-78** after migratory insertion as initial reaction product **I-77**. This compound bears highly reactive *C*-nucleophilic center capable of Mannich addition to a third component of the MCR—imine giving substituted amino indolones **356** in a diastereoselective fashion.

**Scheme 110:**
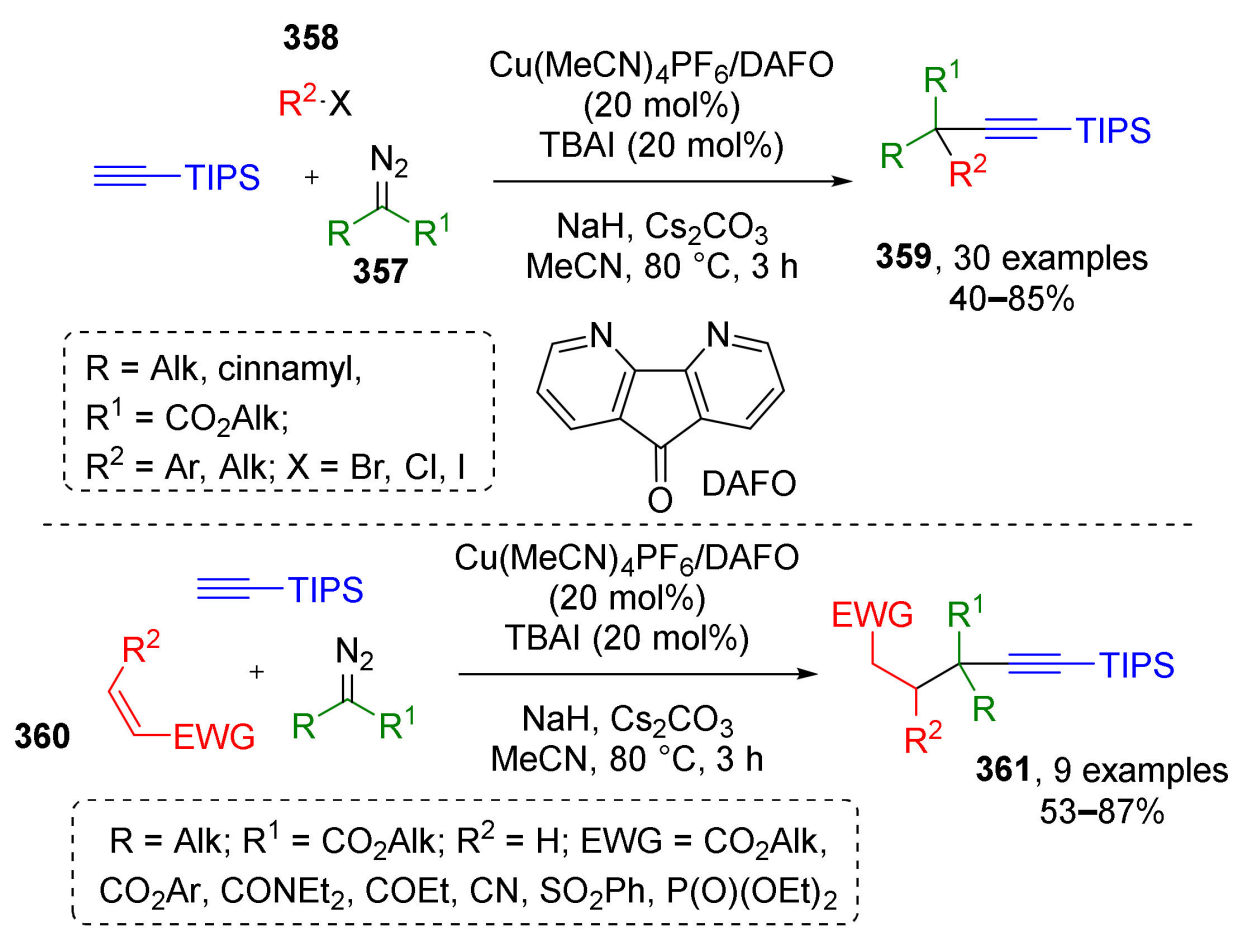
Cu-catalyzed functionalization of terminal acetylenes with diazo compounds combined with alkylation or Michael addition.

This acetylide strategy can be also coupled with trapping by alkylation or Michael addition at the last step leading to formation of quaternary carbon atom without heteroatoms attached. W. Jianbo and co-workers developed a simple and efficient procedure for assembly of compounds **359** and **361** from diazo esters or diazo lactones **357**, TIPS-acetylene and alkyl halides **358**, or Michael acceptors **360** (Scheme 110) [113]. The reaction tolerated a remarkably broad spectrum of substrates including primary and secondary halides (chlorides also), esters, amides, and nitriles of acrylic acid, vinyl ketones, sulfones, and phosphonates. Interestingly the nature of the chelate ligand additive was found to be crucial for this MCR—when classic 1,10-phenanthroline was used instead of 4,5-diazafluoren-9-one (DAFO), the major product was acetylene alkylated with alkyl halide, not a diazo compound.

**Scheme 111:**
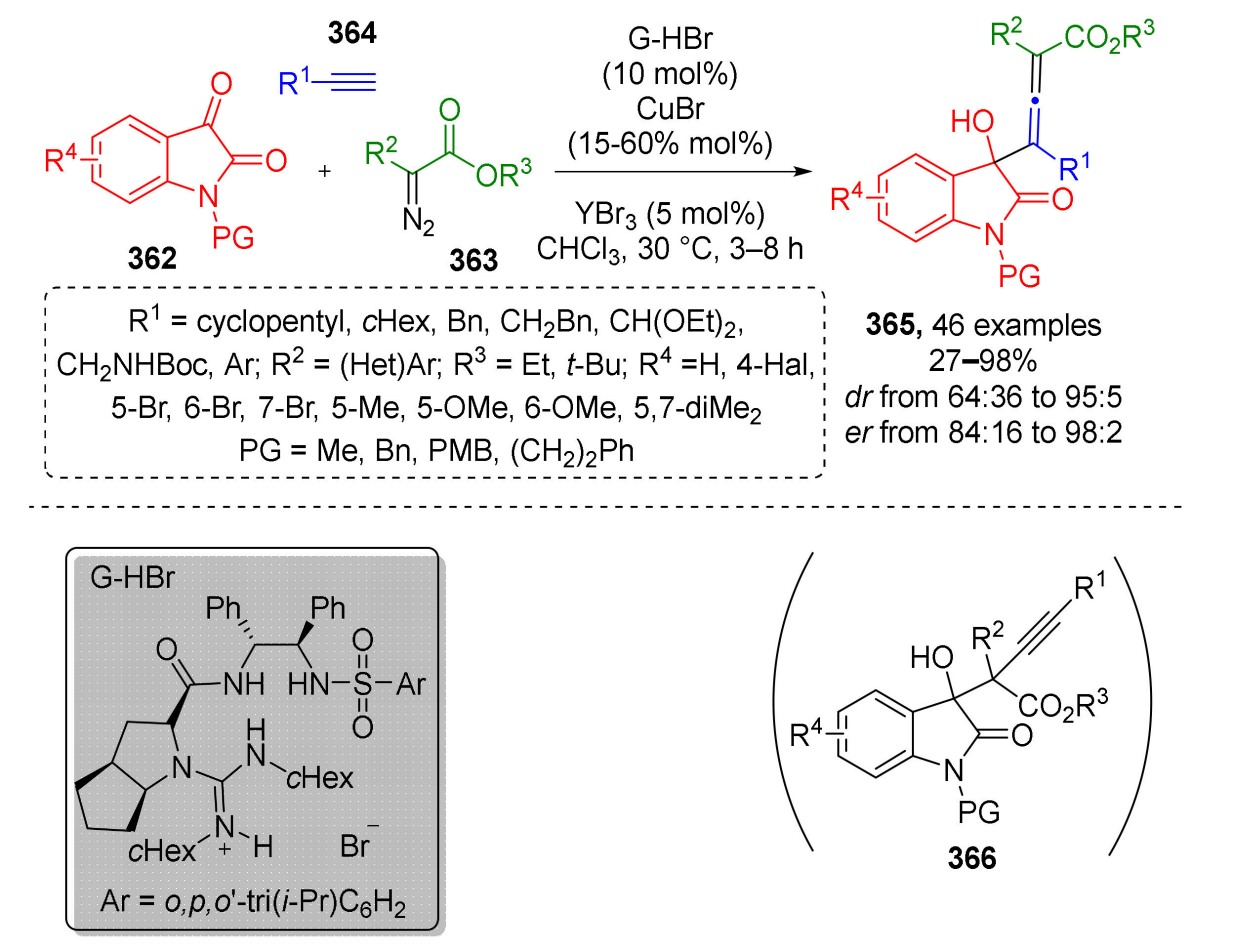
Asymmetric three-component reaction of α-diazo esters, terminal alkynes, and isatins.

Carbonyl compounds such as isatins **362** have also been applied as electrophilic reagents for this approach (Scheme 111) [114]. Depending on the combination of substituents in two other starting materials—diazo ester **363** and acetylene **364** the formation of two products is possible—either allene **365** or alkyne **366**. The use of complex catalytic system consisting of HBr/CuBr/YBr_3_ and chiral organic guanidinium ligand G-HBr allowed authors to perform this MCR in high chemo-, enantio-, and diastereoselective fashion for a broad scope of substrates with isolation of allenes **365**.

**Scheme 112:**
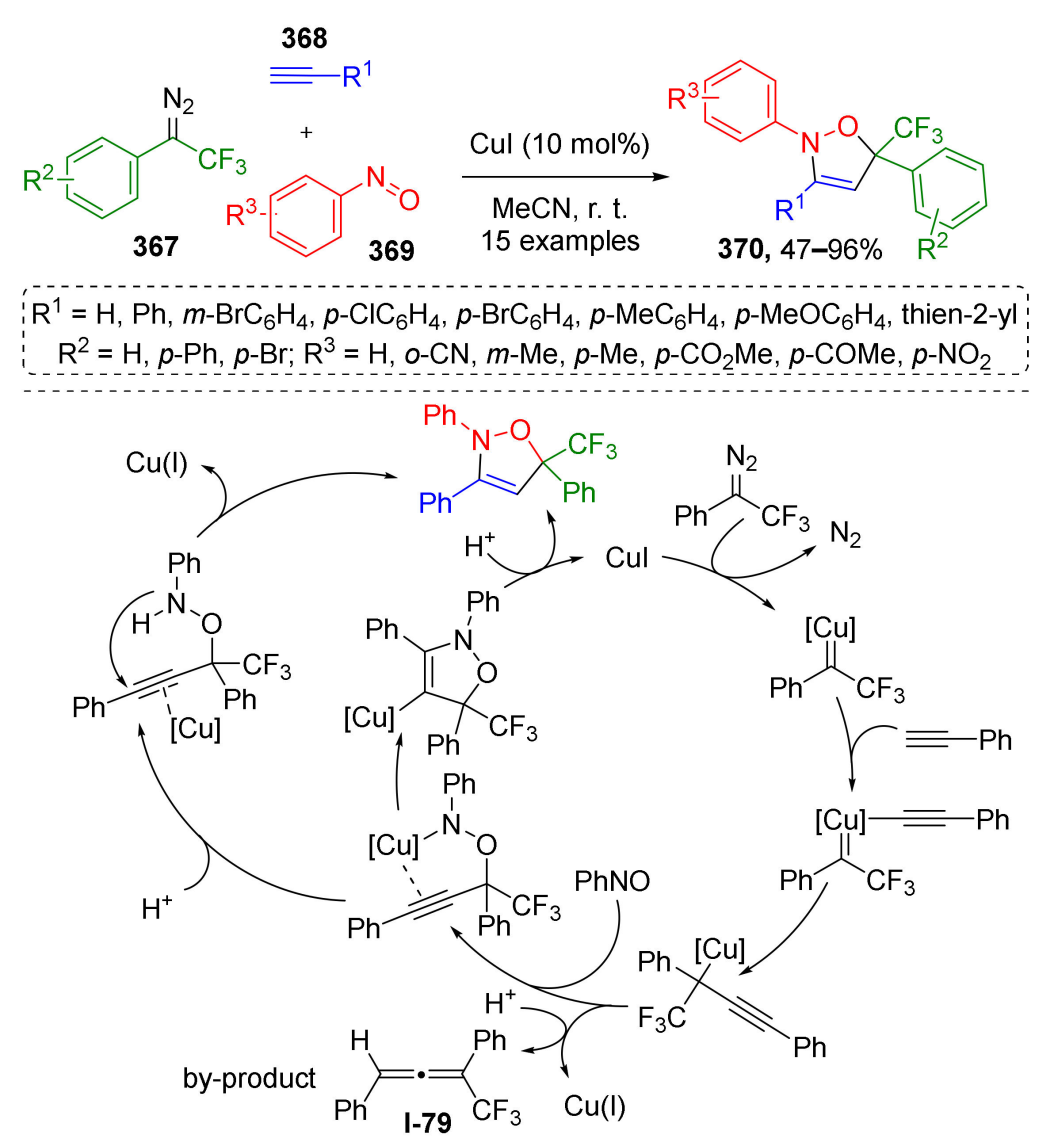
Cu(I)-catalyzed three-component reaction of diazo compound with terminal alkyne and nitrosobenzene.

Another interesting type of electrophilic trapping of Cu carbene intermediate obtained from trifluoromethylated diazo compounds **367** and terminal arylacetylenes **368** was developed by W. Hu and co-workers in 2018 (Scheme 112) [115]. In this case, the third reaction component was nitrosoarene **369** which allowed preparation of rare trifluoromethyl substituted dihydroisoxazoles **370**. The authors also observed allenes **I-79** as by-products arising from protonation of Cu carbenoid intermediate.

**Scheme 113:**
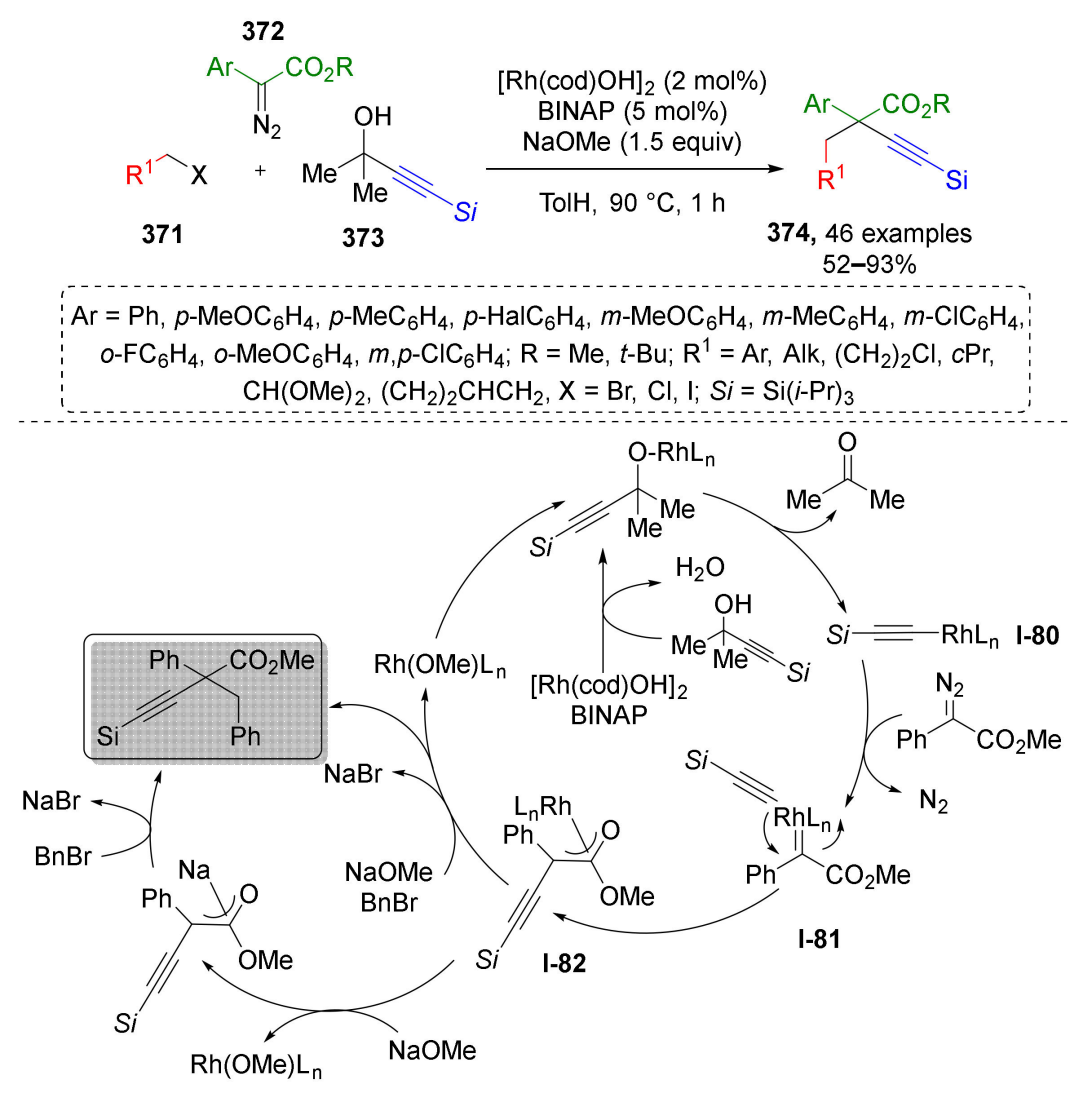
Three-component preparation of functionalized propargylates via Rh-acetylides.

Chemistry of terminal acetylenes is traditionally associated with copper catalysis, but in some cases other metals like palladium or rhodium can be used instead to give better results. For example, J. Wang et al. developed a Rh-catalyzed protocol for construction of acetylenes **374** with an all-carbon quaternary center in alpha-position from alkyl halides **371**, diazo esters **372**, and *tert*-propargyl alcohols **373** (Scheme 113) [116]. The latter were elegantly used as precursors for terminal acetylenes **I-80** which were generated in situ after elimination of acetone molecule (a retro-Favorskii reaction). The next step was coordination of alkyne and diazo compound to rhodium giving complex **I-81** bearing acetylide and carbenoid moieties, followed by migratory insertion of alkynyl group and formation of C-C bond between these two starting materials. This coordinated half-product **I-82** can be alkylated by a third reaction component, alkyl halide, in the presence of base NaOMe, either directly or after dissociation of Rh-complex, giving final products **374**. This MCR was shown to be general and independent of steric or electronic properties of substituents in all components.

Another very powerful type of an alkyne-based MCR of diazo compounds employs base-promoted acetylene-allene rearrangement to produce highly functionalized allenes, which can be transformed into alkenes after addition of nucleophilic species. The key intermediates here are propargylic systems, often generated from diazo compound and terminal acetylene.

**Scheme 114:**
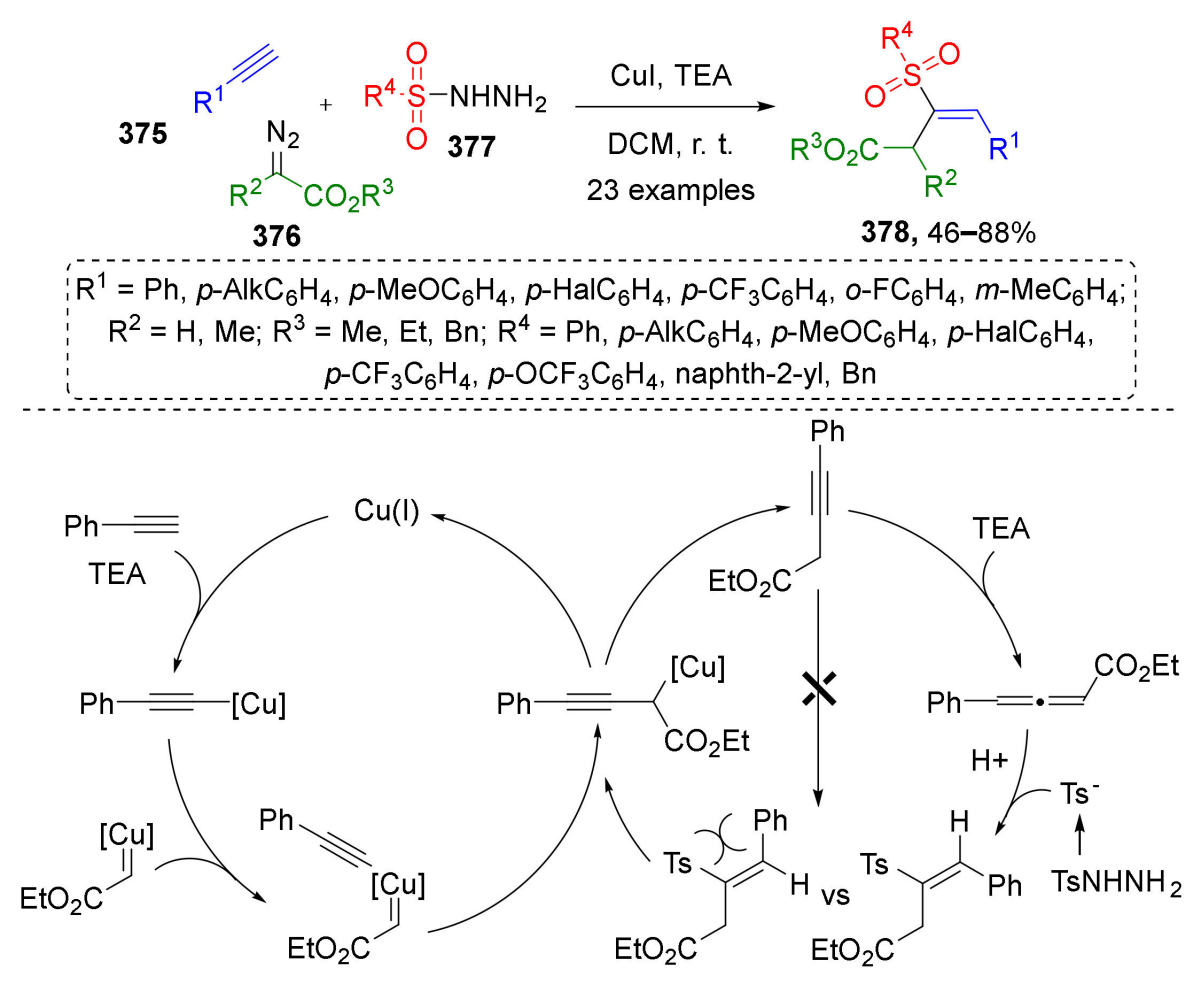
Copper-catalyzed 1,1-difunctionalization of terminal alkynes.

Z. Wang et al., in 2019, reported such a protocol for preparation of vinyl sulfones **378** from aryl acetylenes **375**, diazo esters **376**, and sulfonyl hydrazines **377** under CuI catalysis (Scheme 114) [117]. In this approach for 1,1-(geminal) difunctionalization of acetylenes sulfonyl hydrazine served as a source of nucleophile—tosyl anion. Notably, only one stereoisomer of product **378** was formed during reaction.

**Scheme 115:**
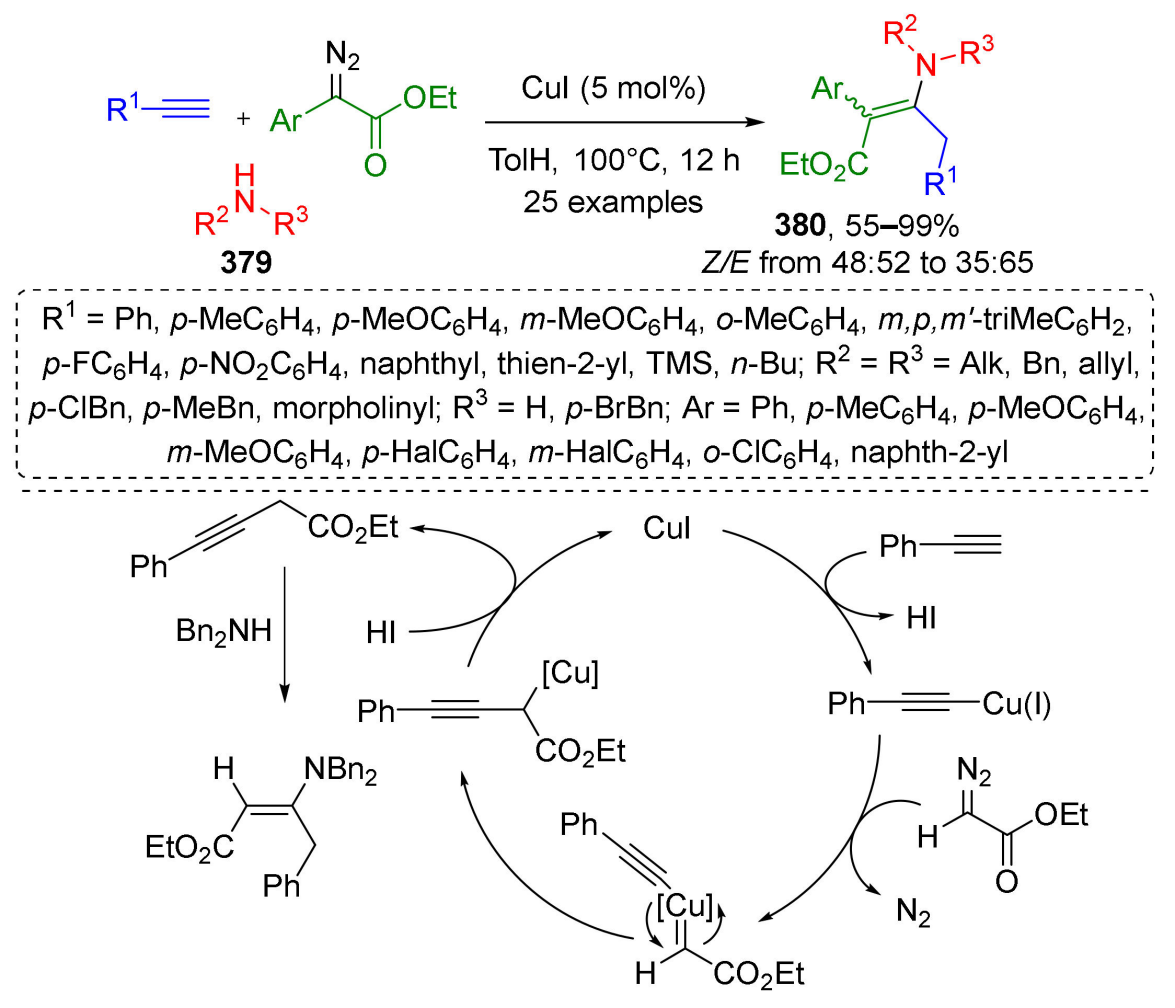
Cu-catalyzed difunctionalization of terminal alkynes with diazo esters and amines.

Depending on reaction conditions, nucleophilic addition to propargylic substrates can occur directly without rearrangement, thus representing a 1,3- (or vicinal) difunctionalization of starting acetylenes. One such example was published by H. Huang and co-workers in 2016 (Scheme 115) [118]. The authors discovered that secondary amines **379** can be involved in three-component Cu-catalyzed reaction with aryl acetylenes and diazo esters to give β-enamino esters **380**. In contrast to the previous work, no external base was used which prevented acetylene-allene rearrangement and resulted in direct hydroamination of triple C-C bond with dibenzyl amine. This reaction showed a general substrate scope but low stereoselectivity—all products were isolated as *E*-/*Z*- mixtures.

**Scheme 116:**
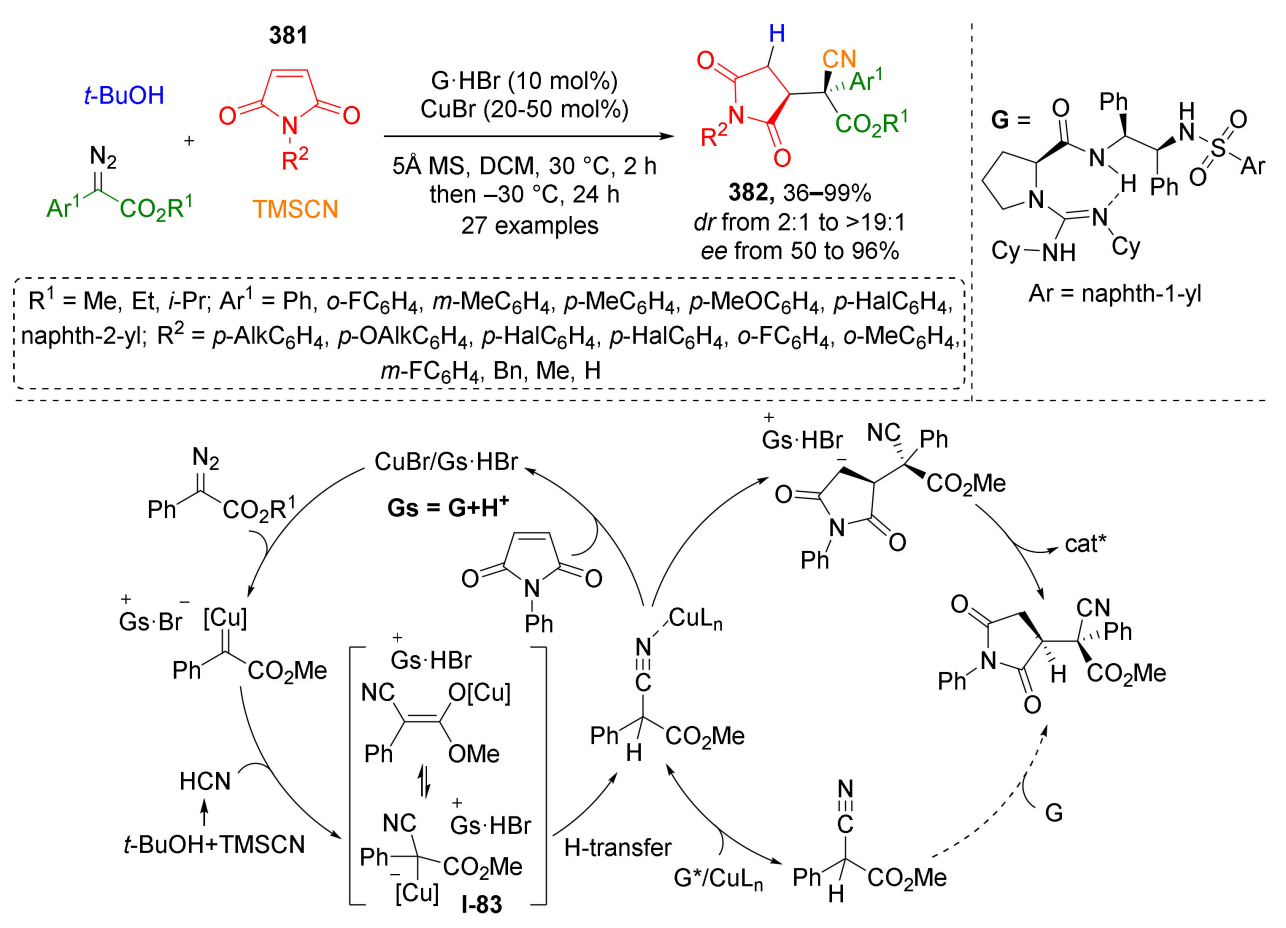
Asymmetric hydrocyanation/Michael reaction of α-diazoacetates via Cu(I)/chiral guanidine catalysis.

Copper catalysis can be efficiently used to facilitate reactions of diazo compounds not only with acetylenes. In 2020, X. Liu and co-workers showed that chiral succinimides with exocyclic all-carbon asymmetric center can be assembled via three-component reaction of diazo esters, cyanide anion, and *N*-arylmaleimides **381** (Scheme 116) [119]. The key step of this sequence was Michael addition of Cu-carbenoid **I-83** derived from diazo compound and HCN generated in situ from *t*-BuOH and TMSCN. Chiral additive, guanidinium salt **G**, enabled stereoselective preparation of products **382** with quite high diastereo- and enantioselectivities.

Several of the recently developed MCRs of diazo compounds employ an interesting reactivity of 2-oxazolines which are prone to ring-opening reactions providing *N*-(ω-substituted ethyl) carbamoyl fragments to the target molecules. When combined with CH-activation and chemistry of diazo compounds, this approach gives annelated lactams.

**Scheme 117:**
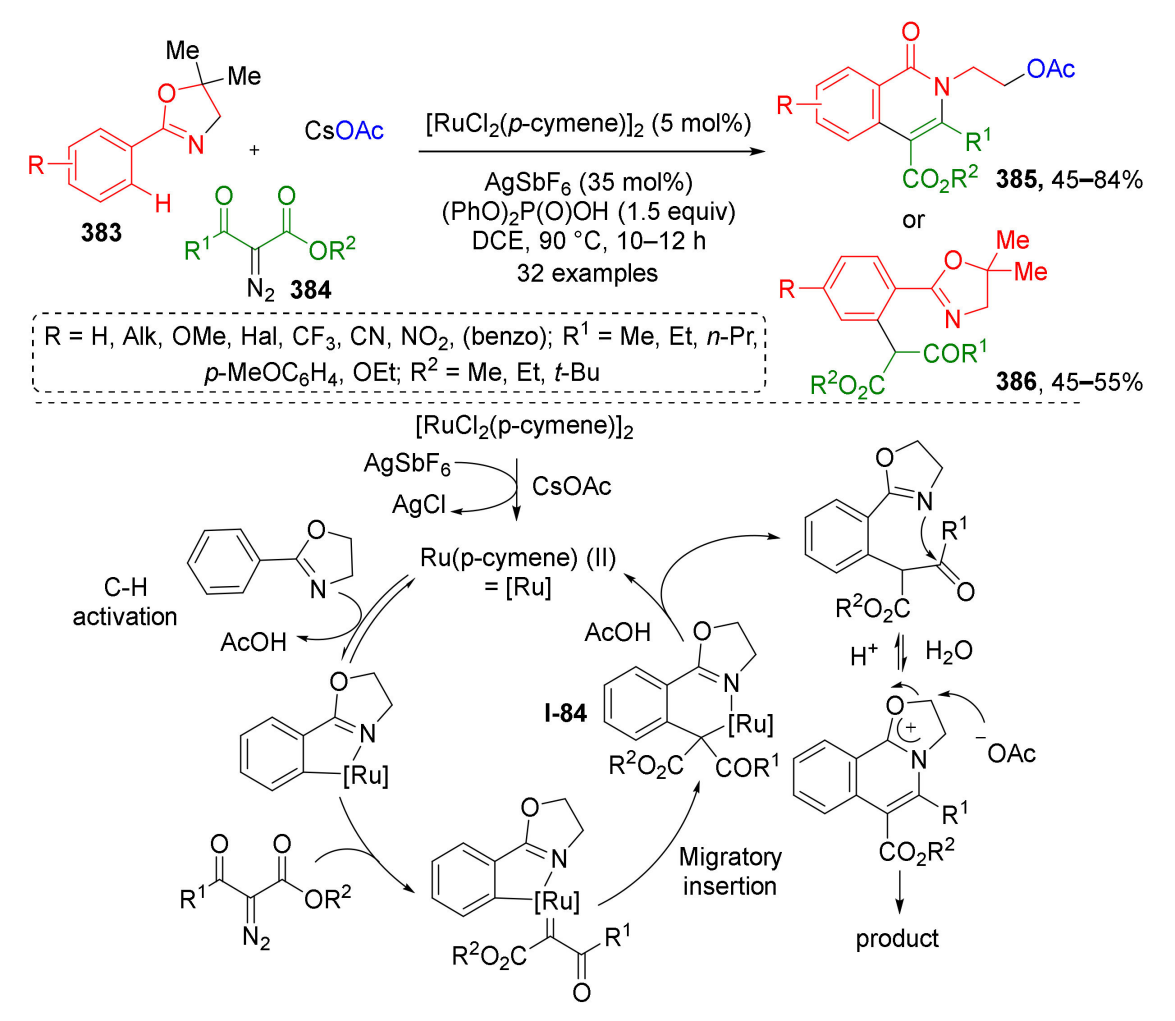
Oxazoline-assisted Ru(II)-catalyzed C-H functionalization based on carbene migratory insertion.

M. Kapur et al. showed that such a three-component reaction of aryl oxazolines **383**, diazo ketoesters **384**, and cesium acetate opens facile access to functionalized isoquinolones **385** under dual Rh/Ag catalysis (Scheme 117) [120]. Noteworthy, the reaction proceeds via formation of rhodacyclic species **I-84**. Acetate anion serves here as nucleophilic reagent opening the oxazoline ring and is incorporated into the molecule as a part of substituent at the nitrogen atom. The reaction was found to be sensitive to the substitution pattern in all components and the substrates with dimethyloxazoline ring, or heteroaryloxazolines, or diazo diketones were not suitable for the preparation of isoquinolones. In some cases, only arylation of diazo compound occurred without ring opening of oxazoline giving compounds **386**.

**Scheme 118:**
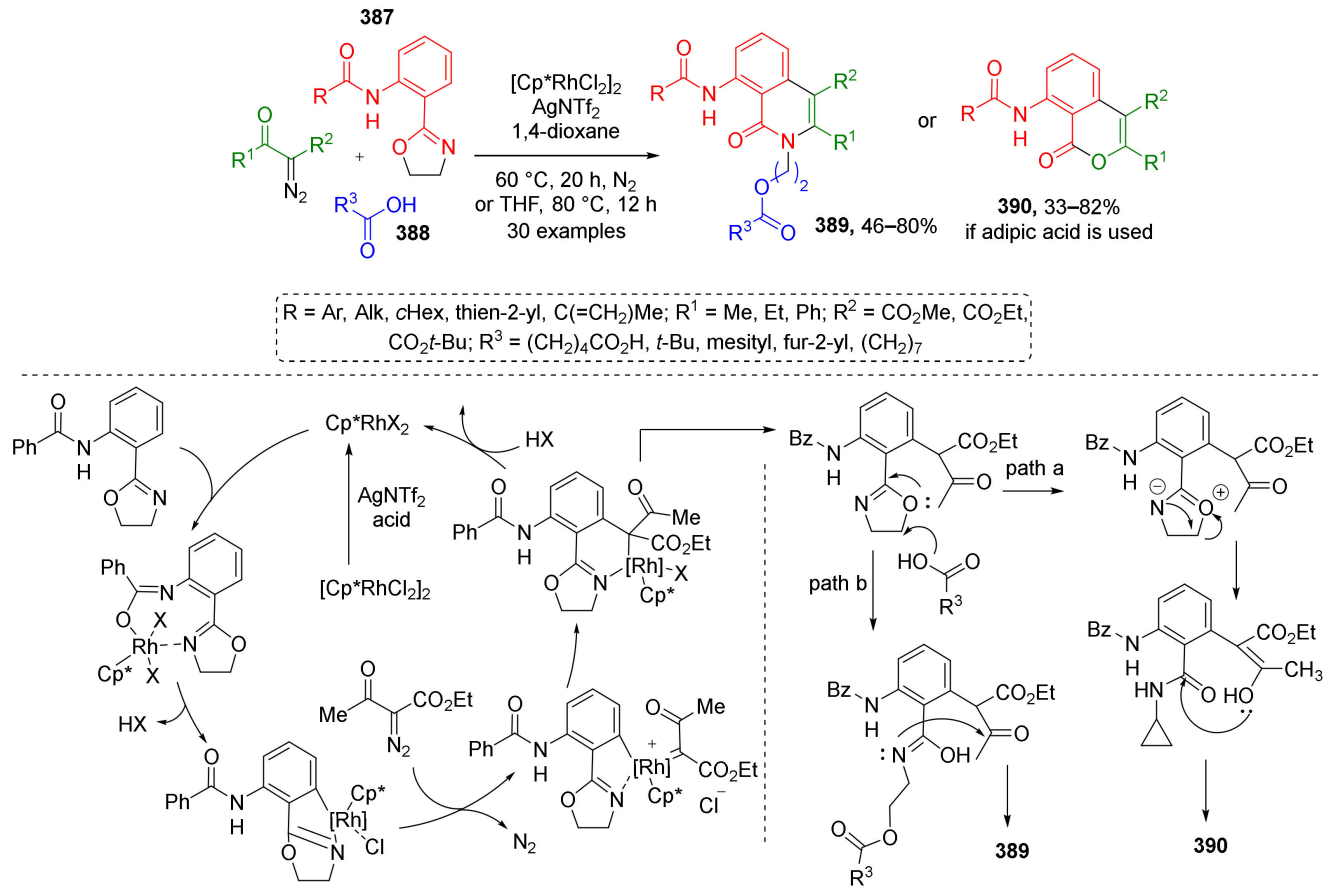
Rhodium(III)-catalyzed chemodivergent annulations between phenyloxazoles and diazo carbonyls via C–H activation.

It was also shown that carboxylic acids can be used as the nucleophilic component for this reaction. In 2020, B. Chen et al. applied this approach to aryloxazoline **387** with ortho-benzoylamino group, diazo ethyl acetoacetate, and a series of monocarboxylic acids **388**, which provided isoquinolones **389** under conditions similar to the previous work (Scheme 118) [121]. Remarkably, when authors used dicarboxylic adipic acid as reaction partner, no incorporation of the acid molecule into the target product was observed and isocoumarines **390** were isolated instead of isoquinolones **389**. This happened due to loss of the ethylamino fragment via rearrangement of oxazoline ring to cyclopropylaminocarbamoyl group and further displacement of aziridine molecule by nucleophilic attack of enol coming from CH-activation of arene and its coupling with diazo ketoester.

**Scheme 119:**
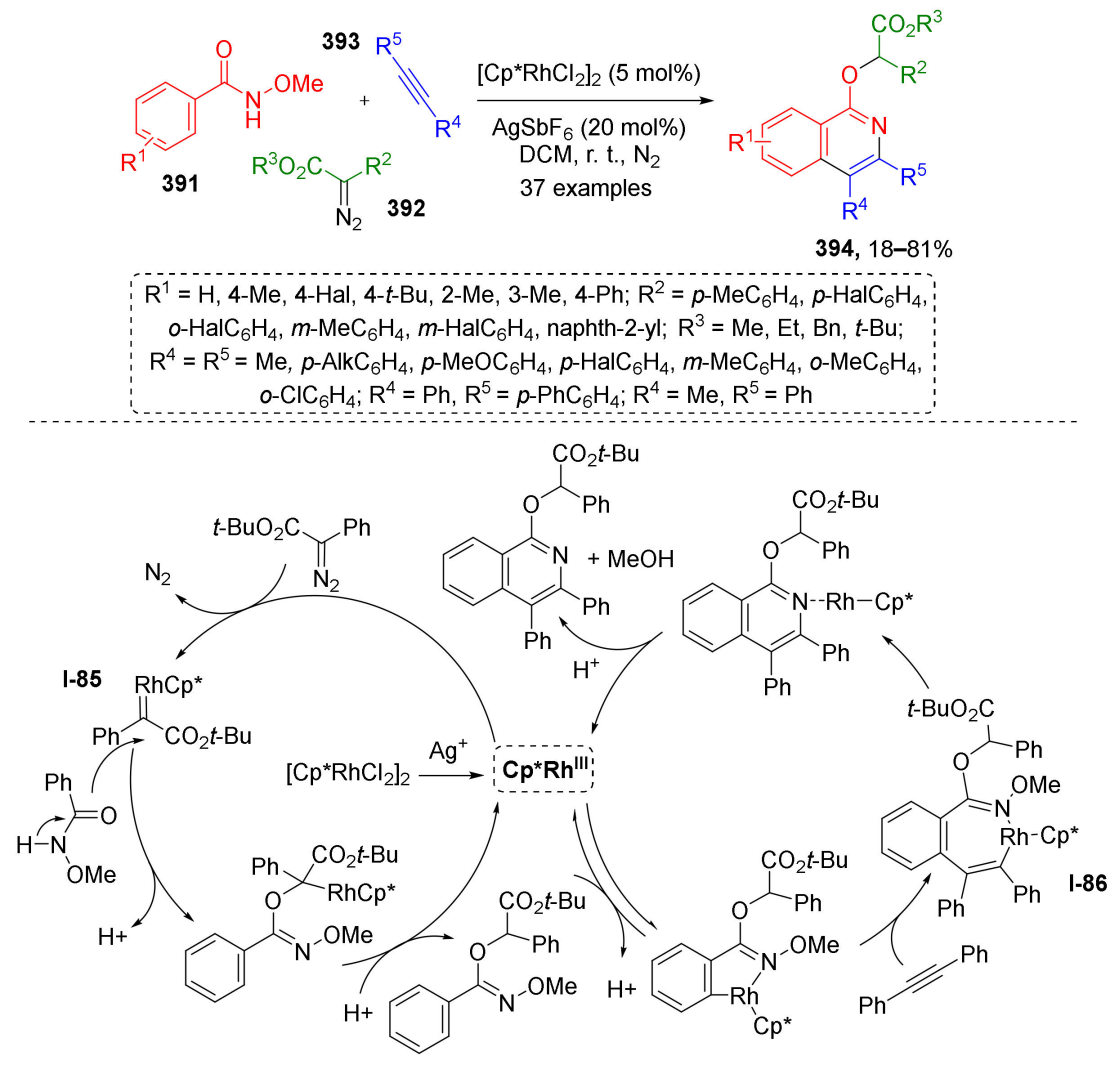
Three-component synthesis of isoquinoline derivatives from hydroxamic acids, diazo esters, and acetylenes by a relay catalysis.

CH-activation of acyclic *O*-alkyl hydroxamic acids which are close analogs of oxazolines has also recently been improved. In 2019, J. Wang and co-workers developed a three-component protocol for the preparation of *O*-substituted hydroxyisoquinolines **394** from hydroxamic acid **391**, diazo esters **392**, and diarylacetylenes **393** under dual rhodium/silver catalysis (Scheme 119) [122]. This reaction included *O*-alkylation of hydroxamic acid with Rh carbenoid **I-85** derived from diazo compound followed by Rh-mediated CH-activation of phenyl ring in this intermediate. A seven-membered rhodacycle **I-86** was then formed after insertion of acetylene molecule, followed by extrusion of Rh complex, which included the OMe group from the former hydroxamic acid moiety as one of the ligands thus forming the target isoquinoline molecule **394**. The reaction was found to be general regarding the substrate scope, but suffered from absence of regioselectivity when unsymmetrical diaryl acetylenes were used.

**Scheme 120:**
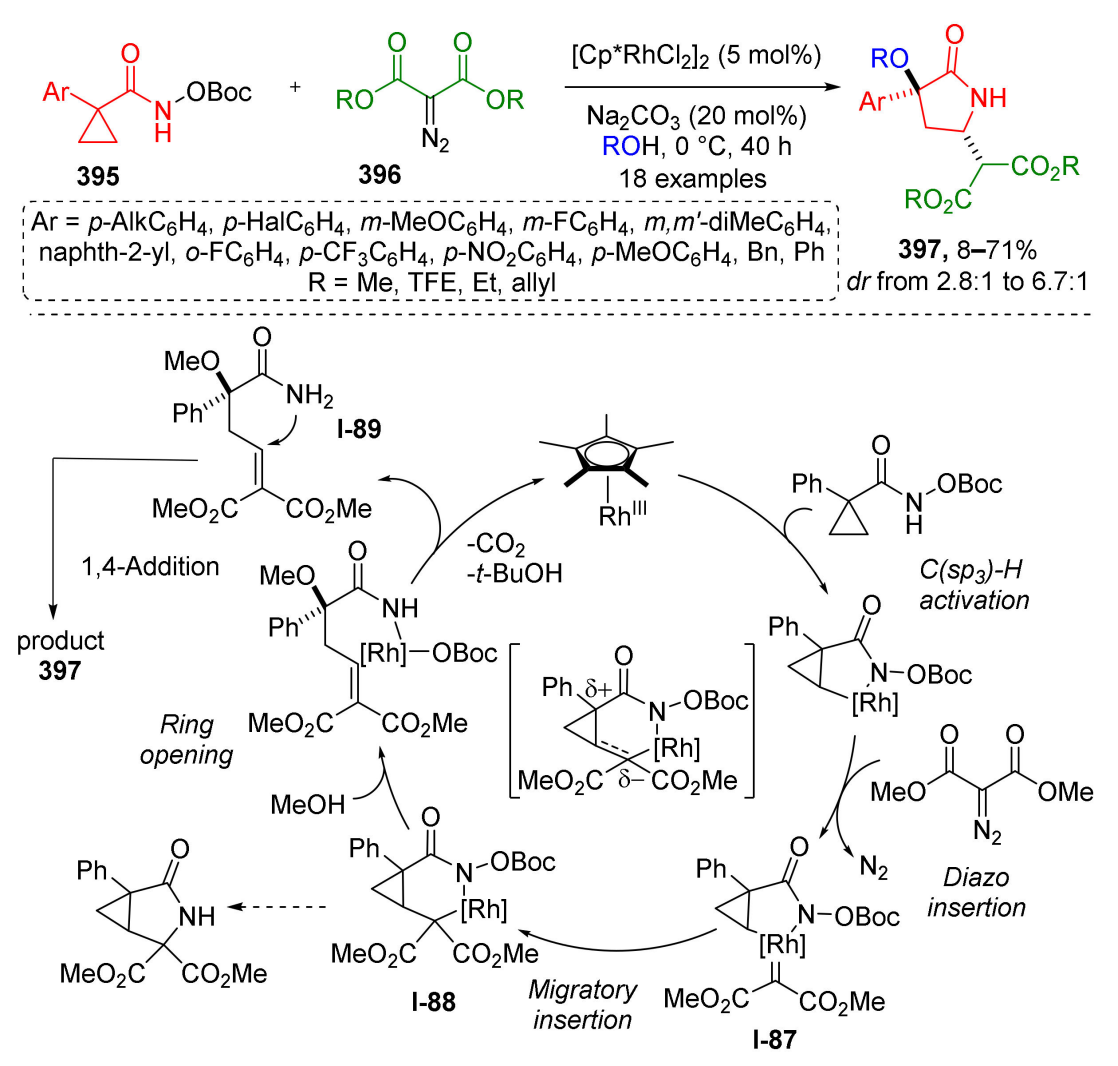
Rh(III)-catalyzed cyclopropane C−H/C−C activation sequence provides diastereoselective access to α-alkoxylated γ-lactams.

Not only an aromatic C_sp2_-H bond can be activated under rhodium catalysis. N. Cramer et al., in 2020, developed a three-component protocol for preparation of functionalized 2-pyrrolidones **397** from α-spirocyclic-*O*-Boc hydroxamic acids **395**, diazo malonates **396**, and primary alcohols (Scheme 120) [123]. This reaction started with coordination of hydroxamic acid and diazo compound to rhodium atom forming five-membered rhodacyclic carbenoid complex **I-87**. This process included C_sp3_-H bond activation in cyclopropyl moiety and was followed by migratory insertion to produce six-membered rhodacycle **I-88**. The latter was attacked by an alcohol molecule accompanied by cleavage of *N*-deprotected half-product **I-89** from the rhodium complex. The last step was intramolecular Michael addition of free amide NH_2_-group to the double bond of the acrylate system leading to 2-pyrrolidones **397**. The reaction proceeded with moderate diastereoselectivity.

## 7. Conclusions

In conclusion, we have summarized the results on developing multicomponent reactions involving diazo compounds published within the last five years. The type of MCR was analyzed by the way the components are combined and at which step the diazo reagent is involved, as well as the type of reaction intermediates—stable or reactive ones. Another point of our classification was the mechanism of the key step of the MCR.

The amount of discovered new publications in this area (at least 100) evidences their great attractiveness and potential. This is especially relevant for medicinal chemistry, since many of the developed approaches give new opportunities for diversity-oriented synthesis of biologically relevant scaffolds such as spirocyclic compounds, lactams, indolines, azepines, and others.

The majority of developed MCRs are based on denitrogenative transformations and generation of reactive species, ylides mostly, while combinations of reactants giving stable isolable intermediates are less common. From the mechanistic point of view, cycloadditions still prevail among reported approaches. Significant progress has been made in improving stereoselectivity of diazo-MCRs—at least one third of all discussed protocols describe enantioselective reactions and the most investigated are approaches to systems with chiral quaternary carbon atom. In terms of design of catalytic systems, it can be noted that many researchers shifted their focus from metal catalysis to organocatalysts or catalyst-free protocols, which is a big step forward towards cost-efficient and environmentally benign diazo MCRs. While most publications in the area of diazo MCRs describe synthesis of heterocyclic compounds, approaches to carbocyclic ones are still underdeveloped (only six publications were found). Additionally, it should be noted that MCRs with more than three components are still rare too—only five such protocols (4-CR) have been discovered during the literature search.

## Data Availability

Not applicable.
